# Mechanochemical Solvent‐Free Catalytic C−H Methylation

**DOI:** 10.1002/anie.202010202

**Published:** 2020-12-17

**Authors:** Shengjun Ni, Matic Hribersek, Swarna K. Baddigam, Fredric J. L. Ingner, Andreas Orthaber, Paul J. Gates, Lukasz T. Pilarski

**Affiliations:** ^1^ Department of Chemistry—BMC Uppsala University Box 576 75123 Uppsala Sweden; ^2^ Department of Chemistry—Ångström Laboratories Uppsala University Box 523 75120 Uppsala Sweden; ^3^ School of Chemistry University of Bristol Cantock's Close, Clifton Bristol BS8 1TS UK

**Keywords:** arenes, C−H functionalization, mechanochemistry, organometallics, rhodium

## Abstract

The mechanochemical, solvent‐free, highly regioselective, rhodium‐catalyzed C−H methylation of (hetero)arenes is reported. The reaction shows excellent functional‐group compatibility and is demonstrated to work for the late‐stage C−H methylation of biologically active compounds. The method requires no external heating and benefits from considerably shorter reaction times than previous solution‐based C−H methylation protocols. Additionally, the mechanochemical approach is shown to enable the efficient synthesis of organometallic complexes that are difficult to generate conventionally.

## Graphical abstract

Catalytic C−H methylation proceeds efficiently under solvent‐free, mechanochemical conditions, including the late‐stage functionalization of biologically active substrates and otherwise difficult‐to‐make metalacyclic complexes.

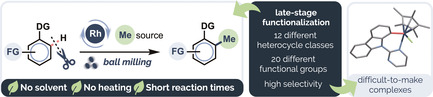

## Introduction

The methylation of bioactive molecules can dramatically improve their potency by enhancing lipophilicity, binding interactions, metabolic stability and numerous other properties (benefits collectively referred to as the “magic methyl effect”).^[1]^ Approximately 40 % of the 200 best‐selling drugs in 2019 contained a C−Me unit.^[2]^ New synthetic C−H methylation strategies have become highly sought after and significant recent efforts have been devoted to their discovery.^[1b]^ The use of transition metal catalysis for this purpose ranks among the most attractive of approaches^[3]^ but its potential is far from fully explored.

Despite their many benefits, a significant number of transition metal‐catalyzed C−H functionalizations (including C−H methylations) rely on toxic and/or environmentally damaging solvents, for example, 1,2‐dichloroethane (DCE), the legal regulation of which is becoming increasingly stringent.^[4]^ More generally, solvent waste presents a formidable challenge for the sustainability of chemical synthesis;^[5]^ in the pharmaceutical industry alone, an estimated 85 % of waste by mass is attributable to solvent use.^[6]^

In light of these concerns, mechanochemistry offers an enticing alternative to established, solution‐based approaches.^[7]^ The use of mechanical action (e.g. grinding or milling) for reagent mixing and activation can provide powerful advantages. These include shortened reaction times, lower operating temperatures, access to new mechanistic pathways,^[8]^ avoidance of solvent use and even the option of carrying out otherwise air‐sensitive reactions under aerobic conditions.^[9]^ For its potential to help usher in a greener era in synthesis, IUPAC recently listed “reactive extrusion” (mechanochemistry) among the top ten “chemical innovations that will change our world”.^[10]^ Mechanocatalytic C−H functionalization is a burgeoning area of research and has delivered some impressive recent advances.^[11]^ Here, we describe a mechanochemical catalytic C−H methylation that entirely avoids solvent as a reaction medium and that can be used even for the late‐stage functionalization (LSF) of bioactive molecules.

## Results and Discussion

Table 1 shows selected results from an optimization of our C−H methylation protocol using phenylpyridine (**1**) as a workhorse substrate. These reactions were performed in stainless steel (SS) milling vessels (14 mL internal volume) equipped with a single SS ball (10 mm diameter) using a mixer mill (MM) capable of oscillating at frequencies up to 36 Hz. To avoid neurotoxic C1 reagents such as MeI or SnMe_4_, and to encourage a broad substrate scope (e.g. by outcompeting oxidative addition to aryl halides), we began our study using MeB(OH)_2_ as the methyl source under oxidative conditions.^[12]^ [Cp*RhCl_2_]_2_ proved to be the only effective catalyst precursor^[13]^ amongst a variety we tested; others, including [RuCl_2_(*p*‐cymene)]_2_ and [Cp*Co(CO)I_2_],^[14]^ gave no conversion of the starting material. Initially, **1** was milled at 36 Hz for 40 min in the presence of [Cp*RhCl_2_]_2_ (5.0 mol %), MeB(OH)_2_ (2.0 equiv) and Ag_2_CO_3_ (1.2 equiv), which gave **2** in 62 % yield (entry 1). A lower loading of MeB(OH)_2_ gave a modestly lower yield, despite a doubled reaction time (entry 2). All other Ag^I^ salts we tested (Ag_2_O, AgOAc, and AgF) proved less effective than Ag_2_CO_3_ but outperformed Cu(OAc)_2_⋅H_2_O significantly (entries 3–5 vs. 6).^[15]^ A 2 h reaction time allowed us to keep the Ag_2_CO_3_ loading at 1.5 equiv and milling frequency at 30 Hz, and still obtain **2** in an improved yield of 77 % (entry 7 vs. entry 1). At 36 Hz the same conditions gave **2** in a 92 % yield (entry 8).

**Table 1:** Optimization of reaction conditions. 
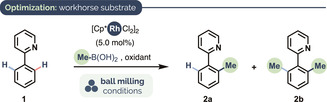

Entry^[a]^	MeB(OH)_2_ (equiv)	Oxidant (equiv)	Freq. [Hz], *T* [min]	Yield [%]^[b]^ (**2 a**/**2 b**)
1	2.0	Ag_2_CO_3_ (1.2)	36, 40	62 (96:4)
2	1.2	Ag_2_CO_3_ (1.2)	36, 80	54 (96:4)
3	2.0	Ag_2_O (1.2)	36, 40	54 (93:7)
4	2.0	AgOAc (2.4)	36, 40	17 (70:30)
5	2.0	AgF (2.4)	36, 40	53 (97:3)
6	2.0	Cu(OAc)_2_⋅H_2_O (1.2)	36, 60	5 (n.d.)
7	2.0	Ag_2_CO_3_ (1.5)	30, 120	77 (94:6)
8	2.0	Ag_2_CO_3_ (1.5)	36, 120	92 (89:11)

[a] All reactions shown were carried out in a mixer mill (MM) using a stainless steel (SS) milling vessel (14 mL internal vol.) equipped with one SS ball (10 mm diameter). 0.3 mmol scale. [b] Determined by ^1^H NMR spectroscopy using 1,3,5‐trimethoxybenzene as an internal standard.

Some notable advantages of the mechanochemical protocol emerged from these early experiments. First, reaction times of 1–2 h are substantially shorter than those used in related C−H methylations, for which 16–24 h is typical. Secondly, whilst low regioselectivity in the C−H functionalization of symmetrical substrates is a common challenge (usually, mono‐/difunctionalized product ratios fall in the range of 3–4:1), throughout our optimization the **2 a**/**2 b** ratio remained very high: up to an exceptional 97:3 (≈32:1, Table 1, entry 5).

**Scheme 1:**
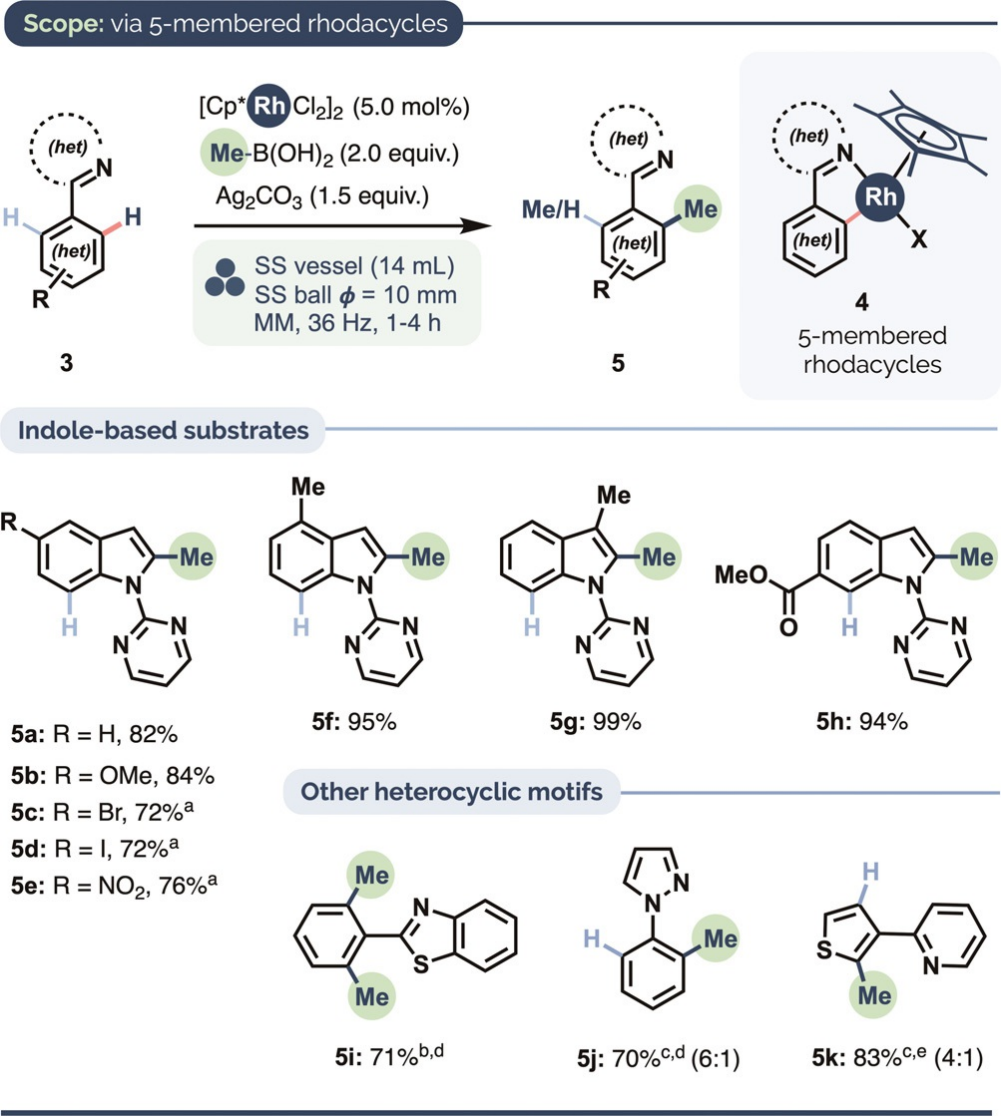
Heteroarene C−H methylation via five‐membered rhodacyclic intermediates. Conditions: 0.3 mmol scale, MeB(OH)_2_ (2.0 equiv), [Cp*RhCl_2_]_2_ (5.0 mol %), Ag_2_CO_3_ (1.5 equiv), SS vessel (14 mL internal vol.), one SS ball (10 mm diameter), 36 Hz. [a] Minor amounts of dimethylated products observed [b] 10 mol % catalyst loading. [c] Major/minor regioisomeric ratio shown in parentheses. [d] Conditions from Scheme 2. MeBF_3_K (6.0 equiv) [e] 1.2 equiv of MeB(OH)_2_ used.

We applied our optimized protocol to the C−H methylations of various heteroarenes expected to proceed via five‐membered rhodacyclic intermediates of type **4** (Scheme 1). Pyrimidine‐directed C2−H methylation of indoles[^11g^, ^16^] (products **5 a**–**5 h**) occurred in good to quantitative yields and with generally excellent C2−H regioselectivity. Electron‐rich (e.g. product **5 b**), electron‐poor (**5 e**, **5 h**) and potentially sterically hindered (**5 g**) substrates performed very well, as did those bearing C‐halogen units (**5 c**, **5 d**), which leaves open the prospect of their subsequent derivatization via coupling strategies. Our mechanochemical protocol also proved compatible with benzothiazole‐ (**5 i**) and pyrazole‐directed (**5 j**) C−H methylation; C2−H methylation of the thiophene ring system gave **5 k** in very good yield.

**Scheme 2:**
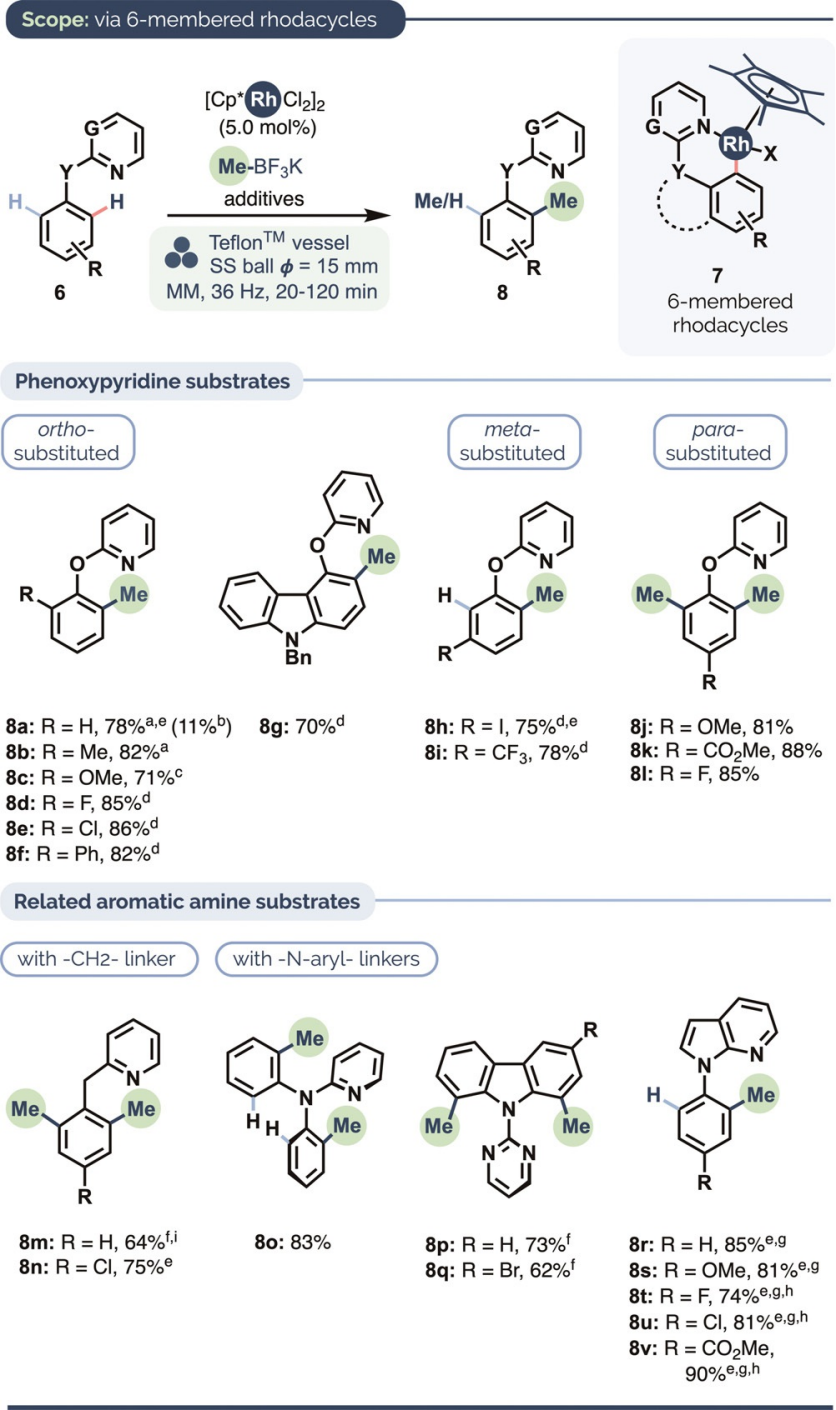
Heteroarene C−H methylation via six‐membered rhodacyclic intermediates. Conditions: 0.3 mmol scale, MeBF_3_K (6.0 equiv), [Cp*RhCl_2_]_2_ (5.0 mol %), AgSbF_6_ (20 mol %), Ag_2_CO_3_ (3.0 equiv), Teflon vessel, SS ball (15 mm diameter), 36 Hz, 2 h. [a] 2.0 equiv MeBF_3_K and 1.5 equiv Ag_2_CO_3_. [b] Yield using SS milling vessel under otherwise identical conditions. [c] 4.0 equiv MeBF_3_K and 2.5 equiv Ag_2_CO_3_. [d] 3.0 equiv MeBF_3_K and 1.5 equiv Ag_2_CO_3_. [e] Dimethylation observed as the minor product (see the Supporting Information for details). [f] 4.0 equiv MeBF_3_K. [g] 1.5 equiv MeBF_3_K and 1.5 equiv Ag_2_CO_3_. [h] 25 Hz. [i] [Cp*RhCl_2_]_2_ (10 mol %), AgSbF_6_ (40 mol %).

**Figure 1:**
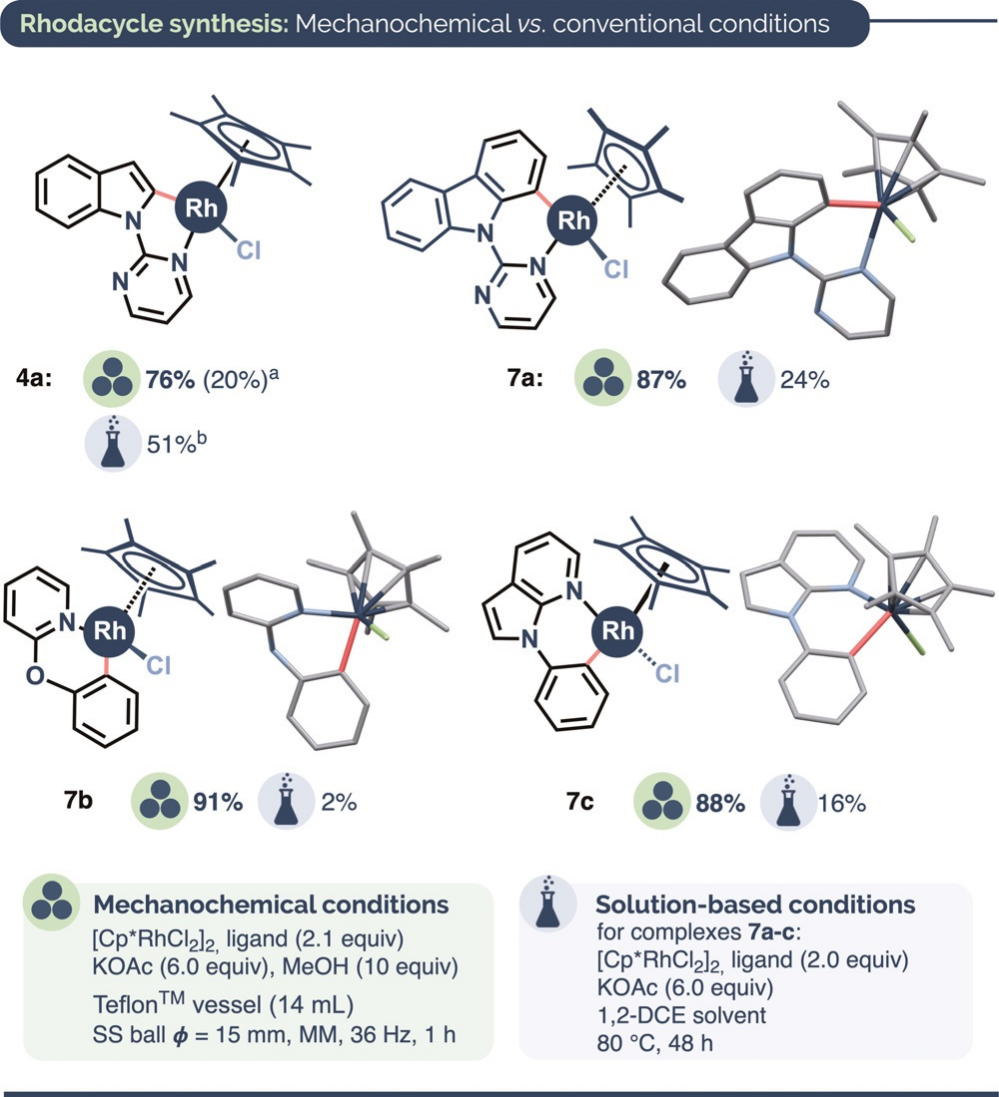
Comparison of mechanochemical (ball milling) and conventional solution‐based conditions for rhodacycle preparation. [a] Previously reported yield using ball milling at 30 Hz.^[11g]^ [b] Average yield from five previously reported syntheses using solution‐based conditions.^[33]^

Translating these conditions for substrates giving rise to six‐membered metallacycles (Scheme 2), whose formation is thermodynamically less favored, required re‐optimization: Me‐BF_3_K instead of MeB(OH)_2_ gave the best yields in the presence of substoichiometric AgSbF_6_. Unlike for the reactions in Scheme 1, Teflon milling vessels gave higher yields than their stainless steel counterparts (e.g. product **8 a**, Scheme 2). The influence of milling vessel material as a parameter is not yet fully understood in the context of mechanochemical reactions. The next part of our study thus examined phenoxypyridine substrates, which are useful as masked phenol surrogates^[17]^ as well as aryl pseudohalides,^[18]^ and which occur in numerous biologically active compounds (see Figure 1 below) and luminescent materials.^[19]^ These provided good yields with electron‐donating and electron‐withdrawing groups at *ortho* (**8 a**–**8 g**), *meta* (**8 h**–**i**) and *para* positions (**8 j**–**l**). The transformation also worked well when the ether linker was replaced with a ‐CH_2_‐ or ‐NAr‐ unit (products **8 m**–**n** and **8 o**, respectively), including as part of the carbazole core (**8 p**–**q,** *cf*. **8 g**); the pyridinoid ring of 7‐azaindole also proved able efficiently to direct the C−H methylation of both electron‐poor and electron‐rich arenes (**8 r**–**8 v**). These results indicate there is some leeway in the σ‐donating strength of the directing group.^[20]^

The new conditions also allowed the smooth C7−H methylation of indoline **9** to give **10** in 74 % yield at 25 Hz and 90 % at 36 Hz (entries 1 and 2, Table 2).^[21]^ With increased amounts of MeBF_3_K and Ag_2_CO_3_, however, we observed the formation of **11** in significant quantities for reactions run at 36 Hz, but not at 25 Hz (entries 3 and 4). Adjusting the catalyst loading allowed for conditions in which the exclusive formation of either **10** or **11** could be selected using only the milling frequency (entries 5 and 6). The C2−H methylation (to give **11**) presumably occurs via an intermediate 2,3‐dehydrogenation enabled by the additional energy input. Catalytic dehydrogenative aromatizations generally require very high temperatures and long reaction times;^[22]^ Rh‐catalyzed dehydrogenative aromatizations of indolines are rare^[23]^ whilst mechanochemical dehydrogenative aromatization as a whole is without precedent.^[24]^ Control experiments confirmed that both Ag_2_CO_3_ and Me‐BF_3_K are required for **11** to form. On the basis of this and studies on Ar−H activation by Cp*Rh^III^Me_2_L complexes,^[25]^ we tentatively suggest C(sp^3^)−H activation might occur via σ‐bond metathesis (**13** to **14**),^[26]^ which has been proposed for some related processes.^[27]^ The divergent outcomes obtained from different milling frequencies suggest an exciting new basis for regiocontrol in mechanochemical C−H functionalization.

**Table 2:** Frequency‐dependent selectivity in the C−H methylation of an indoline substrate. 
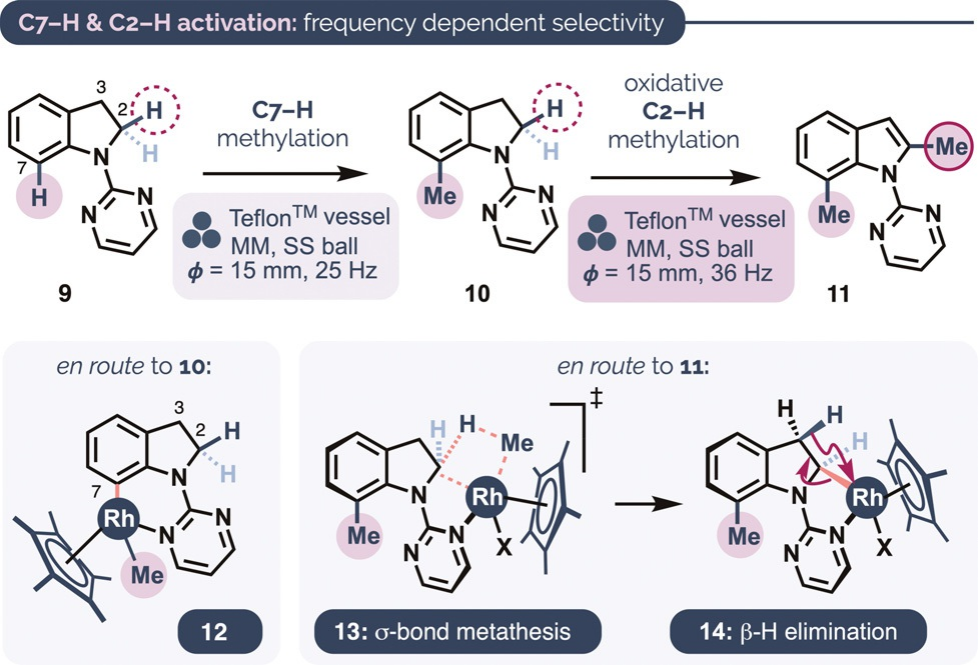

Entry^[a]^	[Cp*RhCl_2_]_2_/ AgSbF_6_ (mol %)	MeBF_3_K/ Ag_2_CO_3_ (equiv)	Freq. [Hz], *T* [h]	Yield [%] (**10**/**11**)
1	5.0/20	1.5/1.5	25, 2	74 (>99:1)
2	5.0/20	1.5/1.5	36, 2	90 (>99:1)^[b]^
3	5.0/20	6.0/3.0	25, 1	38 (>99:1)
4	5.0/20	6.0/3.0	36, 1	68 (59:41)
5	10/40	6.0/3.0	25, 1	75 (>99:1)
6	10/40	6.0/3.0	36, 1	60 (1:>99)

[a] All reactions shown were carried out at a 0.3 mmol scale using a 14 mL Teflon milling vessel equipped with one SS ball (15 mm diameter). [b] Determined by ^1^H NMR spectroscopy using 1,3,5‐trimethoxybenzene as an internal standard.

**Scheme 3:**
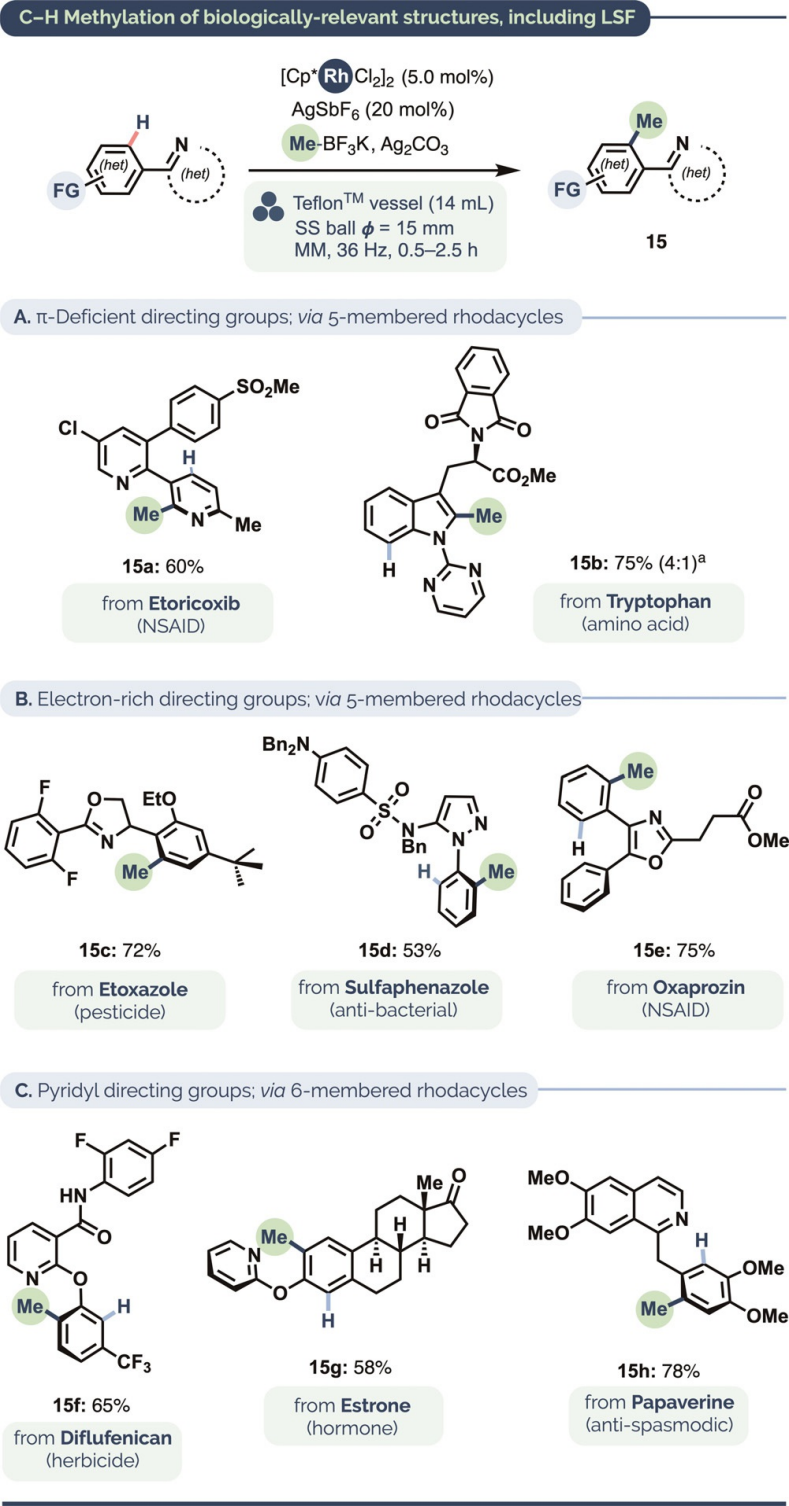
Mechanocatalytic C−H methylation of bioactive motifs, including late‐stage C−H methylation. For specific conditions, see Supporting Information. [a] Conditions from Scheme 1, 6 h. Ratio of mono‐ and dimethylated isomers (major isomer shown).

A hallmark advantage of C−H functionalization is its potential to diversify bioactive molecules at a late stage in a synthetic sequence. This can help obviate de novo syntheses, lower costs and expedite the exploration of chemical space.^[28]^ To the best of our knowledge, this study marks the first time catalytic LSF has been conducted under mechanochemical conditions. We tested the C−H methylation of a range of bioactive substrates, including those based on marketed pharmaceuticals (Etoricoxib, Sulfaphenazole, Oxaprozin and Papaverine), the herbicide Diflufenican and pesticide Etoxazole (Scheme 3) for all of which, reaction times ranged from 30 min to 2.5 h. Exclusively mono‐methylated products were isolated in every case (**15 a**, **15 c**–**15 h**) with the exception of tryptophan derivative **15 b**, which gave minor amounts of the C2,C7‐dimethylated product (not shown). The mechanochemical late‐stage methylation worked well with both π‐deficient and π‐rich directing groups, including pyrazoles^[29]^ and oxazoles,^[30]^ which are prevalent in a large number of bioactive compounds. In total, across our entire substrate range (Scheme 1, Scheme 2, Scheme 3 and, Table 2), our method was compatible with 12 different heterocycle types and 20 different pendant functional groups.

En route to mechanistic experiments, we compared the efficiency of mechanochemical and solution‐based methods for the synthesis of a range of rhodacyclic complexes based on ligands from our C−H methylation scope (Figure 1). It is noteworthy that solution‐based rhodacycle syntheses frequently rely on toxic solvents (e.g. DMF or DCE) and that yields for six‐membered metallacycles generated through C−H activation tend to be modest.^[31]^ The use of mechanochemistry in organometallic synthesis is a growing area of research and has provided routes to various previously inaccessible species.^[32]^ In our experiments, ball milling at 36 Hz outperformed conventional solution‐based methods for both five‐ (**4 a**) and six‐membered (**7 a**–**c**) rhodacycles by a yield margin of up to 89 %, and within a substantially shorter reaction time (1 h vs. 48 h). The structures of previously unreported six‐membered rhodacycles **7 a**–**c** were confirmed spectroscopically as well as by X‐ray crystallography (Figure 1).

We tested complexes **4 a** and **7 c** as C−H methylation catalysts in their own right. Using conditions from Scheme 1 with **4 a** instead of [Cp*RhCl_2_]_2_, product **5 a** was obtained in 92 % yield. Similarly, **7 c** as the catalyst gave **8 r** in 78 % using conditions from Scheme 2. These experiments are consistent with the putative intermediacy of rhodacycles in our mechanochemical reactions.

**Scheme 4:**
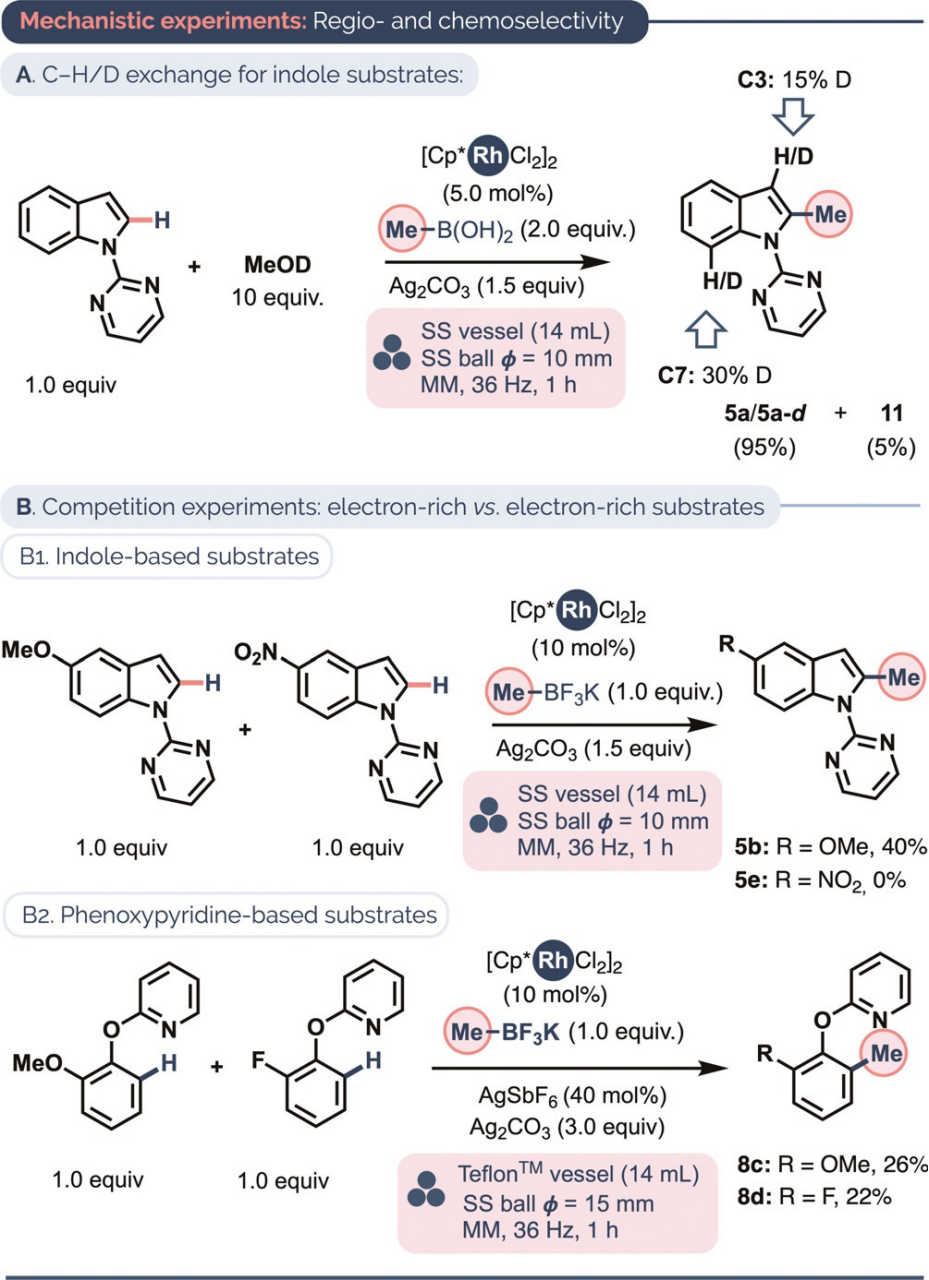
Mechanistic experiments probing the C−H activation step. Spectroscopic yields based on ^1^H NMR using 1,3,5‐trimethoxybenzene as a standard.

Several preliminary mechanistic insights may be inferred from the mechanochemical C−H methylation catalysis. First, although indole C2−H methylation occurs faster than does C7−H methylation at 36 Hz (Scheme 1, products **5 a**–**h**), C7−H methylation occurs even at 25 Hz when a C(sp^2^)2−H unit is unavailable (Table 2, **9** to **10**). Therefore, we reasoned that C7−H rhodation might occur reversibly at 36 Hz, prior to a more difficult transmetalation or oxidation step at the corresponding six‐membered rhodacycle. In line with this, a C−H/D exchange experiment using 1‐(pyrimidin‐2‐yl)‐1*H*‐indole in the presence of 10 equiv MeOD (Scheme 4 A) revealed substantial reversible C7−H activation, even when C2‐methylation is significantly faster. Additionally, competition experiments (Scheme 4 B) showed a strong preference for the methylation of electron‐rich indoles (products **5 b** vs. **5 e**) but not of electron‐rich phenoxypyridines (**8 c** vs. **8 d**), so it is possible that reactions proceeding via 5‐ and 6‐membered intermediates differ somewhat in the C–H rhodation step. That electron‐rich indoles outcompete their electron‐poor counterparts is suggestive of a S_E_Ar or eCMD metalation pathway.^[34]^ Studies by Bolm and co‐workers on a related mechanochemical Rh‐catalyzed C−H functionalization performed under oxidative conditions suggest that C−H rhodation is not the turnover‐limiting step.^[11f]^

Although C−H rhodation can occur readily without AgSbF_6_ present (Figure 1), the catalytic methylation reactions proceeding via six‐membered rhodacycles showed significantly improved yields on inclusion of AgSbF_6_. Plausibly, AgSbF_6_ facilitates the transmetalation or reductive elimination step (or both) when six‐membered rhodacyclic intermediates play a part. This, and the greater efficiency of reactions run with Me‐BF_3_K,^[35]^ suggests that transmetalation to the Rh^III^ center of six‐membered rhodacyclic intermediates might be turnover‐limiting.

In line with this, transmetalation to **7 c** and subsequent reductive elimination required AgSbF_6_ or KPF_6_ to proceed at all (entry 1 vs. entries 2 & 3, Table 3). Even without Ag_2_CO_3_ present, conversion from **7 c** to **8 r** was higher when **16 a** was included as an additive, which we used to mimic the presence of additional substrates in the reaction mixture (entry 4). By contrast, the addition of **16 b** did not affect the yield (entry 2 vs. entry 5). This is similar to our findings for oxidative Ru‐catalyzed C−H arylations using organoboronates,^[16b]^ for which we previously proposed that unreacted substrates may play a role as ancillary/stabilizing ligands in the cycle(s) prior to their own C−H functionalization.

**Table 3:** Effect of different additives on the efficiency of transmetalation to and reductive elimination from complex **7 c**. 
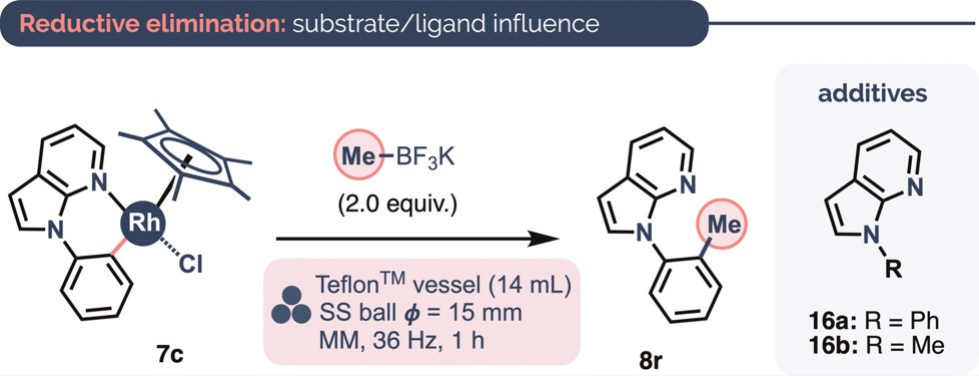

Entry	Additives (equiv)	*T* [min]	Yield [%]
1	None	60	0
2	AgSbF_6_ (1.0)	20	20
3	KPF_6_ (1.0)	20	9
4	AgSbF_6_ (1.0) + **16 a** (1.0)	20	33
5	AgSbF_6_ (1.0) + **16 b** (1.0)	20	20

Yields determined by ^1^H NMR spectroscopy using 1,3,5‐trimethoxybenzene as an internal standard.

## Conclusion

We have described the first catalytic C−H methylation reaction that proceeds under solvent‐free, mechanochemical conditions. Its benefits include high regioselectivity, short reaction times and a broad functional‐ and directing‐group tolerance that encompasses 12 important heterocycle classes and 20 different pendant functional groups. Notably, the reaction can be used for the late‐stage methylation of more complex, biologically active compounds—the first reported examples of catalytic late‐stage C−H functionalization carried out under mechanochemical conditions. We have also described the considerable superiority of ball milling for the synthesis of five‐ and especially six‐membered rhodacyclic species that are difficult to generate conventionally.

## Conflict of interest

The authors declare no conflict of interest.

## Supporting information

As a service to our authors and readers, this journal provides supporting information supplied by the authors. Such materials are peer reviewed and may be re‐organized for online delivery, but are not copy‐edited or typeset. Technical support issues arising from supporting information (other than missing files) should be addressed to the authors.

Supplementary
