# Supplementary material for: Mechanochemical Solvent‐Free Catalytic C−H Methylation
Source: Angew Chem Int Ed Engl. 2020 Dec 17;60(12):6660–6. doi: 10.1002/anie.202010202 (PMC7986365; doi:10.1002/anie.202010202)

Angewandte Chemie
Eine Zeitschrift der Gesellschaft Deutscher Chemiker

Supporting Information

# Mechanochemical Solvent-Free Catalytic C–H Methylation

*Shengjun Ni, Matic Hribersek+, Swarna K. Baddigam+, Fredric J. L. Ingner, Andreas Orthaber, Paul J. Gates, and Lukasz T. Pilarski**

anie_202010202_sm_miscellaneous_information.pdf

# Supporting Information


1. General information S2
2. Origin and synthesis of starting materials S3
   A. Known starting materials S3
   B. Previously unreported starting materials S7
3. C–H Methylation reactions S9
   A. Methylation products via 5-membered rhodacycles S10
   B. Methylation products via 6-membered rhodacycles S14
   C. Late stage C–H methylation reactions S23
4. Synthesis of organometallic complexes S27
5. Mechanistic experiments S29
6. Crystallographic data S33
7. References S36
8. NMR spectra S37

# 1. General information

Mechanochemical reactions were carried out using an InSolido Technologies IST636 mixer mill. Reaction vessels and milling medium used for mechanochemical reactions were purchased either from InSolido Technologies (Teflon$^{TM}$ vessels, 14 mL) or FormTech Scientific (stainless steel (SS) vessels, 14 mL). The average masses of SS balls used in the milling reactions were:

- 10 mm diameter: mean weight of 16 balls: 3.94 g (range between 3.59-4.02 g).
- 15 mm diameter: mean weight of 4 balls: 13.62 g (range between 13.50g to 13.67g).

1,3,5-Trimethoxybenzene or naphthalene internal standard was prepared as a stock solution (0.1 M in EtOAc). Starting materials were bought commercially and used without further purification or synthesized according reported literature procedures.

1,2-Dichloroethane (DCE), 1,4-dioxane and toluene were dried over 4 Å molecular sieves and stored under argon prior to use. EtOH and $H_2O$ were degassed with argon for half an hour before use. $CDCl_3$ was stored over $K_2CO_3$ to neutralize residual acid. The other solvents were obtained commercially and used "as is" without further purification.

Unless otherwise stated, all the reactions were carried out under normal atmospheric conditions. Thin-layer chromatography (TLC) was carried out using aluminium backed plates coated with silica gel 60 (0.20 mm, UV 254) and visualized under ultraviolet light ($\lambda$ = 254 nm) or with $KMnO_4$ staining solution. Purification by column chromatography was performed using silica gel 60 H (particle size 0.063−0.100 mm) or aluminium oxide 60 (particle size 0.063-0.200 mm, active basic). $^{1}H$, $^{13}C$ and $^{19}F$ NMR spectra were recorded on a Varian Unity 400 MHz ($^{1}H$ 400 MHz, $^{13}C$ 101 MHz, $^{19}F$ 376 MHz) or Bruker Avance 500 MHz ($^{1}H$ 500 MHz, $^{13}C$ 125 MHz). Chemical shifts are reported in ppm and referenced indirectly to tetramethylsilane via residual solvent signals ($^{1}H$: $CDCl_3$ at 7.26 ppm, DMSO-$d_6$ at 2.50 ppm, $^{13}C$: $CDCl_3$ at 77.0 ppm, DMSO-$d_6$ at 39.5 ppm; $^{19}F$ chemical shifts were calibrated to an external standard ($CFCl_3$) at 0.00 ppm). Multipicities are reported as: s = singlet, d = doublet, t = triplet, q = quartet, m = multiplet.

High-resolution electrospray ionisation mass spectrometry was performed on a micrOTOF II Focus instrument (Bruker Daltonics, Coventry, UK). High-resolution nanospray ionisation was performed on a Synapt G2S instrument (Waters, Manchester, UK) using a Triversa chip based nanospray source (Advion Biosciences, Norwich, UK). High-resolution electron ionisation mass spectrometry was performed at 70eV on a QExactive GC Orbitrap instrument (Thermo Scientific, Hemel Hempstead, UK). APCI was recorded on an Advion Expression$^{L}$ CMS with positive fragmentation.

# 2. Origin and Synthesis of Starting Materials

## A. Known starting materials

The following compounds were made using previously reported procedures:

- [Cp*RhCl$_2$]$_2$[1]
- 1-(pyrimidin-2-yl)-1*H*-indole[2]
- 5-methoxy-1-(pyrimidin-2-yl)-1*H*-indole[2]
- 5-bromo-1-(pyrimidin-2-yl)-1*H*-indole[2]
- 5-iodo-1-(pyrimidin-2-yl)-1*H*-indole[3]
- 5-nitro-1-(pyrimidin-2-yl)-1*H*-indole[4]
- 4-methyl-1-(pyrimidin-2-yl)-1*H*-indole[2]
- 3-methyl-1-(pyrimidin-2-yl)-1*H*-indole[2]
- Methyl-1-(pyrimidin-2-yl)-1*H*-indole-6-carboxylate[5]
- 2-phenylbenzo[*d*]thiazole[6]
- 2-phenoxypyridine[7]
- 2-(*o*-tolyloxy)pyridine[7]
- 2-(2-methoxyphenoxy)pyridine[7]
- 2-(2-fluorophenoxy)pyridine[8]
- 2-(2-chlorophenoxy)pyridine[7]
- 2-([1,1'-biphenyl]-2-yloxy)pyridine[9]
- 2-(4-methoxyphenoxy)pyridine[7]
- methyl 4-(pyridin-2-yloxy)benzoate[7]
- 2-(4-fluorophenoxy)pyridine[7]
- 9-(pyrimidin-2-yl)-9*H*-carbazole[10]
- 1-(pyrimidin-2-yl)indoline[11]
- methyl 3-(4,5-diphenyloxazol-2-yl)propanoate[12]
- methyl-(*R*)-2-(1,3-dioxoisoindolin-2-yl)-3-(1-(pyrimidin-2-yl)-1*H*-indol-3-yl)propanoate[13]
- 1-methyl-1H-pyrrolo[2,3-*b*]pyridine.[14]

Potassium methyltrifluoroborate[15a]

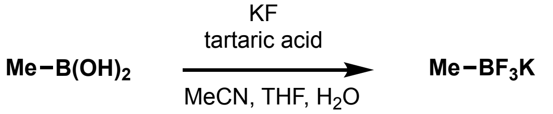


To a suspension of the methylboronic acid (1.20 g, 20.0 mmol) in MeCN (80 mL) at rt was added potassium fluoride (5.23 g, 90.0 mmol, 4.5 equiv.) in $H_2O$ (9.0 mL). The mixture was stirred until complete dissolution of the boronic acid. L-(+)-tartaric acid (7.51 g, 50.0 mmol, 2.5 equiv.) was dissolved into THF (38 mL) and added dropwise to the rapidly stirring biphasic mixture over the course of 1 min. A white precipitate formed that flocculated over a period of 5 min. The mixture was stirred for 30 min and filtered. The flask and filter cake were rinsed with further portions of MeCN (3 × 100 mL). The combined filtrates were concentrated under reduced pressure and the resulting colorless solid was dried under vacuum to give analytically pure $MeBF_3K$ (2.170 g, 89%). Spectra are in agreement with previously reported data.[15]

2-(Thiophen-3-yl)pyridine

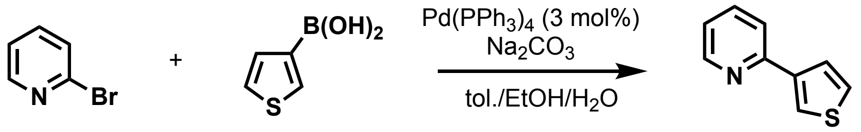


To a solution of 2-bromopyridine (1.03 g, 6.5 mmol, 1.0 equiv.) in toluene (24 mL), EtOH (5 mL) and $H_2O$ (24 mL) was added $Na_2CO_3$ (5.17 g, 48.8 mmol, 7.5 equiv.) followed by $Pd(PPh_3)_4$ (0.225 g, 0.20 mmol, 3 mol%) and thiophen-2-ylboronic acid (1.080 g, 8.5 mmol, 1.3 equiv.) under Ar. The mixture was refluxed for 17 h, and then cooled to rt. To the reaction mixture were added $H_2O$ (50 mL) and $CH_2Cl_2$ (100 mL). The layers were separated and the aqueous component was further extracted with $CH_2Cl_2$ (3 × 100 mL). The combined organic layers were washed with brine (200 mL) and dried over anhydrous $Na_2SO_4$. After removal of the solvent under reduced pressure, the residue was purified by silica-gel column chromatography (pentane/EtOAc = 4:1) to give the compound as a light yellow solid (0.780 g, 74%). Spectra are accordance with the previously reported data.[16]

2-(3-Iodophenoxy)pyridine

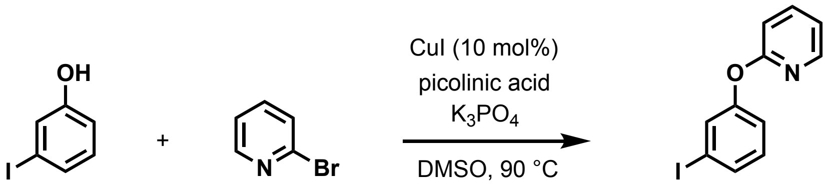


To a 25 mL Schlenk tube, were successively added 3-iodophenol (0.660 g, 3.0 mmol, 1.0 equiv.), 2-bromopyridine (0.569 g, 3.6 mmol, 1.2 equiv.), CuI (0.057 g, 0.3 mmol, 10 mol%), picolinic acid (0.074 g, 0.6 mmol, 20 mol%.), $K_3PO_4$ (1.27 g, 6.0 mmol, 2.0 equiv.) and DMSO (6.0 mL). The tube was evacuated and backfilled with argon carefully three times. The reaction mixture was stirred at 90 °C until completion. The reaction mixture was cooled down. Then water (10 mL) was added and extracted with EtOAc (3 × 10 mL). The combined organic phases were then washed with water (3 × 50 mL) and brine (50 mL) dried over anhydrous $Na_2SO_4$ and filtered. After removal of the solvent under reduced pressure, the residue was purified by silica-gel column chromatography (pentane/EtOAc = 40:1) to afford the desired product as a colorless solid (0.713 g, 80%). Spectra are in accordance with previously reported data.[17]

3-Bromo-9-(pyrimidin-2-yl)-9*H*-carbazole

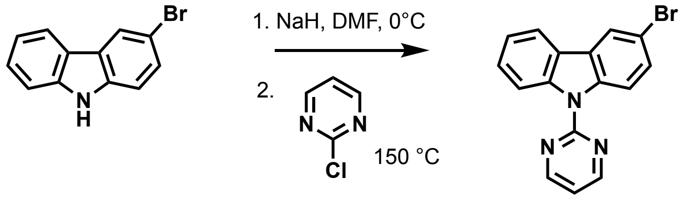


NaH (60% dispersion in mineral oil, 0.400 g, 10.0 mmol, 2.0 equiv.) was added to a stirring solution of 3-bromo-9*H*-carbazole (1.23 g, 5.0 mmol, 1.0 equiv.) in DMF (5.0 mL) at 0 °C. After stirring for 1 hour at 0 °C, 2-chloropyrimidine (1.15 g, 10.0 mmol, 2.0 equiv.) was added and the mixture was stirred at 150 °C for 21 h. The reaction mixture was then cooled to rt, poured onto ice and extracted with EtOAc (3 × 100 mL). The organic extract was washed

successively with $H_2O$ (2 × 200 mL) and brine (200 mL). The residue was dried over anhydrous $Na_2SO_4$, filtered and concentrated under reduced pressure. After the solvent was removed, the crude product was purified by silica-gel column chromatography (pentane/$CH_2Cl_2$ = 1:1) to afford the final product as a colorless solid (1.17 g, 78%). Spectra are in accordance with previously reported data.[11]

(8*R*,9*S*,13*S*,14*S*)-13-Methyl-3-(pyridin-2-yloxy)-6,7,8,9,11,12,13,14,15,16-decahydro-17*H*-cyclopenta[*a*]phenanthren-17-one

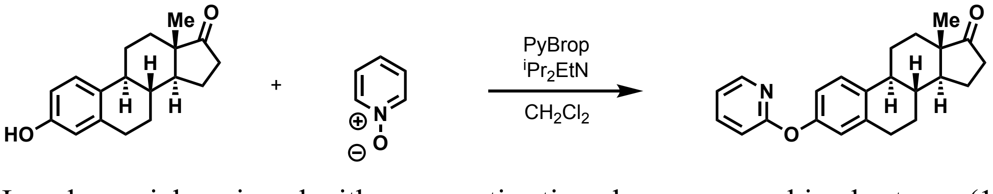


In a dram vial equipped with a magnetic stirrer bar were combined estrone (1.00 g, 3.7 mmol, 1.0 equiv.), $^iPr_2EtN$ (2.4 mL, 13.8 mmol, 3.75 equiv.) and $CH_2Cl_2$ (9.6 mL). The solution was treated with pyridine-*N*-oxide (0.390 g, 4.7 mmol, 1.1 equiv.), followed by bromotripyrrolidinophosphonium hexafluorophosphate (2.20 g, 4.8 mmol, 1.3 equiv.). The vial was capped and stirred at rt overnight. After complete starting material consumption (TLC analysis), the reaction was poured into saturated sodium bicarbonate solution (50 mL) and extracted with $CH_2Cl_2$ (3 × 100 mL). The combined organic phases were washed with brine (100 mL), dried over anhydrous $Na_2SO_4$ and filtered. The solvent was removed under reduced pressure and the crude material was purified by silica-gel column chromatography (pentane/EtOAc = 3:1) to afford the title compound as a colourless solid (0.841 g, 65%). Spectra are in accordance with previously reported data.[18]

Syntheses of 7-azaindole derivatives:

The following were synthesized according to a literature procedure:[19]

- 1-Phenyl-1*H*-pyrrolo[2,3-*b*]pyridine
- 1-(4-methoxyphenyl)-1*H*-pyrrolo[2,3-*b*]pyridine
- 1-(4-fluorophenyl)-1*H*-pyrrolo[2,3-*b*]pyridine
- 1-(4-chlorophenyl)-1*H*-pyrrolo[2,3-*b*]pyridine
- Methyl 4-(1*H*-pyrrolo[2,3-*b*]pyridin-1-yl)benzoate

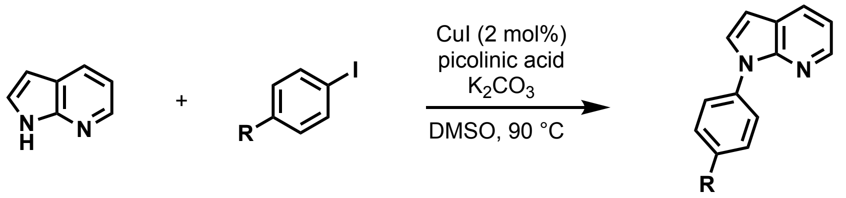


To a 25 mL Schlenk tube, were successively added the iodoarene (3.0 mmol, 1.0 equiv.), 7-azaindole (0.425 g, 3.6 mmol, 1.2 equiv.), CuI (0.011 g, 0.06 mmol, 2 mol%), $K_2CO_3$ (1.040 g 7.5 mmol, 2.5 equiv.), picolinic acid (0.015 g, 0.12 mmol, 20 mol%) and DMSO (6.0 mL). Then evacuated the tube and backfilled with argon carefully for three times. The reaction mixture was stirred at 90 °C until complete conversion of the starting material (TLC analysis). The reaction mixture was cooled to rt and $H_2O$ (10 mL) was added and followed by extracted

with EtOAc (3 × 10 mL). The organic phase was then washed with $H_2O$ (3 × 50 mL) and brine (50 mL), dried over anhydrous $Na_2SO_4$ and filtered. After removal of the solvent under reduced pressure, the residue was purified by silica-gel column chromatography. Spectra were in accordance with previously reported data.[20]

*N*-phenylpyridin-2-amine and *N*,*N*-diphenylpyridin-2-amine

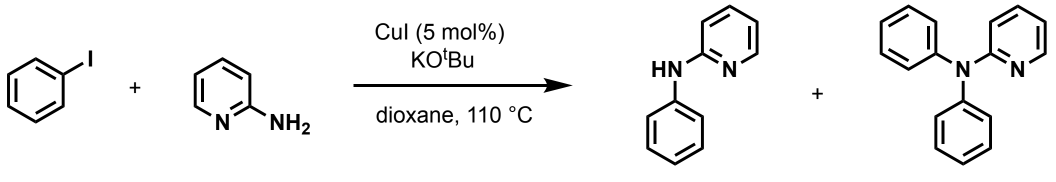


To a 25 mL Schlenk tube, were successively added pyridin-2-amine (0.471 g, 5.0 mmol, 1.0 equiv.), CuI (0.048 g, 0.3 mmol, 5 mol%), KO$^t$Bu (0.729 g, 6.5 mmol, 1.3 equiv.). The tube was evacuated and backfilled with Ar three times. Dioxane (7.5 mL) and iodobenzene (1.122 g, 5.5 mmol, 1.1 equiv.) were added under an Ar atmosphere. The mixture was stirred at 110 °C until complete consumption of the starting material (TLC analysis). The mixture was cooled to rt extracted with $Et_2O$ (3 × 10 mL). After removal of the solvent under reduced pressure, the residue was purified by silica-gel column chromatography (pentane/EtOAc = 20:1, then 5:1) to afford *N*-phenylpyridin-2-amine as a light yellow solid (0.400 g, 59%) and *N*,*N*-diphenylpyridin-2-amine as a light yellow solid (0.210 g, 22%). Spectra were in accordance with previously reported data.[21]

Etoricoxib

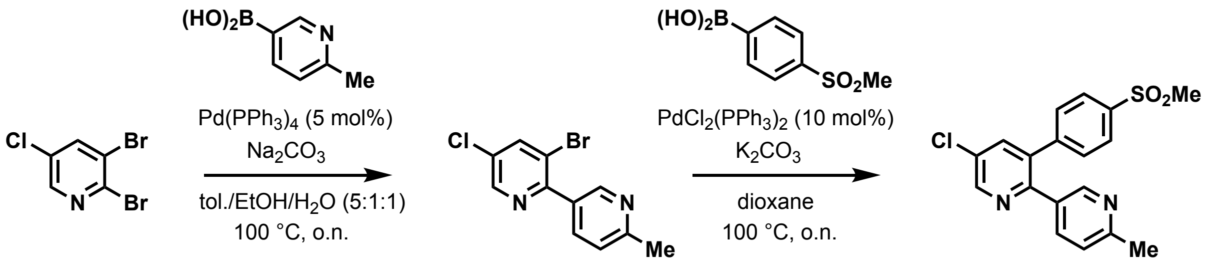


A 100 mL round bottom flask was filled with 2,3-dibromo-5-chloropyridine (0.814 g, 3 mmol, 1.0 equiv.), (6-methylpyridin-3-yl)boronic acid (0.452 g, 3.3 mmol, 1.1 equiv.), $Pd(PPh_3)_4$ (0.173 g, 0.15 mmol, 5 mol%), and $Na_2CO_3$ (0.954 g, 9.0 mmol, 3.0 equiv.), then evacuated and backfilled with Ar three times. EtOH (5 mL), $H_2O$ (5 mL) and toluene (25 mL) were added and the reaction mixture was stirred at 100 °C overnight at which point the starting material was fully consumed (TLC analysis). The mixture was extracted with EtOAc (3 × 10 mL), dried over $Na_2SO_4$ and concentrated under reduced pressure. The residue was further purified by silica-gel column chromatography (pentane/EtOAc = 10:1, then 5:1), to give 3-bromo-5-chloro-6'-methyl-2,3'-bipyridine as a light yellow solid (0.560 g, 66%). $^1$H NMR (400 MHz, $CDCl_3$) $\delta$ 8.84 (s, 1H), 8.60 (s, 1H), 8.02 (s, 1H), 7.90 (d, $J$ = 8.0 Hz, 1H), 7.25 (d, $J$ = 8.0 Hz, 1H), 2.63 (s, 3H). $^{13}$C NMR (101 MHz, $CDCl_3$): 159.0, 153.6, 149.5, 147.3, 140.6, 137.0, 131.4, 131.0, 122.4, 119.6, 24.4. HRMS-ESI: calcd for $C_{11}H_9BrClN_2$ $[M+H]^+$, 282.9632; found, 282.9636.

To a mixture of 3-bromo-5-chloro-6'-methyl-2,3'-bipyridine (0.284 g, 1 mmol, 1.0 equiv.), (4-(methylsulfonyl)phenyl)boronic acid (0.400 g, 2.0 mmol, 2.0 equiv.), $Pd(PPh_3)_2Cl_2$ (0.073 g,

0.1 mmol, 10 mol%) and $K_2CO_3$ (0.415 g, 3.0 mmol, 3.0 equiv.) was added dioxane (6.0 mL). The reaction was stirred at 100 °C overnight until the starting material was fully consumed (TLC analysis). The reaction mixture was cooled to rt, diluted with $H_2O$ (20 mL) and extracted with EtOAc (3 × 20 mL). Then the organic layer was dried over $Na_2SO_4$, filtered and concentrated under reduced pressure. The residue was purified by silica-gel column chromatography ($CH_2Cl_2$/MeOH = 100:1, then 50:1), leading to the final pure product as a colorless solid (0.192 g, 54%). Spectra were in accordance with previously reported data.[18]

## B. Previously unreported starting materials

2-(3-(Trifluoromethyl)phenoxy)pyridine

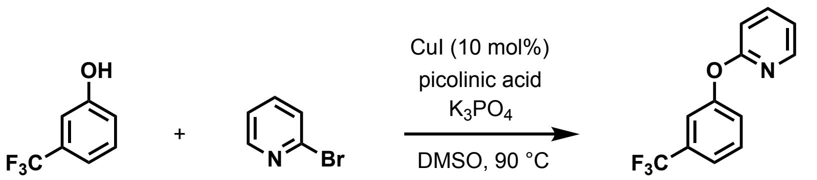


To a 25 mL Schlenk tube, were successively added 3-(trifluoromethyl)phenol (0.486 g, 3.0 mmol, 1.0 equiv.), 2-bromopyridine (0.569 g, 3.6 mmol, 1.2 equiv.), CuI (0.057 g, 0.3 mmol, 10 mol%), picolinic acid (0.074 g, 0.6 mmol, 20 mol%.), $K_3PO_4$ (1.274 g, 6.0 mmol, 2.0 equiv.) and DMSO (6.0 mL). The tube was then evacuated and backfilled with Ar three times. The reaction mixture was stirred at 90 °C until complete consumption of starting materials (monitored by $^1$H NMR). The reaction mixture was cooled to rt, diluted with $H_2O$ (10 mL) and extracted with EtOAc (3 × 10 mL). The combined organic phases were then washed with water (3 × 50 mL) and brine (50 mL), dried over anhydrous $Na_2SO_4$ and filtered. After removal of the solvent under reduced pressure, the residue was purified by silica-gel column chromatography (pentane/EtOAc = 40:1) to afford the title product as a colorless solid (0.666 g, 93%). $^1$H NMR (400 MHz, $CDCl_3$) $\delta$ 8.20 (dd, $J$ = 5.1, 2.0 Hz, 1H), 7.73 (ddd, $J$ = 8.3, 7.2, 2.0 Hz, 1H), 7.51 (dd, $J$ = 7.9, 7.9 Hz, 1H), 7.45 (d, $J$ = 7.9 Hz, 1H), 7.42 (s, 1H), 7.34 (d, $J$ = 7.9 Hz, 1H), 7.05 (ddd, $J$ = 7.2, 5.0, 1.0 Hz, 1H), 6.98 (dd, $J$ = 8.2, 0.9 Hz, 1H). $^{19}$F NMR (376 MHz, $CDCl_3$) $\delta$ -62.68 (s). $^{13}$C NMR (101 MHz, $CDCl_3$): $\delta$ 163.0, 154.3, 147.7, 139.8, 132.1 (q, $J$ = 32.6 Hz), 130.1, 124.6, 123.7 (q, $J$ = 272.4 Hz), 121.2 (q, $J$ = 3.9 Hz), 119.1, 118.2 (q, $J$ = 3.8 Hz), 112.0. HRMS-ESI: calcd for $C_{12}H_9F_3NO$ [M+H]$^+$, 240.0631; found, 240.0633.

9-Benzyl-4-(pyridin-2-yloxy)-9*H*-carbazole

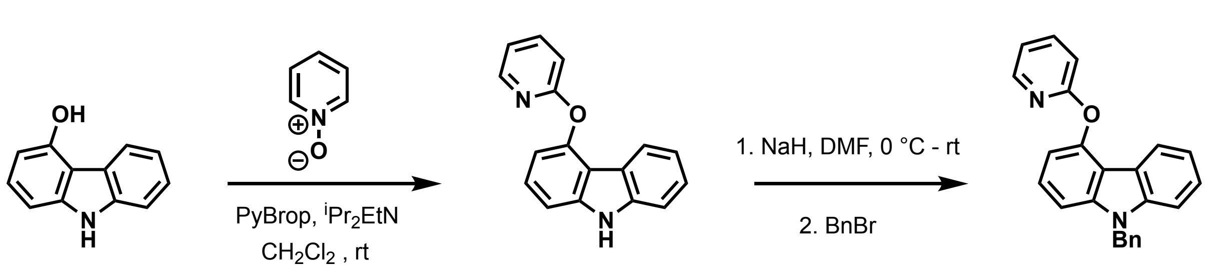


To a suspension of 4-hydroxycarbazole (0.037 g, 0.20 mmol, 1.0 equiv.), pyridine-*N*-oxide (0.023 g, 0.24 mmol, 1.2 equiv.), PyBrop (0.121 g, 0.26 mmol, 1.3 equiv.) in $CH_2Cl_2$ (0.4 mL)

was added $^iPr_2EtN$ (139 μL, 0.8 mmol, 4.0 equiv.). The solution was stirred at rt until complete consumption of starting materials (TLC analysis). The reaction mixture was diluted with saturated aqueous $NaHCO_3$ (5.0 mL) and extracted with $CH_2Cl_2$ (3 × 5 mL). The combined organic layers were washed with brine (15 mL), dried over anhydrous $Na_2SO_4$ and filtered. After removal of the solvent under reduced pressure, the residue was purified by silica-gel column chromatography (pent/EtOAc = 4:1) to afford 4-(pyridin-2-yloxy)-9*H*-carbazole as a colorless solid (0.044 g, 85%). $^1H$ NMR (400 MHz, DMSO-$d_6$) $\delta$ 11.46 (br s, 1H), 8.09 (dd, $J$ = 4.9, 2.0 Hz, 1H), 7.87 (ddd, $J$ = 8.8, 7.1, 2.1 Hz, 1H), 7.64 (d, $J$ = 7.8 Hz, 1H), 7.48 (d, $J$ = 8.1 Hz, 1H), 7.44 – 7.28 (m, 3H), 7.18 – 7.08 (m, 2H), 7.06 – 6.97 (m, 1H), 6.89 (dd, $J$ = 7.2, 1.4 Hz, 1H). $^{13}C$ NMR (101 MHz, DMSO-$d_6$): $\delta$ 163.2, 148.5, 147.6, 141.7, 140.2, 139.5, 126.2, 125.4, 121.7, 120.3, 118.8, 115.3, 111.2, 110.9, 110.7, 107.8. HRMS-ESI: calcd for $C_{17}H_{12}N_2NaO$ $[M+Na]^+$, 283.0842; found, 283.0843.

To a solution of 4-(pyridin-2-yloxy)-9*H*-carbazole (0.500 g, 1.9 mmol, 1.0 equiv.) in dry DMF (7 mL) cooled to 0 °C was added sodium hydride (60% dispersion on mineral oil, 0.154 g, 3.8 mmol, 2.0 equiv.) in portions. The reaction mixture was stirred under argon for 1 h after which benzyl bromide (0.657 g, 3.8 mmol, 2.0 equiv.) was added. The mixture was stirred overnight at rt until complete consumption of the starting material (TLC analysis). The reaction mixture was poured into $H_2O$ (20 mL) and extracted with EtOAc (3 × 20 mL). The organic phase was then washed with $H_2O$ (3 × 50 mL) and brine (50 mL), then dried ($Na_2SO_4$) and filtered. After removal of the solvent under reduced pressure, the residue was purified by silica-gel column chromatography (pent/EtOAc = 20:1, then 10:1) to afford the desired product as a brown solid (0.352 g, 52%). $^1H$ NMR (400 MHz, $CDCl_3$) $\delta$ 8.26 (ddd, $J$ = 5.0, 2.0, 0.8 Hz, 1H), 7.99 (ddd, $J$ = 7.9, 0.8, 0.3 Hz, 1H), 7.73 (ddd, $J$ = 8.3, 7.2, 2.0 Hz, 1H), 7.53 – 7.32 (m, 3H), 7.33 – 7.22 (m, 4H), 7.22 – 7.09 (m, 3H), 7.07 – 6.91 (m, 3H), 5.53 (s, 2H). $^{13}C$ NMR (101 MHz, $CDCl_3$): $\delta$ 163.5, 149.2, 147.7, 142.7, 140.5, 140.0, 136.9, 128.8, 127.5, 126.5, 126.4, 125.8, 123.0, 121.1, 119.6, 118.5, 115.7, 111.6, 111.0, 108.6, 105.8, 46.9. HRMS-ESI: calcd for $C_{24}H_{18}N_2NaO$ $[M+Na]^+$, 373.1311; found, 373.1304.

*N*-protection of Sulfaphenazole

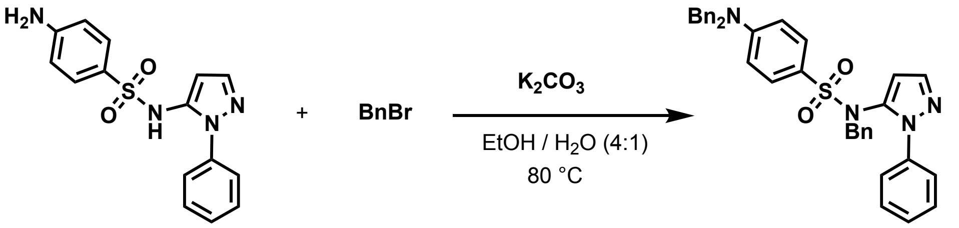


In a round bottom flask, EtOH (3.2 mL) and distilled $H_2O$ (0.8 mL) were added to a mixture of Sulfaphenazole (0.314 g, 1.0 mmol, 1.0 equiv.), benzyl bromide (0.513 g, 3.0 mmol, 3.0 equiv.) and $K_2CO_3$ (0.415 g, 3.0 mmol, 3.0 equiv.). The reaction mixture was stirred at 80 °C overnight until the starting material was fully consumed (TLC analysis). Add water (10 mL) to the mixture, extracted with EtOAc (3 × 10 mL), dried ($Na_2SO_4$), filtered and concentrated under reduced pressure. The residue was purified by silica-gel column chromatography (pentane/EtOAc = 4:1), affording the product as a light yellow solid (0.292 g, 50%). $^1H$ NMR (400 MHz, $CDCl_3$) $\delta$ 7.55 (s, 1H), 7.53 (s, 2H), 7.41 – 7.29 (m, 8H), 7.24 (d, $J$ = 8.0 Hz, 5H), 7.15 (d, $J$ = 6.1 Hz, 3H), 7.04 (dd, $J$ = 7.6, 7.5 Hz, 2H), 6.84 (d, $J$ = 7.5 Hz, 2H), 6.76 (d, $J$ = 7.1 Hz, 2H), 6.00 (s, 1H), 4.76 (s, 4H), 4.38 (s, 2H). $^{13}C$ NMR (101 MHz, $CDCl_3$): 152.7, 139.4, 138.6, 138.3, 136.8, 134.0, 130.5, 129.4, 129.0, 128.4, 128.2, 128.0, 127.6, 127.5, 126.4, 125.2, 123.7, 111.5, 104.2, 56.7, 54.2. HRMS-ESI: calcd for $C_{36}H_{32}N_4NaO_2S$ $[M+Na]^+$, 607.2138; found, 607.2147.

# 3. C–H Methylation Reactions

**Solution-based C–H methylation protocols:**

Methylation of 2-phenylpyridine:

To a 15 mL Schlenk tube, were successively added 2-phenylpyridine (0.047 g, 0.3 mmol, 1.0 equiv.), $MeB(OH)_2$ (0.073 g, 0.6 mmol, 2.0 equiv.), $[Cp^*RhCl_2]_2$ (0.009 g, 0.015 mmol, 5 mol%) and $Ag_2CO_3$ (0.124 g, 0.45 mmol, 1.5 equiv.). The tube was evacuated and backfilled with Ar three times and DCE (4.5 mL) added. The reaction was stirred at 100 °C for 2 h and cooled to rt. The crude mixture was filtered through a thin layer of Celite (3 cm), eluting with EtOAc (50 mL). 1,3,5-Trimethoxybenzene was added as a stock solution (0.1 M in EtOAc) for use as an NMR standard and the filtrate was concentrated under reduced pressure. The products were obtained in 80% spectroscopic yield (mono/di-methylated product ratio = 85:15).

Methylation of 2-phenoxypyridine:

To a 15 mL Schlenk tube, were successively added phenoxypyridine (0.051 g, 0.3 mmol, 1.0 equiv.), $MeBF_3K$ (0.220 g, 1.8 mmol, 6.0 equiv.), $[Cp^*RhCl_2]_2$ (0.009 g, 0.015 mmol, 5 mol%), $AgSbF_6$ (0.021g, 0.06 mmol, 20 mol%) and $Ag_2CO_3$ (0.207 g, 0.75 mmol, 2.5 equiv.). The tube was evacuated and backfilled with Ar three times before adding DCE (4.5 mL). The reaction was stirred at 100 °C for 2 h and cooled to rt. The crude material inside filtered through a thin layer of Celite (3 cm), eluting with EtOAc (50 mL). 1,3,5-Trimethoxybenzene was added as a stock solution (0.1 M in EtOAc) for use as an NMR standard and the filtrate was concentrated under reduced pressure. The products were obtained in 87% spectroscopic yield (mono/di-methylated product ratio = 53:47).

**Mechanochemical C–H methylation protocols:**

**General procedure 1:** A stainless steel milling vessel (14 mL internal volume) was charged with substrate (0.3 mmol), $MeB(OH)_2$ (1.2-4.0 equiv.), $[Cp^*RhCl_2]_2$ (0.009 g, 0.015 mmol, 5 mol%), $Ag_2CO_3$ (0.124 g, 0.045 mmol, 1.5 equiv.) and one stainless steel ball (10 mm diameter). The vessel was mounted into the holding station of a mixer mill and milling was conducted at the indicated frequency for the indicated time. The vessel was then cooled to rt and the crude material was washed out with EtOAc or MeOH, filtered through a thin layer of Celite (3 cm), eluting with EtOAc (200 mL) or an EtOAc/MeOH mixture (1:1, 200 mL). 1,3,5-Trimethoxybenzene or naphthalene was added as a stock solution (0.1 M in EtOAc) for use as an NMR standard and the filtrate was concentrated under reduced pressure. The residue was further purified by silica-gel column chromatography to give the product.

**General procedure 2:** A Teflon™ milling vessel (14 mL internal volume) was charged with substrate (0.3 mmol), $MeBF_3K$ (1.5-6.0 equiv.), $[Cp^*RhCl_2]_2$ (5 or 10 mol%), $AgSbF_6$ (20 or 40 mol%), $Ag_2CO_3$ (1.5-3.0 equiv.) and a stainless steel ball (15 mm diameter). The vessel was mounted into the holding station of a mixer mill and milling was started at the indicated frequency (25 or 36 Hz) and continued for the indicated time. The crude reaction mixture was washed out with EtOAc or MeOH, then filtered through a thin layer of Celite (3 cm), eluting with EtOAc (200 mL) or an EtOAc/MeOH mixture (1:1, 200 mL). For indicated cases, 1,3,5-trimethoxybenzene was added as a stock solution (0.1 M in EtOAc) for use as an NMR

standard, the filtrate was concentrated under reduced pressure and analysed by $^1$H NMR. The residue was further purified by silica-gel column chromatography to give the product.

**A note about milling reaction temperature measurements:**
The temperature of the interior wall of the milling vessels as well as that of the stainless steel balls were measured using a laser thermometer immediately after the cessation of several reactions. Representative values for Teflon™ and stainless steel balls are:

14 mL Teflon™ vessel (15 mm SS ball):
At 25 Hz after 1 h: 45 °C (inside wall and ball).
At 36 Hz after 1 h: 73 °C (inside wall and ball).

14 mL SS vessel (10 mm SS ball):
At 36 Hz after 1 h: 46 °C (inside wall and ball).

## A. Methylation products via 5-membered rhodacycles

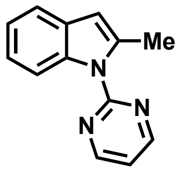


**2-Methyl-1-(pyrimidin-2-yl)-1*H*-indole (5a)**[22]
According to general procedure 1, the reaction was stopped after 2 h. The mono/di-methylated product ratio was 44:1 according to crude $^1$H NMR spectroscopy and after purification, the title compound was obtained as a colorless solid (0.058 g, 92%). $^1$H NMR (400 MHz, $CDCl_3$): $\delta$ 8.79 (d, $J$ = 4.8 Hz, 2H), 8.29 (d, $J$ = 8.0 Hz, 1H), 7.51 (d, $J$ = 7.9 Hz, 1H), 7.24 – 7.17 (m, 2H), 7.14 (t, $J$ = 4.8 Hz, 1H), 6.43 (s, 1H), 2.72 (s, 3H).

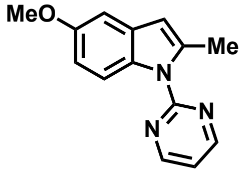


**5-Methoxy-2-methyl-1-(pyrimidin-2-yl)-1*H*-indole (5b)**[23]
According to general procedure 1, the reaction was stopped after 1 h. The mono/di-methylated product ratio is 49:1 according to $^1$H NMR spectroscopy and after purification, the title compound was obtained as a light yellow solid (0.060 g, 84%). $^1$H NMR (400 MHz, $CDCl_3$): $\delta$ 8.75 (d, $J$ = 4.8 Hz, 2H), 8.25 (d, $J$ = 9.0 Hz, 1H), 7.10 (t, $J$ = 4.8 Hz, 1H), 6.98 (d, $J$ = 2.6 Hz, 1H), 6.84 (dd, $J$ = 9.1, 2.6 Hz, 1H), 6.36 (s, 1H), 3.86 (s, 3H), 2.72 (s, 3H).

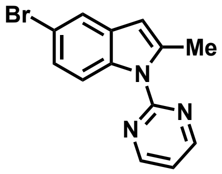

**5-Bromo-2-methyl-1-(pyrimidin-2-yl)-1*H*-indole (5c)**[24]

According to general procedure 1, the reaction was stopped after 1 h. The mono/di-methylated product ratio is 49/1 according to the crude $^{1}$H NMR and after purification, the title compound was obtained as a colorless solid (0.063 g, 72%). $^{1}$H NMR (400 MHz, $CDCl_3$): $\delta$ 8.78 (d, $J$ = 4.8 Hz, 2H), 8.18 (d, $J$ = 8.8 Hz, 1H), 7.62 (d, $J$ = 2.0 Hz, 1H), 7.28 (dd, $J$ = 8.9, 2.1 Hz, 1H), 7.16 (t, $J$ = 4.8 Hz, 1H), 6.37 (q, $J$ = 1.0 Hz, 1H), 2.71 (d, $J$ = 1.1 Hz, 3H).

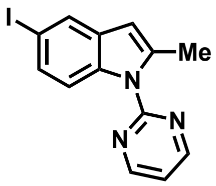

**5-Iodo-2-methyl-1-(pyrimidin-2-yl)-1*H*-indole (5d)**

According to general procedure 1, the reaction was stopped after 4 h. The mono/di-methylated product ratio is 28/1 according to the crude $^{1}$H NMR and after purification, the title compound was obtained as a colorless solid (0.073 g, 72%). $^{1}$H NMR (400 MHz, $CDCl_3$): $\delta$ 8.78 (d, $J$ = 4.8 Hz, 2H), 8.08 (d, $J$ = 8.8 Hz, 1H), 7.83 (d, $J$ = 1.7 Hz, 1H), 7.46 (dd, $J$ = 8.8, 1.8 Hz, 1H), 7.15 (t, $J$ = 4.8 Hz, 1H), 6.35 (s, 1H), 2.71 (s, 3H). $^{13}$C NMR (101 MHz, $CDCl_3$): $\delta$ 158.10, 158.08, 138.8, 136.0, 131.9, 130.6, 128.2, 117.2, 116.1, 105.6, 85.6, 16.6. HRMS-ESI: calcd for $C_{13}H_{11}N_3I$ $[M+H]^+$, 335.9992; found, 335.9991.

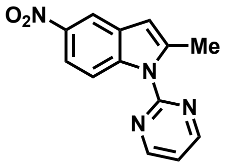

**2-Methyl-5-nitro-1-(pyrimidin-2-yl)-1*H*-indole (5e)**

According to general procedure 1, the reaction was stopped after 3 h. The mono/di-methylated product ratio is 24/1 according to the crude $^{1}$H NMR and after purification, the title compound was obtained as a yellow solid (0.058 g, 74%). $^{1}$H NMR (400 MHz, $CDCl_3$): $\delta$ 8.85 (d, $J$ = 4.8 Hz, 2H), 8.43 (d, $J$ = 2.4 Hz,1H), 8.30 (d, $J$ = 9.2 Hz, 1H), 8.10 (dd, $J$ = 9.2, 2.4 Hz, 1H), 7.27 (t, $J$ = 4.8 Hz, 1H), 6.57 (s, 1H), 2.74 (s, 3H). $^{13}$C NMR (101 MHz, $CDCl_3$): $\delta$ 158.4, 157.7, 143.0, 141.3, 139.8, 128.9, 118.2, 117.7, 115.9, 113.9, 107.2, 16.5. HRMS-ESI: calcd for $C_{13}H_{11}N_4O_2[M+H]^+$, 255.0877; found, 255.0879.

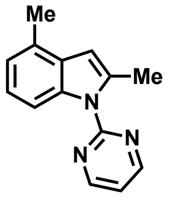

**2,4-Dimethyl-1-(pyrimidin-2-yl)-1*H*-indole (5f)**[24]

According to general procedure 1, the reaction was stopped after 1 h. No C7 methylation was detected and after purification, the title compound was obtained as a colorless solid (0.061 g, 95%). $^{1}$H NMR (400 MHz, $CDCl_3$): $\delta$ 8.78 (d, $J$ = 4.8 Hz, 2H), 8.11 (d, $J$ = 8.4 Hz, 1H), 7.16 – 7.08 (m, 2H), 6.98 (d, $J$ = 7.2 Hz, 1H), 6.46 (s, 1H), 2.72 (s, 3H), 2.53 (s, 3H).

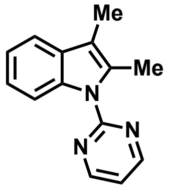

**2,3-Dimethyl-1-(pyrimidin-2-yl)-1*H*-indole (5g)**[23]

According to general procedure 1, the reaction was stopped after 1 h. No C7 methylation was detected and after purification, the title compound was obtained as a colorless solid (0.066 g, 99%). $^{1}$H NMR (400 MHz, $CDCl_3$): $\delta$ 8.77 (d, $J$ = 4.8 Hz, 2H), 8.31 – 8.20 (m, 1H), 7.53 – 7.45 (m, 1H), 7.24 – 7.17 (m, 2H), 7.11 (t, $J$ = 4.8 Hz, 1H), 2.63 (s, 3H), 2.29 (s, 3H).

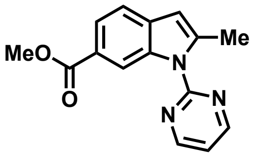

**Methyl 2-methyl-1-(pyrimidin-2-yl)-1*H*-indole-6-carboxylate (5h)**

According to general procedure 1, the reaction was stopped after 3 h. According to general procedure 1, the reaction was stopped after 3 h and after purification, the title compound was obtained as a colorless solid (0.075 g, 94%). $^{1}$H NMR (500 MHz, $CDCl_3$): $\delta$ 8.94 (d, $J$ = 1.6 Hz, 1H), 8.84 (d, $J$ = 4.8 Hz, 2H), 7.88 (dd, $J$ = 8.3, 1.4 Hz, 1H), 7.53 (d, $J$ = 8.2 Hz, 1H), 7.21 (t, $J$ = 4.8 Hz, 1H), 6.48 (s, 1H), 3.93 (s, 3H), 2.74 (s, 3H). $^{13}$C NMR (125 MHz, $CDCl_3$): $\delta$ 168.2, 158.3, 158.0, 141.3, 136.2, 133.2, 124.0, 123.1, 119.0, 117.5, 115.9, 106.6, 51.9, 16.7. HRMS-ESI: calcd for $C_{15}H_{14}N_3O_2$ $[M+H]^+$, 268.1081; found, 268.1077.

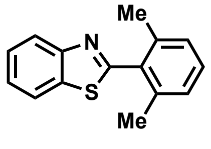

**2-(2,6-Dimethylphenyl)benzo[*d*]thiazole (5i)**[25]

According to general procedure 2, $MeBF_3K$ (0.220 g, 1.8 mmol, 6.0 equiv.), $[Cp^*RhCl_2]_2$ (0.019 g, 10 mol%), $AgSbF_6$ (0.042 g, 40 mol%), $Ag_2CO_3$ (0.207 g, 0.75 mmol, 2.5 equiv.) were added. Milling was conducted at 36 Hz and stopped after 1 h. The mono/di-methylated product ratio is 1:10 according to the crude $^{1}$H NMR and after purification, the title compound was obtained as a colorless solid (0.051 g, 71%). $^{1}$H NMR (400 MHz, $CDCl_3$): $\delta$ 8.12 (ddd, $J$

= 8.1, 1.0, 0.8 Hz, 1H), 7.95 (ddd, $J$ = 8.1, 1.1, 1.0 Hz, 1H), 7.54 (ddd, $J$ = 8.3, 7.2, 1.2 Hz, 1H), 7.45 (ddd, $J$ = 8.2, 7.2, 1.1 Hz, 1H), 7.28 (t, $J$ = 7.7 Hz, 1H), 7.14 (d, $J$ = 7.6 Hz, 2H), 2.21 (s, 6H).

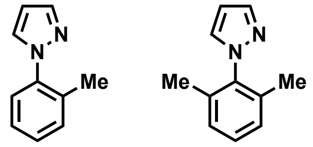


**1-(*o*-Tolyl)-1*H*-pyrazole (5j)[26] and 1-(2,6-dimethylphenyl)-1*H*-pyrazole (5j')[27]**

According to general procedure 2, $MeBF_3K$ (0.220 g, 1.8 mmol, 6.0 equiv.), $[Cp^*RhCl_2]_2$ (0.009 g, 5 mol%), $AgSbF_6$ (0.021 g, 20 mol%), $Ag_2CO_3$ (0.207 g, 0.75 mmol, 2.5 equiv.) were added. Milling was started at 36 Hz and stopped after 4 h. The mono/di-methylated product ratio is 1/1.7 according to the crude $^1$H NMR and after purification, **5j** and **5j'** were obtained as a colorless solid (0.051 g, 71%). **5j**: $^1$H NMR (400 MHz, $CDCl_3$): $\delta$ 7.72 (dd, $J$ = 1.9, 0.7 Hz, 1H), 7.60 (dd, $J$ = 2.3, 0.7 Hz, 1H), 7.39 – 7.27 (m, 4H), 6.45 – 6.43 (m, 1H), 2.25 (s, 2H). **5j'**: $^1$H NMR (400 MHz, $CDCl_3$): $\delta$ 7.74 (dd, $J$ = 1.9, 0.7 Hz, 1H), 7.46 (dd, $J$ = 2.3, 0.7 Hz, 1H), 7.23 (d, $J$ = 7.0 Hz, 1H), 7.16 – 7.09 (m, 2H), 6.48 – 6.42 (m, 1H), 2.01 (s, 6H).

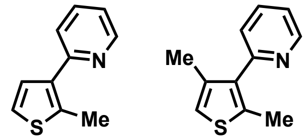


**2-Methyl-3-phenylthiophene (5k) and 2,4-dimethyl-3-phenylthiophene (5k')**

According to general procedure 1, $MeB(OH)_2$ (0.022 g, 0.36 mmol, 1.2 equiv.) was added and the reaction was stopped after 2 h. The mono/di-methylated product ratio is 4:1 according to the crude $^1$H NMR and after purification, **5k** was obtained as a light yellow oil (0.035 g, 67%). $^1$H NMR (500 MHz, $CDCl_3$): $\delta$ 8.67 (ddd, $J$ = 5.0, 1.8, 1.0 Hz, 1H), 7.72 (ddd, $J$ = 7.7, 7.6, 1.9 Hz, 1H), 7.46 (ddd, $J$ = 8.0, 7.9, 1.2 Hz, 1H), 7.31 (d, $J$ = 5.3 Hz, 1H), 7.18 (ddd, $J$ = 7.5, 4.8, 1.2 Hz, 1H), 7.11 (d, $J$ = 5.3 Hz, 1H), 2.68 (s, 3H). $^{13}$C NMR (101 MHz, $CDCl_3$) $\delta$ 155.2, 149.4, 137.7, 137.4, 136.2, 128.7, 122.6, 121.5, 121.1, 14.8. HRMS-ESI: calcd for $C_{10}H_{10}NS$ $[M+H]^+$, 176.0528; found, 176.0525; **5k'** was obtained as a light yellow oil (0.009 g, 16%). $^1$H NMR (500 MHz, $CDCl_3$): $\delta$ 8.71 (d, $J$ = 4.9 Hz, 1H), 7.74 (dd, $J$ = 7.8, 7.7 Hz, 1H), 7.28 (d, $J$ = 7.9 Hz, 1H), 7.22 (dd, $J$ = 7.6, 5.0 Hz, 1H), 6.76 (s, 1H), 2.40 (s, 3H), 2.15 (s, 3H). $^{13}$C NMR (125 MHz, $CDCl_3$) $\delta$ 155.8, 149.6, 138.5, 137.6, 137.1, 136.0, 124.8, 121.5, 117.6, 15.8, 14.3. HRMS-ESI: calcd for $C_{11}H_{12}NS$ $[M+H]^+$, 190.0685; found, 190.0680.

## B. Methylation products via 6-membered rhodacycles

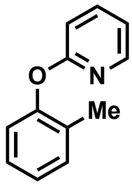


**2-(*o*-Tolyloxy)pyridine (8a)**[28]

According to general procedure 2, $MeBF_3K$ (0.073 g, 0.6 mmol, 2.0 equiv.), $[Cp^*RhCl_2]_2$ (0.009 g, 5 mol%), $AgSbF_6$ (0.021 g, 20 mol%), $Ag_2CO_3$ (0.124 g, 0.45 mmol, 1.5 equiv.) were added. Milling was started at 36 Hz and stopped after 1 h. Using a Teflon™ vessel (as per general procedure 2), a mixture of **8a** and **8b** was obtained in 78% yield (**8a**/**8b** = 56:44). Using a stainless steel vessel, **8a** was obtained in 11% yield (**8a**/**8b** = >99:1).

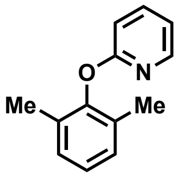


**2-(2,6-Dimethylphenoxy)pyridine (8b)**[28]

According to general procedure 2, $MeBF_3K$ (0.220 g, 1.8 mmol, 6.0 equiv.), $[Cp^*RhCl_2]_2$ (0.009 g, 5 mol%), $AgSbF_6$ (0.021 g, 20 mol%), $Ag_2CO_3$ (0.207 g, 0.75 mmol, 2.5 equiv.) were added. Milling was started at 36 Hz and stopped after 2 h. No monomethylated product was detected and after purification, the title compound was obtained as a colorless solid (0.051 g, 85%). $^1$H NMR (400 MHz, $CDCl_3$): $\delta$ 8.15 (ddd, $J$ = 5.0, 2.0, 0.8 Hz, 1H), 7.65 (ddd, $J$ = 8.3, 7.1, 2.0 Hz, 1H), 7.15 – 7.02 (m, 3H), 6.93 (ddd, $J$ = 7.2, 5.0, 0.9 Hz, 1H), 6.81 (ddd, $J$ = 8.3, 0.9, 0.8 Hz, 1H), 2.13 (s, 6H).

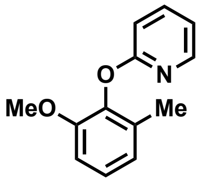


**2-(2-Methoxy-6-methylphenoxy)pyridine (8c)**[29]

According to general procedure 2, $MeBF_3K$ (0.146 g, 1.2 mmol, 4.0 equiv.), $[Cp^*RhCl_2]_2$ (0.009 g, 5 mol%), $AgSbF_6$ (0.021 g, 20 mol%), $Ag_2CO_3$ (0.207 g, 0.75 mmol, 2.5 equiv.) were added and the reaction was stopped after 1 h. After purification, the title compound was obtained as colorless solid (0.046 g, 71%).

$^1$H NMR (400 MHz, $CDCl_3$): $\delta$ 8.14 (ddd, $J$ = 5.0, 2.1, 0.8 Hz, 1H), 7.64 (ddd, $J$ = 8.4, 7.1, 2.0 Hz, 1H), 7.11 (dd, $J$ = 8.0, 7.9 Hz, 1H), 6.92 (ddd, $J$ = 7.2, 5.0, 0.9 Hz, 1H), 6.90 – 6.82 (m, 3H), 3.73 (s, 3H), 2.17 (s, 3H).

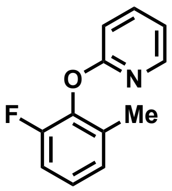

**2-(2-Fluoro-6-methylphenoxy)pyridine (8d)**

According to general procedure 2, $MeBF_3K$ (0.110 g, 0.9 mmol, 3.0 equiv.), $[Cp^*RhCl_2]_2$ (0.009 g, 5 mol%), $AgSbF_6$ (0.021 g, 20 mol%), $Ag_2CO_3$ (0.124 g, 0.45 mmol, 1.5 equiv.) were added and the reaction was stopped after 1 h. The title compound was obtained as a colorless solid (0.052 g, 85%). $^1H$ NMR (400 MHz, $CDCl_3$): $\delta$ 8.13 (ddd, $J$ = 4.9, 2.0, 0.9 Hz, 1H), 7.69 (ddd, $J$ = 8.3, 7.2, 2.0 Hz, 1H), 7.13-6.96 (m, 5H), 2.12 (s, 3H). $^{19}F$ NMR (376 MHz, $CDCl_3$) $\delta$ -129.16 – -129.21 (m). $^{13}C$ NMR (101 MHz, $CDCl_3$): $\delta$ 162.7, 155.2 (d, $J$ = 247.6 Hz), 147.6, 139.39, 139.38 (d, $J$ = 12.5 Hz), 133.8 (d, $J$ = 1.0 Hz), 126.0 (d, $J$ = 3.1 Hz), 125.6 (d, $J$ = 7.7 Hz), 118.3, 114.0 (d, $J$ = 18.7 Hz), 110.2, 16.1 (d, $J$ = 2.6 Hz). HRMS-EI: calcd for $C_{12}H_9NFO$ $[M-H]^+$, 202.0663; found, 202.0663.

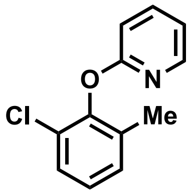

**2-(2-Chloro-6-methylphenoxy)pyridine (8e)**[30]

According to general procedure 2, $MeBF_3K$ (0.110 g, 0.9 mmol, 3.0 equiv.), $[Cp^*RhCl_2]_2$ (0.009 g, 5 mol%), $AgSbF_6$ (0.021 g, 20 mol%), $Ag_2CO_3$ (0.124 g, 0.45 mmol, 1.5 equiv.) were added and the reaction was stopped after 1 h. After purification, the title compound was obtained as a colorless solid (0.057 g, 86%). $^1H$ NMR (400 MHz, $CDCl_3$): $\delta$ 8.12 (ddd, $J$ = 5.0, 2.0, 0.9 Hz, 1H), 7.70 (ddd, $J$ = 8.2, 7.2, 2.0 Hz, 1H), 7.31 (dd, $J$ = 7.9, 1.7 Hz, 1H), 7.18 (ddd, $J$ = 7.6, 1.8, 0.9 Hz, 1H), 7.10 (dd, $J$ = 7.8, 7.7 Hz, 1H), 7.02 – 6.90 (m, 2H), 2.19 (s, 3H).

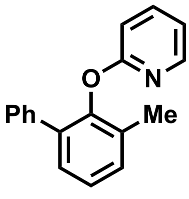

**2-((3-Methyl-[1,1'-biphenyl]-2-yl)oxy)pyridine (8f)**[31]

According to general procedure 2, $MeBF_3K$ (0.110 g, 0.9 mmol, 3.0 equiv.), $[Cp^*RhCl_2]_2$ (0.009 g, 5 mol%), $AgSbF_6$ (0.021 g, 20 mol%), $Ag_2CO_3$ (0.124 g, 0.45 mmol, 1.5 equiv.) were added and the reaction was stopped after 1 h. After purification, the title compound was obtained as colorless solid (0.064 g, 82%). $^1H$ NMR (400 MHz, $CDCl_3$): $\delta$ 8.05 (ddd, $J$ = 5.0, 2.0, 0.8 Hz, 1H), 7.50 (ddd, $J$ = 8.4, 7.2, 2.0 Hz, 1H), 7.44 – 7.35 (m, 2H), 7.33 – 7.12 (m, 6H), 6.81 (ddd, $J$ = 7.1, 5.0, 0.9 Hz, 1H), 6.65 (d, $J$ = 8.3 Hz, 1H), 2.18 (s, 3H).

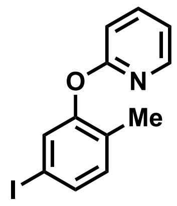

**2-(5-Iodo-2-methylphenoxy)pyridine (8h)**

According to general procedure 2, $MeBF_3K$ (0.110 g, 0.9 mmol, 3.0 equiv.), $[Cp^*RhCl_2]_2$ (0.009 g, 5 mol%), $AgSbF_6$ (0.021 g, 20 mol%), $Ag_2CO_3$ (0.124 g, 0.45 mmol, 1.5 equiv.) were added. Milling was started at 36 Hz and stopped after 0.5 h. The mono/di-methylated product ratio is 19/1 according to the crude $^1H$ NMR and after purification, the title compound was obtained as a colorless solid (0.070 g, 75%). $^1H$ NMR (400 MHz, $CDCl_3$): $\delta$ 8.17 (d, $J$ = 3.9 Hz, 1H), 7.68 (dd, $J$ = 8.8, 7.4 Hz, 1H), 7.45 (d, $J$ = 8.0 Hz, 1H), 7.39 (s, 1H), 7.01 – 6.97 (m, 2H), 6.89 (d, $J$ = 8.3 Hz, 1H), 2.13 (s, 3H). $^{13}C$ NMR (101 MHz, $CDCl_3$): $\delta$ 163.2, 152.8, 147.8, 139.5, 134.1, 132.8, 130.69, 130.67, 118.4, 110.9, 90.1, 16.1. HRMS-EI: calcd for $C_{12}H_{10}INO$ $[M]^{+\cdot}$, 310.9802; found, 310.9799.

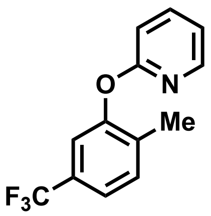

**2-(2-Methyl-5-(trifluoromethyl)phenoxy)pyridine (8i)**

According to general procedure 2, $MeBF_3K$ (0.110 g, 0.9 mmol, 3.0 equiv.), $[Cp^*RhCl_2]_2$ (0.009 g, 5 mol%), $AgSbF_6$ (0.021 g, 20 mol%), $Ag_2CO_3$ (0.124 g, 0.45 mmol, 1.5 equiv.) were added. Milling was started at 36 Hz and stopped after 1 h. No dimethylated product was detected and the title compound was obtained as a colorless solid (0.059 g, 78%). $^1H$ NMR (400 MHz, $CDCl_3$): $\delta$ 8.16 (dd, $J$ = 5.2, 2.0 Hz, 1H), 7.71 (ddd, $J$ = 8.3,7.2, 2.0 Hz, 1H), 7.41 – 7.36 (m, 2H), 7.32 (s, 1H), 7.01 (ddd, $J$ = 7.2, 5.0, 0.9 Hz, 1H), 6.94 (d, $J$ = 8.3 Hz, 1H), 2.24 (s, 3H). $^{19}F$ NMR (376 MHz, $CDCl_3$) $\delta$ -62.30 (s). $^{13}C$ NMR (101 MHz, $CDCl_3$): $\delta$ 163.1, 152.3, 147.8, 139.6, 135.1 (q, $J$ = 0.9 Hz), 131.7, 129.5 (q, $J$ = 32.9 Hz), 123.8 (q, $J$ = 272.0 Hz), 121.7 (q, $J$ = 3.8 Hz), 118.9 (q, $J$ = 3.9 Hz), 118.6, 111.1, 16.5. HRMS-EI: calcd for $C_{13}H_9F_3NO$ $[M-H]^+$, 252.0631; found, 252.0631.

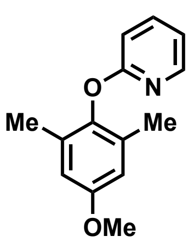

**5-Methoxy-1,3-dimethyl-2-phenoxybenzene (8j)**

According to general procedure 2, $MeBF_3K$ (0.220 g, 1.8 mmol, 6.0 equiv.), $[Cp^*RhCl_2]_2$ (0.009 g, 5 mol%), $AgSbF_6$ (0.021 g, 20 mol%), $Ag_2CO_3$ (0.207 g, 0.75 mmol, 2.5 equiv.) were added. Milling was started at 36 Hz and stopped after 2 h. No monomethylated product was detected and after purification, the title compound was obtained as a colorless solid (0.056 g, 81%). $^1H$ NMR (400 MHz, $CDCl_3$): $\delta$ 8.16 (ddd, $J$ = 5.0, 2.0, 0.8 Hz, 1H), 7.64 (ddd, $J$ = 8.3, 7.1, 2.0 Hz, 1H), 6.92 (ddd, $J$ = 7.2, 5.0 Hz, 1.0 Hz, 1H), 6.80 (ddd, $J$ = 8.3, 1.0, 0.8 Hz, 1H),

6.64 (s, 2H), 3.79 (s, 3H), 2.10 (s, 6H). $^{13}$C NMR (101 MHz, $CDCl_3$): $\delta$ 163.5, 156.4, 148.0, 144.0, 139.3, 131.9, 117.5, 113.7, 109.6, 55.3, 16.8. HRMS-EI: calcd for $C_{14}H_{15}NO_2$ $[M]^{+\cdot}$, 229.1097; found, 229.1097.

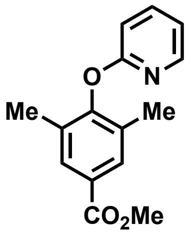


**Methyl 3,5-dimethyl-4-phenoxybenzoate (8k)**

According to general procedure 2, $MeBF_3K$ (0.220 g, 1.8 mmol, 6.0 equiv.), $[Cp*RhCl_2]_2$ (0.009 g, 5 mol%), $AgSbF_6$ (0.021 g, 20 mol%), $Ag_2CO_3$ (0.207 g, 0.75 mmol, 2.5 equiv.) were added. Milling was started at 36 Hz and after 2 h. No monomethylated product was detected and after purification, the title compound was obtained as a colorless solid (0.068 g, 88%). $^{1}$H NMR (400 MHz, $CDCl_3$): $\delta$ 8.11 (ddd, $J$ = 5.0, 2.0, 0.8 Hz, 1H), 7.81 (s, 2H), 7.69 (ddd, $J$ = 8.4, 7.2, 2.0 Hz, 1H), 6.95 (ddd, $J$ = 7.2, 5.0, 0.9 Hz, 1H), 6.89 (ddd, $J$ = 8.3, 0.9, 0.8 Hz, 1H), 3.90 (s, 3H), 2.15 (s, 6H). $^{13}$C NMR (101 MHz, $CDCl_3$): $\delta$ 166.9, 162.7, 154.4, 147.9, 139.5, 131.5, 130.3, 126.9, 118.0, 110.0, 52.0, 16.6. HRMS-EI: calcd for $C_{15}H_{15}NO_3$ $[M]^{+\cdot}$, 257.1046; found, 257.1046.

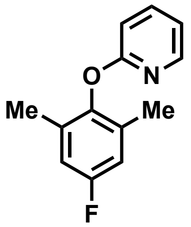


**2-(3-Fluoro-2,6-dimethylphenoxy)pyridine (8l)**

According to general procedure 2, $MeBF_3K$ (0.220 g, 1.8 mmol, 6.0 equiv.), $[Cp*RhCl_2]_2$ (0.009 g, 5 mol%), $AgSbF_6$ (0.021 g, 20 mol%), $Ag_2CO_3$ (0.207 g, 0.75 mmol, 2.5 equiv.) were added. Milling was started at 36 Hz and stopped after 2 h. No monomethylated product was detected and after purification, the title compound was obtained as a light yellow solid (0.051 g, 78%). $^{1}$H NMR (400 MHz, $CDCl_3$): $\delta$ 8.14 (ddd, $J$ = 5.0, 2.2, 0.9 Hz, 1H), 7.67 (ddd, $J$ = 8.8, 7.1, 2.0 Hz, 1H), 6.94 (ddd, $J$ = 7.2, 5.0, 1.1 Hz, 1H), 6.86 (ddd, $J$ = 8.3, 1.0, 0.9 Hz, 1H), 6.81 (d, $J$ = 8.9 Hz, 2H), 2.10 (s, 6H). $^{19}$F NMR (376 MHz, $CDCl_3$) $\delta$ -118.69 – -118.75 (m). $^{13}$C NMR (101 MHz, $CDCl_3$): $\delta$ 163.0 (d, $J$ = 1.1 Hz), 159.5 (d, $J$ = 242.6 Hz), 147.9, 146.1 (d, $J$ = 2.7 Hz), 139.4, 132.9 (d, $J$ = 8.7 Hz), 117.8, 115.0 (d, $J$ = 22.6 Hz), 109.8, 16.7 (d, $J$ = 1.5 Hz). HRMS-EI: calcd for $C_{13}H_{12}FNO$ $[M]^{+\cdot}$, 217.0897; found, 217.0897.

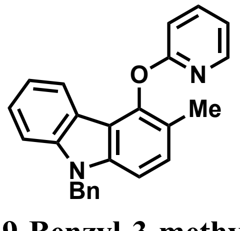


**9-Benzyl-3-methyl-4-(pyridin-2-yloxy)-9*H*-carbazole (8g)**

According to general procedure 2, $MeBF_3K$ (0.110 g, 0.9 mmol, 3.0 equiv.), $[Cp*RhCl_2]_2$ (0.009 g, 5 mol%), $AgSbF_6$ (0.021 g, 20 mol%), $Ag_2CO_3$ (0.124 g, 0.45 mmol, 1.5 equiv.) were added. Milling was started at 36 Hz and stopped after 2 h. After purification, the title compound

was obtained as a light yellow solid (0.077 g, 70%). $^{1}$H NMR (400 MHz, $CDCl_3$): $\delta$ 8.21 (ddd, $J$ = 5.1, 2.1, 1.0 Hz, 1H), 7.90 (d, $J$ = 7.8 Hz, 1H), 7.67 (ddd, $J$ = 7.8, 7.3, 2.0 Hz, 1H), 7.38 – 7.17 (m, 9H), 7.08 (ddd, $J$ = 7.9, 6.6, 1.2 Hz, 1H), 6.97 – 6.92 (m, 2H), 5.50 (s, 2H), 2.33 (s, 3H). $^{13}$C NMR (101 MHz, $CDCl_3$): $\delta$ 163.3, 148.3, 146.3, 141.1, 140.7, 139.5, 137.1, 128.74, 128.69, 127.4, 126.4, 125.6, 122.8, 121.3, 121.0, 119.3, 117.8, 116.5, 109.5, 108.5, 106.1, 46.8, 15.7. HRMS-ESI: calcd for $C_{25}H_{21}N_2O$ $[M+H]^+$, 365.1648; found, 365.1652.

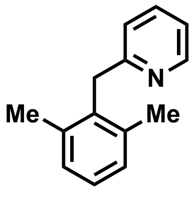


**2-(2,6-Dimethylbenzyl)pyridine (8m)**[32]

According to general procedure 2, $MeBF_3K$ (0.146 g, 1.2 mmol, 4.0 equiv.), $[Cp^*RhCl_2]_2$ (0.019 g, 10 mol%), $AgSbF_6$ (0.042 g, 40 mol%), $Ag_2CO_3$ (0.207 g, 0.75 mmol, 2.5 equiv.) were added. Milling was started at 36 Hz and stopped after 1 h. No monomethylated product was detected and the title compound was obtained as a light yellow solid (0.038 g, 64%). $^{1}$H NMR (400 MHz, $CDCl_3$): $\delta$ 8.56 (ddd, $J$ = 4.9, 1.9, 1.0 Hz, 1H), 7.49 (ddd, $J$ = 7.8, 7.7, 1.9 Hz, 1H), 7.14-7.05 (m, 4H), 6.72 (ddd, $J$ = 8.0, 1.0, 0.9 Hz, 1H), 4.25 (s, 2H), 2.26 (s, 6H).

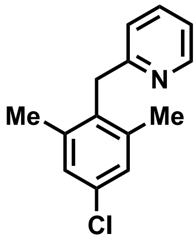


**2-(4-Chloro-2,6-dimethylbenzyl)pyridine (8n)**

According to general procedure 2, $MeBF_3K$ (0.146 g, 1.2 mmol, 4.0 equiv.), $[Cp^*RhCl_2]_2$ (0.009 g, 5 mol%), $AgSbF_6$ (0.021 g, 20 mol%), $Ag_2CO_3$ (0.207 g, 0.75 mmol, 2.5 equiv.) were added. Milling was started at 36 Hz and stopped after 0.5 h. No monomethylated product was detected and after purification, the title compound was obtained as a yellow oil (0.052 g, 75%). $^{1}$H NMR (400 MHz, $CDCl_3$): $\delta$ 8.55 (dd, $J$ = 4.9, 1.0 Hz, 1H), 7.50 (ddd, $J$ = 7.7, 7.7, 1.9 Hz, 1H), 7.10 – 7.05 (m, 3H), 6.73 (dd, $J$ = 7.9, 1.0 Hz, 1H), 4.18 (s, 2H), 2.23 (s, 6H). $^{13}$C NMR (101 MHz, $CDCl_3$): $\delta$ 159.62, 149.46, 139.13, 136.57, 134.26, 131.76, 127.93, 121.27, 121.14, 37.75, 20.22. HRMS-EI: calcd for $C_{14}H_{14}ClN$ $[M]^{+\cdot}$, 231.0809; found, 231.0811.

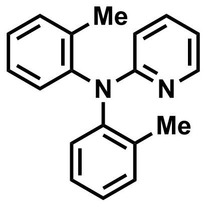


***N*, *N*-di-*o*-tolylpyridin-2-amine (8o)**

According to general procedure 2, $MeBF_3K$ (0.220 g, 1.8 mmol, 6.0 equiv.), $[Cp^*RhCl_2]_2$ (0.009 g, 5 mol%), $AgSbF_6$ (0.021 g, 20 mol%), $Ag_2CO_3$ (0.207 g, 0.75 mmol, 2.5 equiv.) were added. Milling was started at 36 Hz and stopped after 1 h. The title compound was detected as the only product and the purification leading to final product as a colorless solid (0.068 g, 83%). $^{1}$H NMR (400 MHz, $CDCl_3$): $\delta$ 8.21 (ddd, $J$ = 5.0, 2.0, 0.9 Hz, 1H), 7.41 (ddd, $J$ = 8.5, 7.2, 2.0 Hz, 1H), 7.25 – 7.23 (m, 2H), 7.18 – 7.10 (m, 4H), 7.03 (dd, $J$ = 7.5, 1.8 Hz, 2H), 6.69 (ddd, $J$

= 7.2, 5.0, 1.0 Hz, 1H), 6.43 (ddd, $J$ = 8.4, 1.0, 0.9 Hz, 1H), 2.08 (s, 6H). $^{13}$C NMR (101 MHz, $CDCl_3$): $\delta$ 158.9, 148.5, 144.4, 137.2, 135.2, 131.5, 127.9, 127.0, 125.7, 114.8, 112.0, 18.9. HRMS-EI: calcd for $C_{19}H_{17}N_2$ [M-H]$^+$, 273.1386; found, 273.1385.

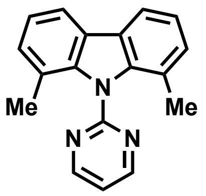


**1,8-Dimethyl-9-(pyrimidin-2-yl)-9*H*-carbazole (8p)**

According to general procedure 2, $MeBF_3K$ (0.146 g, 1.2 mmol, 4.0 equiv.), $[Cp^*RhCl_2]_2$ (0.009 g, 5 mol%), $AgSbF_6$ (0.021 g, 20 mol%), $Ag_2CO_3$ (0.207 g, 0.75 mmol, 2.5 equiv.) were added. Milling was conducted at 36 Hz and stopped after 0.5 h. No monomethylated product was detected and after purification, the title compound was obtained as a light yellow solid (0.060 g, 73%). $^1$H NMR (400 MHz, $CDCl_3$): $\delta$ 8.93 (d, $J$ = 4.8 Hz, 2H), 7.97 (d, $J$ = 7.7 Hz, 2H), 7.47 (t, $J$ = 4.9 Hz, 1H), 7.19 (dd, $J$ = 7.6, 7.3 Hz, 2H), 7.12 (d, $J$ = 7.3 Hz, 2H), 1.87 (s, 6H). $^{13}$C NMR (101 MHz, $CDCl_3$): $\delta$ 162.0, 158.4, 140.7, 128.8, 124.7, 121.3, 120.8, 120.7, 118.1, 18.8. HRMS-EI: calcd for $C_{18}H_{15}N_3$ [M]$^{+\cdot}$, 273.1260; found, 273.1256.

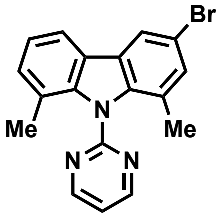


**3-Bromo-1,8-dimethyl-9-(pyrimidin-2-yl)-9*H*-carbazole (8q)**

According to general procedure 2, $MeBF_3K$ (0.146 g, 1.2 mmol, 4.0 equiv.), $[Cp^*RhCl_2]_2$ (0.009 g, 5 mol%), $AgSbF_6$ (0.021 g, 20 mol%), $Ag_2CO_3$ (0.207 g, 0.75 mmol, 2.5 equiv.) were added. Milling was conducted at 36 Hz and stopped after 0.5 h. No monomethylated product was detected and after purification, the title compound was obtained as a light yellow solid (0.065 g, 62%). $^1$H NMR (400 MHz, $CDCl_3$): $\delta$ 8.92 (d, $J$ = 4.9 Hz, 2H), 8.08 (d, $J$ = 1.1 Hz, 1H), 7.91 (d, $J$ = 6.8 Hz, 1H), 7.47 (t, $J$ = 4.9 Hz, 1H), 7.24 (d, $J$ = 1.1 Hz, 1H), 7.20 (dd, $J$ = 6.8, 7.2 Hz, 1H), 7.15 (d, $J$ = 7.2 Hz, 1H), 1.85 (s, 3H), 1.83 (s, 3H). $^{13}$C NMR (101 MHz, $CDCl_3$): $\delta$ 161.4, 158.5, 140.9, 139.3, 131.0, 129.5, 126.4, 123.3, 121.5, 121.2, 120.9, 120.8, 118.2, 113.5, 18.8, 18.6. HRMS-EI: calcd for $C_{18}H_{14}BrN_3$ [M]$^{+\cdot}$, 351.0366; found, 351.0364.

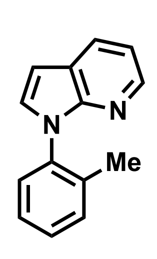


**1-(*o*-tolyl)-1*H*-pyrrolo[2,3-*b*]pyridine (8r)[33]**

According to general procedure 2, $MeBF_3K$ (0.055 g, 0.45 mmol, 1.5 equiv.), $[Cp^*RhCl_2]_2$ (0.009 g, 5 mol%), $AgSbF_6$ (0.021 g, 20 mol%), $Ag_2CO_3$ (0.124 g, 0.45 mmol, 1.5 equiv.) were added. Milling was started at 36 Hz and stopped after 20 min. The mono/di-methylated product ratio is 30/1 and after purification, the title compound was obtained as a colorless solid (0.053 g, 85%). $^1$H NMR (400 MHz, $CDCl_3$): $\delta$ 8.34 (dd, $J$ = 4.7, 1.6 Hz, 1H), 7.99 (dd, $J$ = 7.8, 1.6

Hz, 1H), 7.43 – 7.31 (m, 4H), 7.28 (d, $J$ = 3.5 Hz, 1H), 7.11 (dd, $J$ = 7.9, 4.7 Hz, 1H), 6.63 (d, $J$ = 3.5 Hz, 1H), 2.10 (s, 3H).

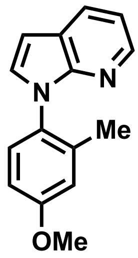


**1-(4-Methoxy-2-methylphenyl)-1*H*-pyrrolo[2,3-*b*]pyridine (8s)**

According to general procedure 2, $MeBF_3K$ (0.055 g, 0.45 mmol, 1.5 equiv.), $[Cp^*RhCl_2]_2$ (0.009 g, 5 mol%), $AgSbF_6$ (0.021 g, 20 mol%), $Ag_2CO_3$ (0.124 g, 0.45 mmol, 1.5 equiv.) were added. Milling was started at 36 Hz and stopped after 20 min. The mono/di-methylated product ratio is 29/1 and after purification, the title compound was obtained as a colorless solid (0.058 g, 81%). $^1H$ NMR (400 MHz, $CDCl_3$): $\delta$ 8.32 (dd, $J$ = 4.7, 1.6 Hz, 1H), 7.97 (dd, $J$ = 7.8, 1.6 Hz, 1H), 7.25 (d, $J$ = 8.5 Hz, 1H), 7.25 (d, $J$ = 3.6 Hz, 1H), 7.09 (dd, $J$ = 7.8, 4.7 Hz, 1H), 6.90 (d, $J$ = 2.9 Hz, 1H), 6.86 (dd, $J$ = 8.5, 2.9 Hz, 1H), 6.60 (d, $J$ = 3.5 Hz, 1H), 3.85 (s, 3H), 2.04 (s, 3H). $^{13}C$ NMR (101 MHz, $CDCl_3$): $\delta$ 159.4, 148.4, 143.7, 137.4, 130.1, 129.5, 129.2, 128.8, 120.3, 116.2, 116.0, 112.0, 100.4, 55.5, 18.2. HRMS-EI: calcd for $C_{15}H_{13}N_2O$ $[M-H]^+$, 237.1022; found, 237.1022.

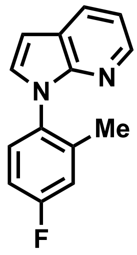


**1-(4-Fluoro-2-methylphenyl)-1*H*-pyrrolo[2,3-*b*]pyridine (8t)**

According to general procedure 2, $MeBF_3K$ (0.055 g, 0.45 mmol, 1.5 equiv.), $[Cp^*RhCl_2]_2$ (0.009 g, 5 mol%), $AgSbF_6$ (0.021 g, 20 mol%), $Ag_2CO_3$ (0.124 g, 0.45 mmol, 1.5 equiv.) were added. Milling was started at 25 Hz and stopped after 2 h. The mono/di-methylated product ratio is 18/1 and after purification, the title compound was obtained as a colorless solid (0.050 g, 74%). $^1H$ NMR (400 MHz, $CDCl_3$): $\delta$ 8.32 (dd, $J$ = 4.7, 1.6 Hz, 1H), 7.98 (dd, $J$ = 7.9, 1.6 Hz, 1H), 7.30 (dd, $J$ = 8.6, 5.4 Hz, 1H), 7.24 (d, $J$ = 3.6 Hz, 1H), 7.13 – 7.07 (m, 2H), 7.02 (ddd, $J$ = 8.4, 8.1, 3.0 Hz, 1H), 6.63 (d, $J$ = 3.5 Hz, 1H), 2.07 (s, 3H). $^{19}F$ NMR (376 MHz, $CDCl_3$) $\delta$ -113.89 – -113.97 (m). $^{13}C$ NMR (101 MHz, $CDCl_3$): $\delta$ 162.2 (d, $J$ = 247.2 Hz), 148.3, 143.8, 138.6 (d, $J$ = 8.6 Hz), 133.2 (d, $J$ = 2.9 Hz), 129.8 (d, $J$ = 9.1 Hz), 129.1, 129.0, 120.4, 117.7 (d, $J$ = 22.5 Hz), 116.3, 113.6 (d, $J$ = 22.6 Hz), 101.0, 18.1 (d, $J$ = 1.5 Hz). HRMS-EI: calcd for $C_{14}H_{10}FN_2$ $[M-H]^+$, 225.0823; found, 225.0821.

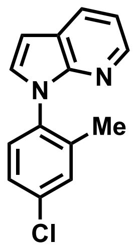

**1-(4-Chloro-2-methylphenyl)-1*H*-pyrrolo[2,3-*b*]pyridine (8u)**

According to general procedure 2, $MeBF_3K$ (0.055 g, 0.45 mmol, 1.5 equiv.), $[Cp^*RhCl_2]_2$ (0.009 g, 5 mol%), $AgSbF_6$ (0.021 g, 20 mol%), $Ag_2CO_3$ (0.124 g, 0.45 mmol, 1.5 equiv.) were added. Milling was started at 25 Hz and stopped after 2 h. The mono/di-methylated product ratio is 22/1 and after purification, the title compound was obtained as a colorless solid (0.077 g, 81%). $^1H$ NMR (400 MHz, $CDCl_3$): $\delta$ 8.32 (dd, $J$ = 4.7, 1.6 Hz, 1H), 7.98 (dd, $J$ = 7.8, 1.6 Hz, 1H), 7.38 (d, $J$ = 2.3, 1H), 7.31 (dd, $J$ = 8.3, 2.0 Hz, 1H), 7.27 (d, $J$ = 8.3, 1H), 7.24 (d, $J$ = 3.5 Hz, 1H), 7.12 (dd, $J$ = 7.9, 4.7 Hz, 1H), 6.64 (d, $J$ = 3.5 Hz, 1H), 2.08 (s, 3H). $^{13}C$ NMR (101 MHz, $CDCl_3$): $\delta$ 148.2, 143.8, 137.8, 135.8, 134.0, 131.0, 129.4, 129.1, 128.8, 126.9, 120.5, 116.4, 101.2, 18.0. HRMS-EI: calcd for $C_{14}H_{10}ClN_2$ [M-H]+, 241.0527; found, 241.0527.

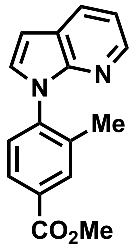

**Methyl 3-methyl-4-(1*H*-pyrrolo[2,3-*b*]pyridin-1-yl)benzoate (8v)**

According to general procedure 2, $MeBF_3K$ (0.055 g, 0.45 mmol, 1.5 equiv.), $[Cp^*RhCl_2]_2$ (0.009 g, 5 mol%), $AgSbF_6$ (0.021 g, 20 mol%), $Ag_2CO_3$ (0.124 g, 0.45 mmol, 1.5 equiv.) were added. Milling was started at 25 Hz and stopped after 2 h. The mono/di-methylated product ratio is 31/1 and after purification, the title compound was obtained as a colorless solid (0.072 g, 90%). $^1H$ NMR (400 MHz, $CDCl_3$): $\delta$ 8.33 (dd, $J$ = 4.7, 1.6 Hz, 1H), 8.08 (d, $J$ = 1.5 Hz, 1H), 8.01 – 7.98 (m, 2H), 7.42 (d, $J$ = 8.1 Hz, 1H), 7.28 (d, $J$ = 3.6 Hz, 1H), 7.13 (dd, $J$ = 7.8, 4.7 Hz, 1H), 6.66 (d, $J$ = 3.6 Hz, 1H), 3.95 (s, 3H), 2.19 (s, 3H). $^{13}C$ NMR (101 MHz, $CDCl_3$): $\delta$ 166.6, 148.1, 143.9, 141.3, 135.9, 132.6, 129.8, 129.1, 128.7, 128.13, 128.05, 120.6, 116.5, 101.5, 52.2, 18.3. HRMS-EI: calcd for $C_{16}H_{13}N_2O_2$ $[M-H]^+$, 265.0972; found, 265.0974.

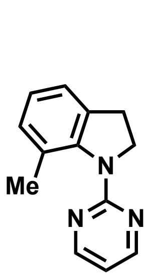

**7-Methyl-1-(pyrimidin-2-yl)indoline (10)**

According to general procedure 2, $MeBF_3K$ (0.055 g, 0.45 mmol, 1.5 equiv.), $[Cp^*RhCl_2]_2$ (0.009 g, 5 mol%), $AgSbF_6$ (0.021 g, 20 mol%), $Ag_2CO_3$ (0.124 g, 0.45 mmol, 1.5 equiv.) were added. Milling was started at 25 Hz and stopped after 2 h. No dimethylated product was

detected and after purification, the title compound was obtained as a colorless solid (0.047 g, 74%). $^{1}$H NMR (400 MHz, $CDCl_3$): $\delta$ 8.44 (d, $J$ = 4.8 Hz, 2H), 7.09 (d, $J$ = 7.3 Hz, 1H), 7.06 (d, $J$ = 6.7 Hz, 1H), 6.98 (dd, $J$ = 7.4, 7.4 Hz, 1H), 6.69 (t, $J$ = 4.8 Hz, 1H), 4.41 (t, $J$ = 7.7 Hz, 2H), 3.07 (t, $J$ = 7.7 Hz, 2H), 2.20 (s, 3H). $^{13}$C NMR (101 MHz, $CDCl_3$): $\delta$ 161.2, 157.6, 142.9, 134.5, 129.5, 128.1, 124.0, 121.9, 112.1, 53.3, 29.9, 20.8. HRMS-ESI: calcd for $C_{13}H_{13}N_3$ $[M]^{+\cdot}$, 211.1104; found, 211.1101.

For variations of these conditions, see Table 2 in the manuscript.

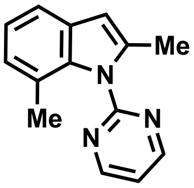


**2,7-Dimethyl-1-(pyrimidin-2-yl)-1*H*-indole (11)**

According to general procedure 2, substrate **9** (0.059 g, 0.3 mmol, 1.0 equiv.), $MeBF_3K$ (0.220 g, 1.8 mmol, 6.0 equiv.), $[Cp^*RhCl_2]_2$ (0.019 g, 10 mol%), $AgSbF_6$ (0.042 g, 40 mol%), $Ag_2CO_3$ (0.248 g, 0.9 mmol, 3.0 equiv.) were added. Milling was carried out at 36 Hz and stopped after 1 h. No monomethylated product was detected and after purification, the title compound was obtained as a colorless solid (0.040 g, 60%). No monomethylated product was detected and after purification, the title compound was obtained as a colorless solid (0.024 g, 54%). $^{1}$H NMR (400 MHz, $CDCl_3$): $\delta$ 8.87 (d, $J$ = 4.9 Hz, 2H), 7.41 (d, $J$ = 7.7 Hz, 1H), 7.31 (t, $J$ = 4.9 Hz, 1H), 7.07 (dd, $J$ = 7.6, 7.4 Hz, 1H), 6.94 (d, $J$ = 7.0 Hz, 1H), 6.40 (s, 1H), 2.35 (d, $J$ = 1.1 Hz, 3H), 1.96 (s, 3H). $^{13}$C NMR (101 MHz, $CDCl_3$): $\delta$ 158.9, 158.3, 137.7, 136.5, 129.7, 124.7, 121.6, 121.2, 119.0, 117.7, 104.1, 20.0, 14.0. HRMS-ESI: calcd for $C_{14}H_{13}N_3$ $[M]^{+\cdot}$, 223.1104; found, 223.1100.

For variations of these conditions, see Table 2 in the manuscript.

## C. Late stage C–H methylation reactions

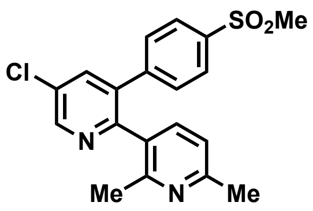


**5-Chloro-2',6'-dimethyl-3-(4-(methylsulfonyl)phenyl)-2,3'-bipyridine (15a)**

According to general procedure 2, $MeBF_3K$ (0.110 g, 0.9 mmol, 3.0 equiv.), $[Cp^*RhCl_2]_2$ (0.009 g, 5 mol%), $AgSbF_6$ (0.021 g, 20 mol%), $Ag_2CO_3$ (0.124 g, 0.45 mmol, 1.5 equiv.) were added. Milling was conducted at 36 Hz and stopped after 0.5 h. No dimethylation product was detected and after purification, the title compound was obtained as a light yellow solid (0.067 g, 60%). $^1H$ NMR (400 MHz, $CDCl_3$): $\delta$ 8.70 (s, 1H), 7.83 (d, $J$ = 7.4 Hz, 2H), 7.77 (s, 1H), 7.32 – 7.27 (m, 3H), 6.94 (d, $J$ = 7.8 Hz, 1H), 3.03 (s, 3H), 2.51 (s, 3H), 2.18 (s, 3H). $^{13}C$ NMR (101 MHz, $CDCl_3$): $\delta$ 158.1, 155.0, 154.6, 148.1, 143.2, 140.0, 138.0, 137.3, 136.1, 131.2, 130.6, 130.1, 127.6, 120.4, 44.4, 24.4, 22.8. HRMS-ESI: calcd for $C_{19}H_{18}ClN_2O_2S$ $[M+H]^+$, 373.0772; found, 373.0769.

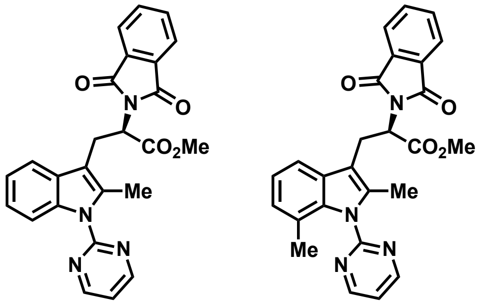


**Methyl-(*R*)-2-(1,3-dioxoisoindolin-2-yl)-3-(2-methyl-1-(pyrimidin-2-yl)-1*H*-indol-3-yl)propanoate (15b) and methyl (*R*)-3-(2,7-dimethyl-1-(pyrimidin-2-yl)-1*H*-indol-3-yl)-2-(1,3-dioxoisoindolin-2-yl)propanoate (15b')**

According to general procedure 1, $MeB(OH)_2$ (0.072 g, 1.2 mmol, 4.0 equiv.) was added and the reaction was stopped after 6 h. The mono/di-methylated product ratio is 3.4/1 according to the crude $^1H$ NMR and after purification, **15b** was obtained as a light yellow solid (0.079 g, 60%). $^1H$ NMR (400 MHz, $CDCl_3$): $\delta$ 8.72 (d, $J$ = 4.7 Hz, 2H), 8.15 (d, $J$ = 7.8 Hz, 1H), 7.74 (d, $J$ = 3.2 Hz, 2H), 7.64 (d, $J$ = 2.9 Hz, 2H), 7.51 (d, $J$ = 7.3 Hz, 1H), 7.10 (m, 3H), 5.27 (dd, $J$ = 9.9, 5.8 Hz, 1H), 3.80 (s, 3H), 3.77 – 3.60 (m, 2H), 2.59 (s, 3H). $^{13}C$ NMR (101 MHz, $CDCl_3$): $\delta$ 169.6, 167.5, 158.2, 157.9, 136.0, 135.1, 133.9, 131.7, 129.3, 123.4, 122.6, 121.7, 117.5, 116.9, 113.5, 111.9, 52.8, 51.9, 24.3, 13.5. HRMS-ESI: calcd for $C_{25}H_{21}N_4O_4$ $[M+H]^+$, 441.1557; found, 441.1549; **15b'** as a light yellow solid (0.020 g, 15%). $^1H$ NMR (400 MHz, $CDCl_3$): $\delta$ 8.81 (d, $J$ = 4.7 Hz, 2H), 7.77 (dd, $J$ = 5.5, 3.0 Hz, 2H), 7.66 (dd, $J$ = 5.6, 3.0 Hz, 2H), 7.43 (d, $J$ = 7.9 Hz, 1H), 7.28 (t, $J$ = 4.9 Hz, 1H), 7.02 (dd, $J$ = 7.6, 7.4 Hz, 1H), 6.87 (d, $J$ = 7.3 Hz, 1H), 5.36 – 5.21 (m, 1H), 3.78 (s, 3H), 3.76 – 3.56 (m, 2H), 2.22 (s, 3H), 1.86 (s, 3H). $^{13}C$ NMR (101 MHz, $CDCl_3$): $\delta$ 169.6, 167.5, 158.9, 158.3, 135.9, 135.5, 133.9, 131.8,

129.5, 125.1, 123.4, 121.4, 121.1, 119.0, 115.8, 109.7, 52.8, 52.1, 24.6, 19.9, 11.3. HRMS-ESI: calcd for $C_{26}H_{23}N_4O_4$ [M+H]$^+$, 455.1714; found, 455.1705.

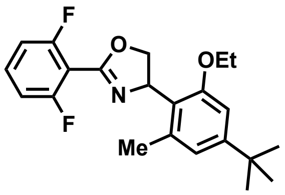


**4-(4-(*tert*-Butyl)-2-ethoxy-6-methylphenyl)-2-(2,6-difluorophenyl)-4,5-dihydrooxazole (15c)**

According to general procedure 2, $MeBF_3K$ (0.110 g, 0.9 mmol, 3.0 equiv.), [Cp*$RhCl_2$]$_2$ (0.009 g, 5 mol%), $AgSbF_6$ (0.021 g, 20 mol%), $Ag_2CO_3$ (0.207 g, 0.75 mmol, 2.5 equiv.) were added. Milling was started at 36 Hz and stopped after 1 h. No dimethylation product was detected and after purification, the title compound was obtained as a colorless solid (0.081 g, 72%). $^1$H NMR (400 MHz, $CDCl_3$): $\delta$ 7.38 (tt, $J$ = 8.4, 6.1 Hz, 1H), 6.96 (dd, $J$ = 8.3, 8.1 Hz, 2H), 6.782 (s, 1H), 6.778 (s, 1H), 5.83 (dd, $J$ = 11.3, 9.1 Hz, 1H), 4.65 (dd, $J$ = 11.3, 7.7 Hz, 1H), 4.55 (dd, $J$ = 9.2, 7.7 Hz, 1H), 4.12 – 3.97 (m, 2H), 2.43 (s, 3H), 1.32 (t, $J$ = 7.0 Hz, 3H), 1.29 (s, 9H). $^9$F NMR (376 MHz, $CDCl_3$) $\delta$ -108.13 – -108.21 (m). $^{13}$C NMR (101 MHz, $CDCl_3$): $\delta$ 161.3 (dd, $J$ = 256.4, 6.2 Hz,), 158.0, 156.6 (t, $J$ = 2.3 Hz), 151.7, 137.4, 131.9 (t, $J$ = 10.5 Hz), 123.0, 120.1, 112.0-111.7 (m), 107.8 (t, $J$ = 17.4 Hz), 107.3, 71.5, 64.3, 63.4, 34.6, 31.3, 20.5, 14.5. HRMS-EI: calcd for $C_{22}H_{25}F_2NO_2$ [M]$^{+\cdot}$, 373.1848; found, 373.1848.

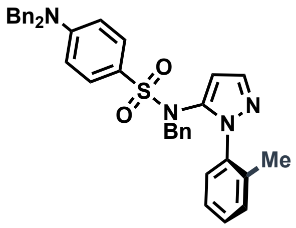


***N*-benzyl-4-(dibenzylamino)-*N*-(1-(o-tolyl)-1*H*-pyrazol-5-yl)benzenesulfonamide (15d)**

According to general procedure 2, $MeBF_3K$ (0.110 g, 0.9 mmol, 3.0 equiv.), [Cp*$RhCl_2$]$_2$ (0.019 g, 10 mol%), $AgSbF_6$ (0.042 g, 40 mol%), $Ag_2CO_3$ (0.124 g, 0.45 mmol, 1.5 equiv.) were added. Milling was conducted at 36 Hz and stopped after 1 h. No dimethylation product was detected and after purification, the title compound was obtained as a colorless solid (0.095 g, 53%). $^1$H NMR (400 MHz, $CDCl_3$): $\delta$ 7.58 (s, 1H), 7.46 (d, $J$ = 8.6 Hz, 2H), 7.39 (dd, $J$ = 7.4, 7.1 Hz, 4H), 7.32 (dd, $J$ = 7.2, 6.8 Hz, 2H), 7.26 – 7.19 (m, 6H), 7.13 (t, $J$ = 7.2 Hz, 3H), 7.05 (dd, $J$ = 7.8, 7.6 Hz, 1H), 6.91 (d, $J$ = 7.6 Hz, 2H), 6.87 – 6.86 (m, 1H), 6.74 (d, $J$ = 8.7 Hz, 2H), 6.09 (s, 1H), 4.76 (s, 4H), 4.38 (s, 2H), 1.71 (s, 3H). $^{13}$C NMR (101 MHz, $CDCl_3$): $\delta$ 152.6, 139.0, 138.9, 137.2, 136.8, 136.8, 134.8, 130.7, 130.5, 129.1, 128.9, 128.7, 128.3, 128.0, 127.9, 127.5, 126.4, 125.6, 123.8, 111.4, 103.2, 56.5, 54.2, 17.4. HRMS-ESI: calcd for $C_{37}H_{35}N_4O_2S$ [M+H]$^+$, 599.2475; found, 599.2479.

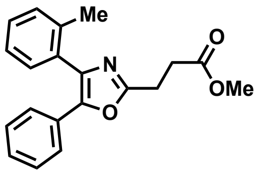


**Methyl 3-(5-phenyl-4-(*o*-tolyl)oxazol-2-yl)propanoate (15e)**

According to general procedure 2, $MeBF_3K$ (0.091 g, 0.75 mmol, 2.5 equiv.), $[Cp^*RhCl_2]_2$ (0.019 g, 10 mol%), $AgSbF_6$ (0.042 g, 40 mol%), $Ag_2CO_3$ (0.207 g, 0.75 mmol, 2.5 equiv.) were added. Milling was conducted at 36 Hz and stopped after 0.5 h. The mono/di-methylated product ratio is 98/2 and after purification, the title compound was obtained as a colorless solid (0.072 g, 75%). $^1H$ NMR (400 MHz, $CDCl_3$): $\delta$ 7.40 – 7.18 (m, 9H), 3.73 (s, 3H), 3.21 (t, $J$ = 7.5 Hz, 2H), 2.93 (t, $J$ = 7.5 Hz, 2H), 2.19 (s, 3H). $^{13}C$ NMR (101 MHz, $CDCl_3$): $\delta$ 172.5, 161.3, 145.7, 137.4, 134.9, 132.2, 130.5, 130.2, 128.71, 128.66, 128.6, 127.8, 126.0, 124.8, 51.9, 30.8, 23.5, 19.8. HRMS-EI: calcd for $C_{20}H_{19}NO_3$ $[M]^{+\cdot}$, 321.1359; found, 321.1360.

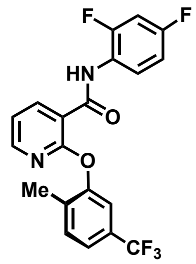


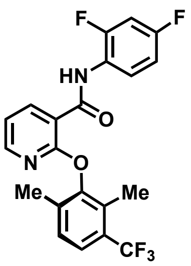


***N*-(2,4-difluorophenyl)-2-(2-methyl-5-(trifluoromethyl)phenoxy)nicotinamide (15f)** and ***N*-(2,4-difluorophenyl)-2-(2,6-dimethyl-3-(trifluoromethyl)phenoxy)nicotinamide (15f')**:

According to general procedure 2, $MeBF_3K$ (0.220 g, 1.8 mmol, 6.0 equiv.), $[Cp^*RhCl_2]_2$ (0.019 g, 10 mol%), $AgSbF_6$ (0.042 g, 40 mol%), $Ag_2CO_3$ (0.248 g, 0.9 mmol, 3.0 equiv.) were added. Milling was conducted at 36 Hz and stopped after 2.5 h. No dimethylation product was detected and after purification, the title compound was obtained as a colorless solid (0.080 g, 65%).

Preparation of **15f** was also conducted on a 10x scale according to a modified version of general procedure 2. Diflufenican (1.183 g, 3.0 mmol, 1.0 equiv.), $MeBF_3K$ (2.195, 18 mmol, 6.0 equiv.), $[Cp^*RhCl_2]_2$ (0.181 g, 0.30 mmol, 10 mol %), $Ag_2CO_3$ (2.480 g, 9.0 mmol, 3.0 equiv.), $AgSbF_6$ (0.411 g, 1.2 mmol, 40 mol %) was added to a 25 mL Teflon$^{TM}$ jar along with one 15 mm SS ball. Milling was conducted at 36 Hz and stopped after 2.5 h. Purification by column chromatography ($SiO_2$, pentane/EtOAc 10:1) afforded the monomethylated product **15f** in 14% isolated yield (0.177 g) as a colorless solid, and dimethylated product **15f'** as a colorless solid in 41% isolated yield (0.518 g).

Data for **15f:** $^1H$ NMR (400 MHz, $CDCl_3$): $\delta$ 10.03 (br s, 1H), 8.72 (dd, $J$ = 7.7, 2.0 Hz, 1H), 8.52 (ddd, $J$ = 9.1, 9.0, 5.9 Hz, 1H), 8.24 (dd, $J$ = 4.8, 2.0 Hz, 1H), 7.51 (dd, $J$ = 8.1, 1.7 Hz, 1H), 7.46 (d, $J$ = 8.0 Hz, 1H), 7.41 (d, $J$ = 1.7 Hz, 1H), 7.25 (dd, $J$ = 7.7, 4.8 Hz, 1H), 6.99 – 6.91 (m, 1H), 6.88 (ddd, $J$ = 11.0, 8.3, 2.8 Hz, 1H), 2.29 (s, 3H). $^{19}F$ NMR (376 MHz, $CDCl_3$) $\delta$ -62.33 (s), -114.54 – -114.62 (m), -125.90 – -125.99 (m). $^{13}C$ NMR (101 MHz, $CDCl_3$): $\delta$ 161.1, 158.7 (dd, $J$ = 246.7, 11.7 Hz), 159.3, 152.8 (dd, $J$ = 246.7, 11.8 Hz), 150.70, 150.65, 142.8, 135.2 (q, $J$ = 1.2 Hz), 132.0, 129.9 (q, $J$ = 33.1 Hz), 123.6 (q, $J$ = 272.1Hz), 123.1-122.8

(m), 119.9, 119.7 (q, $J$ = 3.8 Hz), 116.3, 111.3 (dd, $J$ = 21.7, 3.8 Hz), 103.6 (dd, $J$ = 26.7, 23.3 Hz), 16.5. HRMS-EI: calcd for $C_{20}H_{13}F_5N_2O_2$ $[M]^{+\cdot}$, 408.0892; found, 408.0889.

Data for **15f'**: $^1$H NMR (400 MHz, CDCl3) δ 8.43 (ddd, J = 9.1, 9,0, 5.9 Hz, 1H), 8.11 (br s, 1H), 8.03 (d, J = 5.1 Hz, 1H), 7.45 – 7.34 (m, 3H), 6.96 (d, J = 5.1 Hz, 1H), 6.94 – 6.85 (m, 2H), 2.55 (s, 3H), 2.23 (s, 3H). $^{19}$F NMR (376 MHz, CDCl3) δ -62.33 (s, 3F), -114.22 (qd, J = 8.1, 5.1 Hz, 1F), -126.05 – -126.17 (m, 1F). $^{13}$C NMR (101 MHz, $CDCl_3$) δ 163.4, 160.1 (d, $J$ = 11.7 Hz), 159.1, 157.6 (d, $J$ = 11.6 Hz), 153.9 (d, $J$ = 11.9 Hz), 151.5, 150.8, 148.0, 135.0 (q, $J$ = 1.3 Hz), 131.7, 129.5 (d, $J$ = 33.1 Hz), 123.0 (dd, $J$ = 9.1, 2.3 Hz), 122.2 (q, $J$ = 4.1 Hz), 121.6, 119.2 (q, $J$ = 3.9 Hz), 111.3 (dd, $J$ = 21.8, 3.8 Hz), 103.7 (dd, $J$ = 26.7, 23.3 Hz), 19.7, 16.5. MS-APCI: calcd for $C_{21}H_{15}F_5N_2O_2$ $[M+H]^+$, 423.1; found, 423.1

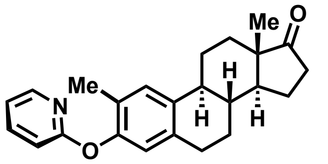


**(8*R*,9*S*,13*S*,14*S*)-2,13-Dimethyl-3-(pyridin-2-yloxy)-6,7,8,9,11,12,13,14,15,16-decahydro-17*H*-cyclopenta[*a*]phenanthren-17-one (15g)**

According to general procedure 2, $MeBF_3K$ (0.220 g, 1.8 mmol, 6.0 equiv.), $[Cp^*RhCl_2]_2$ (0.009 g, 5 mol%), $AgSbF_6$ (0.021 g, 20 mol%), $Ag_2CO_3$ (0.248 g, 0.9 mmol, 3.0 equiv.) were added. Milling was conducted at 36 Hz and stopped after 2 h. No dimethylation product was detected and after purification, the title compound was obtained as a colorless solid (0.063 g, 58%). $^1$H NMR (400 MHz, $CDCl_3$): $\delta$ 8.18 (dd, $J$ = 5.1, 2.0 Hz, 1H), 7.65 (ddd, $J$ = 8.8, 7.2, 2.1 Hz, 1H), 7.17 (s, 1H), 6.94 (dd, $J$ = 7.1, 5.0 Hz, 1H), 6.86 (d, $J$ = 8.3 Hz, 1H), 6.79 (s, 1H), 2.90 – 2.83 (m, 2H), 2.55 – 2.40 (m, 2H), 2.35 – 2.25 (m, 1H), 2.20 – 1.95 (m, 6H), 1.70 – 1.37 (m, 7H), 0.92 (s, 3H). $^{13}$C NMR (101 MHz, $CDCl_3$): $\delta$ 220.9, 163.9, 150.0, 147.8, 139.2, 136.4, 135.4, 128.3, 127.6, 121.7, 117.7, 110.7, 50.5, 48.0, 44.2, 38.1, 35.9, 31.6, 29.0, 26.5, 25.8, 21.6, 16.2, 13.8. HRMS-ESI: calcd for $C_{24}H_{28}NO_2$ $[M+H]^+$, 362.2115; found, 362.2112.

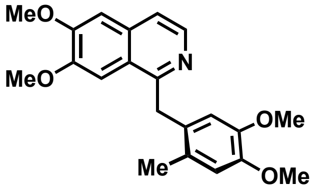


**1-(4,5-Dimethoxy-2-methylbenzyl)-6,7-dimethoxyisoquinoline (15h)**

According to general procedure 2, $MeBF_3K$ (0.110 g, 0.9 mmol, 3.0 equiv.), $[Cp^*RhCl_2]_2$ (0.009 g, 5 mol%), $AgSbF_6$ (0.021 g, 20 mol%), $Ag_2CO_3$ (0.207 g, 0.75 mmol, 2.5 equiv.) were added. Milling was started at 36 Hz and stopped after 2 h. Dimethylation product was detected as the only product and after purification, the title compound was obtained as a light brown solid (0.083 g, 78%). $^1$H NMR (400 MHz, $CDCl_3$): $\delta$ 8.36 (d, $J$ = 5.7 Hz, 1H), 7.43 (d, $J$ = 5.7 Hz, 1H), 7.21 (s, 1H), 7.06 (s, 1H), 6.71 (s, 1H), 6.51 (s, 1H), 4.50 (s, 2H), 4.01 (s, 3H), 3.86 (s, 3H), 3.84 (s, 3H), 3.62 (s, 3H), 2.34 (s, 3H). $^{13}$C NMR (101 MHz, $CDCl_3$): $\delta$ 157.7, 152.4, 149.8, 147.2, 147.0, 140.9, 133.3, 129.6, 128.0, 123.0, 118.6, 113.6, 112.9, 105.3, 103.9, 56.0, 55.86, 55.85, 55.8, 39.5, 19.6. HRMS-ESI: calcd for $C_{21}H_{24}NO_4$ $[M+H]^+$, 354.1700; found, 354.1704.

## 4. Synthesis of Organometallic Complexes

Based on the reported method[34] for synthesizing rhodacycle **4a**, an optimized reaction condition (**general procedure 3**) could provide a better result.

**General procedure 3 (solution-based):** To a 25 ml flash tube was filled with $[Cp^*RhCl_2]_2$ (0.123 g, 0.2 mmol, 1.0 equiv.), substrate (0.4 mmol, 2.0 equiv), KOAc (0.118 g, 1.2 mmol, 6.0 equiv), 1,2-dichloroethane (10 mL) and the resulting mixture was stirred at 80 °C. The reaction mixture was cooled to rt and the solvent was removed under reduced pressure. The reaction mixture was purified by flash column chromatography on aluminium oxide ($CH_2Cl_2$/MeOH = 10:1) to give the corresponding compound.

**General procedure 4 (ball milling):** A Teflon™ milling vessel (14 mL internal volume) was charged with $[Cp^*RhCl_2]_2$ (0.062 g, 0.1 mmol, 1.0 equiv.), substrate (0.21 mmol, 2.1 equiv), KOAc (0.118 g, 1.2 mmol, 6.0 equiv), methanol (10 equiv, 40.5 µL) and a stainless steel ball (15 mm diameter). Milling was conducted at 36 Hz and for 1 h. The reaction mixture was purified by flash column chromatography on aluminium oxide ($Et_2O$, then $CH_2Cl_2$/MeOH = 500:1) to give the pure product.

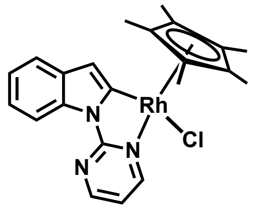


**Rhodacycle 4a:**[35]

According to general procedure 4, **4a** was obtained as an orange solid (0.071 g, 76%; general procedure 3: 96% for 24 hours). $^1$H NMR (400 MHz, $CDCl_3$): $\delta$ 8.67 (dd, $J$ = 4.7, 2.3 Hz, 1H), 8.65 (dd, $J$ = 5.6, 2.3 Hz, 1H), 8.43 (dd, $J$ = 7.9, 1.0 Hz, 1H), 7.42 (dd, $J$ = 7.8, 1.1 Hz, 1H), 7.15 (ddd, $J$ = 7.5, 7.4, 1.2 Hz, 1H), 7.06 (ddd, $J$ = 7.7, 7.2, 1.3 Hz, 1H), 6.94 (dd, $J$ = 5.6, 4.6 Hz, 1H), 6.63 (s, 1H), 1.74 (s, 15H).

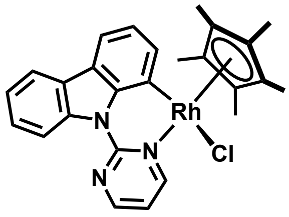


**Rhodacycle 7a:**

According to general procedure 4, **7a** was obtained as an orange solid (0.090 g, 87%; general procedure 3: 24% for 48 hours). $^1$H NMR (500 MHz, $CDCl_3$): $\delta$ 9.28 (dd, $J$ = 5.7, 2.4 Hz, 1H), 8.73 (ddd, $J$ = 8.4, 0.9, 0.8 Hz, 1H), 8.66 (dd, $J$ = 4.4, 2.4 Hz, 1H), 7.95 (ddd, $J$ = 7.4, 1.1, 0.9 Hz, 1H), 7.61 (dd, $J$ = 7.4, 1.1 Hz, 1H), 7.54 (dd, $J$ = 7.5, 1.2 Hz, 1H), 7.45 (ddd, $J$ = 8.4, 7.2, 1.4 Hz, 1H), 7.35 (ddd, $J$ = 7.6, 7.5, 1.0 Hz, 1H), 7.29 (dd, $J$ = 7.5, 7.4 Hz, 1H), 7.02 (dd, $J$ = 5.6, 4.4 Hz, 1H), 1.41 (s, 15H). $^{13}$C NMR (125 MHz, $CDCl_3$): $\delta$ 163.5 (d, $J$ = 1.8 Hz), 157.7, 155.8, 142.4 (d, $J$ = 30.7 Hz), 140.2 (d, $J$ = 2.1 Hz), 138.8, 136.8 (d, $J$ = 1.9 Hz), 129.1, 126.0,

125.6, 123.4, 122.0, 119.9, 119.3, 115.3, 114.2, 96.2 (d, $J$ = 6.5 Hz), 8.8. HRMS-Nanospray: calcd for $C_{26}H_{25}N_3Rh$ [M-Cl]$^+$, 482.1104; found, 482.1107.

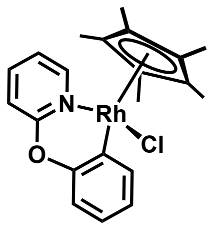


**Rhodacycle 7b:**

According to general procedure 4, **7b** was obtained as an orange solid (0.081 g, 91%; general procedure 3: 2% for 48 hours). $^1$H NMR (400 MHz, $CDCl_3$): $\delta$ 8.75 (dd, $J$ = 5.7, 1.9 Hz, 1H), 7.73 (ddd, $J$ = 8.3, 7.2, 1.9 Hz, 1H), 7.61 (d, $J$ = 7.4 Hz, 1H), 7.15 – 7.01 (m, 3H), 7.01 – 6.90 (m, 2H), 1.47 (s, 15H). $^{13}$C NMR (101 MHz, $CDCl_3$): $\delta$ 162.6, 152.9 (d, $J$ = 2.5 Hz), 152.7 (d, $J$ = 1.7 Hz), 147.1 (d, $J$ = 30.8 Hz), 140.1, 139.8 (d, $J$ = 2.2 Hz), 126.0 (d, $J$ = 0.7 Hz), 123.9, 120.0, 115.6, 114.3, 95.7 (d, $J$ = 6.5 Hz), 8.9. HRMS-Nanospray: calcd for $C_{21}H_{24}NOClRh$ [M+H]$^+$, 444.0601; found, 444.0597.

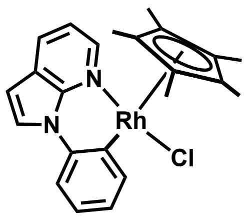


**Rhodacycle 7c:**

According to general procedure 4, **7c** was obtained as an orange solid (0.082 g, 88%; general procedure 3: 16% for 48 hours). $^1$H NMR (500 MHz, $CDCl_3$): $\delta$ 8.65 (d, $J$ = 5.2 Hz, 1H), 7.94 (dd, $J$ = 7.7, 1.4 Hz, 1H), 7.92 (d, $J$ = 3.7 Hz, 1H), 7.84 – 7.76 (m, 1H), 7.19 (dd, $J$ = 7.7, 5.4 Hz, 1H), 7.16 – 7.11 (m, 1H), 7.09 – 7.00 (m, 2H), 6.63 (d, $J$ = 3.8 Hz, 1H), 1.31 (s, 15H). $^{13}$C NMR (101 MHz, $CDCl_3$): $\delta$ 150.1 (d, $J$ = 33.2 Hz), 148.2 (d, $J$ = 1.6 Hz), 145.0, 141.7 (d, $J$ = 2.4 Hz), 136.4, 130.0, 126.0, 125.6, 123.8, 122.6, 118.3, 114.8, 102.8, 95.9 (d, $J$ = 6.4 Hz), 8.7. HRMS-Nanospray: calcd for $C_{23}H_{24}N_2Rh$ [M-Cl]$^+$, 431.0995; found, 431.0993.

# 5. Mechanistic experiments

To test their competence in catalysis, C–H methylation reactions were performed using corresponding rhodacycles as the catalyst.

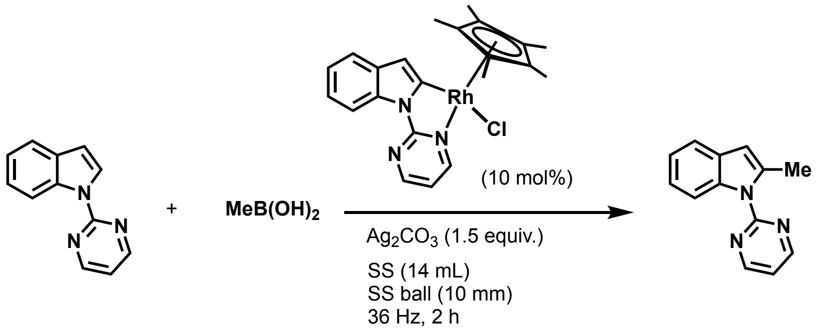


**Procedure:** A stainless steel vessel (14 mL internal volume) was charged with 1-(pyrimidin-2-yl)-1*H*-indole (0.059 g, 0.3 mmol, 1.0 equiv.), $MeB(OH)_2$ (0.036 g, 0.6 mmol, 2.0 equiv.), rhodacycle **4a** (0.014 g, 0.03 mmol, 10 mol%), $Ag_2CO_3$ (0.124 g, 0.45 mmol, 1.5 equiv.) and a stainless steel ball (10 mm diameter). Milling was conducted at 36 Hz for 2 h. The reaction mixture was cooled to rt, washed out with EtOAc or MeOH and filtered through a thin layer of Celite (3 cm) using EtOAc (200 mL) as the eluent. The mixture was concentrated under reduced pressure and 1,3,5-trimethoxybenzene (0.1 M in EtOAc) was added as an internal standard to check reaction progress by $^1H$ NMR spectroscopy. The crude mixture was further purified by flash column chromatography on silica gel to give the corresponding product (0.058 g, 92% yield). (By comparison, the corresponding reaction using $[Cp^*RhCl_2]_2$ (5 mol%) as catalyst gave 82% yield.)

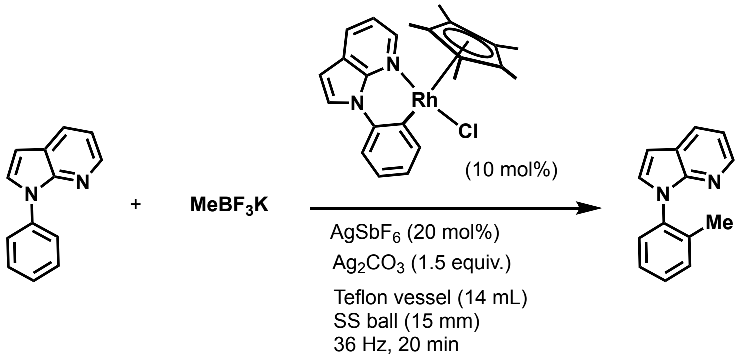


**Procedure:** A Teflon™ vessel (14 mL) was filled with 1-phenyl-*1H*-pyrrolo[2,3-*b*]pyridine (0.058 g, 0.3 mmol, 1.0 equiv.), $MeBF_3K$ (0.055 g, 0.45 mmol, 1.5 equiv.), rhodacycle **7c** (0.014 g, 0.03 mmol, 10 mol%), $AgSbF_6$ (0.021 g, 0.06 mmol, 20 mol%), $Ag_2CO_3$ (0.124 g, 0.45 mmol, 1.5 equiv.) and a stainless steel ball (15 mm diameter). Milling was conducted at 36 Hz for 20 min. The reaction mixture was cooled to rt, washed out with EtOAc or MeOH and then filtered through a thin layer of Celite (3 cm) using EtOAc (200 mL) as the eluent. The mixture was concentrated under reduced pressure and 1,3,5-trimethoxybenzene (0.1 M in EtOAc) was added as an internal standard to check reaction progress by $^1H$ NMR spectroscopy. The crude mixture was further purified by flash column chromatography on silica gel to give the corresponding product (0.049 g, 78% yield). (By comparison, the corresponding reaction using $[Cp^*RhCl_2]_2$ (5 mol%) as catalyst gave 85% yield.)

Reductive elimination reactions:

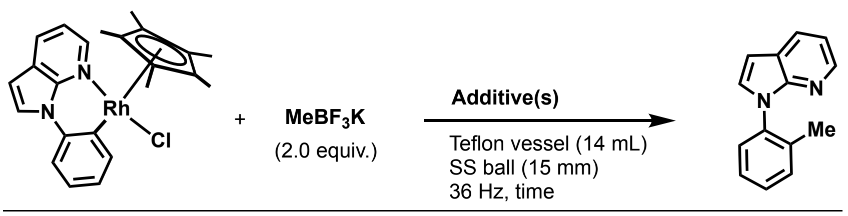


| Entry | Additive(s) (equiv.) | time (min) | Spec. yield (%) |
|---|---|---|---|
| 1 | None | 60 | 0 |
| 2 | $AgSbF_6$ (1.0) | 20 | 20 |
| 3 | $KPF_6$ (1.0) | 20 | 9 |
| 4 | $AgSbF_6$ (1.0) + 7-aza-1-methylindole (1.0) | 20 | 33 |
| 5 | $AgSbF_6$ (1.0) + 7-aza-1-phenylindole (1.0) | 20 | 20 |
| 6 | $AgSbF_6$ (1.0) + 7-aza-1-methylindole (3.0) | 20 | 5 |
| 7 | $AgSbF_6$ (1.0) + pyridine (1.0) | 20 | 2 |

**Procedure:** A Teflon™ vessel (14 mL) was filled with complex **7c** (0.023 g, 0.05 mmol, 1.0 equiv.), $MeBF_3K$ (0.012 g, 0.1 mmol, 2.0 equiv.), additive(s) and a stainless steel ball (15 mm diameter). Milling was conducted at 36 Hz for the indicated time and cooled to rt. The reaction mixture was cooled to room temperature, washed out with EtOAc or MeOH and then filtered through a thin layer of aluminium oxide (3 cm) using $CH_2Cl_2$ (50 mL) and MeOH (50 mL) as the eluent. The mixture was concentrated under reduced pressure and 1,3,5-trimethoxybenzene (0.1 M in EtOAc) was added as an internal standard to check reaction progress by $^1H$ NMR spectroscopy.

H/D exchange experiment:

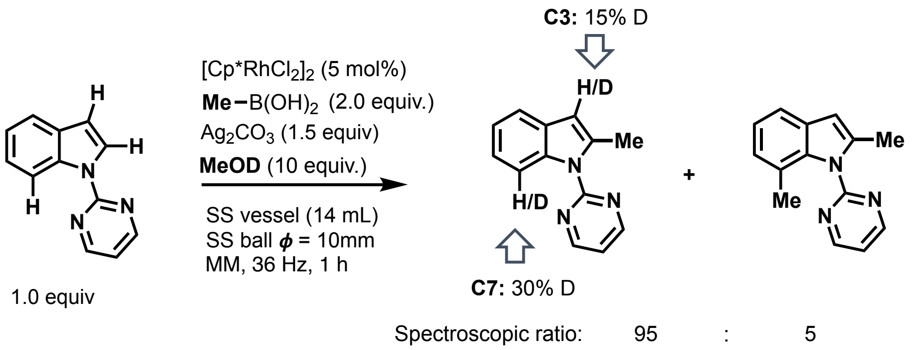


**Procedure:** A Stainless steel vessel (14 mL) was charged with 1-(pyrimidin-2-yl)-1*H*-indole (58.6 mg, 0.3 mmol, 1.0 equiv.), $MeB(OH)_2$ (35.9 mg, 0.6 mmol, 2.0 equiv.), $Ag_2CO_3$ (124 mg, 0.45 mmol, 1.5 equiv.), $Cp^*RhCl_2]_2$ (9.3 mg, 0.015 mmol, 5 mol%). MeOD (0.123 mL, 3.0 mmol, 10 equiv.) was added in one portion along with a stainless steel ball (10 mm diameter). Milling was conducted at 36 Hz for 1 h. The reaction mixture was cooled to rt, washed out with EtOAc and filtered (Celite plug) using EtOAc (200 mL) as the eluent. The mixture was concentrated under reduced pressure and analyzed using $^1H$ NMR spectroscopy. Complete conversion of the starting material was observed to have occurred, resulting in a mixture of only **5a** and **11** (**5a**/**11** = 95:5). Analysis of the signals corresponding to **5a** revealed H/D exchange had occurred at C3 (15% D incorporation) and C7 (30% D incorporation).

Competition reaction: indole-pym based substrates

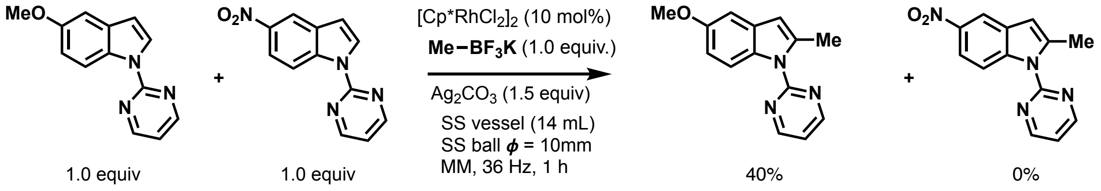


A stainless steel vessel (14 mL) was charged with 5-methoxy-1-(pyrimidin-2-yl)-1*H*-indole (0.034 g, 0.15 mmol, 1.0 equiv.), 5-nitro-1-(pyrimidin-2-yl)-1*H*-indole (0.036 g, 0.15 mmol, 1.0 equiv.), $MeBF_3K$ (0.018 g, 0.15 mmol, 1.0 equiv.), $[Cp^*RhCl_2]_2$ (0.009 g, 0.015 mmol, 10 mol%), $Ag_2CO_3$ (0.062 g, 0.225 mmol, 1.5 equiv.) and a stainless steel ball (10 mm diameter). Milling was conducted at 36 Hz for 1 h and the reaction mixture cooled to rt. The reaction mixture was washed out with EtOAc or MeOH, then filtered through a thin layer of Celite (3 cm) using EtOAc (200 mL) as the eluent. 1,3,5-Trimethoxybenzene (0.1 M in EtOAc) was added as the internal standard. The solvent was removed under reduced pressure and the mixture was analysed by $^1$H NMR spectroscopy. Product **5b** was formed in 40% NMR yield, while product **5e** was not formed at all.

Competition reaction: phenoxypyridine-based substrates

Phenoxypyridine competition reaction 1

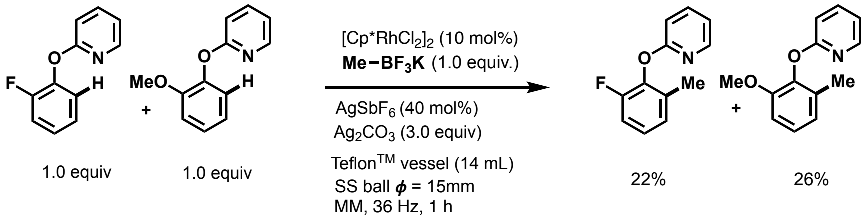


Phenoxypyridine competition reaction 2

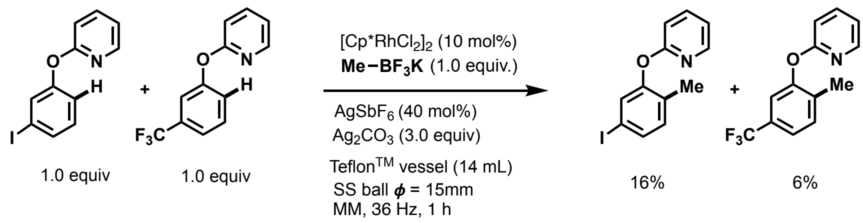


A Teflon$^{TM}$ vessel (14 mL internal volume) was charged with the appropriate competing phenoxypyridine derivatives (0.15 mmol, 1.0 equiv. each), $MeBF_3K$ (0.018 g, 0.15 mmol, 1.0 equiv.), $[Cp^*RhCl_2]_2$ (0.009 g, 0.015 mmol, 10 mol%), $AgSbF_6$ (0.021 g, 0.06 mmol, 40 mol%), $Ag_2CO_3$ (0.124 g, 0.45 mmol, 3.0 equiv.) and a stainless steel ball (15 mm). Milling was conducted at 36 Hz for 1 h after which the reaction was cooled to rt and the vessel washed out with EtOAc. The mixture was filtered through a thin layer of Celite (3 cm) using EtOAc (200 mL) as the eluent. 1,3,5-Trimethoxybenzene (0.1 M in EtOAc) was added as the internal standard and the solvent was removed under reduced pressure. The mixture was analysed by $^1$H NMR spectroscopy.

In the first phenoxy pyridine competition experiment products **8c** and **8d** were formed in a 26% and 22% yield, respectively. In the second competition reaction, product **8h** was formed in 16% spectroscopic yield, while product **8i** was formed in 6% yield.

# 6. Crystallographic data

**X-ray crystallography:** All the measurements performed at 170 K using graphite-monochromatized Mo Kα radiation using a Bruker D8 APEX-II equipped with a CCD camera. Data reduction was performed with SAINT. Absorption corrections for the area detector were performed using SADABS. The structure was solved by direct methods (SHELXT) and refined by full-matrix least-squares techniques against F2 using all data (SHELXL) using the OLEX2 suit of programs. All non-hydrogen atoms were refined with anisotropic displacement parameters if not stated otherwise. Hydrogen atoms constrained in geometric positions to their parent atoms. CCDC 2017322-2017324 contain the supplementary crystallographic data for this paper. These data can be obtained free of charge from The Cambridge Crystallographic Data Centre via www.ccdc.cam.ac.uk/structures.

**Table 1 Crystal data and structure refinement for 7a.**

| | |
|---|---|
| Identification code | 2017324 |
| Empirical formula | $C_{26}H_{25}ClN_3Rh$ |
| Formula weight | 517.85 |
| Temperature/K | 170 |
| Crystal system | monoclinic |
| Space group | $P2_1/n$ |
| a/Å | 8.2604(12) |
| b/Å | 19.114(3) |
| c/Å | 13.797(2) |
| α/° | 90 |
| β/° | 96.948(2) |
| γ/° | 90 |
| Volume/Å$^3$ | 2162.5(5) |
| Z | 4 |
| $\rho_{calc}$g/cm$^3$ | 1.591 |
| μ/mm$^{-1}$ | 0.933 |
| F(000) | 1056.0 |
| Crystal size/mm$^3$ | 0.11 × 0.11 × 0.09 |
| Radiation | $MoK_\alpha$ ($\lambda$ = 0.71073) |
| 2Θ range for data collection/° | 3.658 to 54.388 |
| Index ranges | $-10 \leq h \leq 10, -24 \leq k \leq 24, -17 \leq l \leq 17$ |
| Reflections collected | 24136 |
| Independent reflections | 4809 [$R_{int}$ = 0.0492, $R_{sigma}$ = 0.0388] |
| Data/restraints/parameters | 4809/0/285 |
| Goodness-of-fit on $F^2$ | 1.018 |
| Final R indexes [I>=2σ (I)] | $R_1$ = 0.0287, $wR_2$ = 0.0618 |
| Final R indexes [all data] | $R_1$ = 0.0406, $wR_2$ = 0.0668 |
| Largest diff. peak/hole / e Å$^{-3}$ | 0.36/-0.35 |

**Table 2 Crystal data and structure refinement for 7b.**

| | |
|---|---|
| Identification code | 2017323 |
| Empirical formula | $C_{21}H_{23}ClNORh$ |
| Formula weight | 443.76 |
| Temperature/K | 170 |
| Crystal system | triclinic |
| Space group | P-1 |
| a/Å | 8.7855(9) |
| b/Å | 13.8162(15) |
| c/Å | 15.9543(17) |
| α/° | 87.477(4) |
| β/° | 86.101(4) |
| γ/° | 89.897(3) |
| Volume/Å$^3$ | 1930.2(4) |
| Z | 4 |
| $\rho_{calc}$g/cm$^3$ | 1.527 |
| μ/mm$^{-1}$ | 1.031 |
| F(000) | 904.0 |
| Crystal size/mm$^3$ | 0.09 × 0.07 × 0.04 |
| Radiation | MoKα (λ = 0.71073) |
| 2Θ range for data collection/° | 3.822 to 52.744 |
| Index ranges | $-10 \le h \le 10, -17 \le k \le 17, -19 \le l \le 19$ |
| Reflections collected | 60454 |
| Independent reflections | 7878 [$R_{int}$ = 0.1109, $R_{sigma}$ = 0.0705] |
| Data/restraints/parameters | 7878/0/461 |
| Goodness-of-fit on F$^2$ | 1.029 |
| Final R indexes [I>=2σ (I)] | $R_1$ = 0.0351, w$R_2$ = 0.0753 |
| Final R indexes [all data] | $R_1$ = 0.0514, w$R_2$ = 0.0826 |
| Largest diff. peak/hole / e Å$^{-3}$ | 0.70/-0.78 |

**Table 3 Crystal data and structure refinement for 17c.**

| | |
|---|---|
| Identification code | 2017322 |
| Empirical formula | $C_{23}H_{24}ClN_2Rh$ |
| Formula weight | 466.80 |
| Temperature/K | 170 |
| Crystal system | orthorhombic |
| Space group | $P2_12_12_1$ |
| a/Å | 8.1016(9) |
| b/Å | 14.9741(17) |
| c/Å | 16.4139(18) |
| α/° | 90 |
| β/° | 90 |
| γ/° | 90 |
| Volume/Å$^3$ | 1991.2(4) |

| | |
|---|---|
| Z | 4 |
| $\rho_{calc}$g/cm$^3$ | 1.557 |
| μ/mm$^{-1}$ | 1.002 |
| F(000) | 952.0 |
| Crystal size/mm$^3$ | 0.14 × 0.11 × 0.09 |
| Radiation | MoKα (λ = 0.71073) |
| 2Θ range for data collection/° | 3.682 to 54.66 |
| Index ranges | -10 ≤ h ≤ 10, -19 ≤ k ≤ 19, -21 ≤ l ≤ 21 |
| Reflections collected | 69811 |
| Independent reflections | 4475 [$R_{int}$ = 0.0475, $R_{sigma}$ = 0.0207] |
| Data/restraints/parameters | 4475/0/249 |
| Goodness-of-fit on F$^2$ | 1.100 |
| Final R indexes [I>=2σ (I)] | $R_1$ = 0.0320, w$R_2$ = 0.0735 |
| Final R indexes [all data] | $R_1$ = 0.0365, w$R_2$ = 0.0762 |
| Largest diff. peak/hole / e Å$^{-3}$ | 1.35/-0.89 |
| Flack parameter | -0.012(9) |

# 7. References:


[1] M. Barday, C. Janot, N. R. Halcovitch, J. Muir, C. Aissa, *Angew. Chem. Int. Ed.l* **2017** *56*, 13117-13121.
[2] H. Wu, T. Liu, M. Cui, Y. Li, J. Jian, H. Wang, Z. Zeng, *Org. Biomol. Chem.* **2017** *15*, 536-540.
[3] C. Sollert, K. Devaraj, A. Orthaber, P. J. Gates, L. T. Pilarski, *Chem. Eur. J.* **2015** *21*, 5380-5386.
[4] J. Shi, B. Zhou, Y. Yang, Y. Li, *Org. Biomol. Chem.* **2012** *10*, 8953-8955.
[5] S. Xu, X. Huang, X. Hong, B. Xu, *Org. Lett.* **2012** *14*, 4614-4617.
[6] X.-M. Yan, X.-R. Mao, Z.-Z. Huang, *Heterocycles* **2011** *83*, 1371-1376.
[7] J. Yao, R. Feng, Z. Wu, Z. Liu, Y. Zhang, *Adv. Synth. Catal.* **2013** *355*, 1517-1522.
[8] M. Tobisu, J. Zhao, H. Kinuta, T. Furukawa, T. Igarashi, N. Chatani, *Adv. Synth. Catal.* **2016** *358*, 2417-2421.
[9] H. Kinuta, M. Tobisu, N. Chatani, *J. Am. Chem. Soc.* **2015** *137*, 1593-1600.
[10] L. Zhu, X. Cao, R. Qiu, T. Iwasaki, V. P. Reddy, X. Xu, S.-F. Yin, N. Kambe, *RSC Adv.* **2015** *5*, 39358-39365.
[11] J. A. Leitch, C. J. Heron, J. McKnight, G. Kociok-Köhn, Y. Bhonoah, C. G. Frost, *Chem. Commun.* **2017** *53*, 13039-13042.
[12] R. Horikawa, C. Fujimoto, R. Yazaki, T. Ohshima, *Chem. Eur. J.* **2016** *22*, 12278-12281.
[13] K. Qiao, D. Zhang, K. Zhang, X. Yuan, M.-W. Zheng, T.-F. Guo, Z. Fang, L. Wan, K. Guo, *Org. Chem. Front.* **2018** *5*, 1129-1134.
[14] Q.-B. Zhang, Y.-L. Ban, P.-F. Yuan, S.-J. Peng, J.-G. Fang, L.-Z. Wu, Q. Liu, *Green Chem.* **2017** *19*, 5559-5563.
[15] G. A. Molander, C. S. Yun, M. Ribagorda, B. Biolatto, *J. Org. Chem.* **2003** *68*, 5534-5539.
[16] B. Bhayana, B. P. Fors, S. L. Buchwald, *Org. Lett.* **2009** *11*, 3954-3957.
[17] C. M. G. Azevedo, K. R. Watterson, E. T. Wargent, S. V. F. Hansen, B. D. Hudson, M. A. Kępczyńska, J. Dunlop, B. Shimpukade, E. Christiansen, G. Milligan, C. J. Stocker, T. Ulven, *J. Med. Chem.* **2016** *59*, 8868-8878.
[18] T. Markovic, P. R. D. Murray, B. N. Rocke, A. Shavnya, D. C. Blakemore, M. C. Willis, *J. Am. Chem. Soc.* **2018** *140*, 15916-15923.
[19] X. Yang, H. Xing, Y. Zhang, Y. Lai, Y. Zhang, Y. Jiang, D. Ma, *Chinese J. Chem.* **2012** *30*, 875-880.
[20] G. Qian, X. Hong, B. Liu, H. Mao, B. Xu, *Org. Lett.* **2014** *16*, 5294-5297.
[21] a) I. Güell, X. Ribas, *Eur. J. Org. Chem.* **2014** *2014*, 3188-3195; b) D. Wang, D. Kuang, F. Zhang, Y. Liu, S. Ning, *Tetrahedron Lett.* **2014** *55*, 7121-7123.
[22] D. G. Yu, F. de Azambuja, F. Glorius, *Angew. Chem. Int. Ed.* **2014** *53*, 2754-2758.
[23] H. Wang, S. Yu, Z. Qi, X. Li, *Org. Lett.* **2015** *17*, 2812-2815.
[24] D. Tu, X. Cheng, Y. Gao, P. Yang, Y. Ding, C. Jiang, *Org. Biomol. Chem.* **2016** *14*, 7443-7446.
[25] T. Yamamoto, K. Muto, M. Komiyama, J. Canivet, J. Yamaguchi, K. Itami, *Chem. Eur. J.* **2011** *17*, 10113-10122.
[26] H. W. Lee, A. S. C. Chan, F. Y. Kwong, *Tetrahedron Lett.* **2009** *50*, 5868-5871.
[27] H. Huang, Z. M. Strater, M. Rauch, J. Shee, T. J. Sisto, C. Nuckolls, T. H. Lambert, *Angew. Chem. Int. Ed.* **2019** *58*, 13318-13322.
[28] A. T. Londregan, S. Jennings, L. Wei, *Org. Lett.* **2011** *13*, 1840-1843.
[29] C. Zhang, P. Sun, *J. Org. Chem.* **2014** *79*, 8457-8461.
[30] C. Gao, H. Li, M. Liu, J. Ding, X. Huang, H. Wu, W. Gao, G. Wu, *RSC Adv.* **2017** *7*, 46636-46643.
[31] Q. Wu, C. Du, Y. Huang, X. Liu, Z. Long, F. Song, J. You, *Chem. Sci.* **2015** *6*, 288-293.
[32] X. Chen, J. J. Li, X. S. Hao, C. E. Goodhue, J. Q. Yu, *J. Am. Chem. Soc.* **2006** *128*, 78-79.
[33] A. Mishra, T. K. Vats, I. Deb, *J. Org. Chem.* **2016** *81*, 6525-6534.
[34] X. Zhou, Y. Pan, X. Li, *Angew. Chem. Int. Ed.* **2017** *56*, 8163-8167.
[35] J. Xia, Z. Huang, X. Zhou, X. Yang, F. Wang, X. Li, *Org. Lett.* **2018** *20*, 740-743.

## 8. NMR Spectra

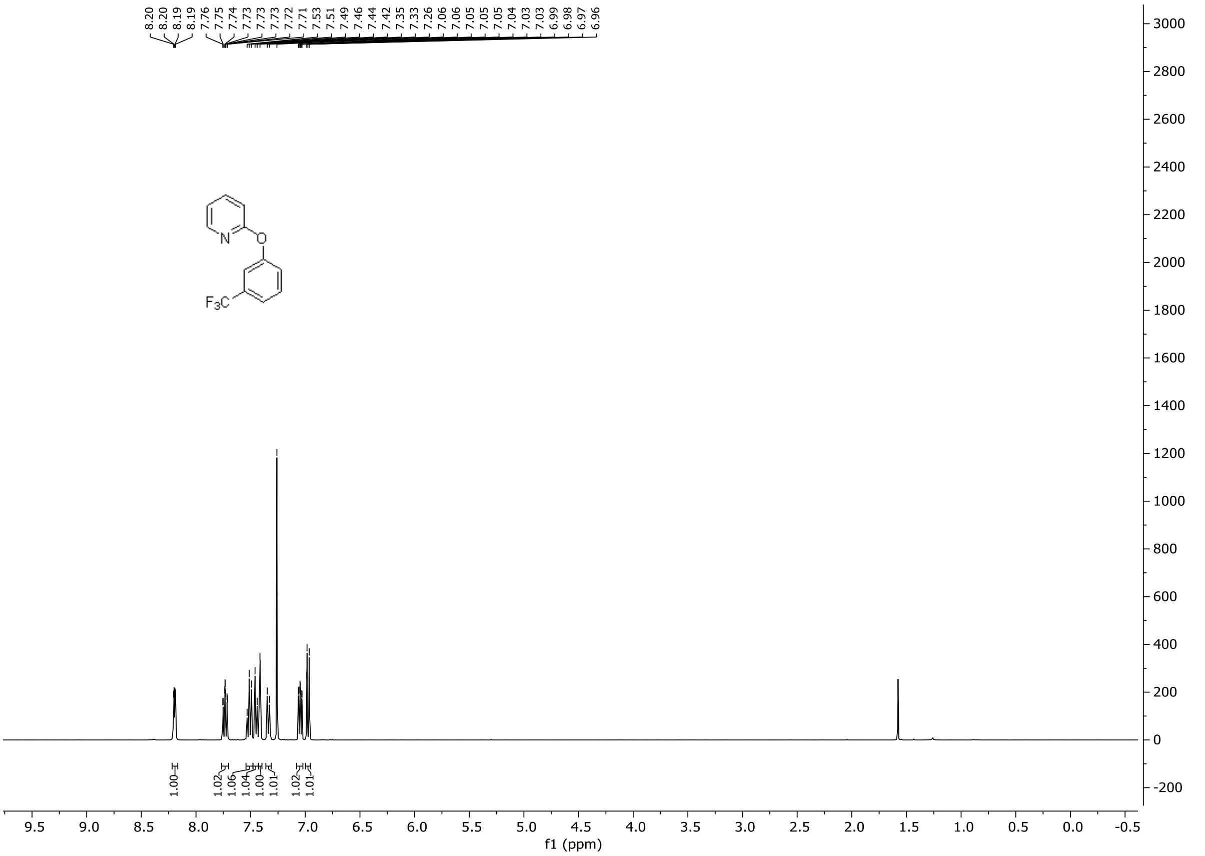
8.20 8.20 8.19 8.19 7.76 7.75 7.74 7.73 7.73 7.73 7.72 7.71 7.53 7.51 7.49 7.46 7.44 7.42 7.35 7.33 7.26 7.06 7.06 7.05 7.05 7.05 7.04 7.03 7.03 6.99 6.98 6.97 6.96
N
O
F3C
1.00
1.02
1.06
1.04
1.00
1.01
1.02
1.01
f1 (ppm)

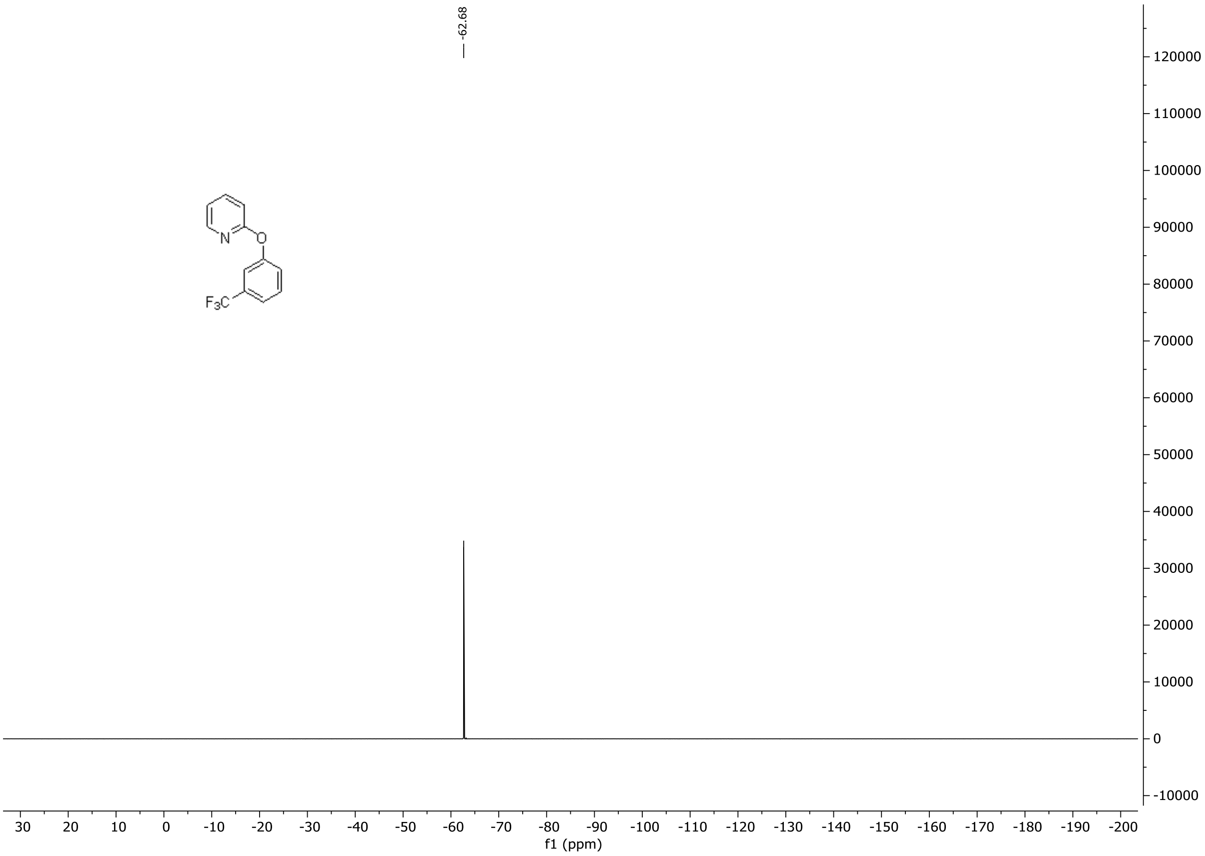
-62.68
N
O
F3C
f1 (ppm)

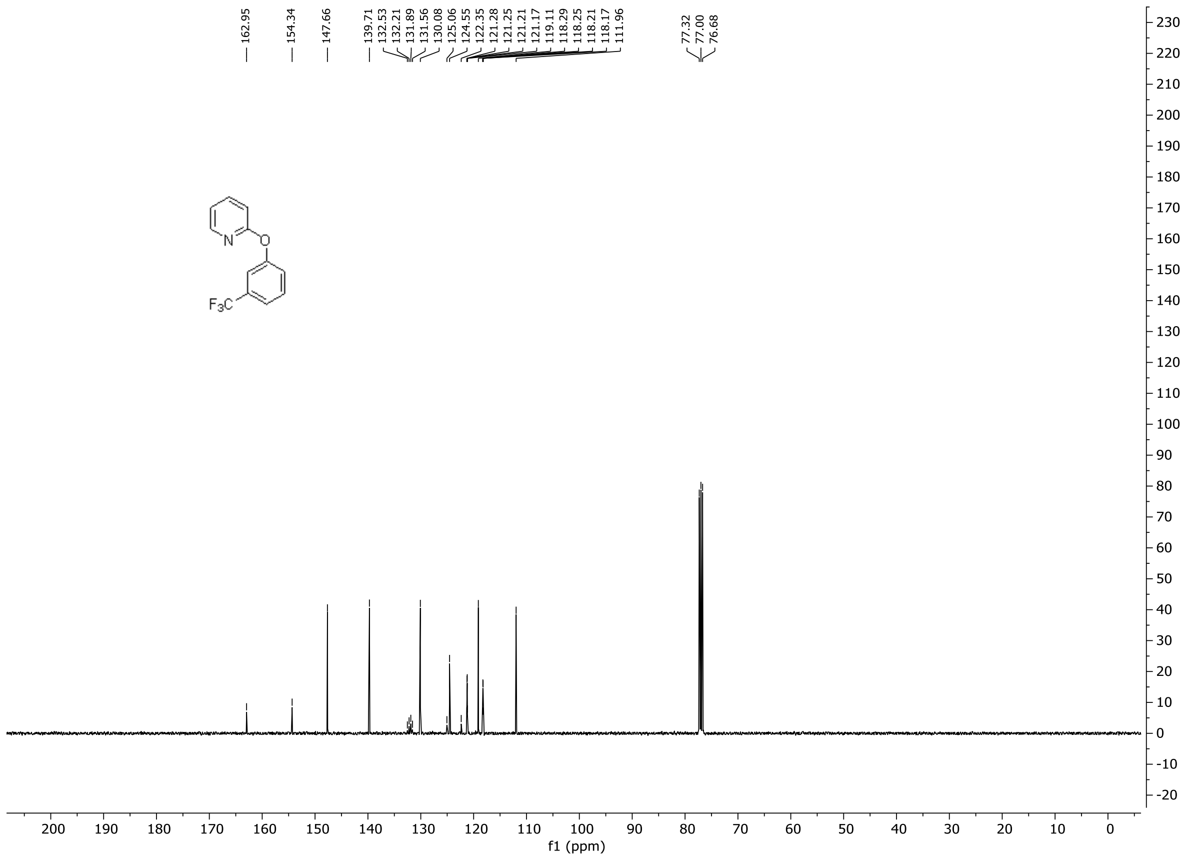
162.95
154.34
147.66
139.71
132.53
132.21
131.89
131.56
130.08
125.06
124.55
122.35
121.28
121.25
121.21
121.17
119.11
118.29
118.25
118.21
118.17
111.96
77.32
77.00
76.68
N
O
F3C
f1 (ppm)

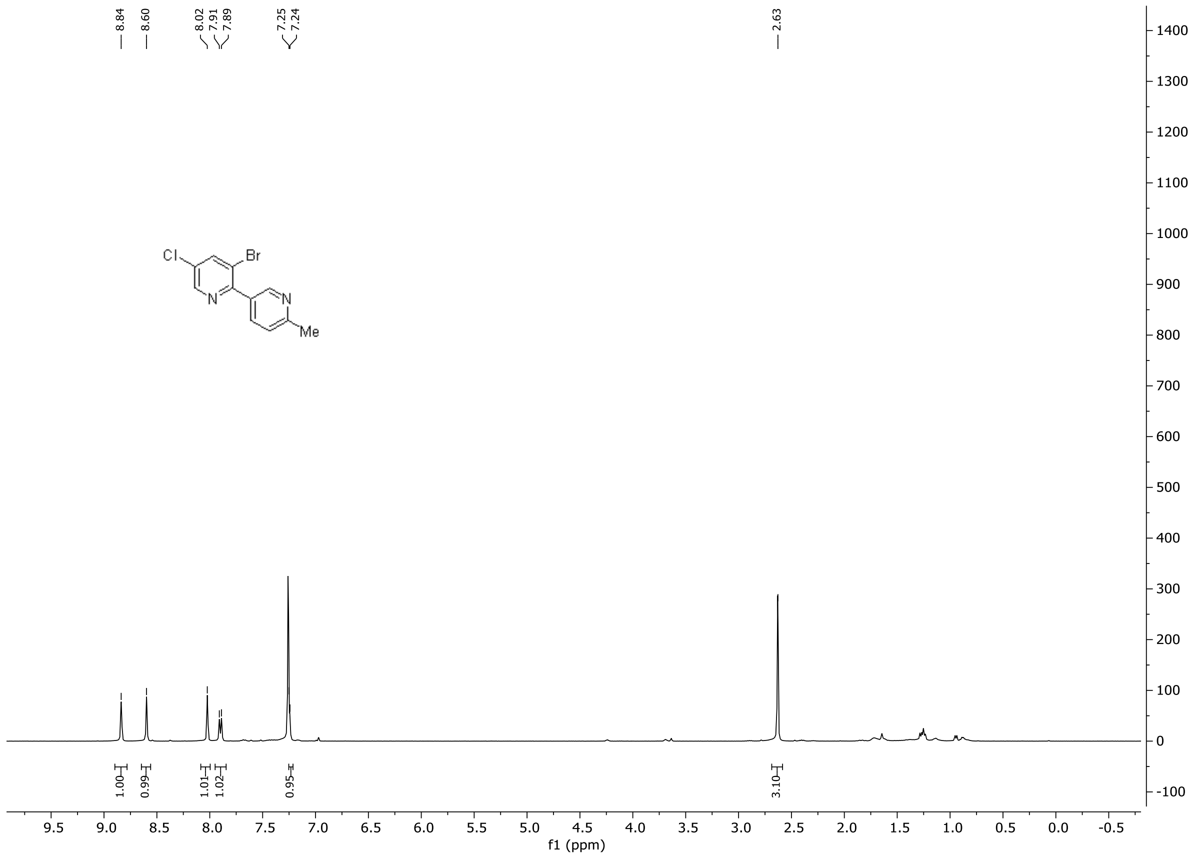
8.84
8.60
8.02
7.91
7.89
7.25
7.24
2.63
Cl
Br
N
N
Me
1.00
0.99
1.01
1.02
0.95
3.10
f1 (ppm)

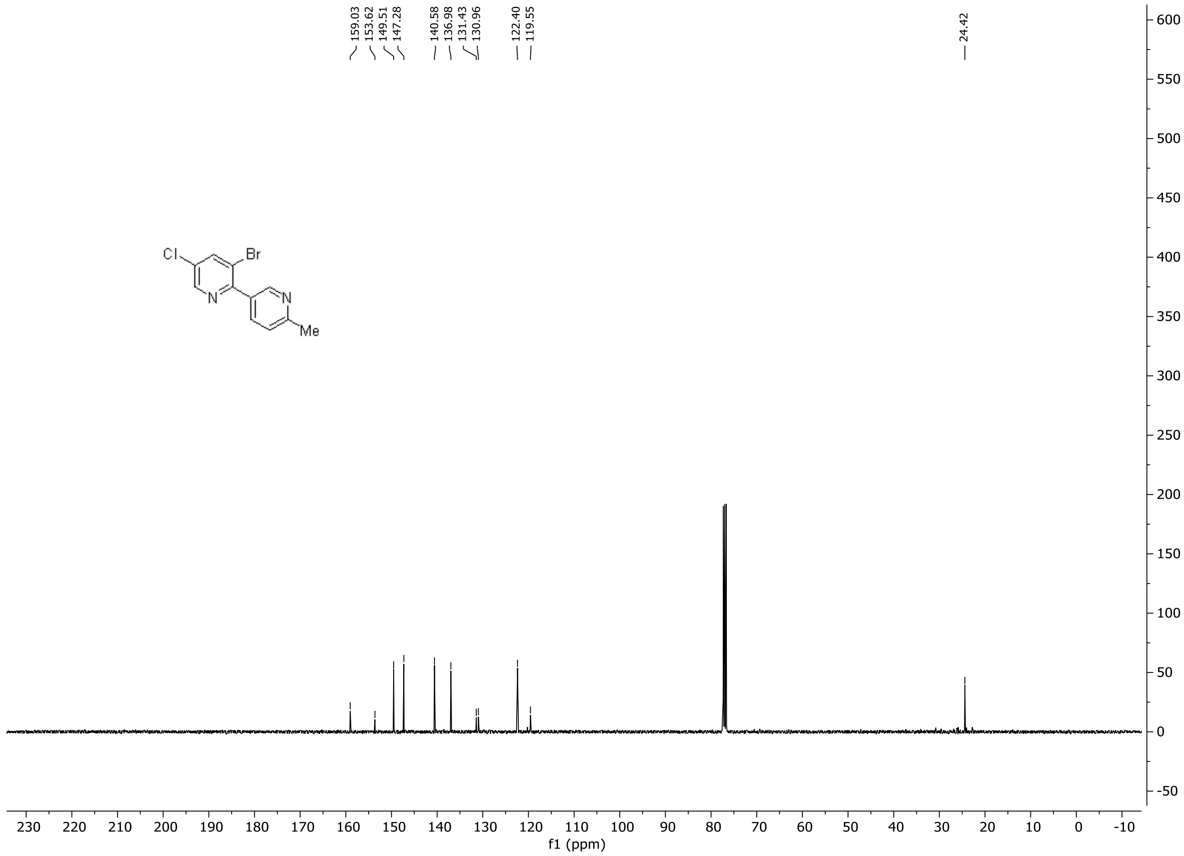
159.03
153.62
149.51
147.28
140.58
136.98
131.43
130.96
122.40
119.55
24.42
Cl
Br
N
N
Me
f1 (ppm)

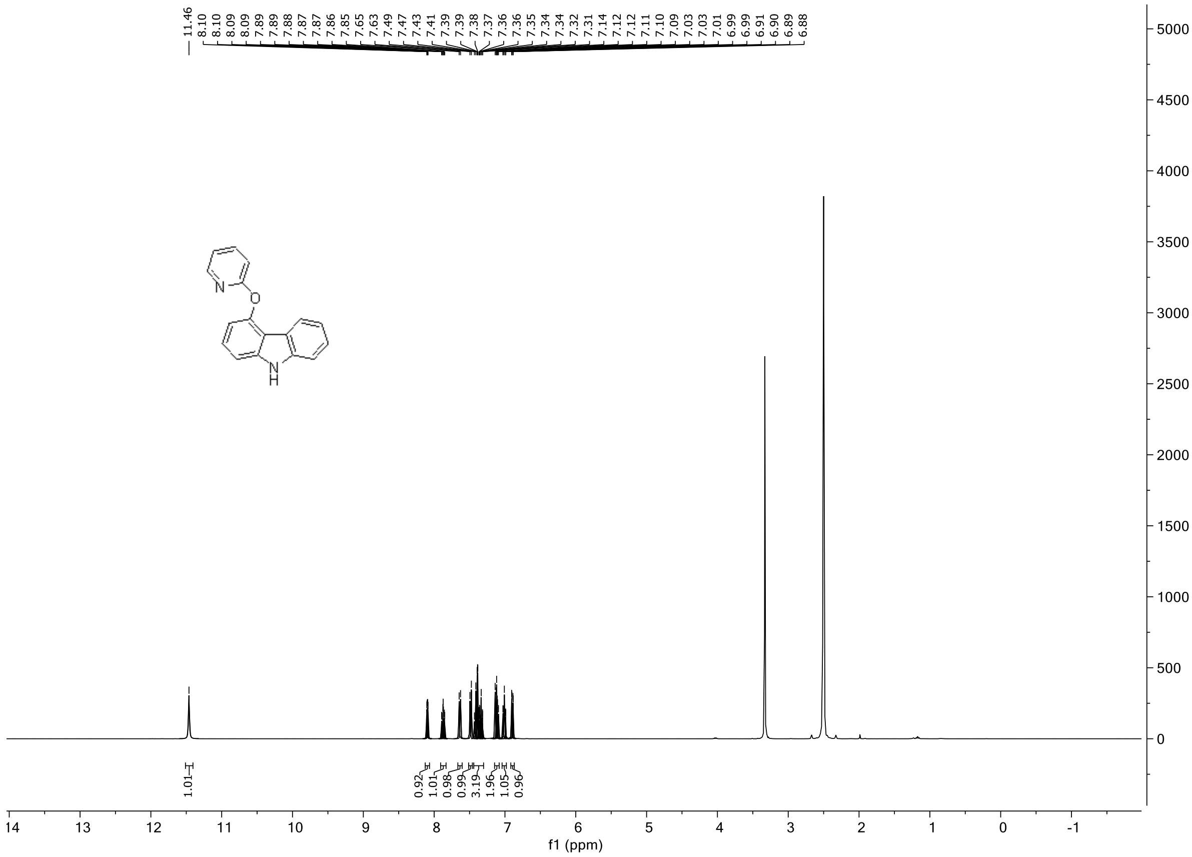
11.46
8.10
8.09
8.09
7.89
7.88
7.87
7.87
7.86
7.85
7.65
7.63
7.49
7.47
7.43
7.41
7.39
7.39
7.38
7.37
7.36
7.35
7.34
7.34
7.32
7.31
7.14
7.12
7.12
7.11
7.10
7.09
7.03
7.01
6.99
6.99
6.91
6.90
6.89
6.88
N
O
H
1.01
0.92
1.01
0.98
0.99
3.19
1.96
1.05
0.96
f1 (ppm)

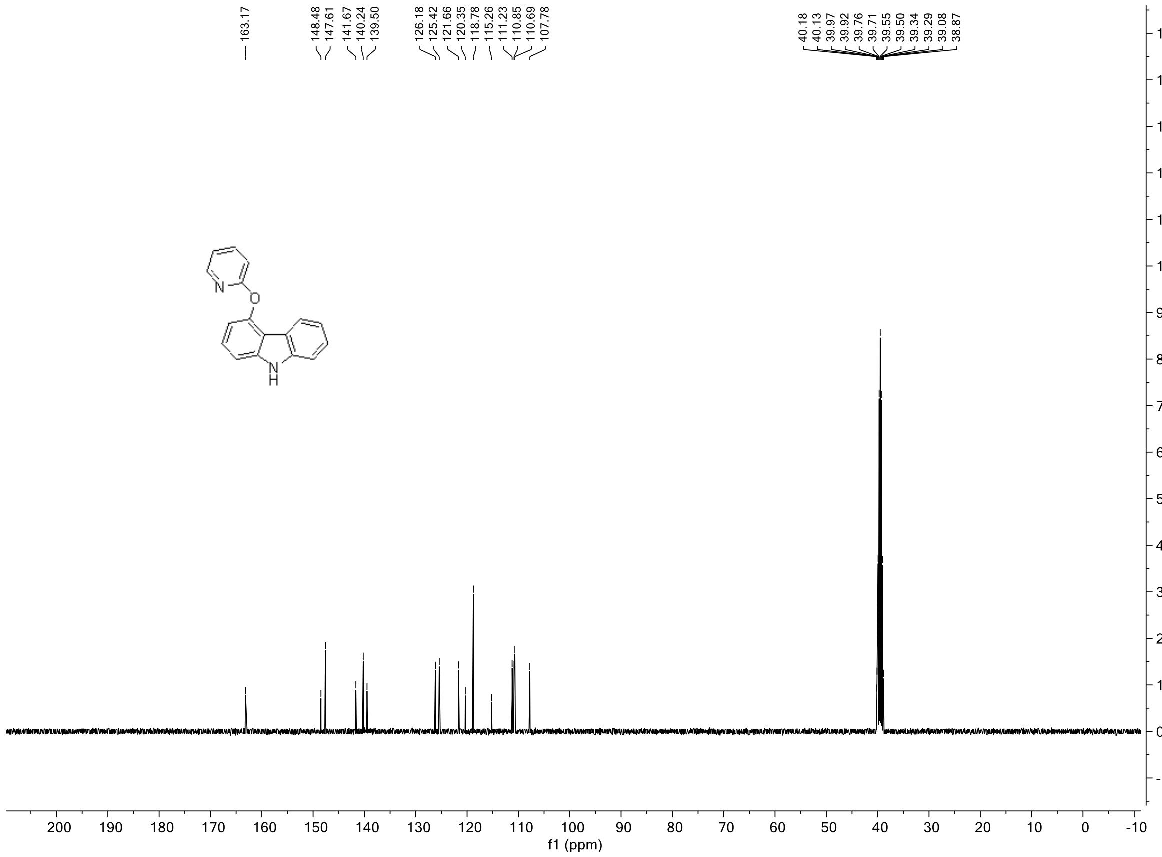
163.17
148.48
147.61
141.67
140.24
139.50
126.18
125.42
121.66
120.35
118.78
115.26
111.23
110.85
110.69
107.78
40.18
40.13
39.97
39.92
39.76
39.71
39.55
39.50
39.34
39.29
39.08
38.87
N
O
H
f1 (ppm)

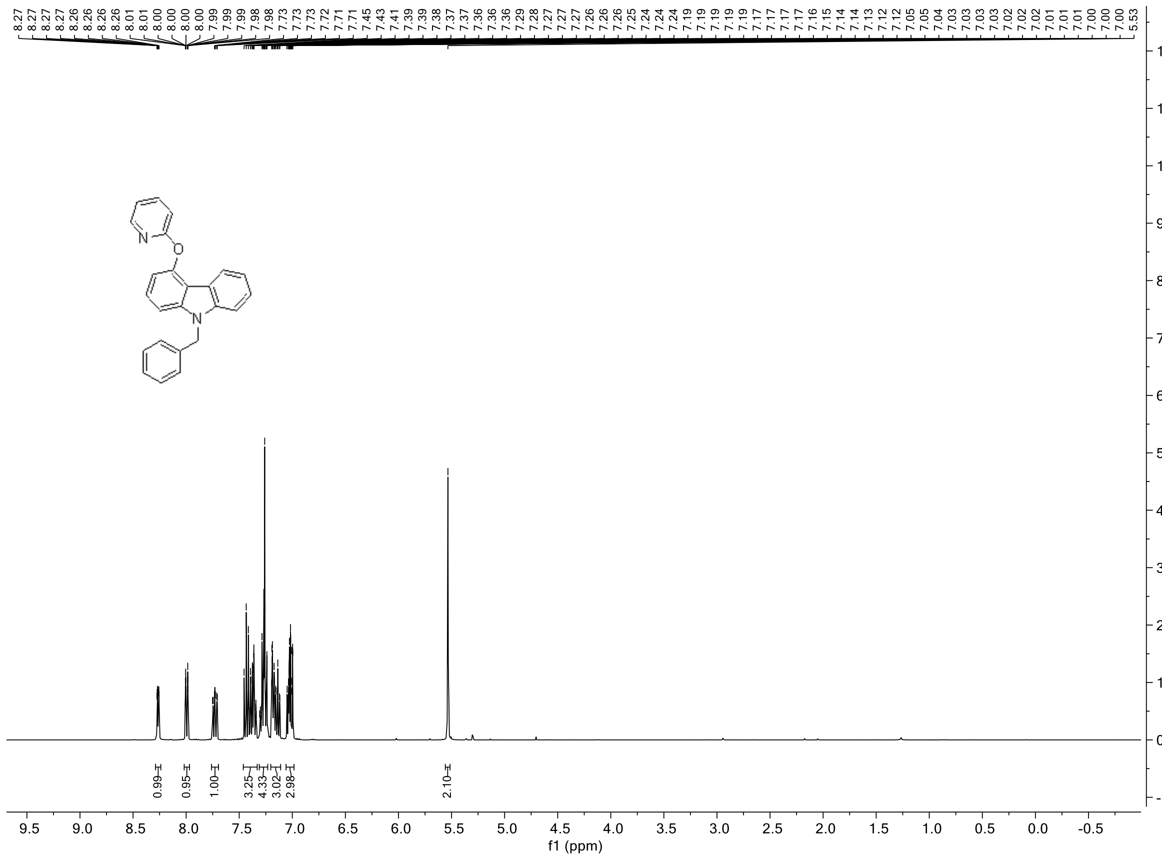
8.27 8.27 8.27 8.26 8.26 8.26 8.26 8.01 8.01 8.00 8.00 8.00 8.00 7.99 7.99 7.99 7.98 7.98 7.73 7.73 7.72 7.71 7.71 7.45 7.43 7.41 7.39 7.38 7.37 7.37 7.36 7.36 7.29 7.28 7.27 7.27 7.26 7.26 7.25 7.24 7.24 7.24 7.19 7.19 7.19 7.19 7.17 7.17 7.17 7.16 7.15 7.14 7.13 7.12 7.12 7.05 7.04 7.03 7.03 7.03 7.03 7.02 7.02 7.01 7.01 7.01 7.00 7.00 5.53
N
O
N
0.99
0.95
1.00
3.25
4.33
3.02
2.98
2.10
f1 (ppm)

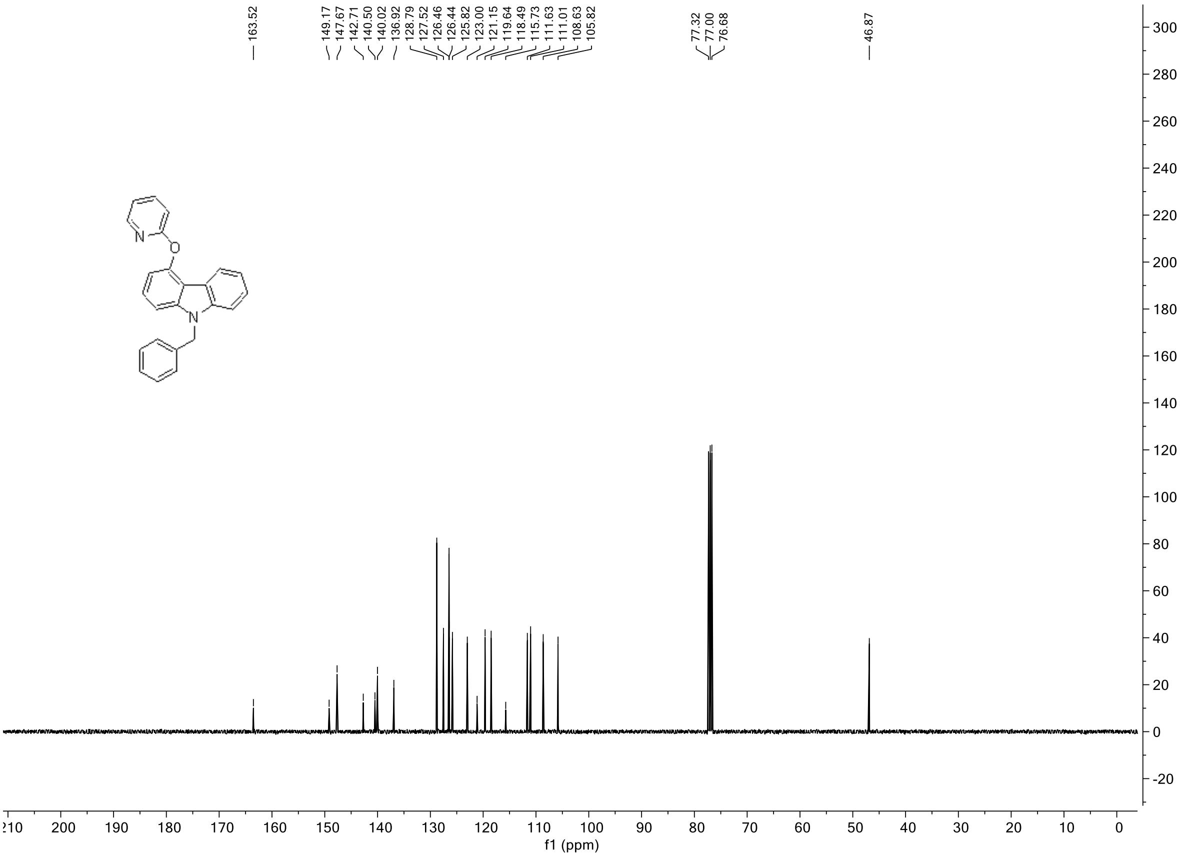

163.52
149.17
147.67
142.71
140.50
140.02
136.92
128.79
127.52
126.46
126.44
125.82
123.00
121.15
119.64
118.49
115.73
111.63
111.01
108.63
105.82
77.32
77.00
76.68
46.87
N
O
N
f1 (ppm)


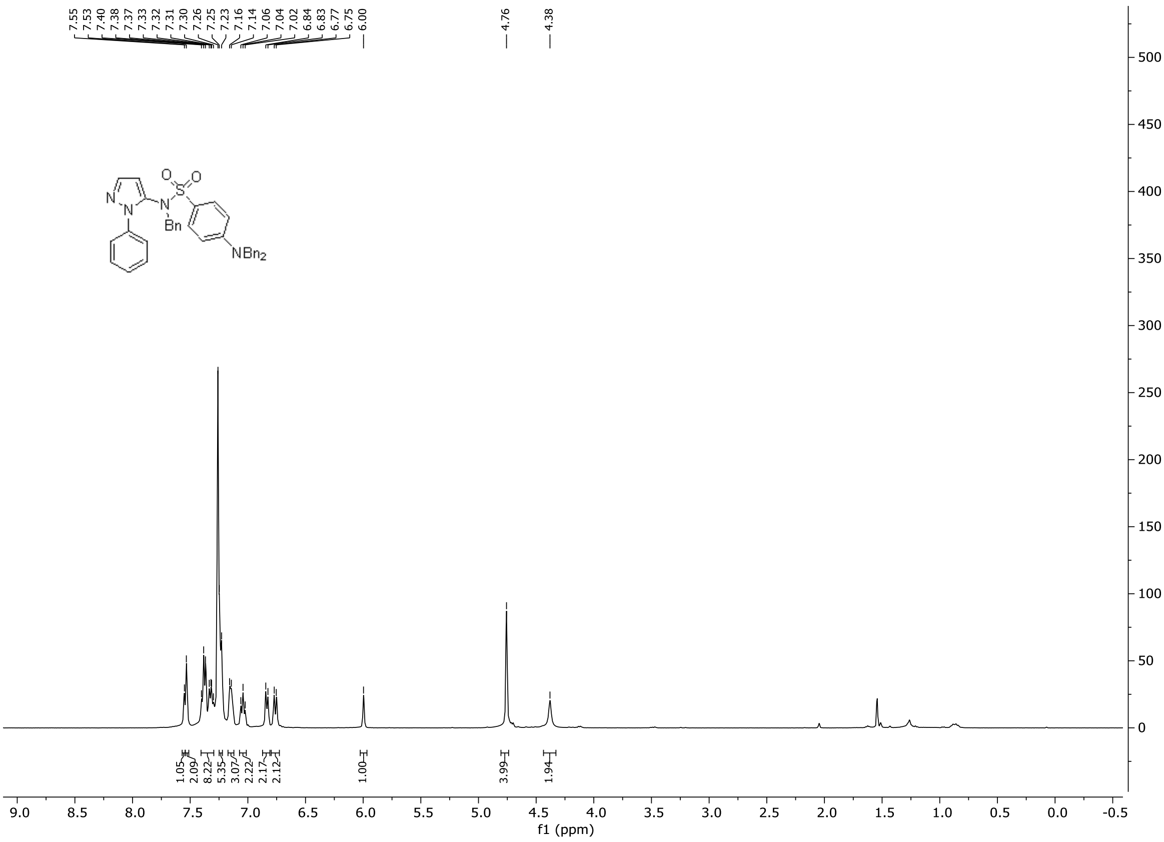

7.55
7.53
7.40
7.38
7.37
7.33
7.32
7.31
7.30
7.26
7.25
7.23
7.16
7.14
7.06
7.04
7.02
6.84
6.83
6.77
6.75
6.00
4.76
4.38
N
N
N
Bn
O
S
O
NBn2
1.05
2.09
8.22
5.35
3.07
2.22
2.17
2.12
1.00
3.99
1.94
f1 (ppm)

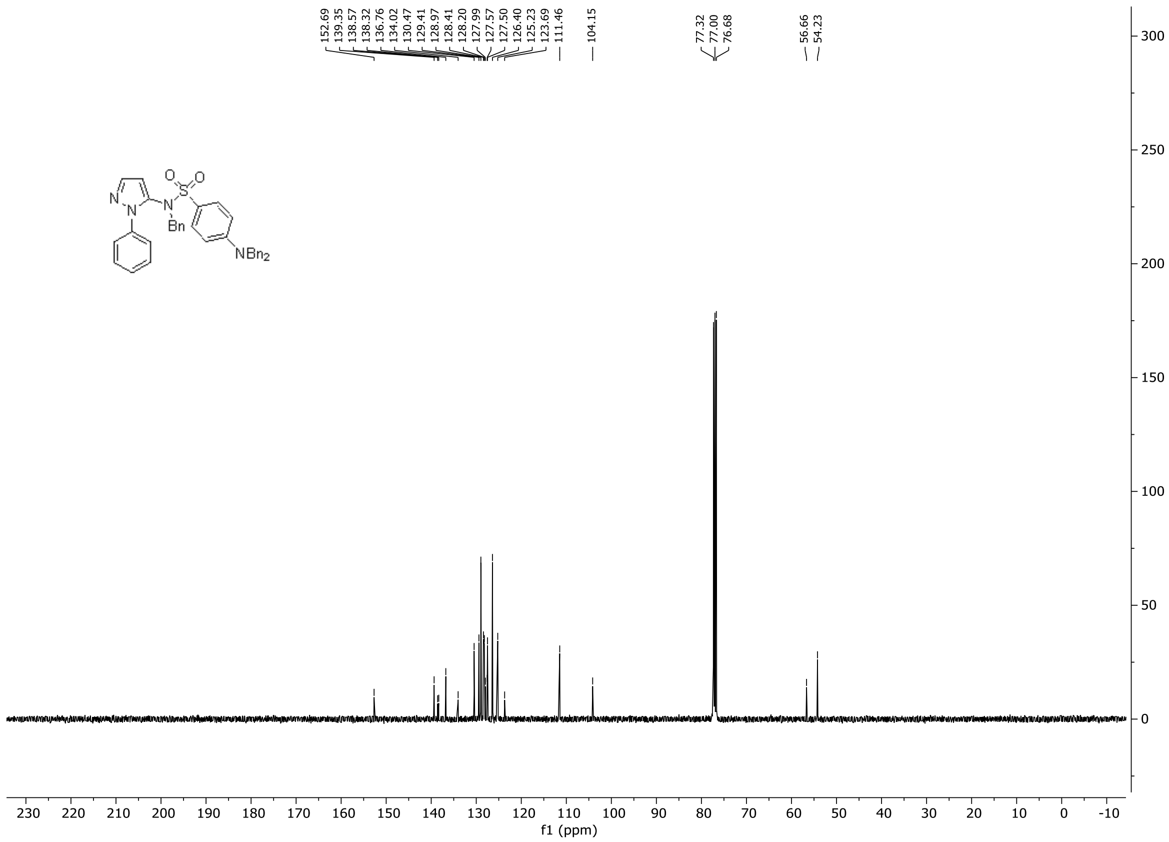
152.69
139.35
138.57
138.32
136.76
134.02
130.47
129.41
128.97
128.41
128.20
127.99
127.57
127.50
126.40
125.23
123.69
111.46
104.15
77.32
77.00
76.68
56.66
54.23
Bn
NBn2
f1 (ppm)

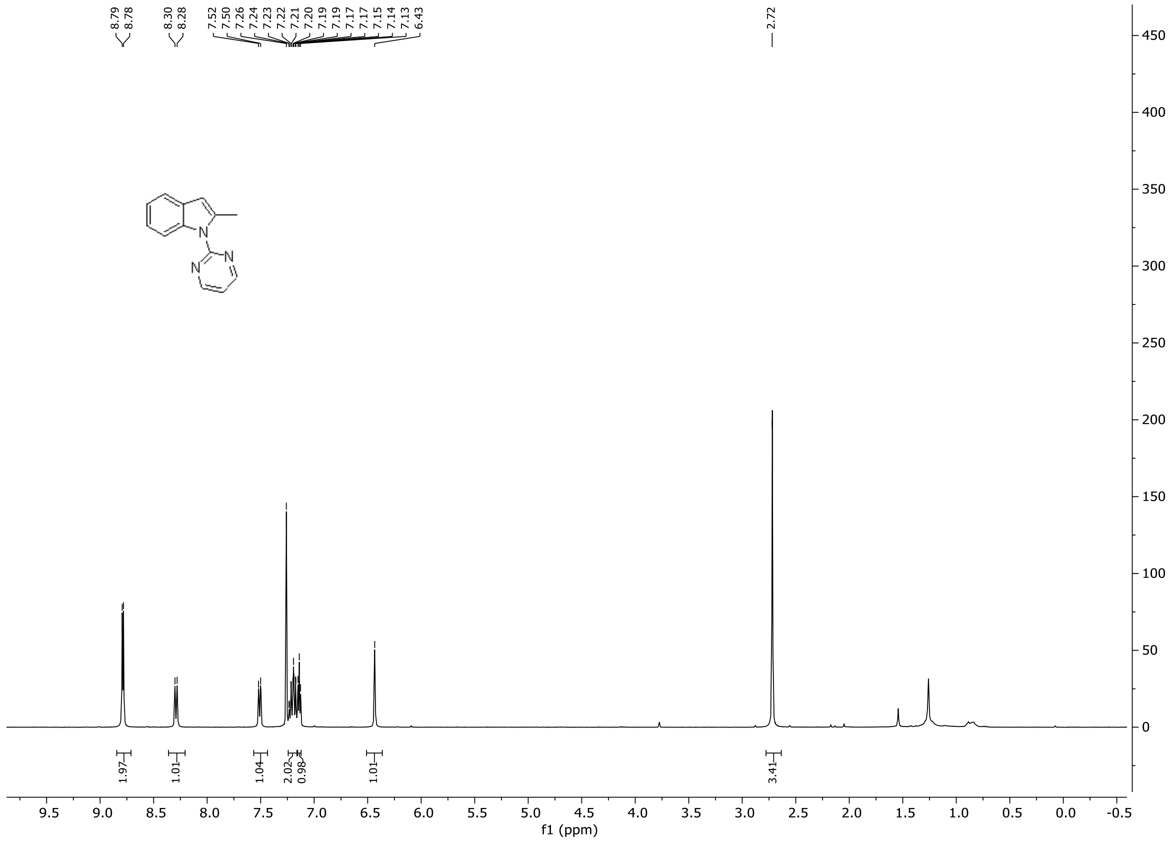
8.79
8.78
8.30
8.28
7.52
7.50
7.26
7.24
7.23
7.22
7.21
7.20
7.19
7.19
7.17
7.15
7.14
7.13
6.43
2.72
1.97
1.01
1.04
2.02
0.98
1.01
3.41
f1 (ppm)

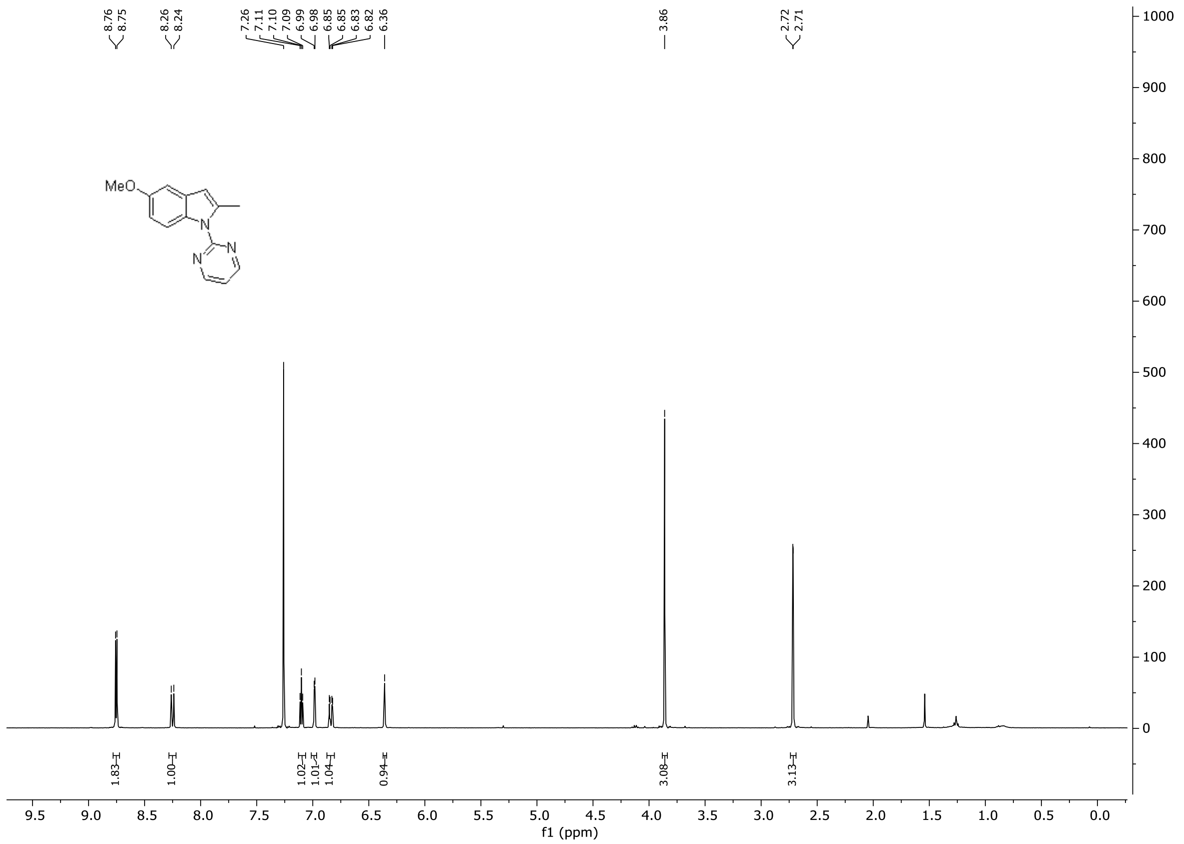
8.76
8.75
8.26
8.24
7.26
7.11
7.10
7.09
6.99
6.98
6.85
6.85
6.83
6.82
6.36
3.86
2.72
2.71
MeO
N
N
N
1.83
1.00
1.02
1.01
1.04
0.94
3.08
3.13
f1 (ppm)

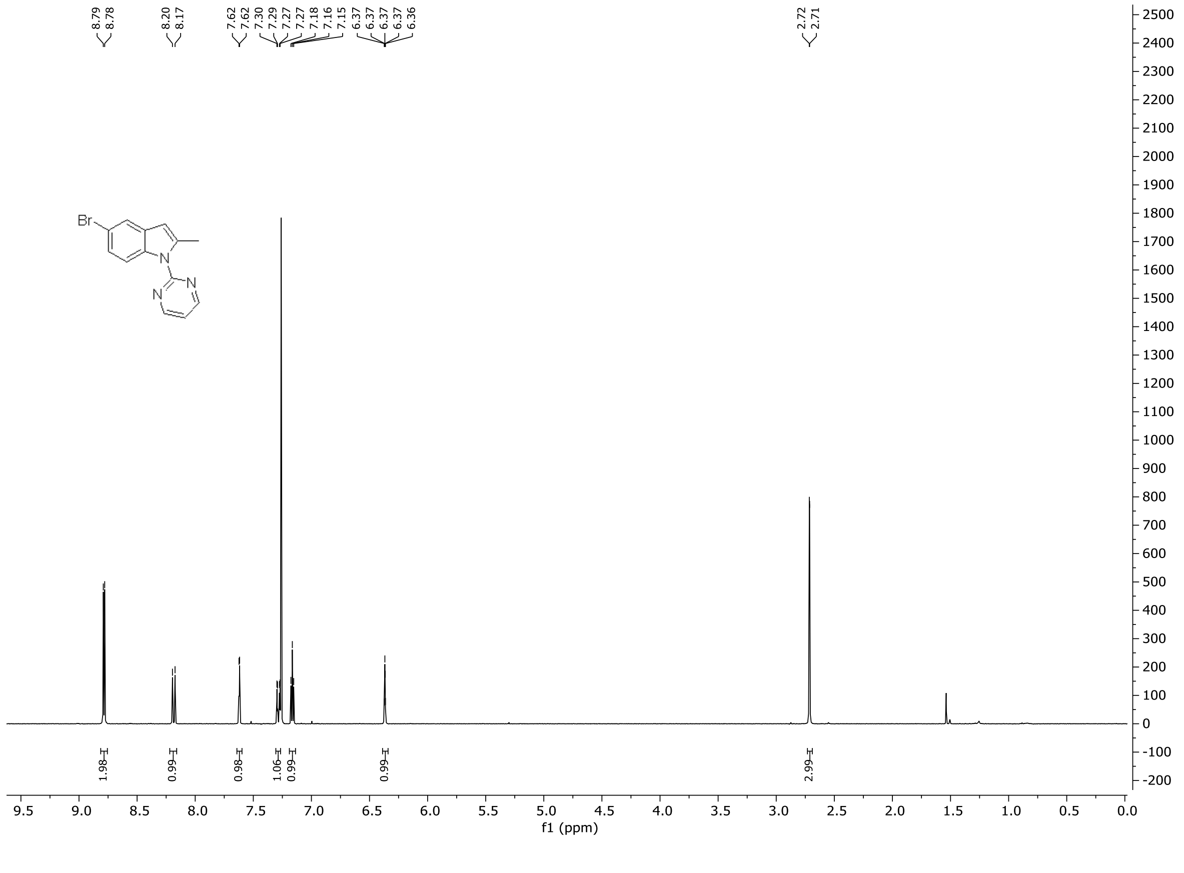
8.79
8.78
8.20
8.17
7.62
7.62
7.30
7.29
7.27
7.27
7.18
7.16
7.15
6.37
6.37
6.37
6.37
6.36
2.72
2.71
Br
N
N
N
1.98
0.99
0.98
1.06
0.99
0.99
2.99
f1 (ppm)

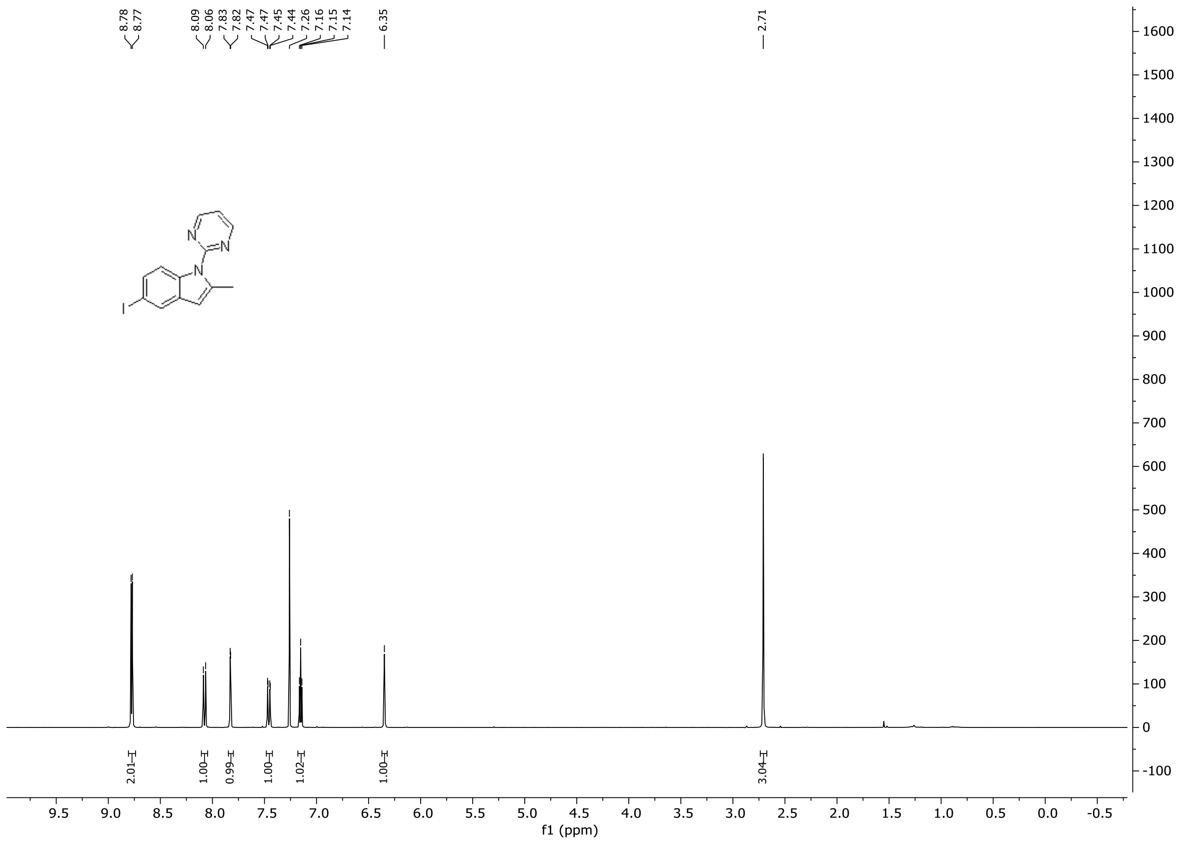
8.78
8.77
8.09
8.06
7.83
7.82
7.47
7.45
7.44
7.26
7.16
7.15
7.14
6.35
2.71
2.01
1.00
0.99
1.00
1.02
1.00
3.04
N
N
N
I
f1 (ppm)

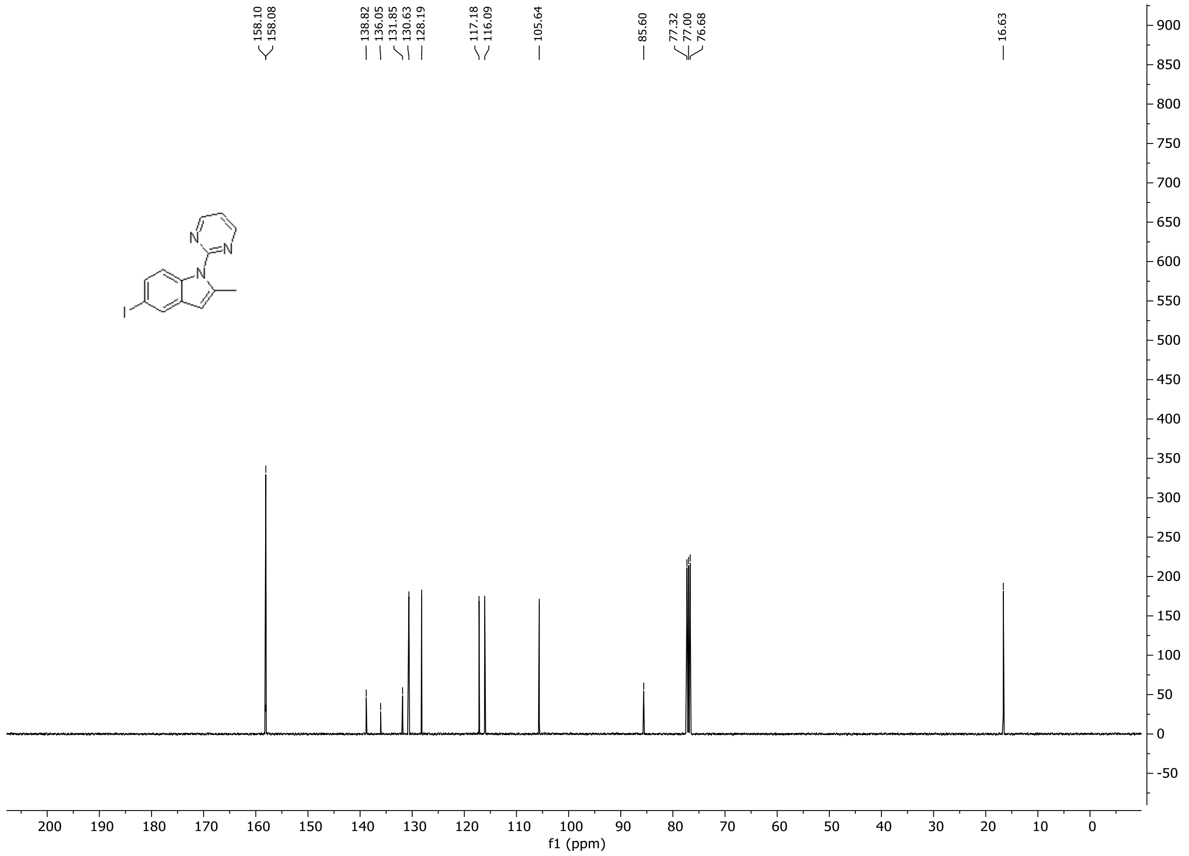
158.10
158.08
138.82
136.05
131.85
130.63
128.19
117.18
116.09
105.64
85.60
77.32
77.00
76.68
16.63
N
N
N
I
f1 (ppm)

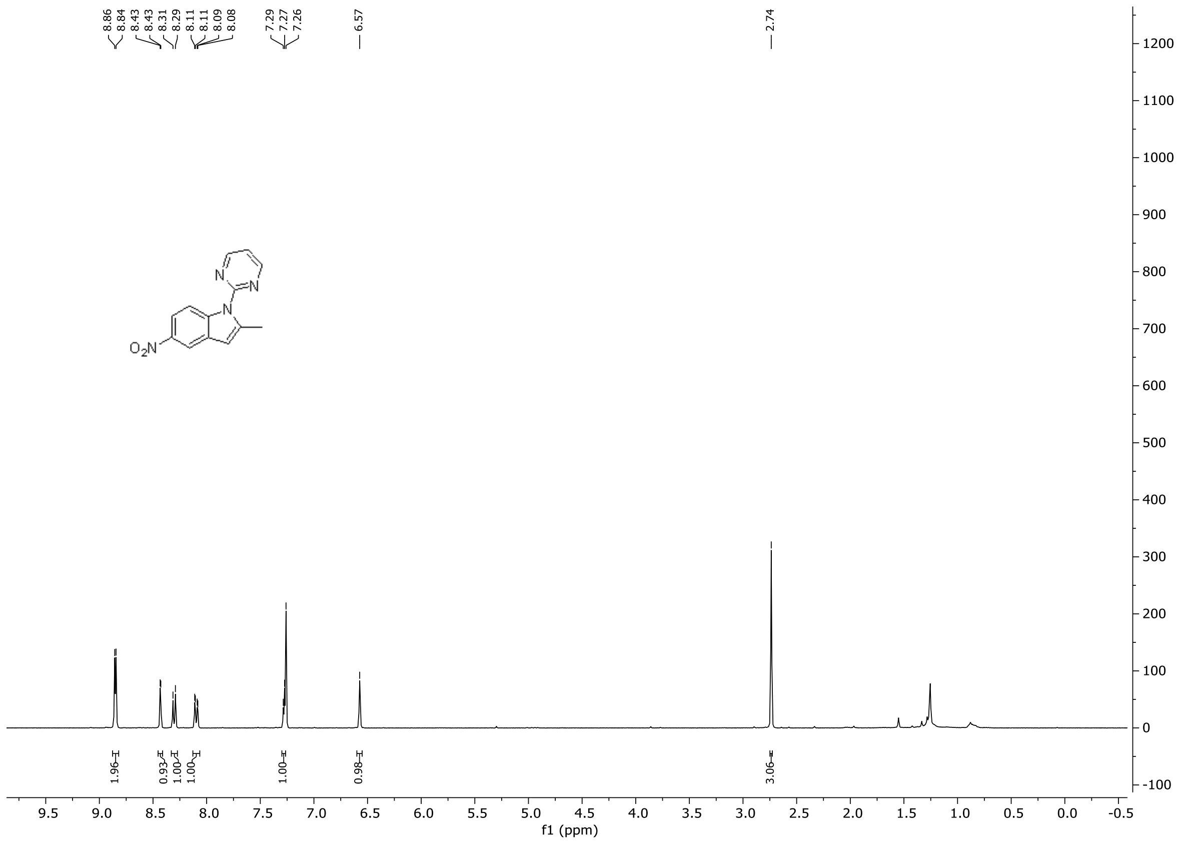
8.86 8.84 8.43 8.31 8.29 8.11 8.11 8.09 8.08
7.29 7.27 7.26
6.57
2.74
1.96 0.93 1.00 1.00 1.00 0.98 3.06
f1 (ppm)
N
N
N
O2N

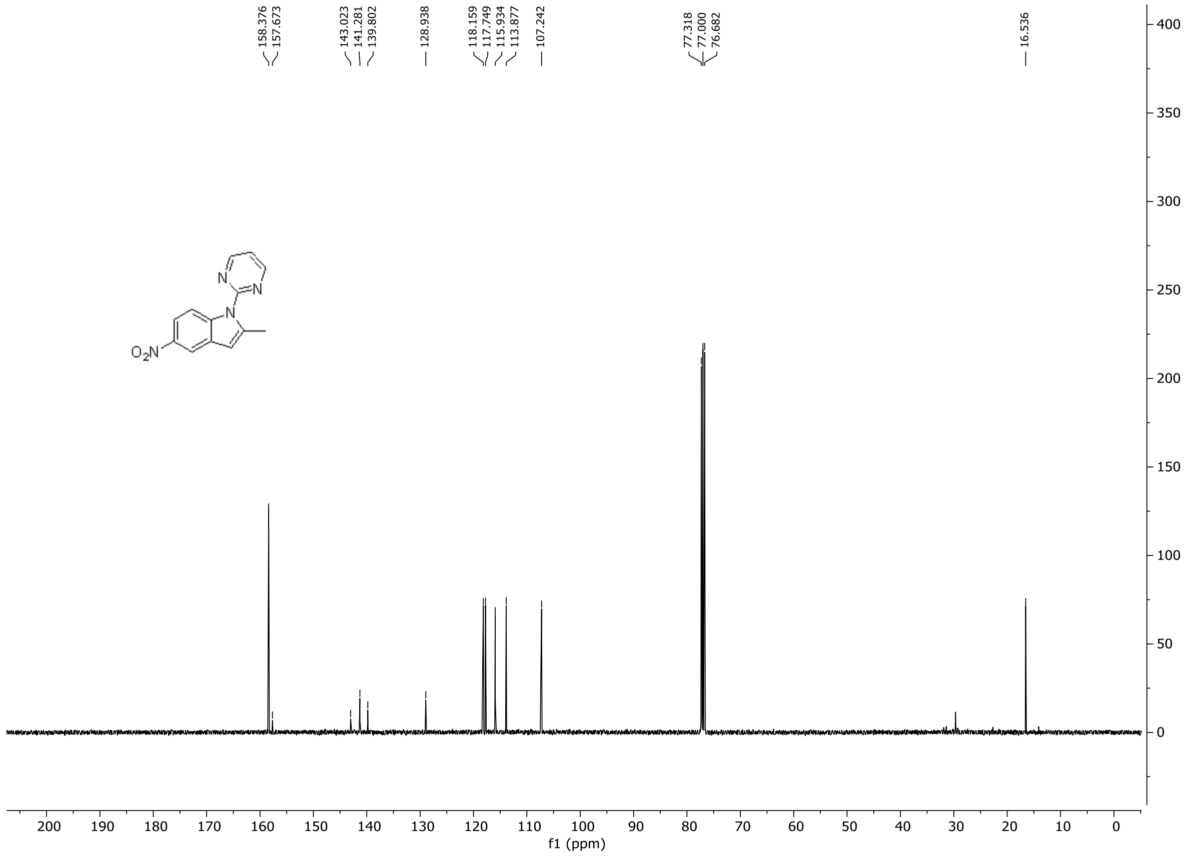
158.376 157.673
143.023 141.281 139.802
128.938
118.159 117.749 115.934 113.877
107.242
77.318 77.000 76.682
16.536
f1 (ppm)
N
N
N
O2N

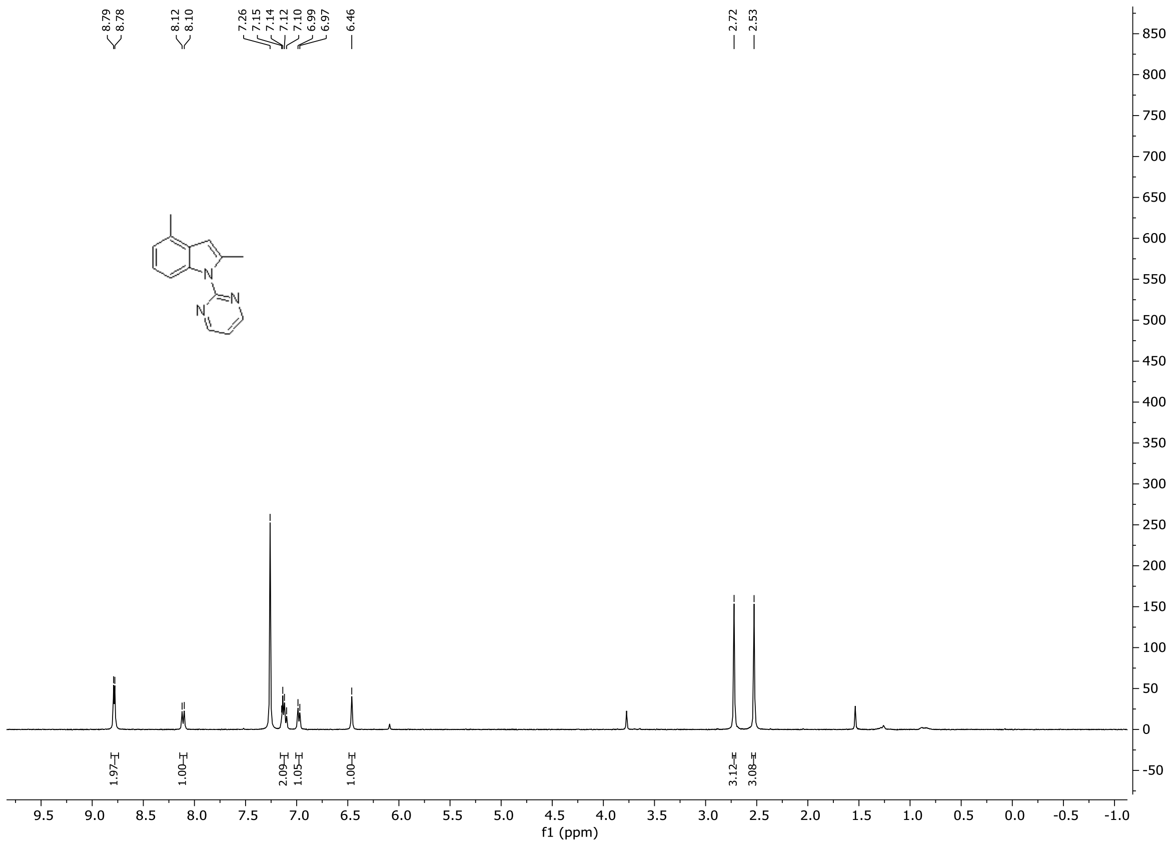

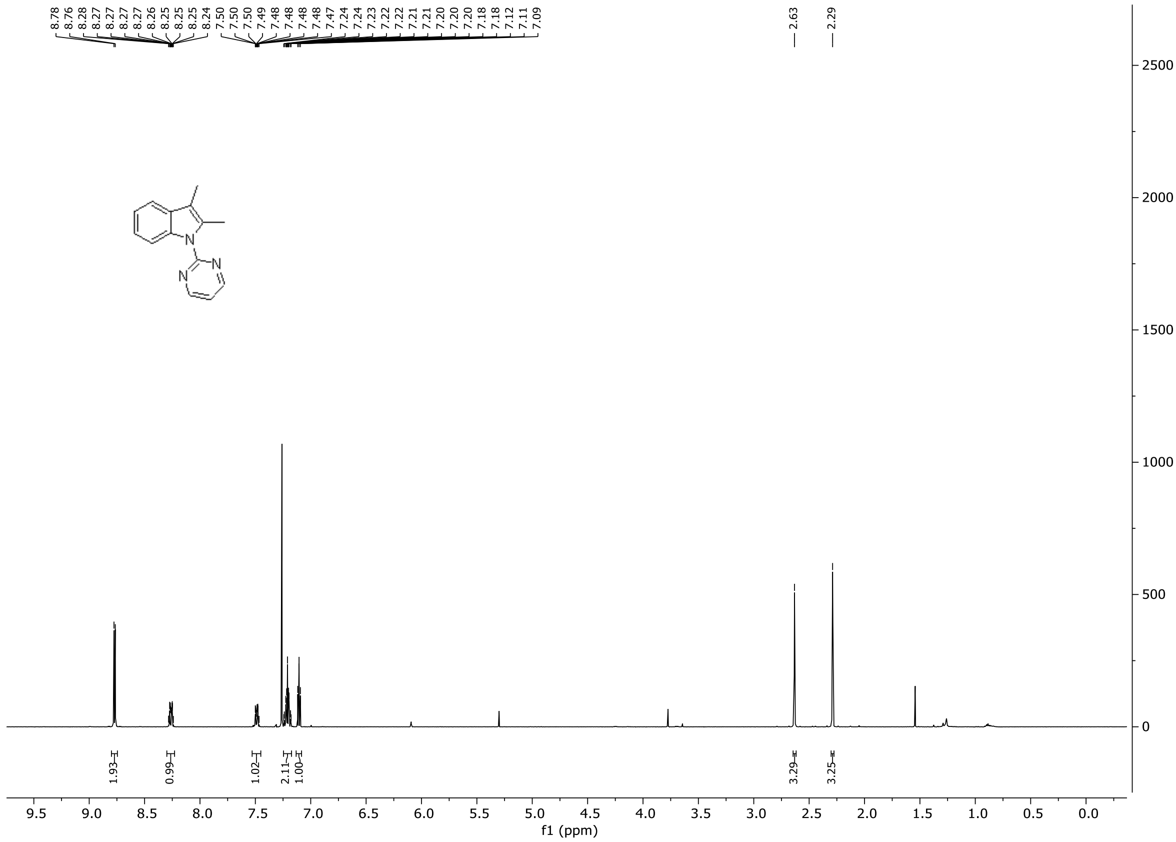

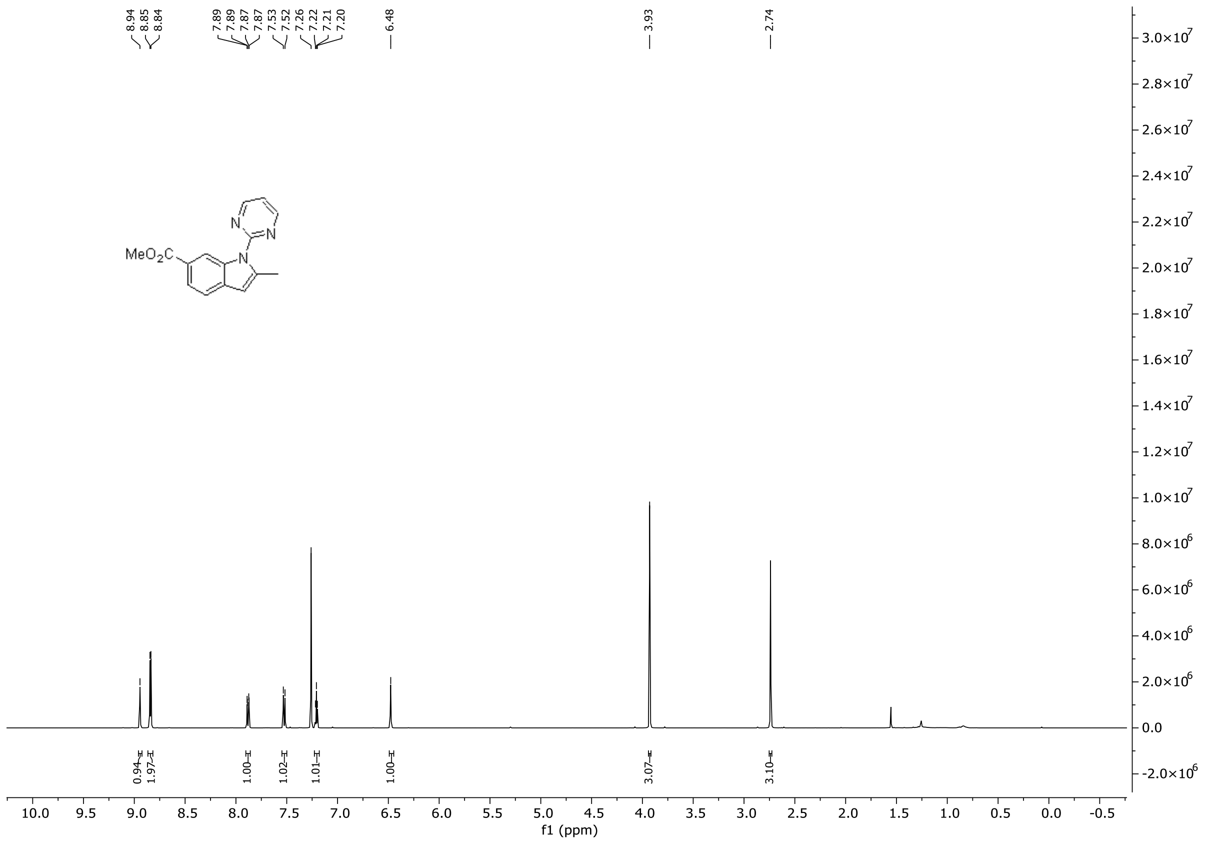
8.94
8.85
8.84
7.89
7.89
7.87
7.87
7.53
7.52
7.26
7.22
7.21
7.20
6.48
3.93
2.74
MeO2C
N
N
N
0.94
1.97
1.00
1.02
1.01
1.00
3.07
3.10
f1 (ppm)

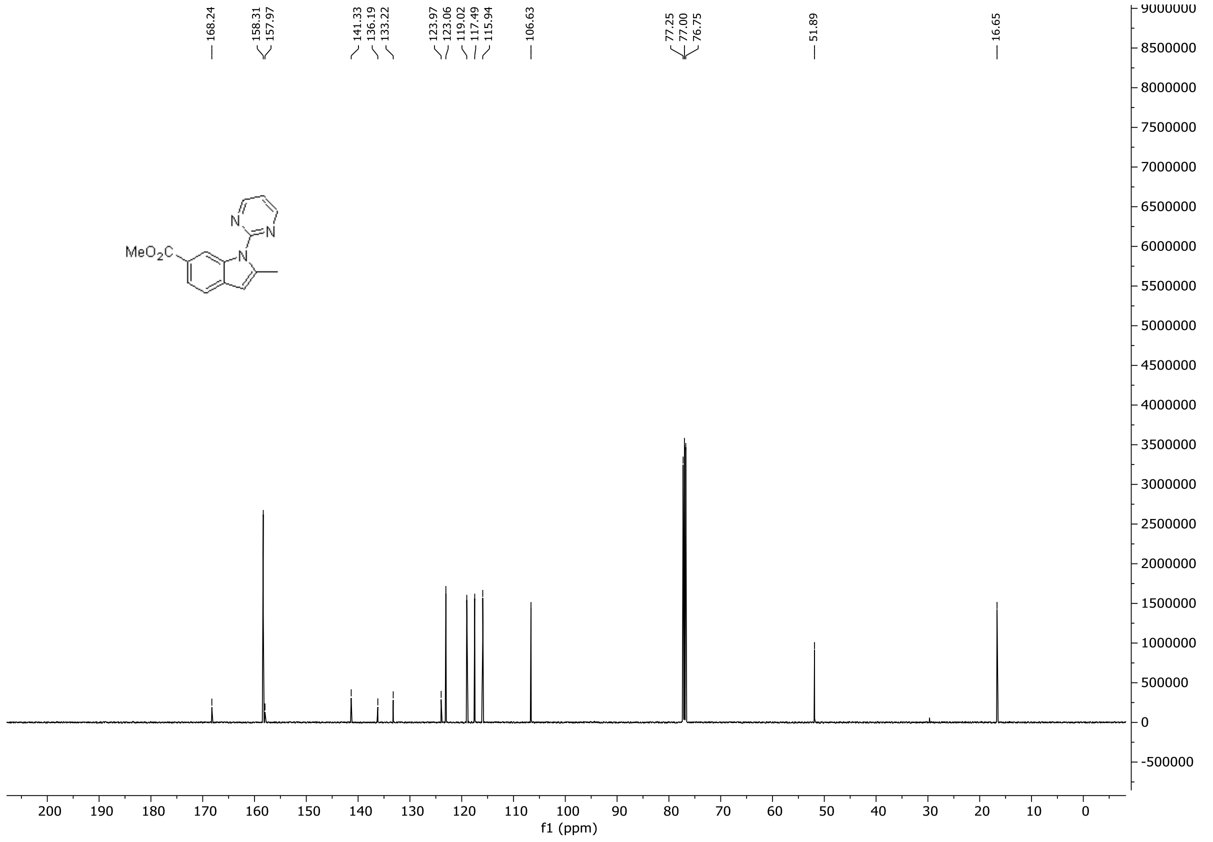
168.24
158.31
157.97
141.33
136.19
133.22
123.97
123.06
119.02
117.49
115.94
106.63
77.25
77.00
76.75
51.89
16.65
MeO2C
N
N
N
f1 (ppm)

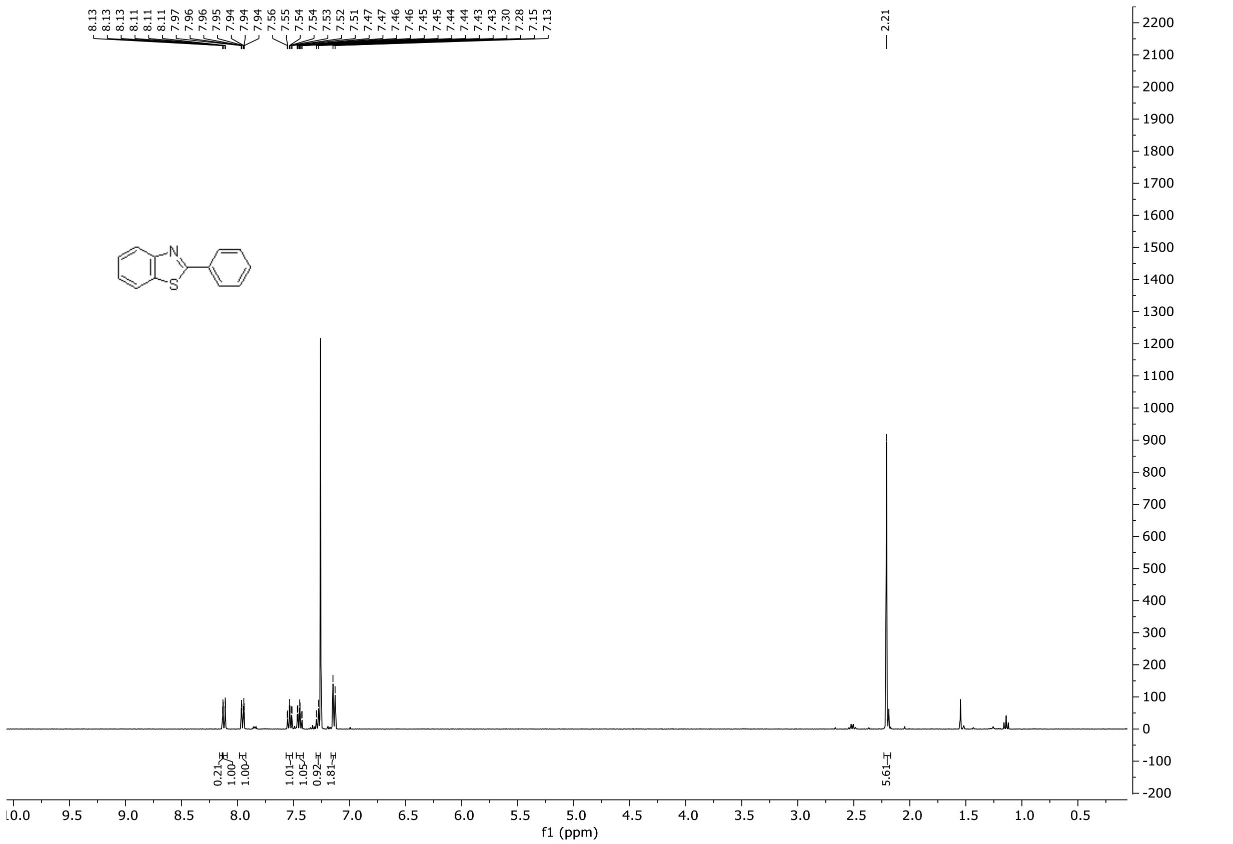
8.13 8.13 8.13 8.11 8.11 8.11 7.97 7.96 7.96 7.95 7.94 7.94 7.94 7.56 7.55 7.54 7.54 7.53 7.52 7.51 7.47 7.47 7.46 7.46 7.45 7.45 7.44 7.44 7.43 7.43 7.30 7.28 7.15 7.13
2.21
N
S
0.21 1.00 1.00 1.01 1.05 0.92 1.81
5.61
f1 (ppm)

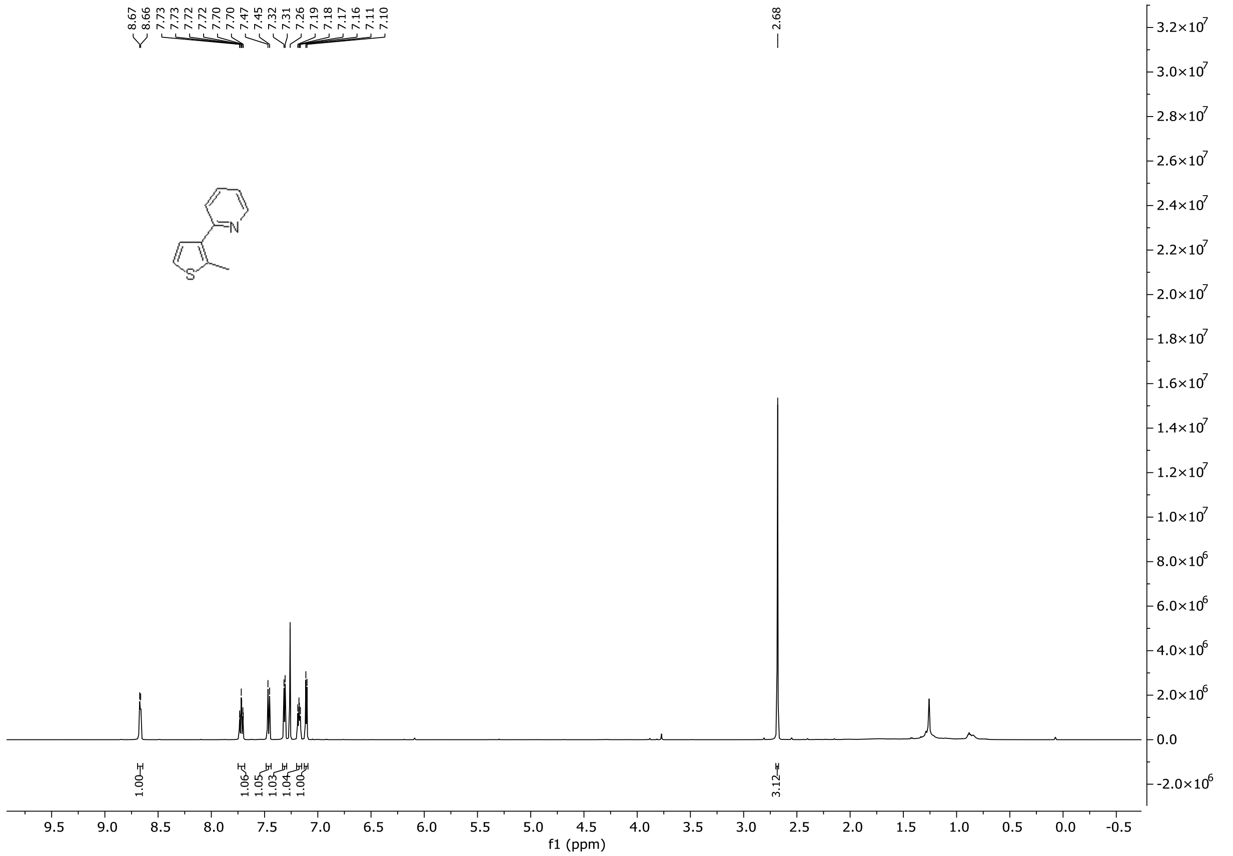
8.67 8.66 7.73 7.72 7.72 7.70 7.70 7.47 7.45 7.32 7.31 7.26 7.19 7.18 7.17 7.16 7.11 7.10
2.68
N
S
1.00 1.06 1.05 1.03 1.04 1.00
3.12
f1 (ppm)

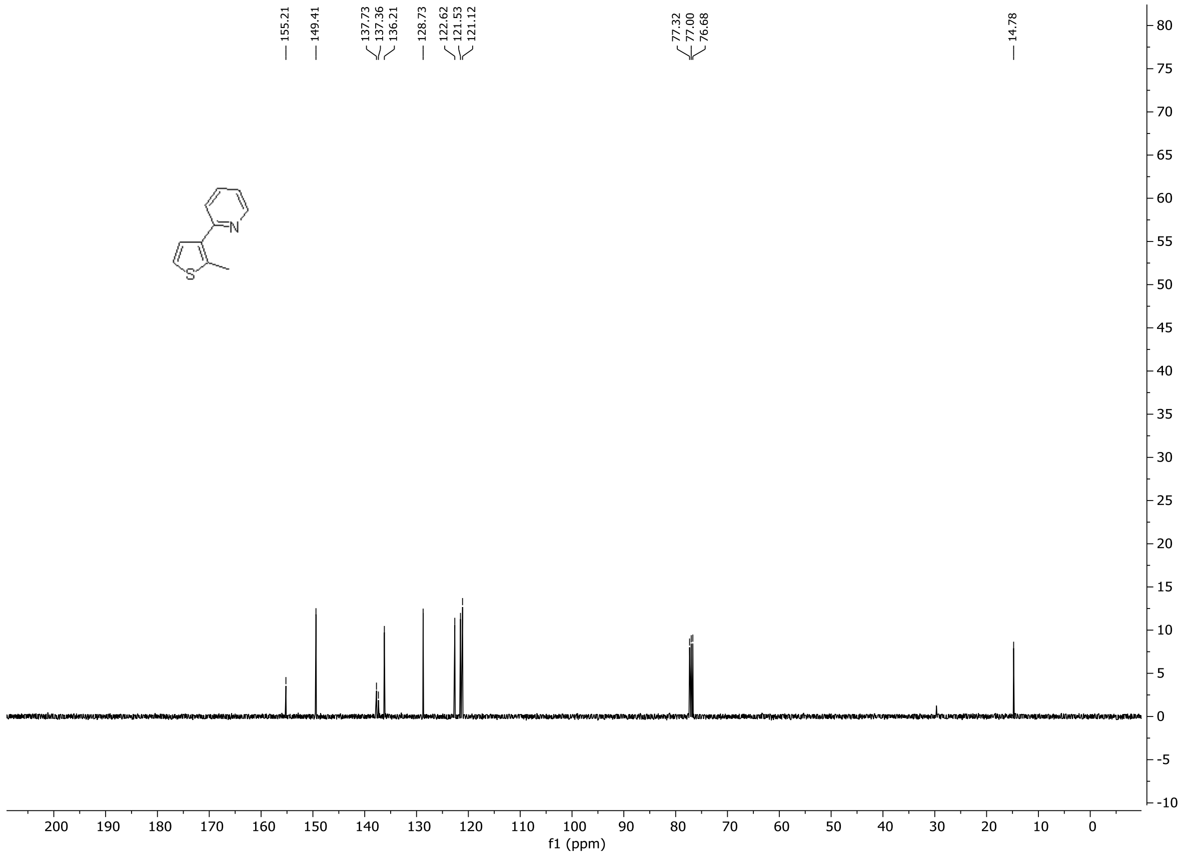
155.21
149.41
137.73
137.36
136.21
128.73
122.62
121.53
121.12
77.32
77.00
76.68
14.78
N
S
f1 (ppm)

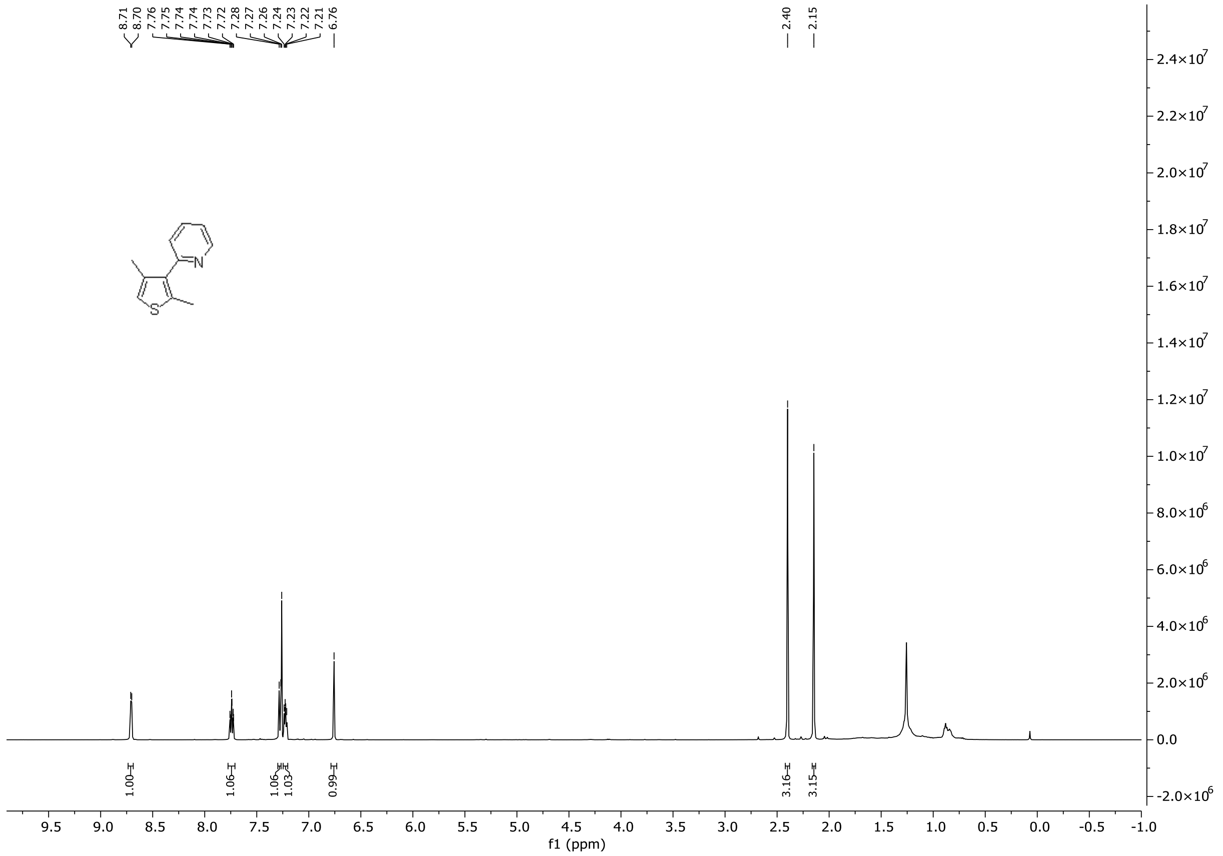
8.71
8.70
7.76
7.75
7.74
7.74
7.73
7.72
7.28
7.27
7.26
7.24
7.23
7.22
7.21
6.76
2.40
2.15
N
S
1.00
1.06
1.06
1.03
0.99
3.16
3.15
f1 (ppm)

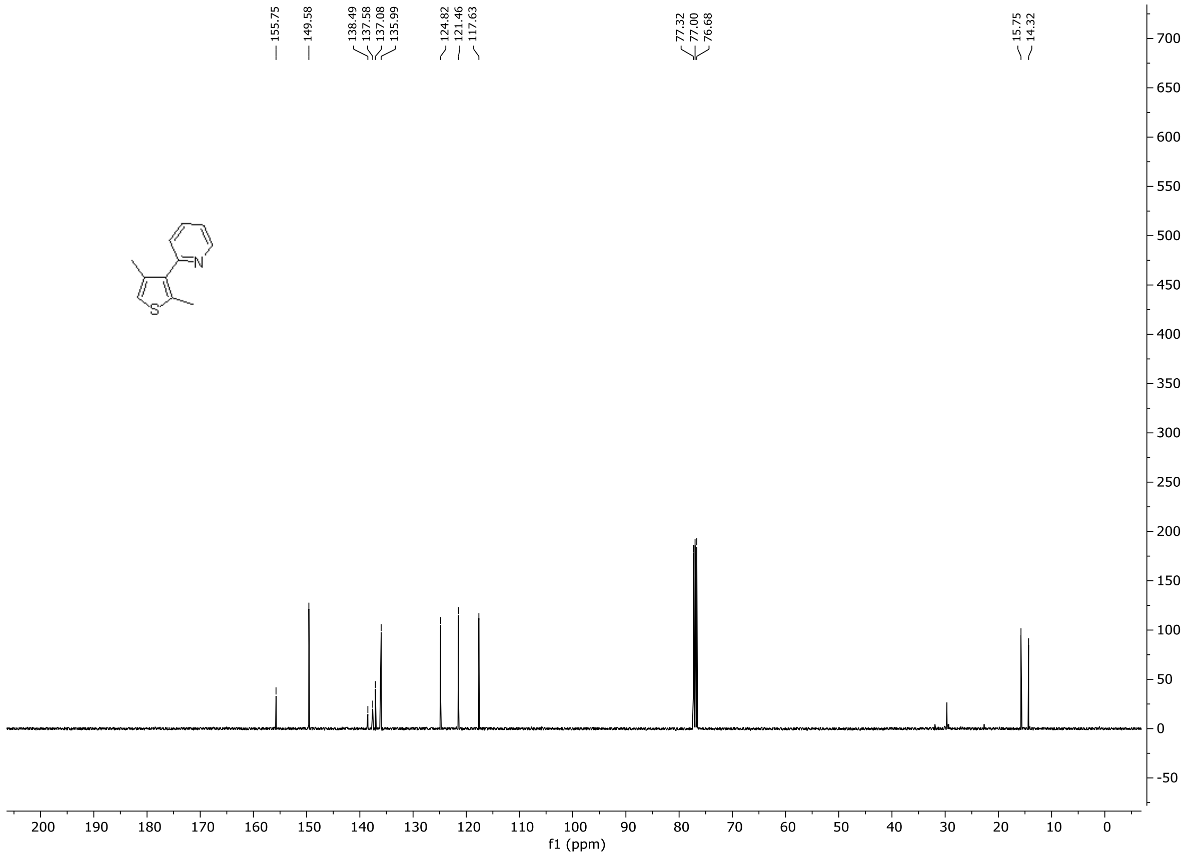
155.75
149.58
138.49
137.58
137.08
135.99
124.82
121.46
117.63
77.32
77.00
76.68
15.75
14.32
N
S
f1 (ppm)

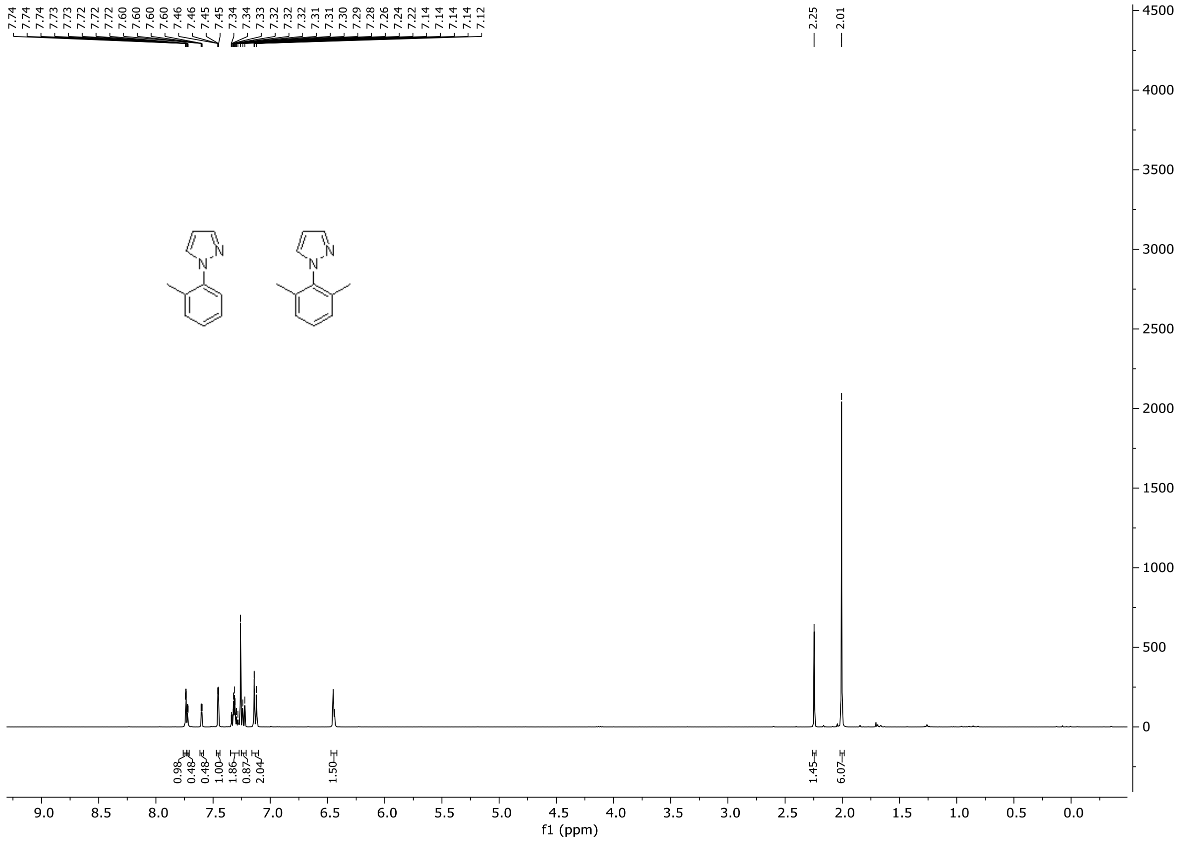
7.74
7.74
7.74
7.73
7.73
7.72
7.72
7.60
7.60
7.60
7.46
7.46
7.45
7.45
7.34
7.34
7.33
7.32
7.32
7.31
7.30
7.29
7.28
7.26
7.24
7.22
7.14
7.14
7.14
7.12
2.25
2.01
N
N
0.98
0.48
0.48
1.00
1.86
0.87
2.04
1.50
1.45
6.07
f1 (ppm)

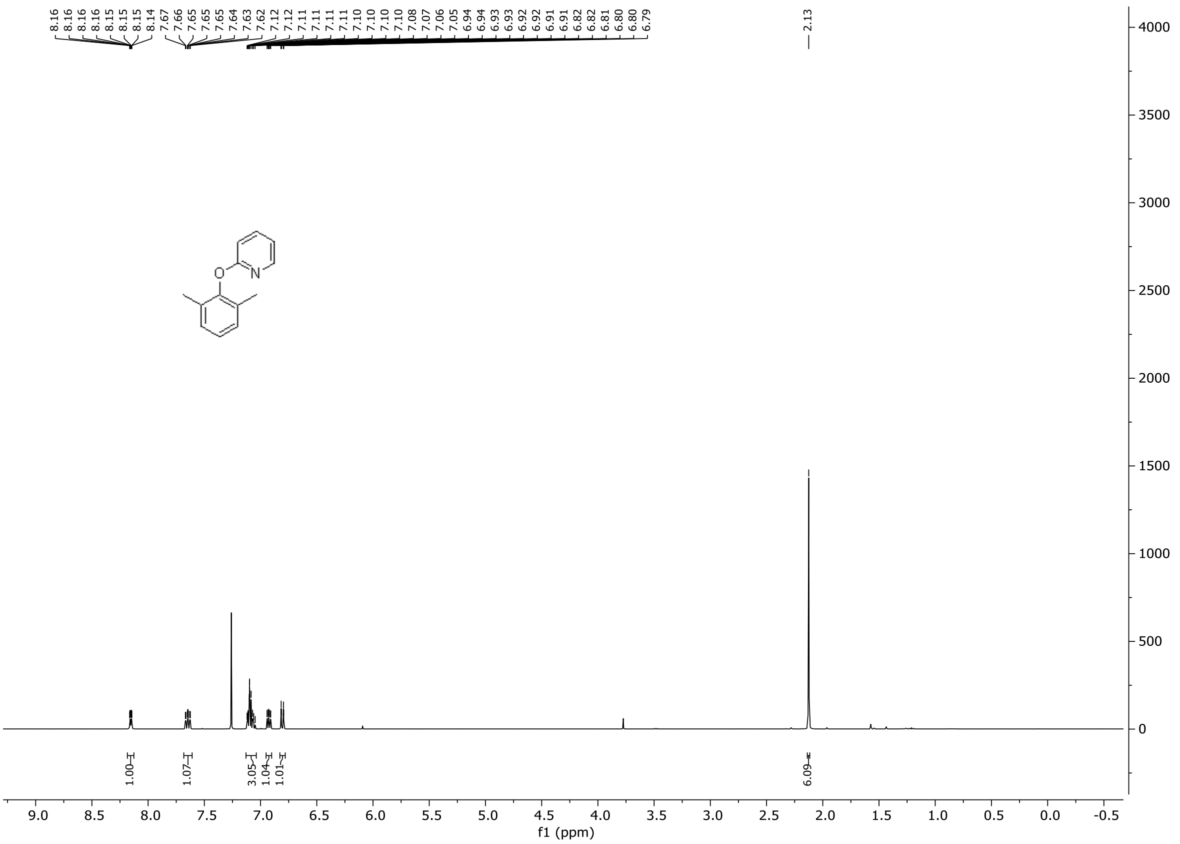
O
N
f1 (ppm)

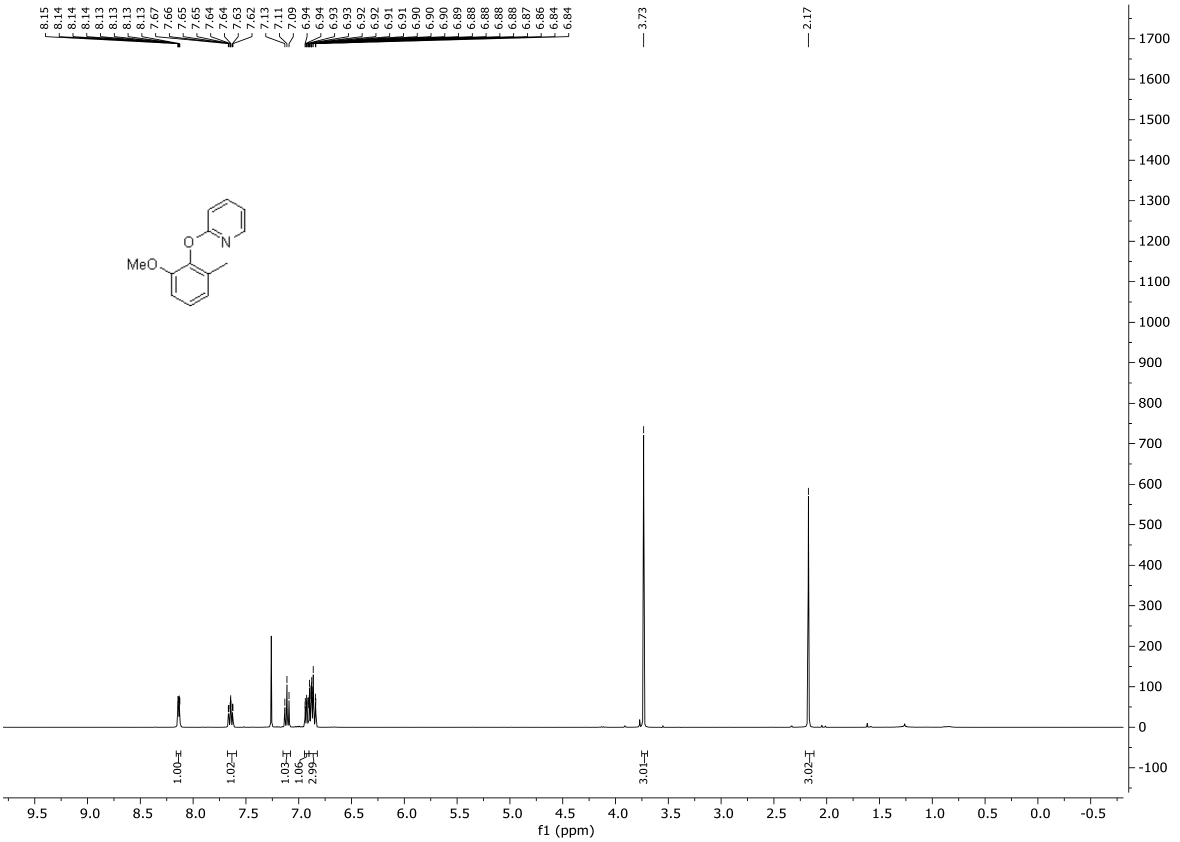
O
N
MeO
f1 (ppm)

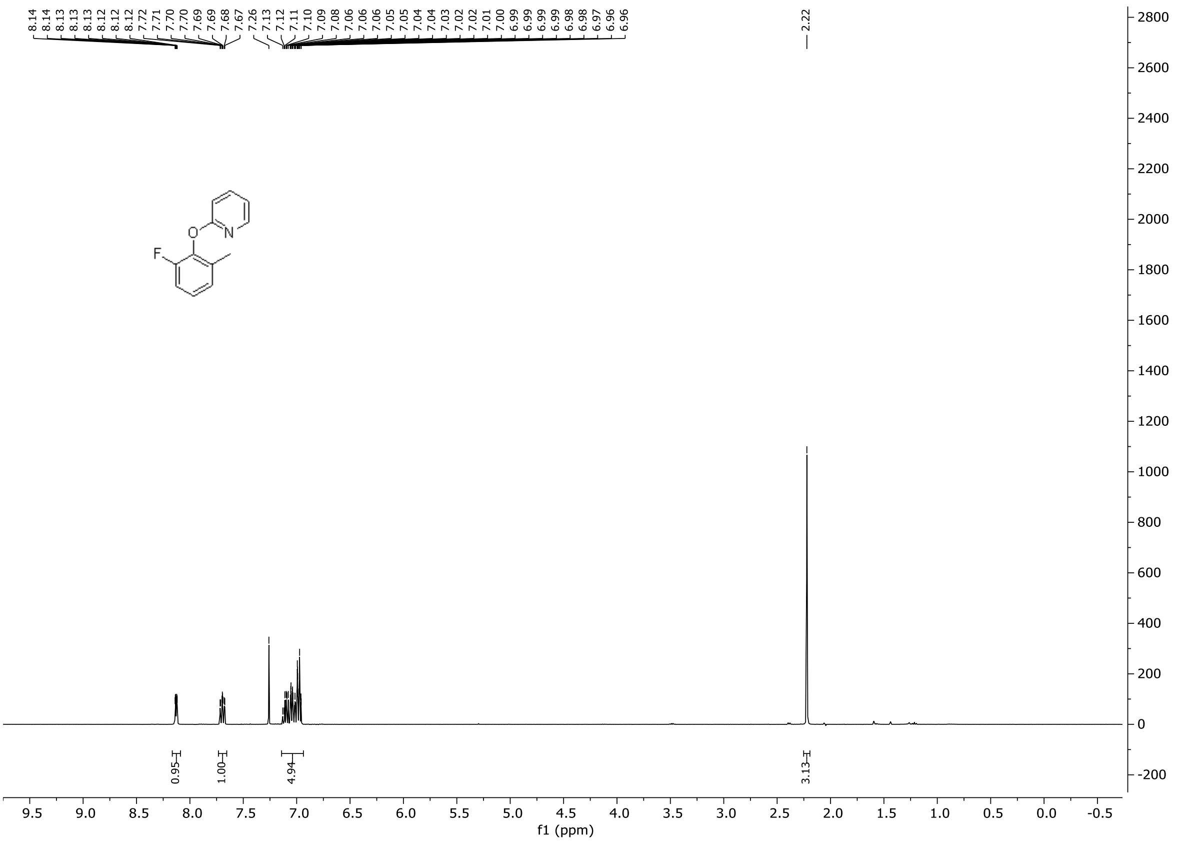
8.14 8.14 8.13 8.13 8.13 8.12 8.12 8.12 7.72 7.71 7.70 7.70 7.69 7.68 7.67 7.26 7.13 7.12 7.11 7.10 7.09 7.08 7.06 7.06 7.06 7.05 7.05 7.04 7.04 7.03 7.02 7.02 7.01 7.00 6.99 6.99 6.99 6.99 6.98 6.98 6.97 6.96 6.96
2.22
F
O
N
0.95
1.00
4.94
3.13
f1 (ppm)

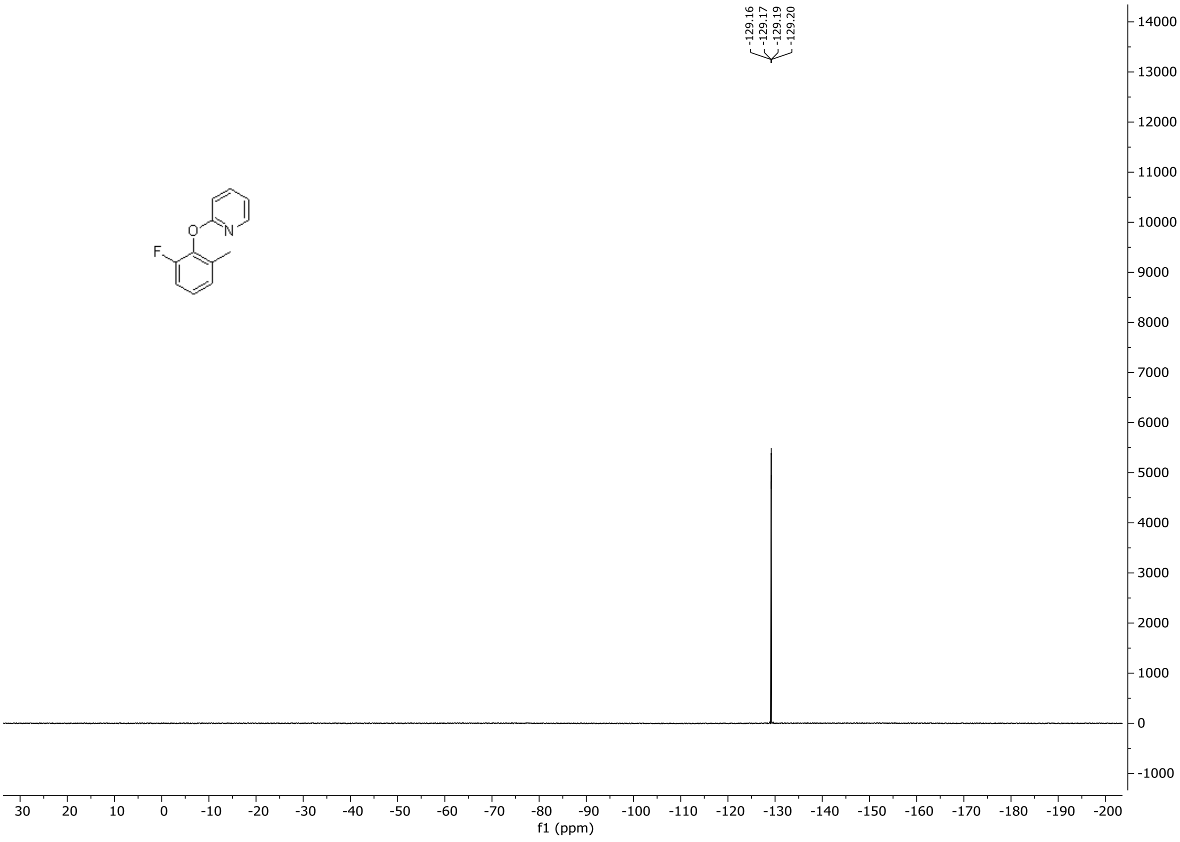
-129.16
-129.17
-129.19
-129.20
F
O
N
f1 (ppm)

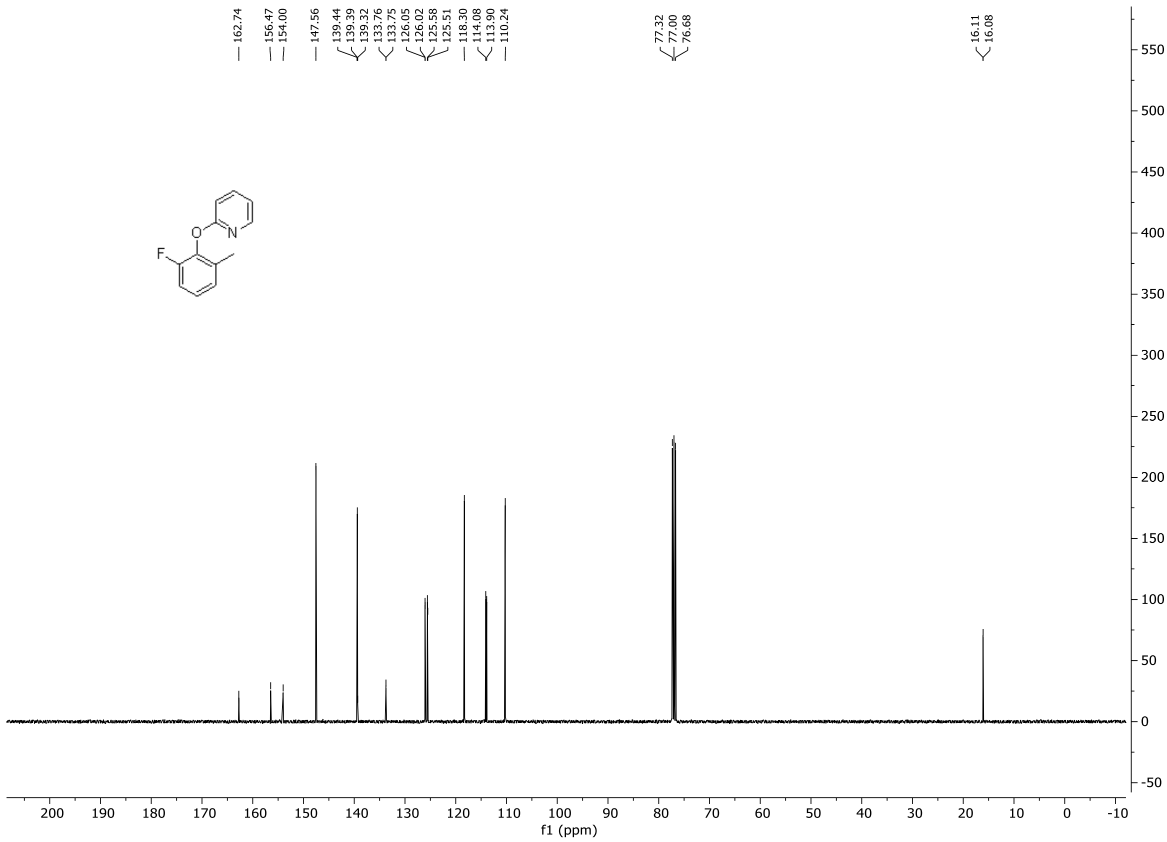
162.74
156.47
154.00
147.56
139.44
139.39
139.32
133.76
133.75
126.05
126.02
125.58
125.51
118.30
114.08
113.90
110.24
77.32
77.00
76.68
16.11
16.08
O
N
F
f1 (ppm)

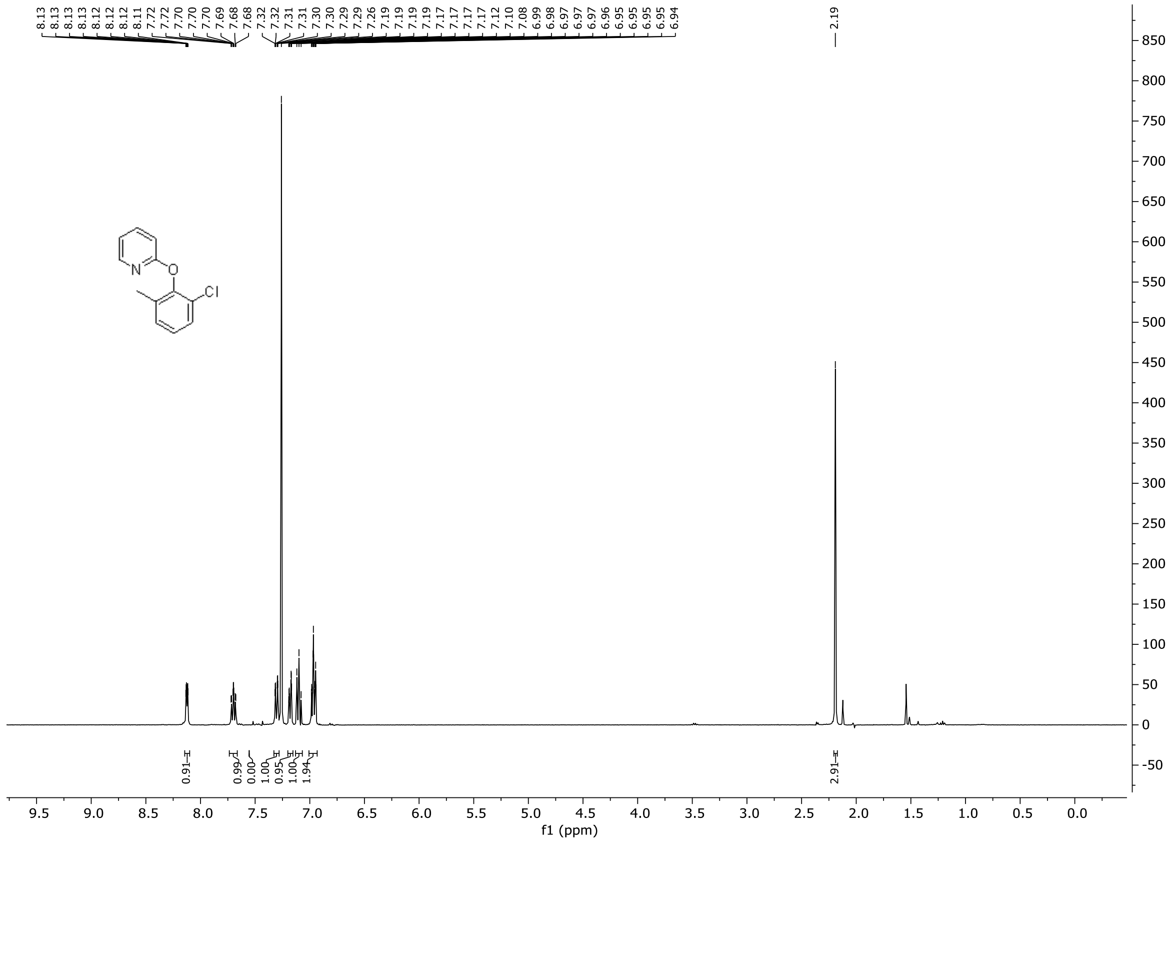
8.13
8.13
8.13
8.12
8.12
8.12
8.11
7.72
7.72
7.70
7.70
7.69
7.68
7.68
7.32
7.32
7.31
7.31
7.30
7.29
7.29
7.26
7.19
7.19
7.19
7.19
7.17
7.17
7.17
7.12
7.10
7.08
6.99
6.98
6.97
6.97
6.96
6.95
6.95
6.95
6.94
2.19
N
O
Cl
0.91
0.99
0.00
1.00
0.95
1.00
1.94
2.91
f1 (ppm)

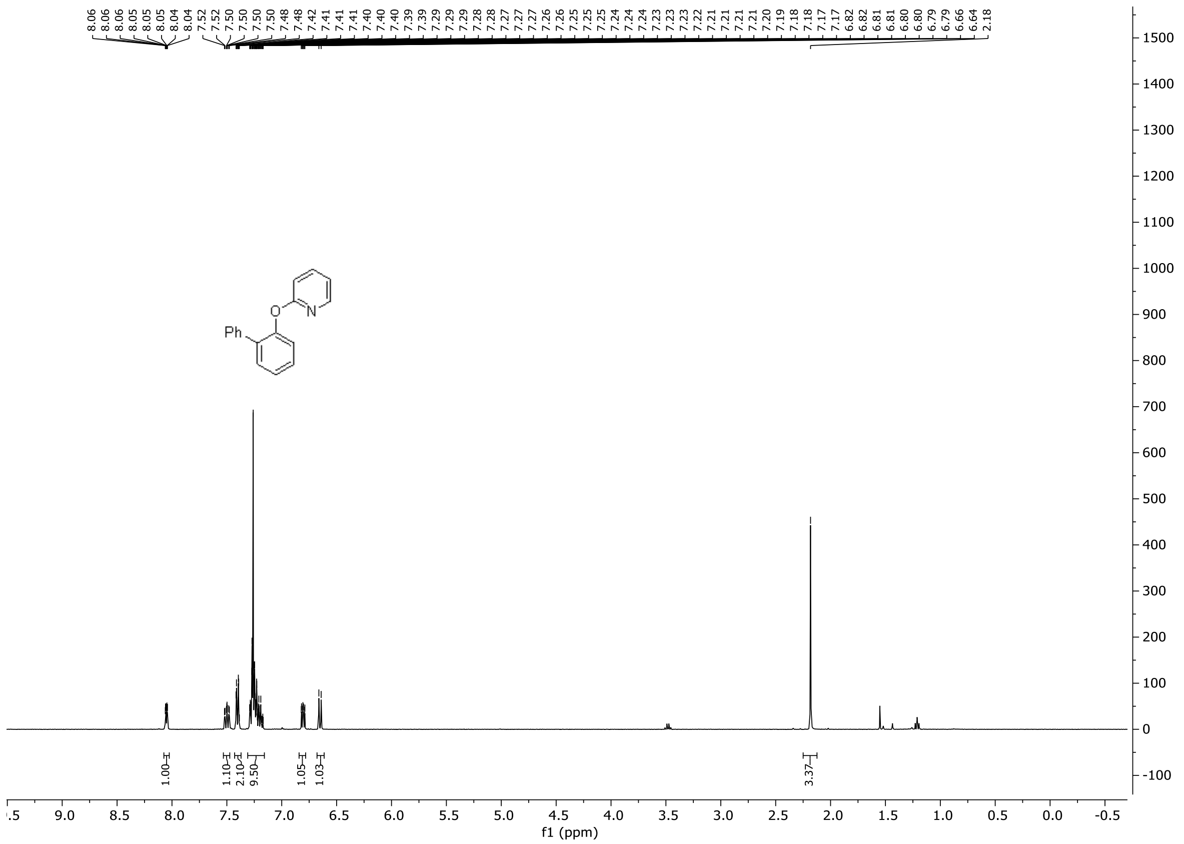
8.06 8.06 8.06 8.05 8.05 8.05 8.04 8.04 7.52 7.52 7.50 7.50 7.50 7.50 7.48 7.48 7.42 7.41 7.41 7.40 7.40 7.40 7.39 7.39 7.29 7.29 7.29 7.28 7.28 7.27 7.27 7.27 7.26 7.26 7.25 7.25 7.25 7.24 7.24 7.24 7.23 7.23 7.23 7.22 7.21 7.21 7.21 7.21 7.20 7.19 7.18 7.18 7.17 7.17 6.82 6.82 6.81 6.81 6.80 6.80 6.79 6.79 6.66 6.64 2.18
Ph
O
N
1.00
1.10
2.10
9.50
1.05
1.03
3.37
f1 (ppm)

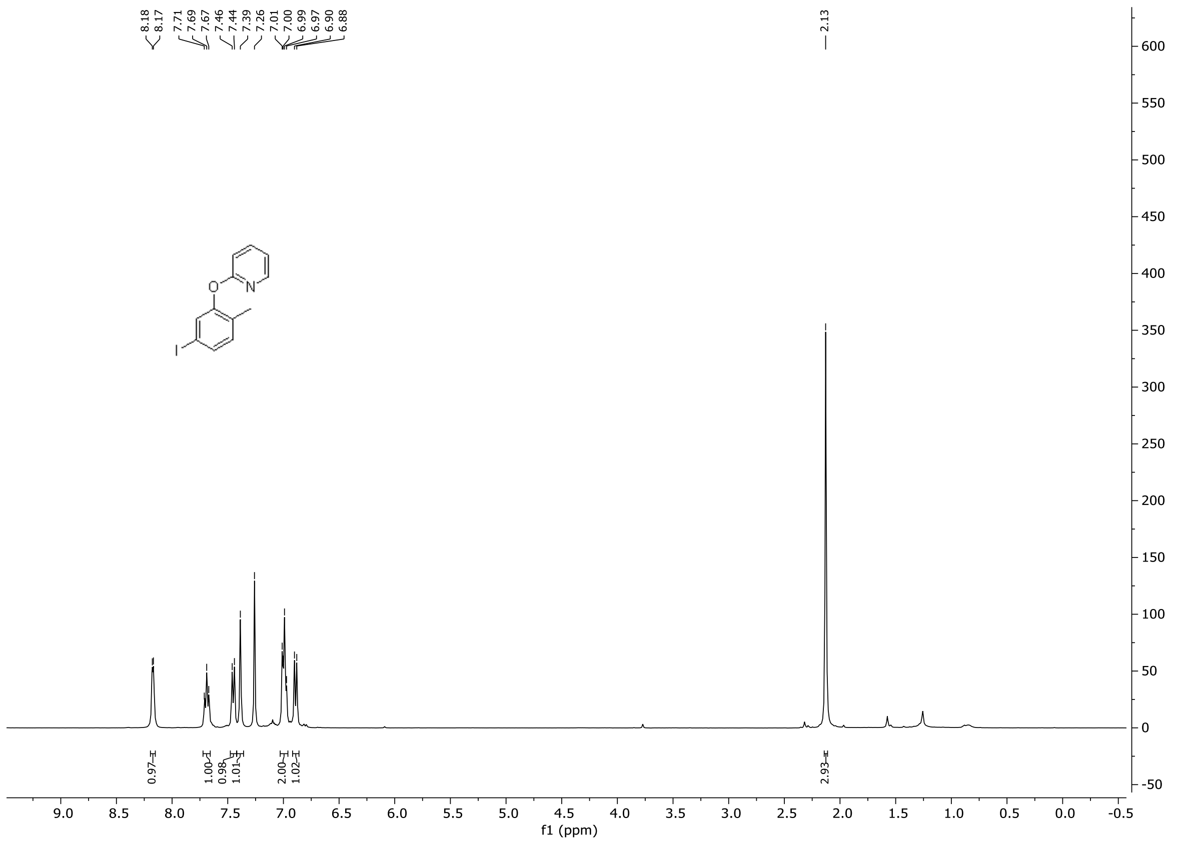
8.18 8.17 7.71 7.69 7.67 7.46 7.44 7.39 7.26 7.01 7.00 6.99 6.97 6.90 6.88
2.13
O
N
I
0.97
1.00
0.98
1.01
2.00
1.02
2.93
f1 (ppm)

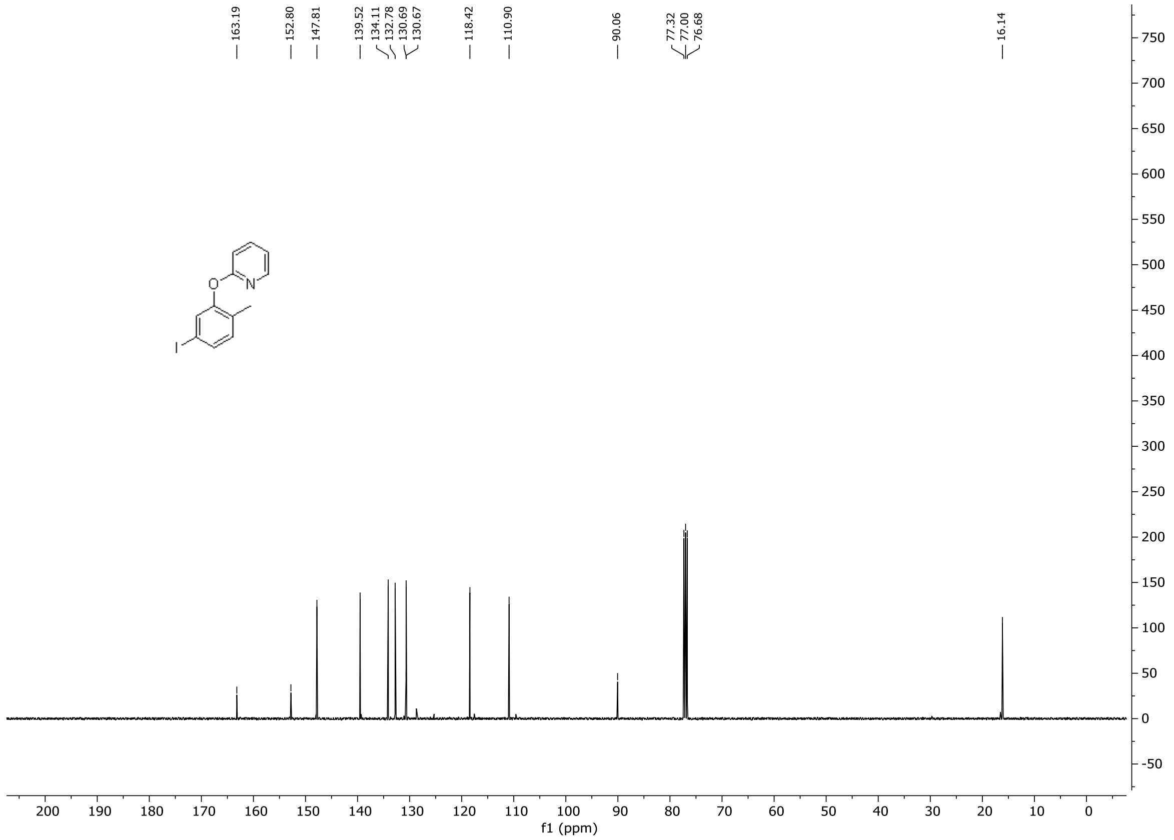
163.19
152.80
147.81
139.52
134.11
132.78
130.69
130.67
118.42
110.90
90.06
77.32
77.00
76.68
16.14
O
N
I
f1 (ppm)

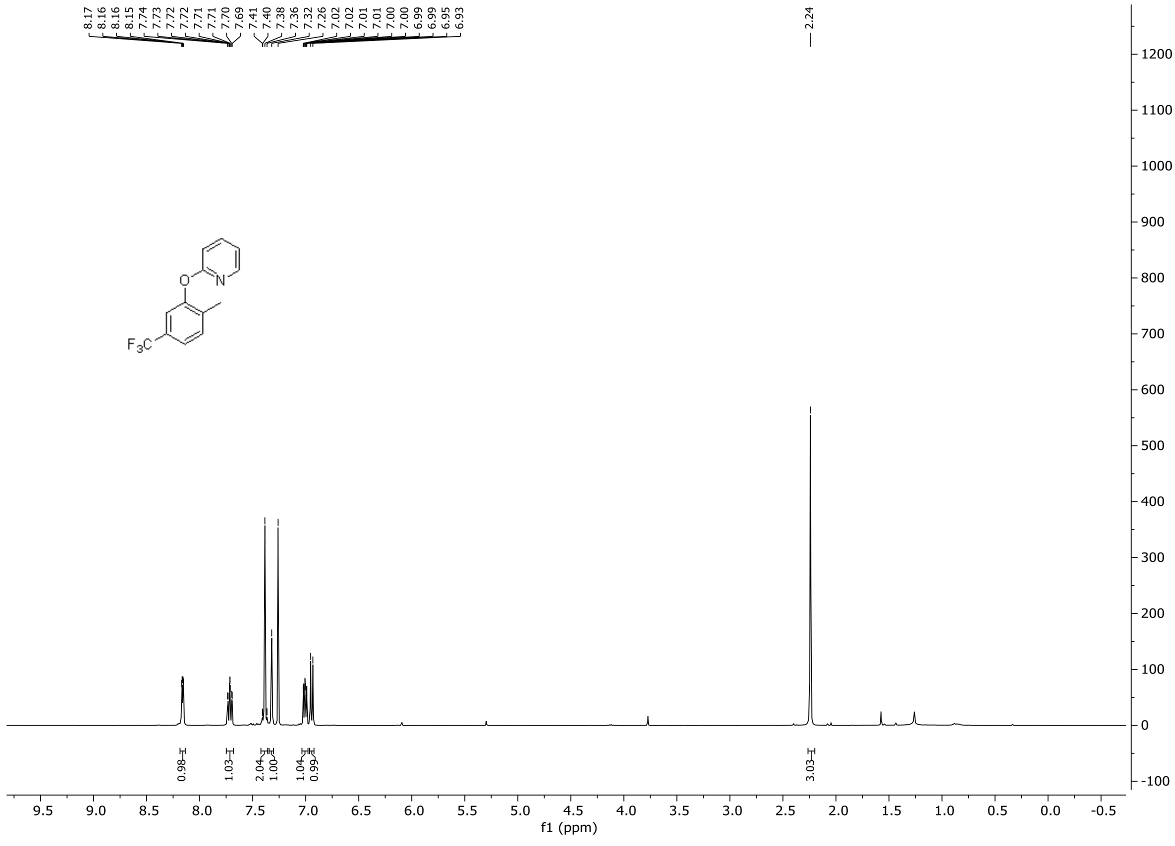
8.17
8.16
8.16
8.15
7.74
7.73
7.72
7.72
7.71
7.71
7.70
7.69
7.41
7.40
7.38
7.36
7.32
7.26
7.02
7.02
7.01
7.01
7.00
6.99
6.99
6.95
6.93
2.24
O
N
F3C
0.98
1.03
2.04
1.00
1.04
0.99
3.03
f1 (ppm)

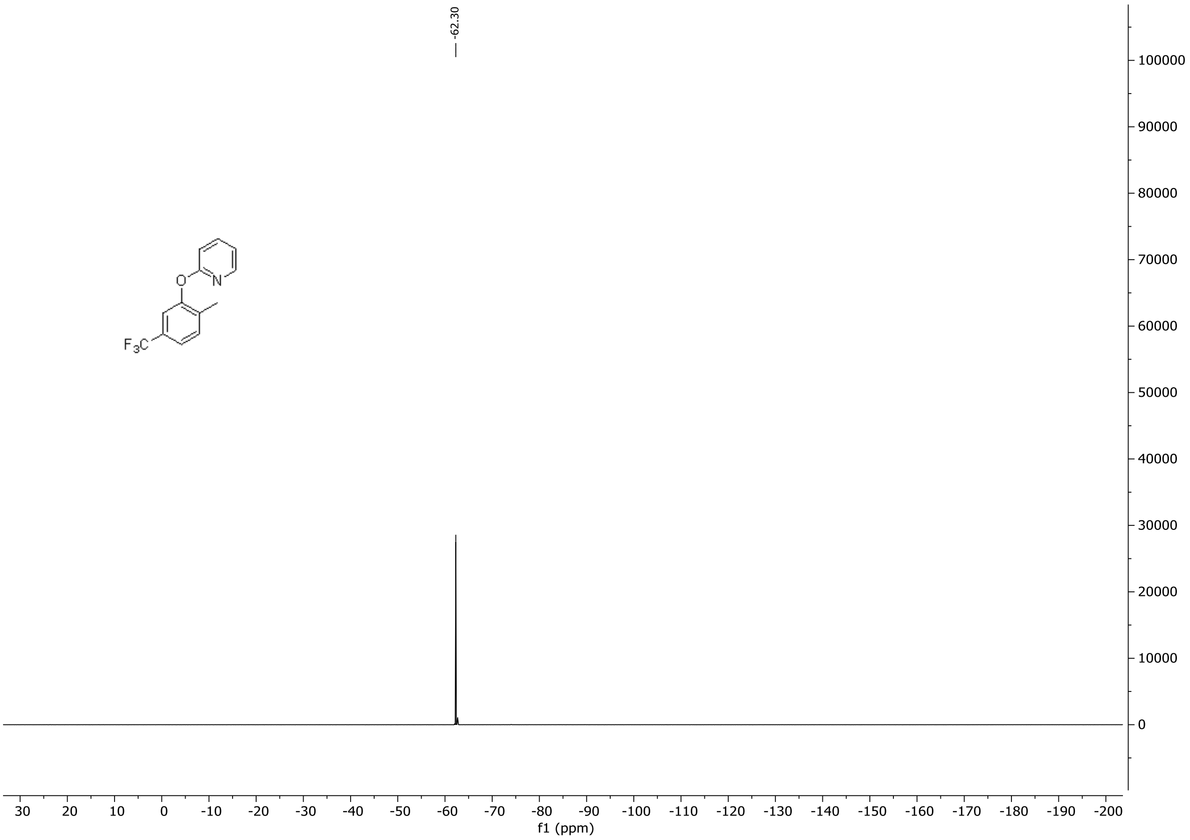
-62.30
O
N
F3C
f1 (ppm)

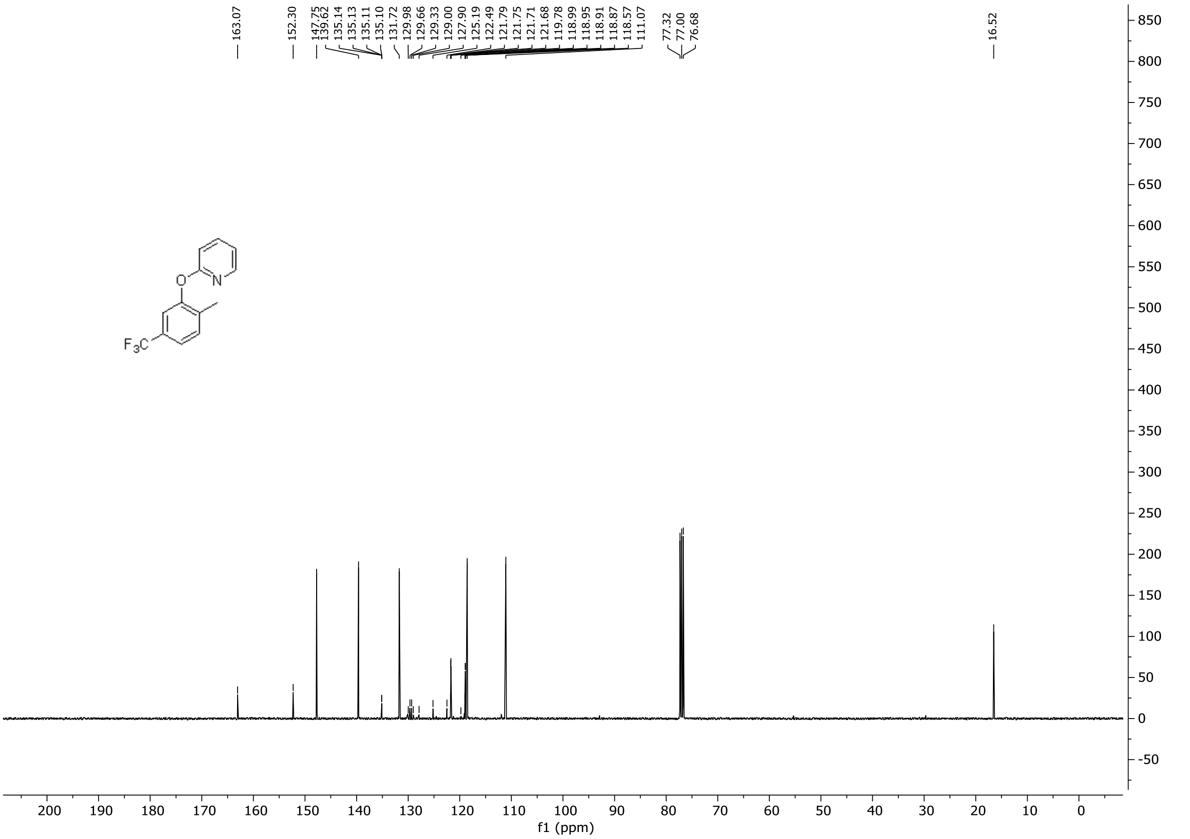
163.07
152.30
147.75
139.62
135.14
135.13
135.11
135.10
131.72
129.98
129.66
129.33
129.00
127.90
125.19
122.49
121.79
121.75
121.71
121.68
119.78
118.99
118.95
118.91
118.87
118.57
111.07
77.32
77.00
76.68
16.52
O
N
F3C
f1 (ppm)

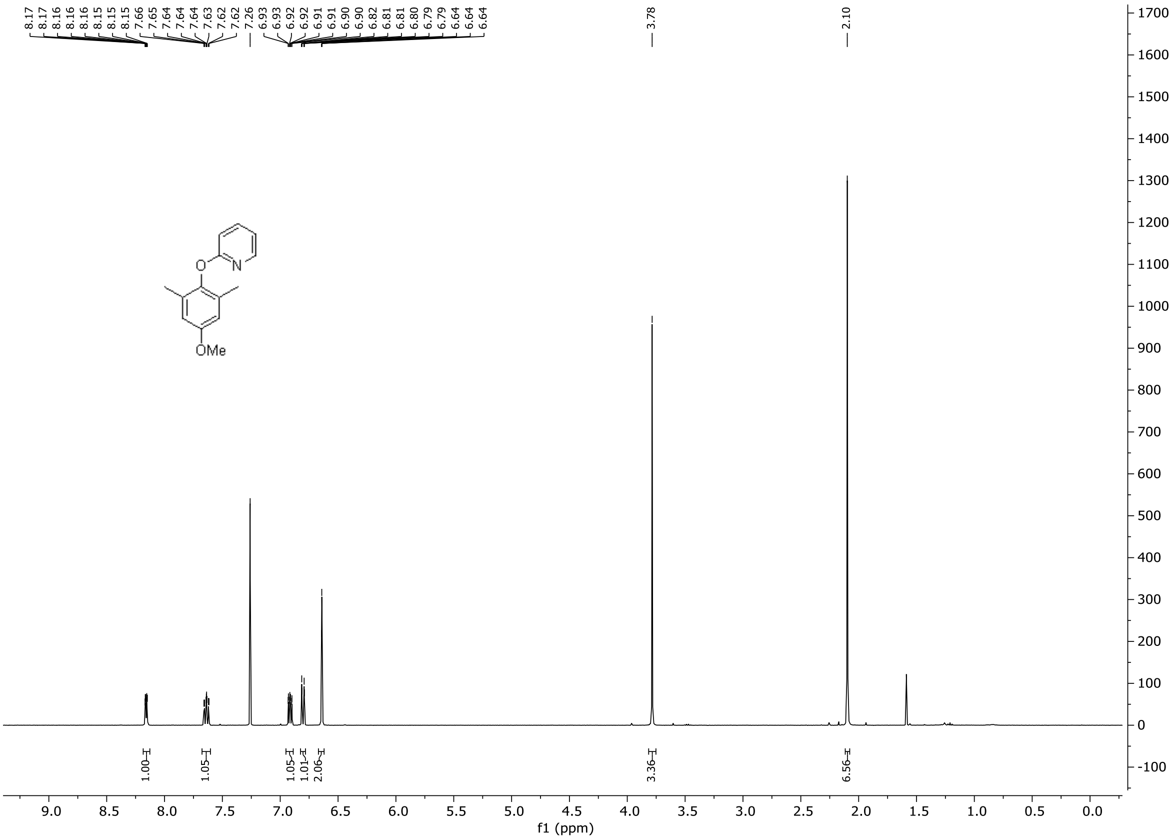
8.17 8.17 8.16 8.16 8.15 8.15 8.15 7.66 7.65 7.64 7.64 7.63 7.63 7.62 7.62 7.26 6.93 6.93 6.92 6.92 6.91 6.91 6.90 6.90 6.82 6.81 6.81 6.80 6.79 6.79 6.64 6.64 6.64
3.78
2.10
OMe
N
O
1.00
1.05
1.05
1.01
2.06
3.36
6.56
f1 (ppm)

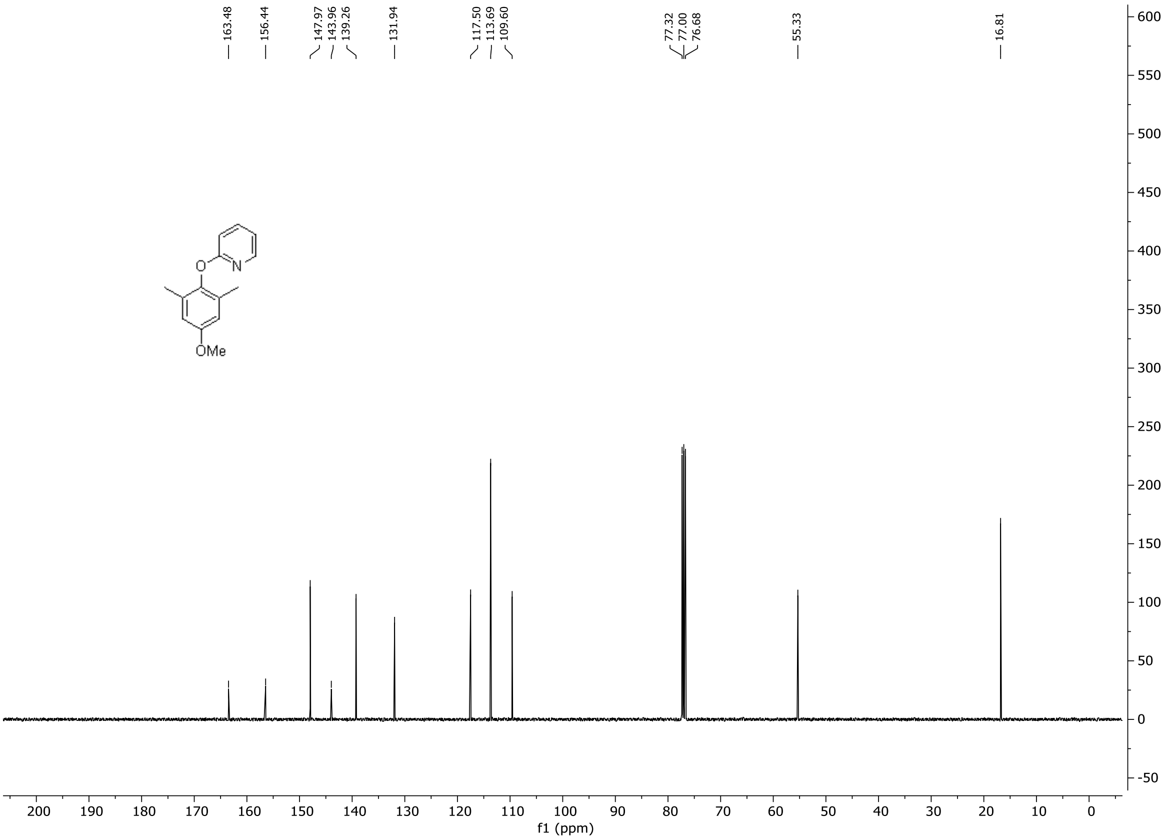
163.48
156.44
147.97 143.96 139.26
131.94
117.50 113.69 109.60
77.32 77.00 76.68
55.33
16.81
OMe
N
O
f1 (ppm)

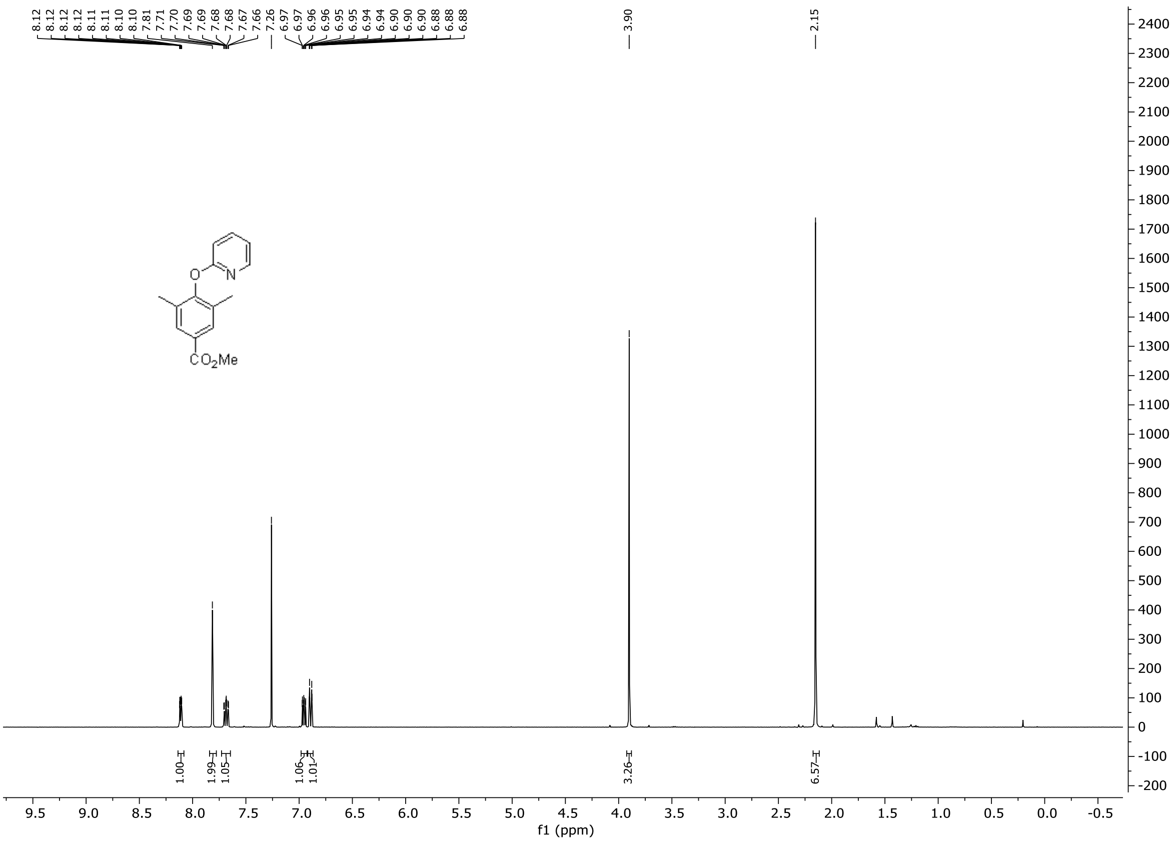
8.12 8.12 8.12 8.12 8.11 8.11 8.10 8.10 7.81 7.71 7.70 7.69 7.69 7.68 7.68 7.67 7.66 7.26 6.97 6.97 6.96 6.96 6.95 6.95 6.94 6.94 6.90 6.90 6.90 6.90 6.88 6.88 6.88
3.90
2.15
O
N
CO2Me
1.00
1.99
1.05
1.06
1.01
3.26
6.57
f1 (ppm)

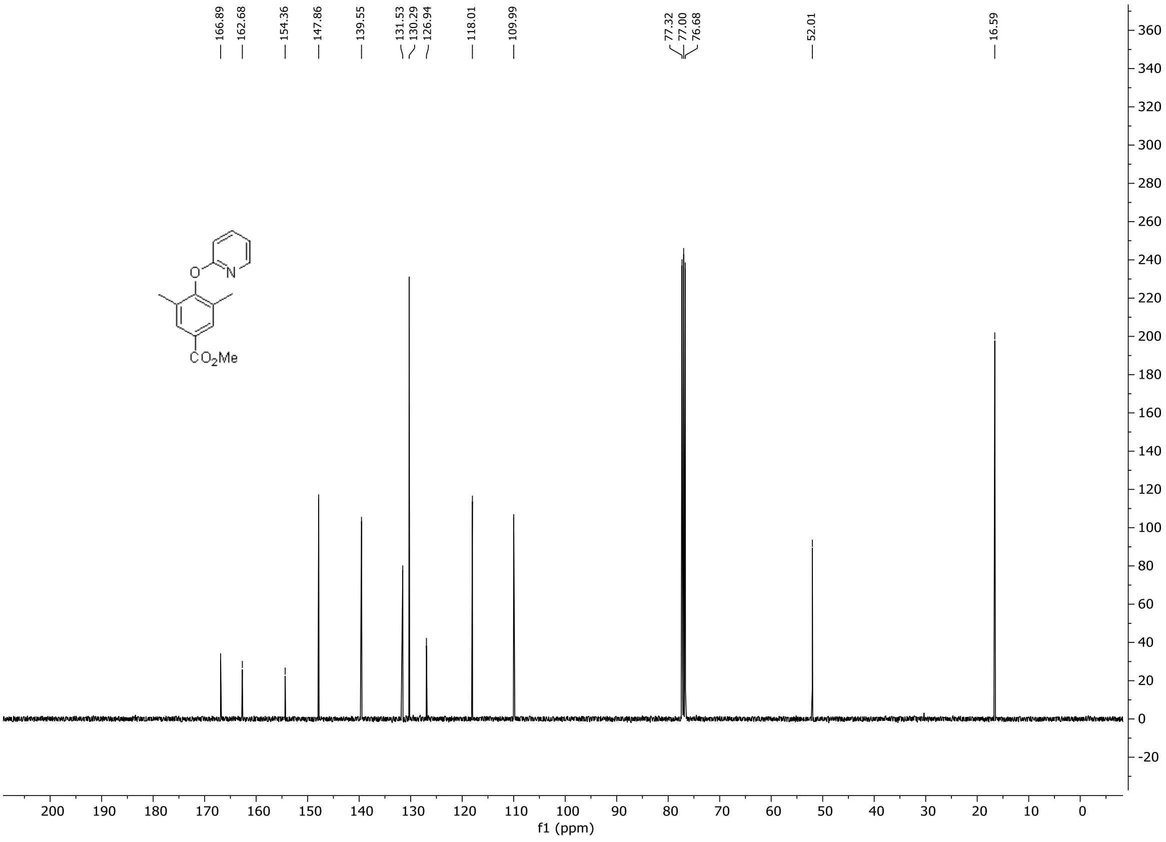
166.89 162.68 154.36 147.86 139.55 131.53 130.29 126.94 118.01 109.99
77.32 77.00 76.68
52.01
16.59
O
N
CO2Me
f1 (ppm)

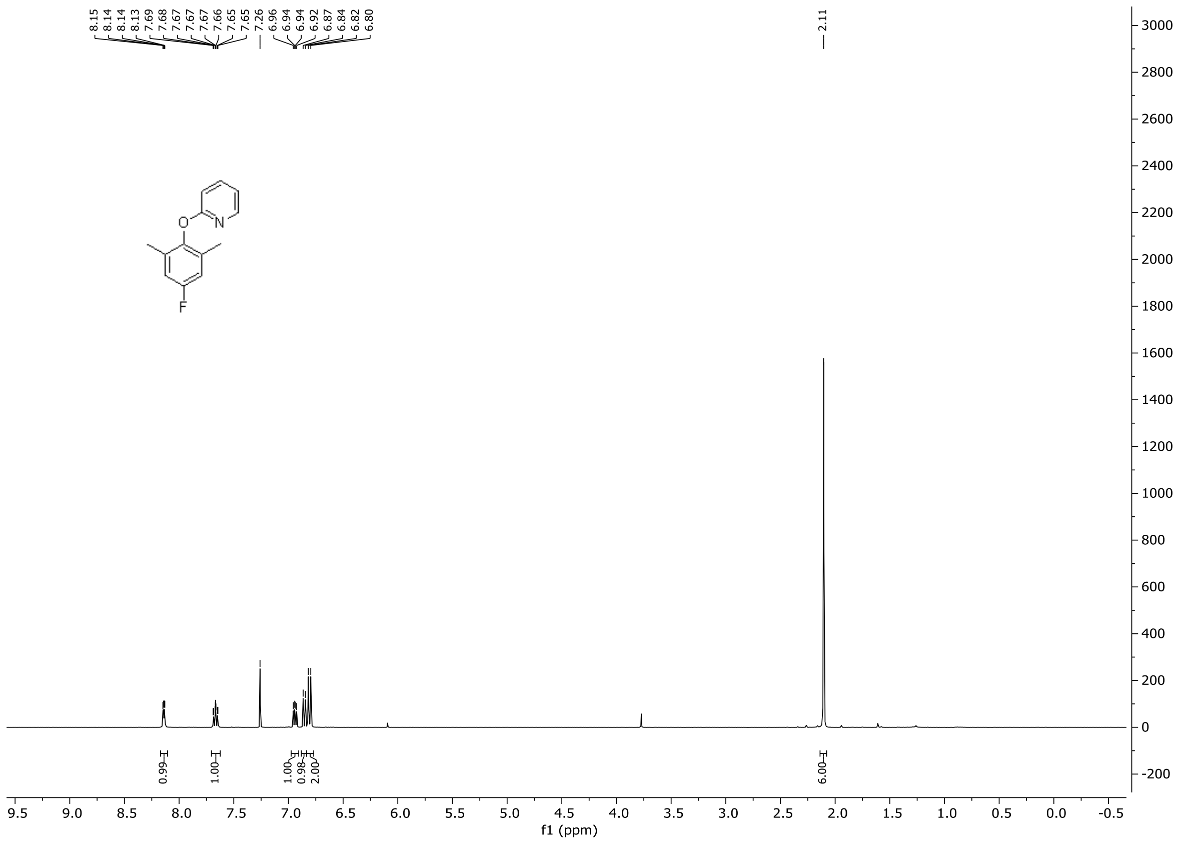
8.15
8.14
8.14
8.13
7.69
7.68
7.67
7.67
7.67
7.66
7.65
7.65
7.26
6.96
6.94
6.94
6.92
6.87
6.84
6.82
6.80
2.11
0.99
1.00
1.00
0.98
2.00
6.00
f1 (ppm)

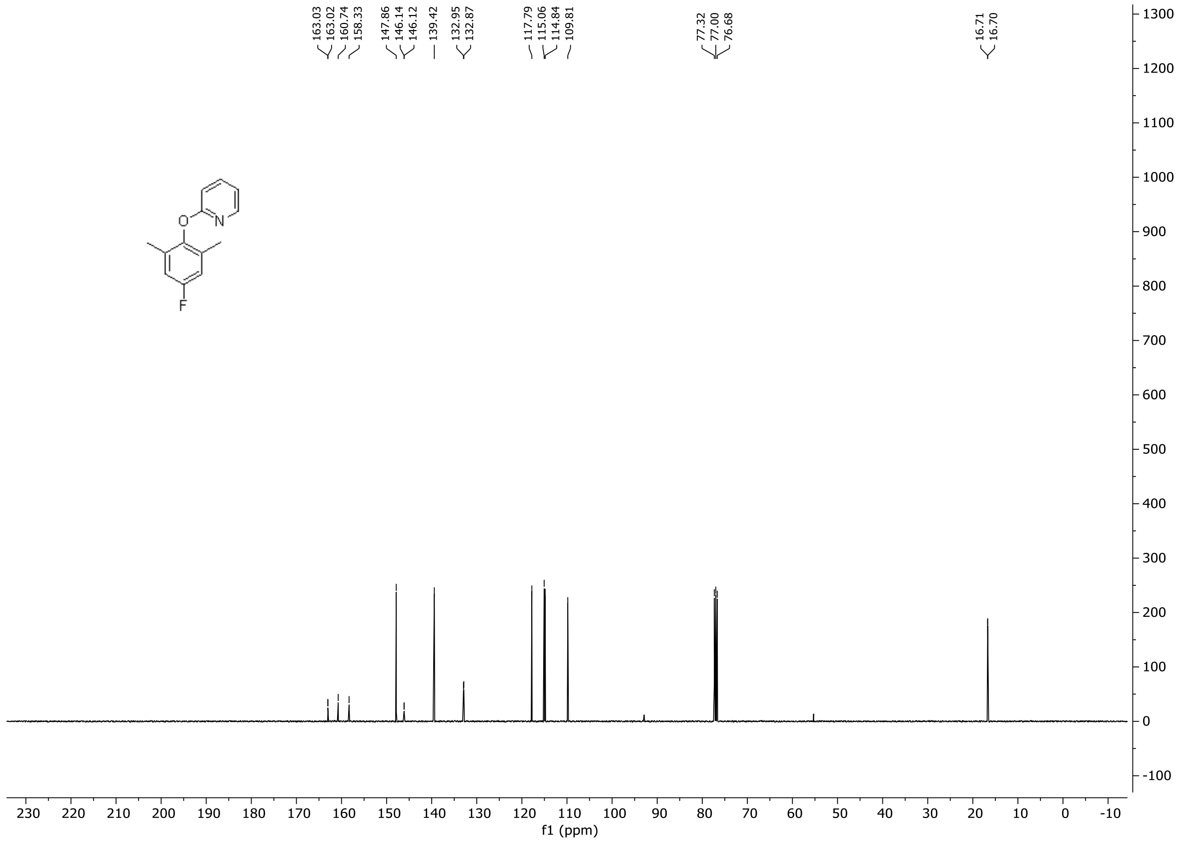
163.03
163.02
160.74
158.33
147.86
146.14
146.12
139.42
132.95
132.87
117.79
115.06
114.84
109.81
77.32
77.00
76.68
16.71
16.70
f1 (ppm)

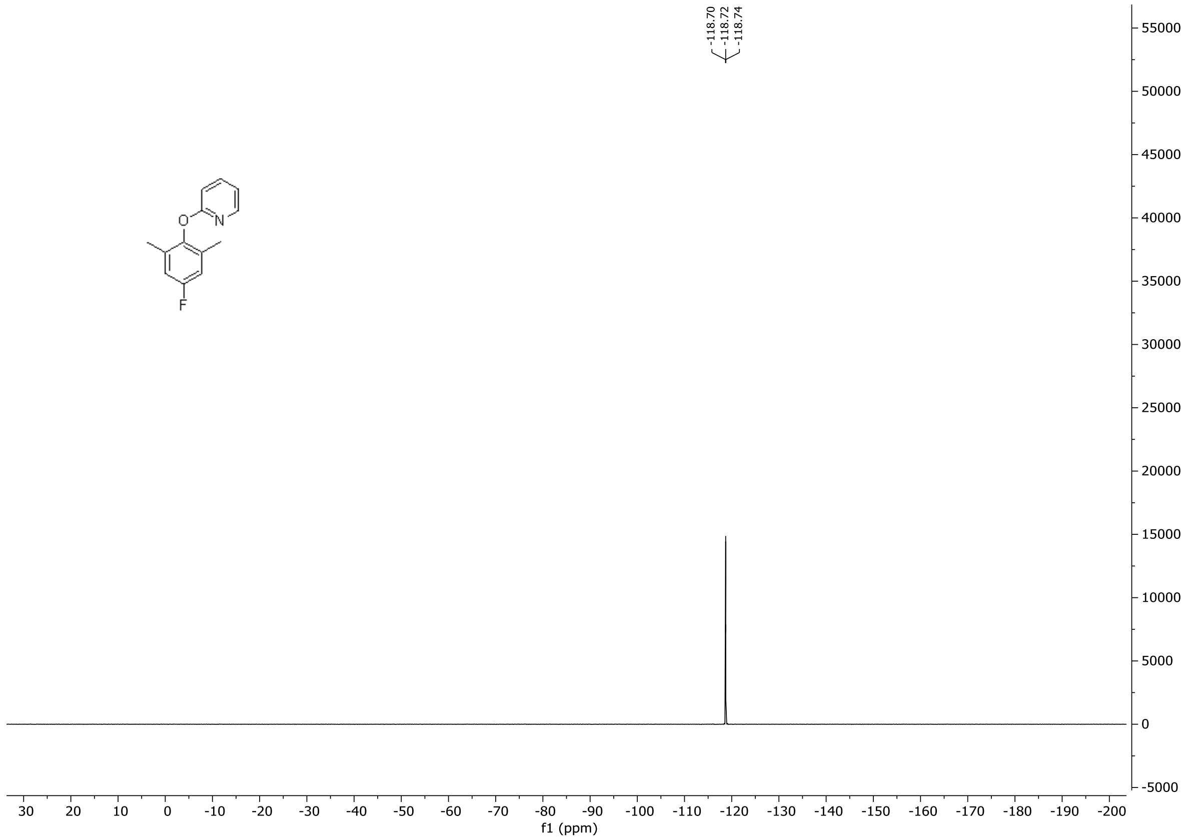
-118.70
-118.72
-118.74
O
N
F
f1 (ppm)

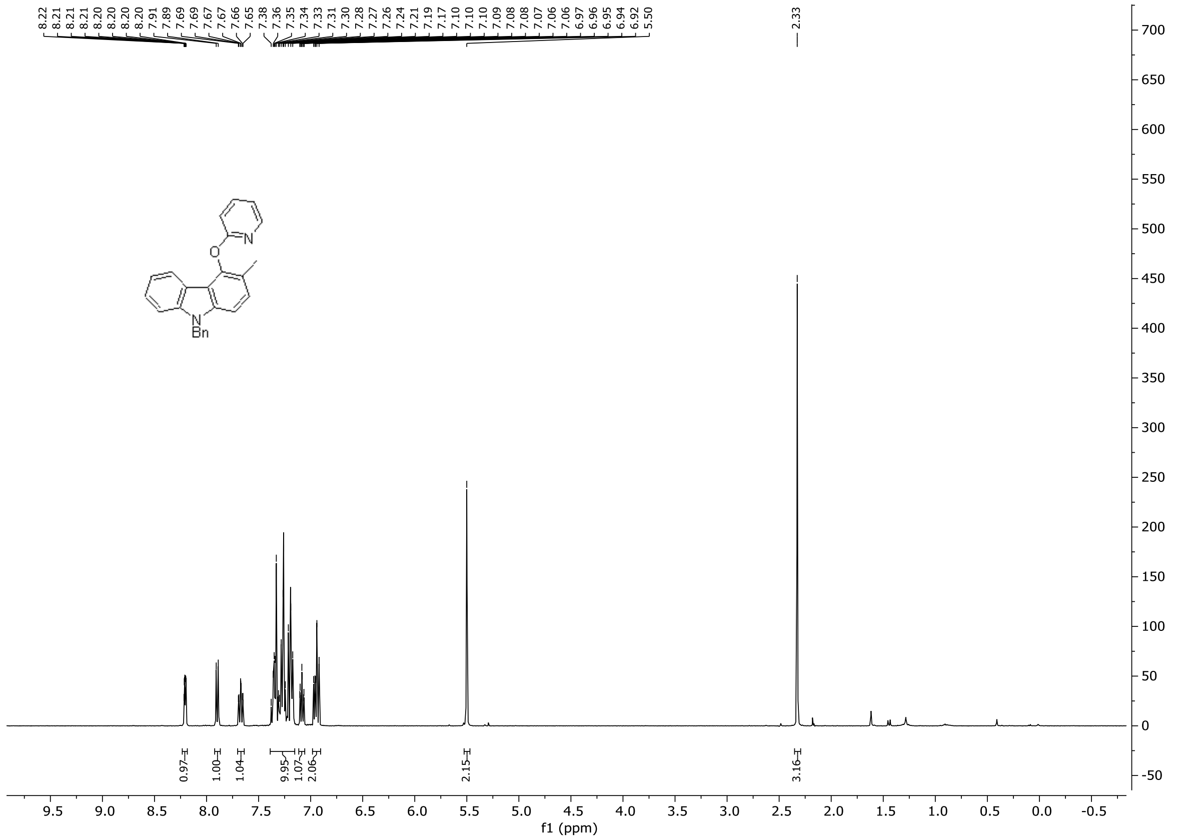
8.22 8.21 8.21 8.20 8.20 8.20 7.91 7.89 7.69 7.69 7.67 7.66 7.65 7.38 7.36 7.35 7.34 7.33 7.31 7.30 7.28 7.27 7.26 7.24 7.21 7.19 7.17 7.10 7.10 7.09 7.08 7.08 7.07 7.06 6.97 6.96 6.95 6.94 6.92 5.50
2.33
O
N
N
Bn
0.97
1.00
1.04
9.95
1.07
2.06
2.15
3.16
f1 (ppm)

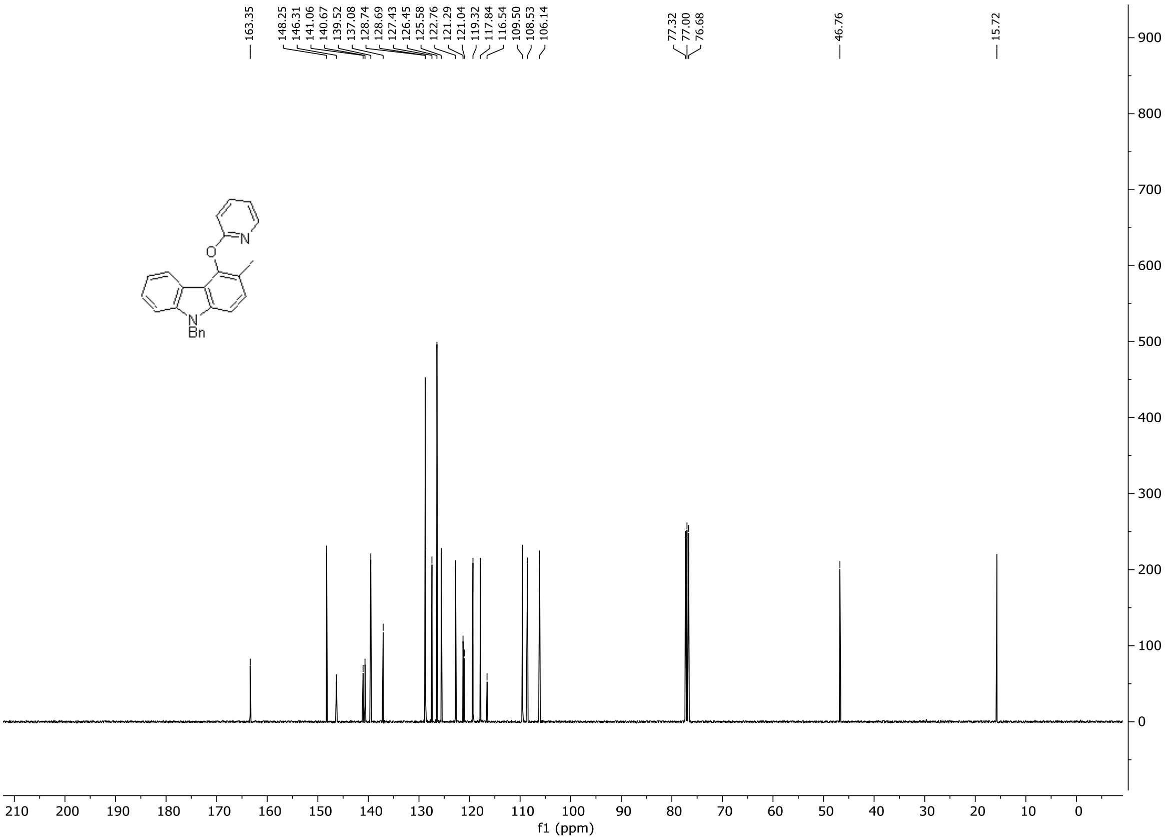
163.35
148.25
146.31
141.06
140.67
139.52
137.08
128.74
128.69
127.43
126.45
125.58
122.76
121.29
121.04
119.32
117.84
116.54
109.50
108.53
106.14
77.32
77.00
76.68
46.76
15.72
O
N
N
Bn
f1 (ppm)

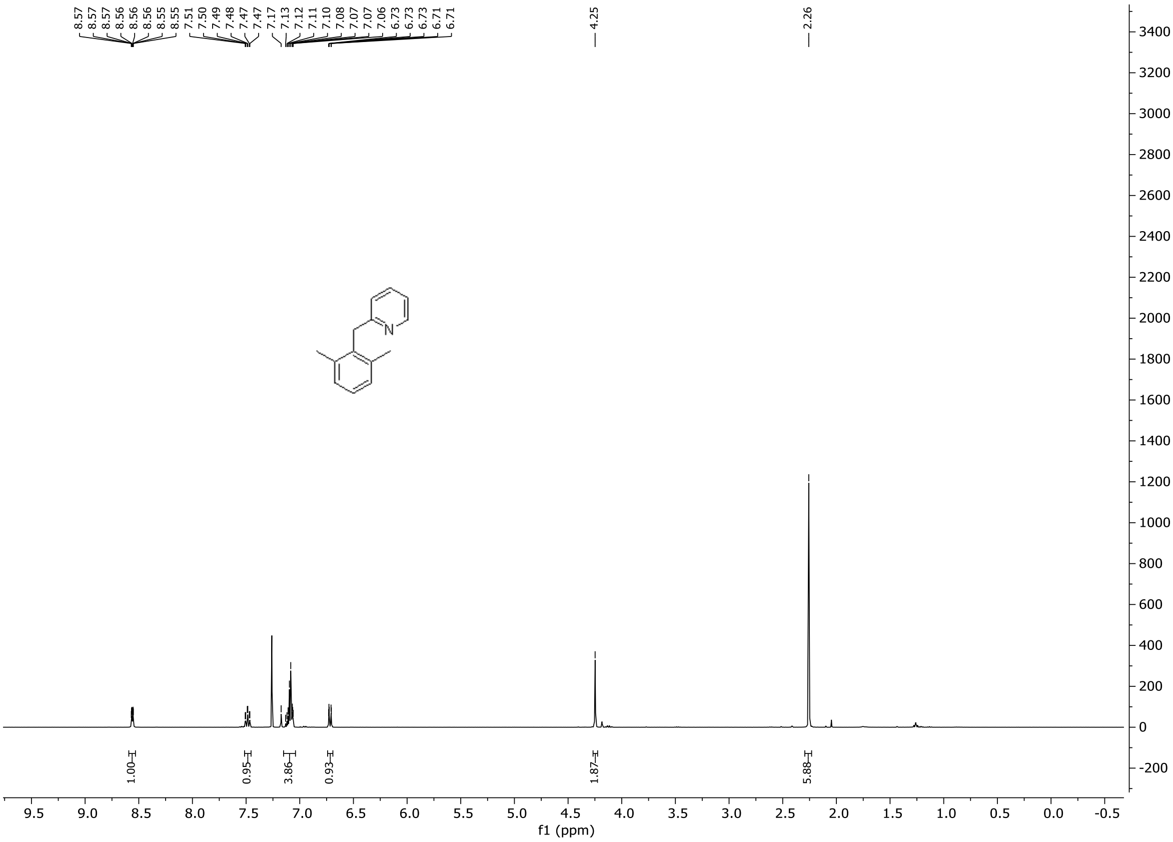
8.57
8.57
8.56
8.56
8.56
8.55
8.55
7.51
7.50
7.49
7.48
7.47
7.47
7.17
7.13
7.12
7.11
7.10
7.08
7.07
7.07
7.06
6.73
6.73
6.73
6.71
6.71
4.25
2.26
N
1.00
0.95
3.86
0.93
1.87
5.88
f1 (ppm)

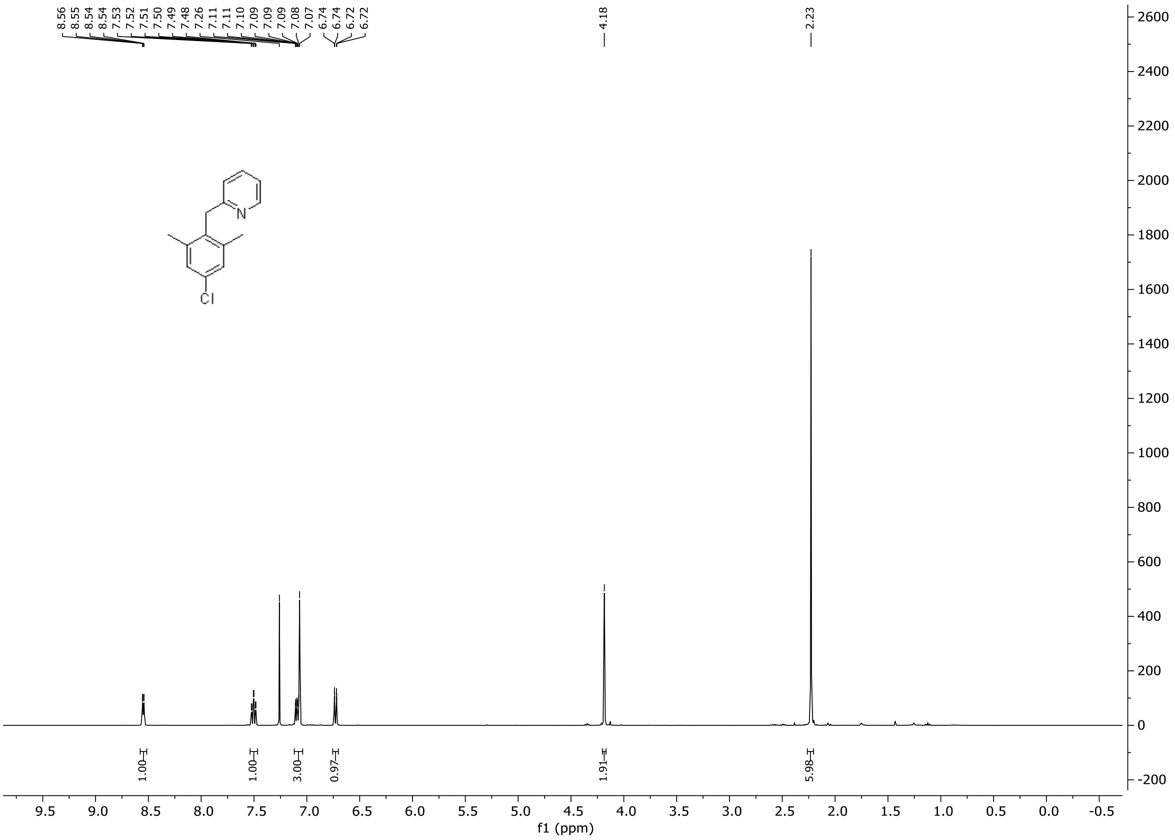
8.56
8.55
8.54
8.54
7.53
7.52
7.51
7.50
7.49
7.48
7.26
7.11
7.11
7.10
7.09
7.09
7.09
7.08
7.07
6.74
6.74
6.72
6.72
4.18
2.23
N
Cl
1.00
1.00
3.00
0.97
1.91
5.98
f1 (ppm)

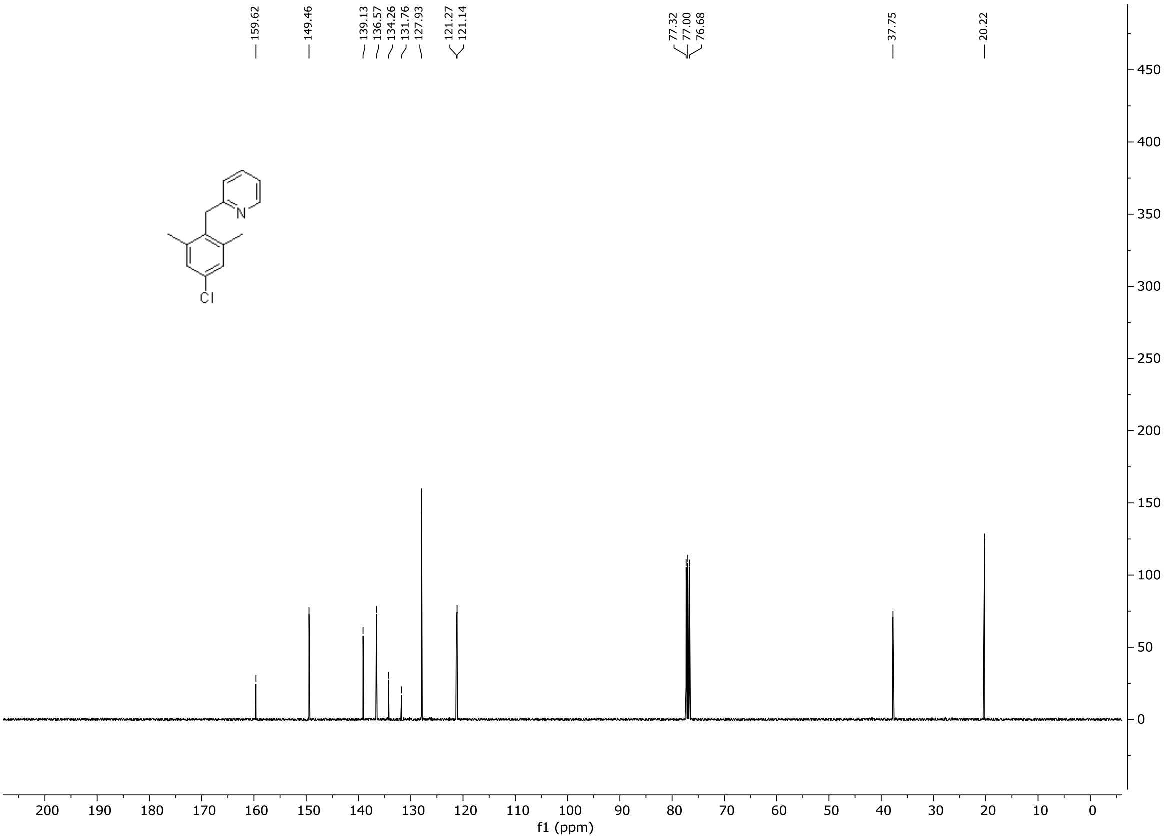
159.62
149.46
139.13
136.57
134.26
131.76
127.93
121.27
121.14
77.32
77.00
76.68
37.75
20.22
N
Cl
f1 (ppm)

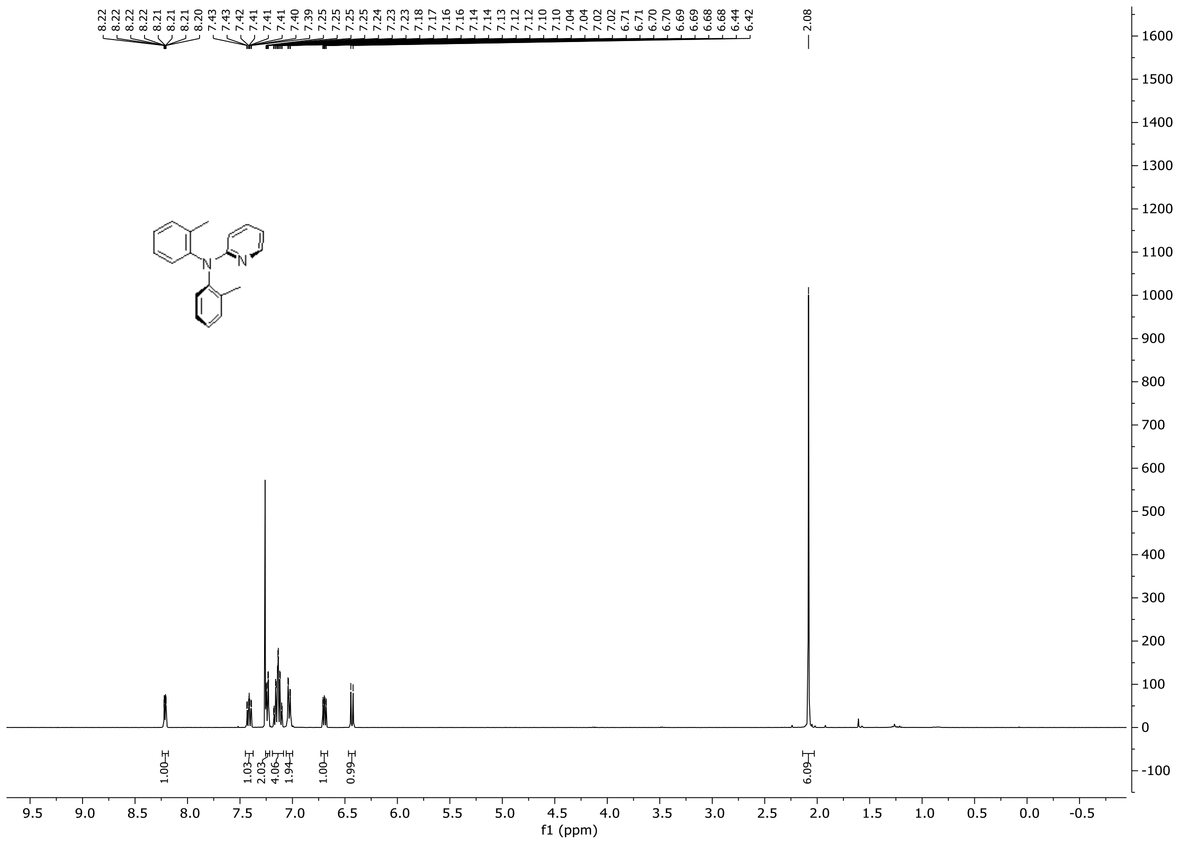
8.22 8.22 8.22 8.22 8.21 8.21 8.21 8.20 7.43 7.43 7.42 7.41 7.41 7.41 7.40 7.39 7.25 7.25 7.25 7.25 7.24 7.23 7.23 7.18 7.17 7.16 7.16 7.14 7.14 7.13 7.12 7.12 7.12 7.10 7.10 7.04 7.04 7.02 7.02 6.71 6.71 6.70 6.70 6.69 6.69 6.68 6.68 6.44 6.42
2.08
N
1.00
1.03
2.03
4.06
1.94
1.00
0.99
6.09
f1 (ppm)

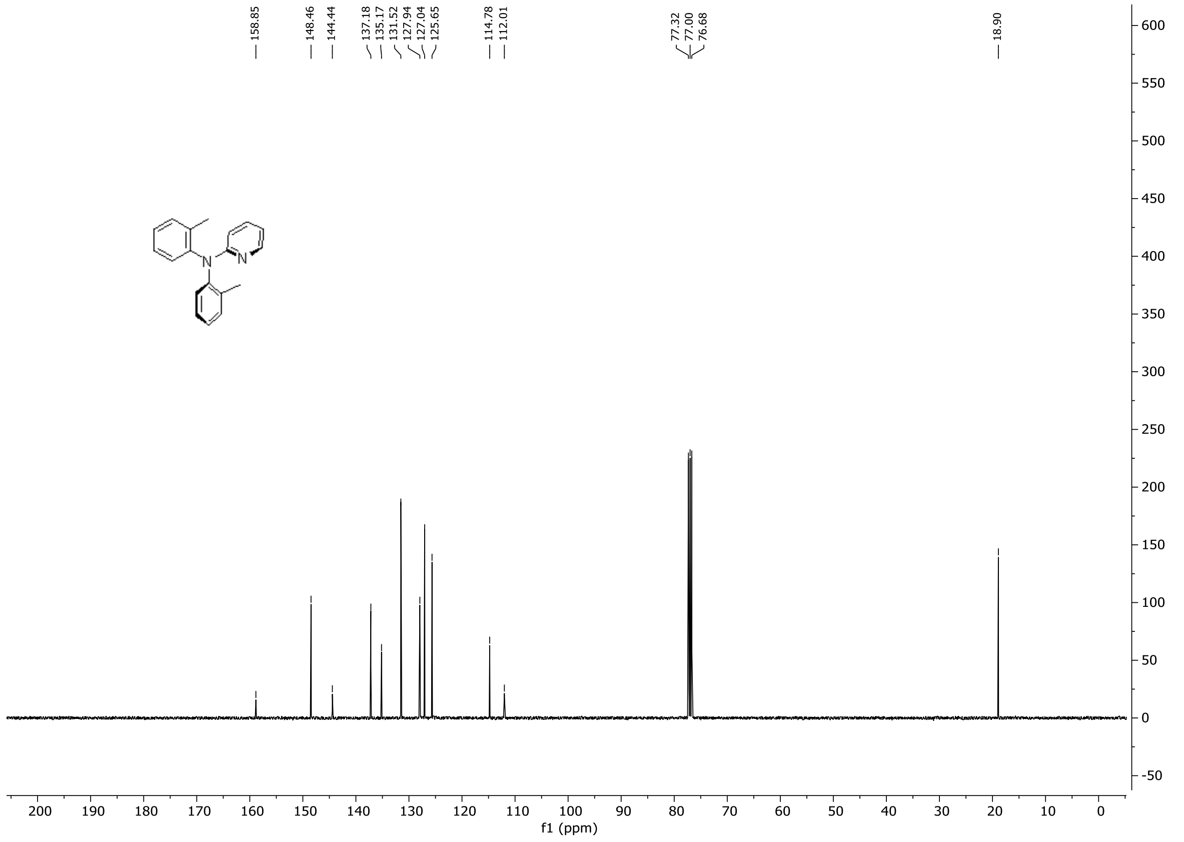
158.85
148.46
144.44
137.18
135.17
131.52
127.94
127.04
125.65
114.78
112.01
77.32
77.00
76.68
18.90
N
f1 (ppm)

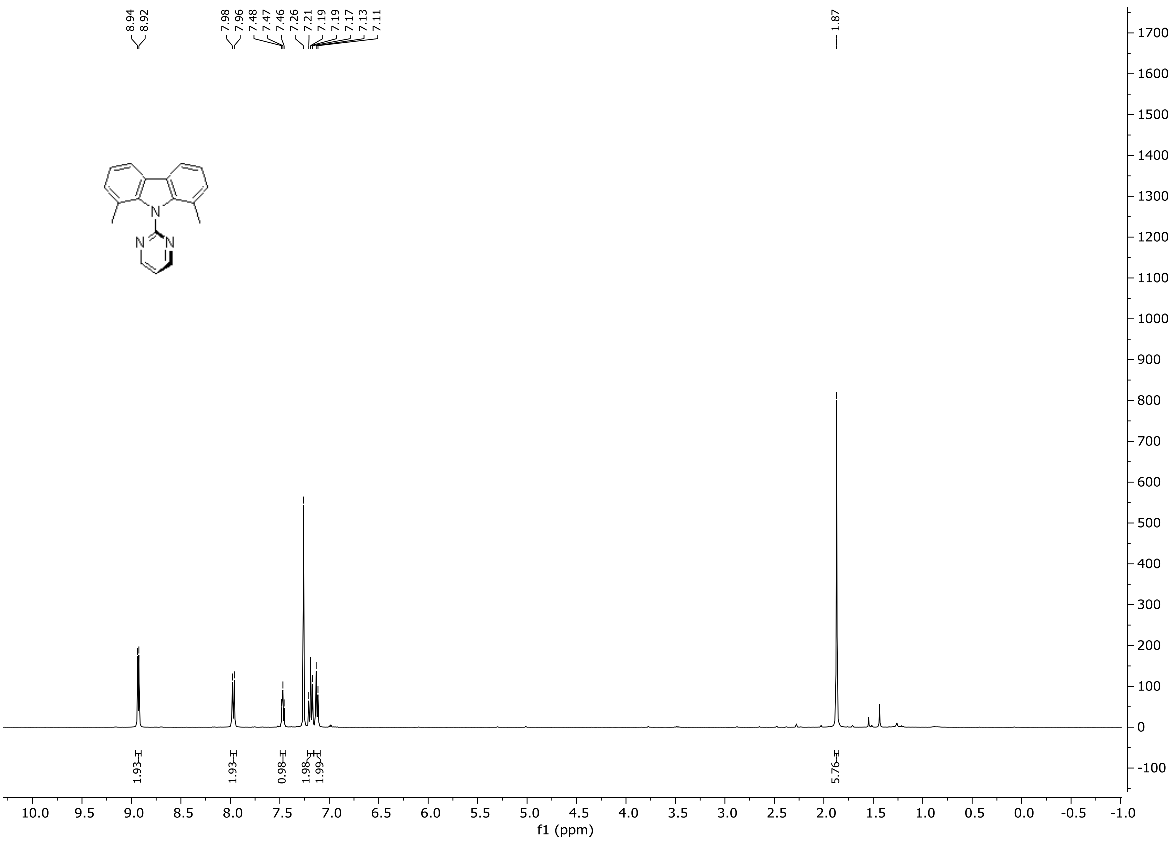
8.94
8.92
7.98
7.96
7.48
7.47
7.46
7.26
7.21
7.19
7.19
7.17
7.13
7.11
1.87
N
N
N
1.93
1.93
0.98
1.98
1.99
5.76
f1 (ppm)

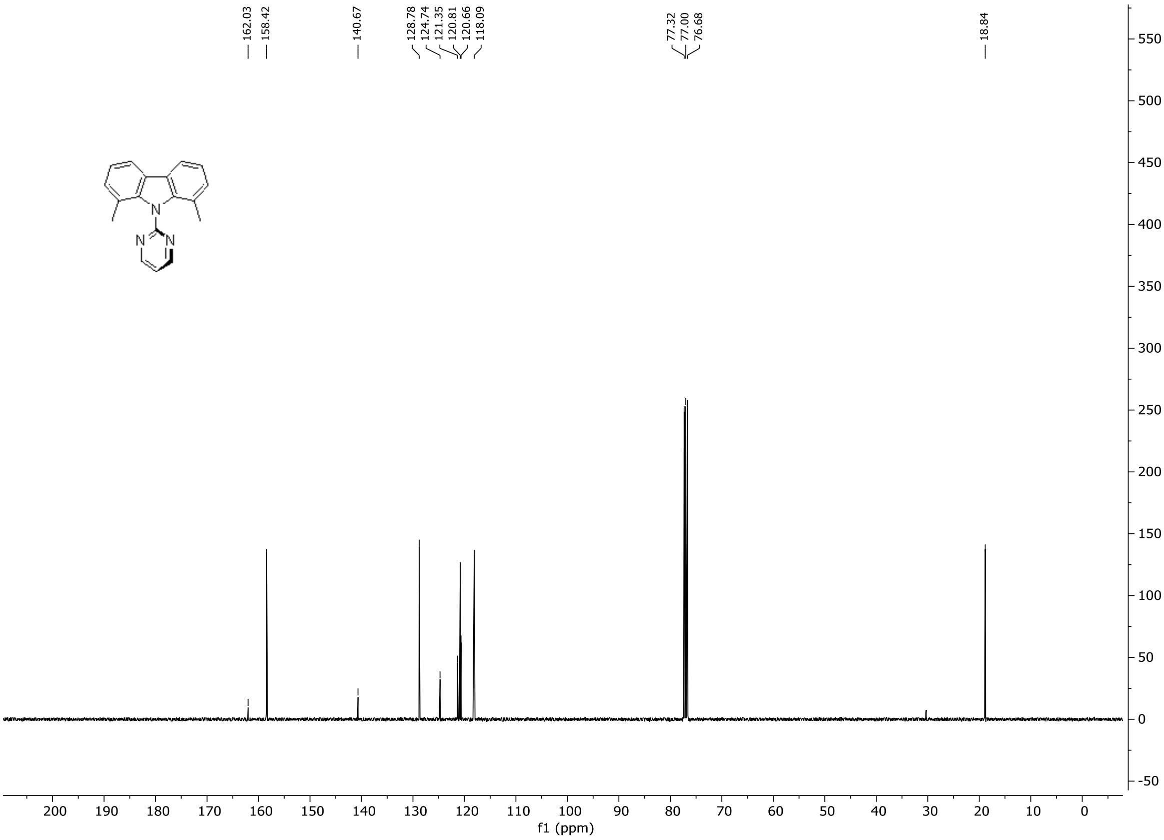
162.03
158.42
140.67
128.78
124.74
121.35
120.81
120.66
118.09
77.32
77.00
76.68
18.84
N
N
N
f1 (ppm)

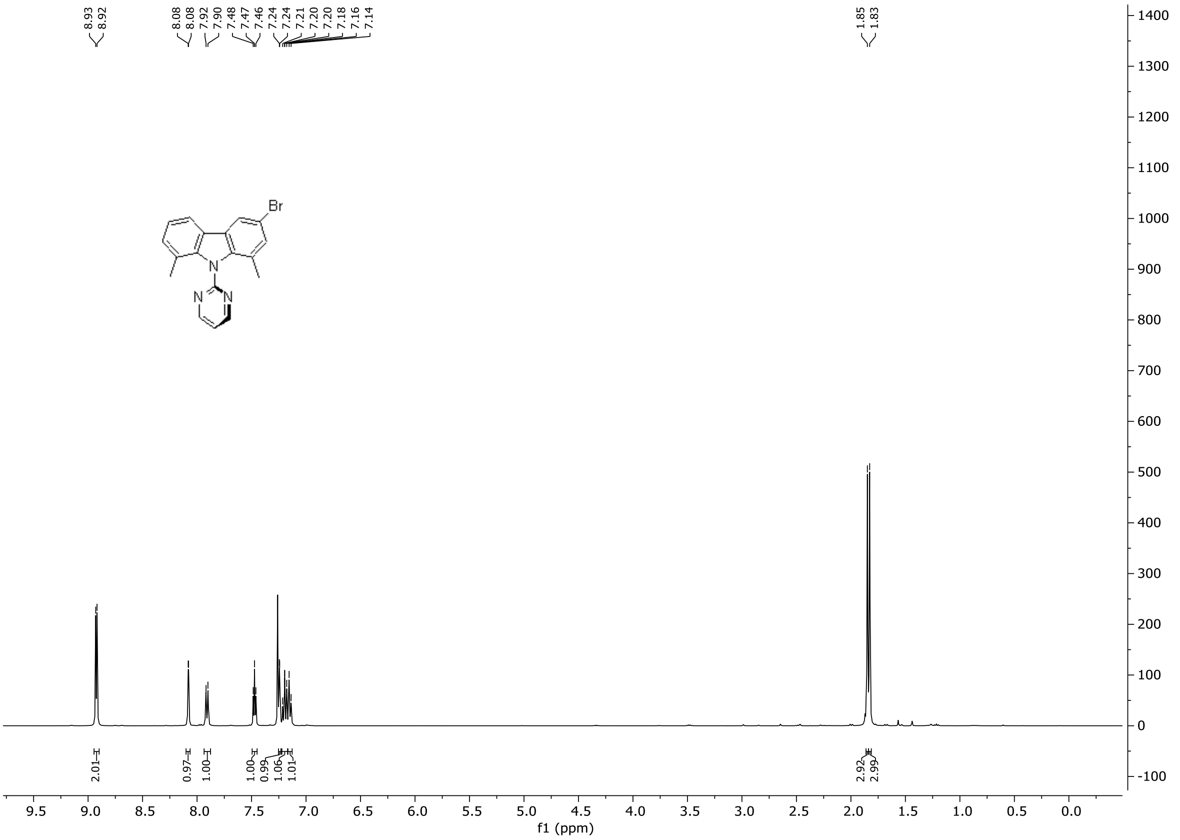
8.93
8.92
8.08
8.08
7.92
7.90
7.48
7.47
7.46
7.24
7.24
7.21
7.20
7.20
7.18
7.16
7.14
1.85
1.83
Br
N
N
N
2.01
0.97
1.00
1.00
0.99
1.06
1.01
2.92
2.99
f1 (ppm)

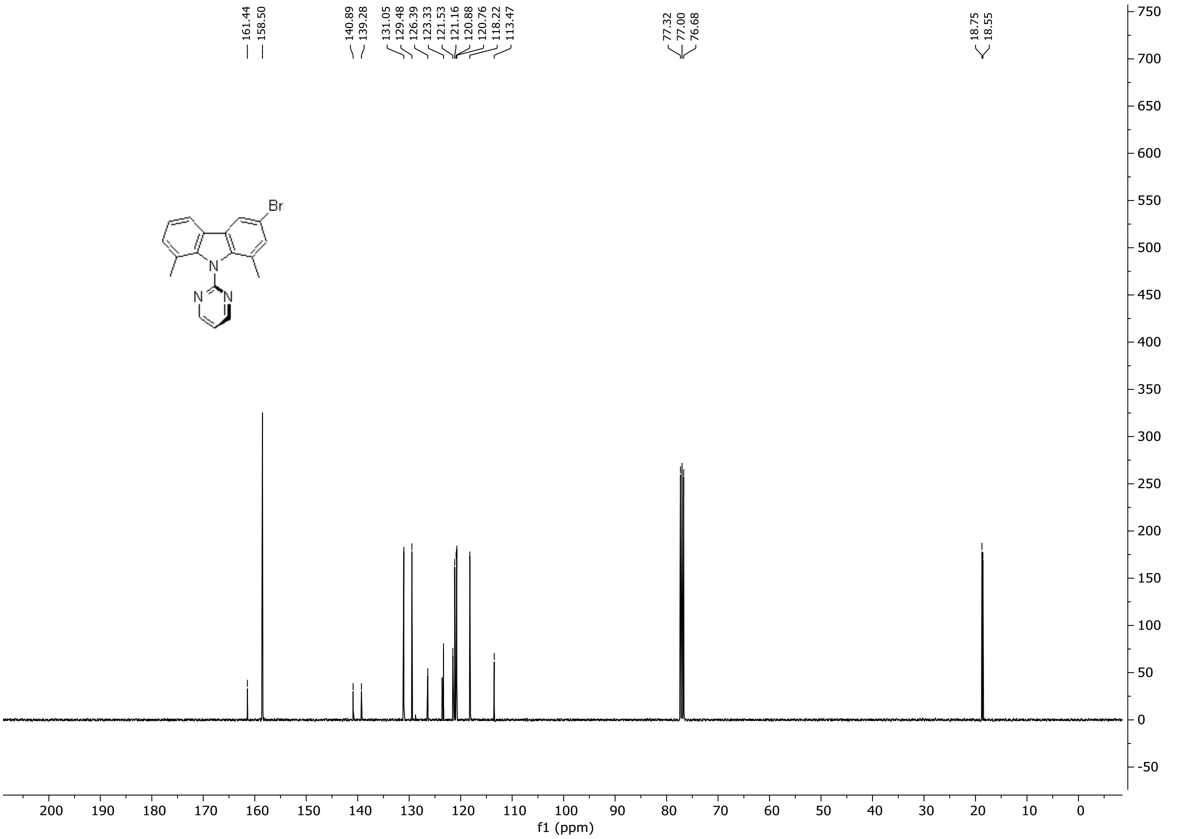
161.44
158.50
140.89
139.28
131.05
129.48
126.39
123.33
121.53
121.16
120.88
120.76
118.22
113.47
77.32
77.00
76.68
18.75
18.55
Br
N
N
N
f1 (ppm)

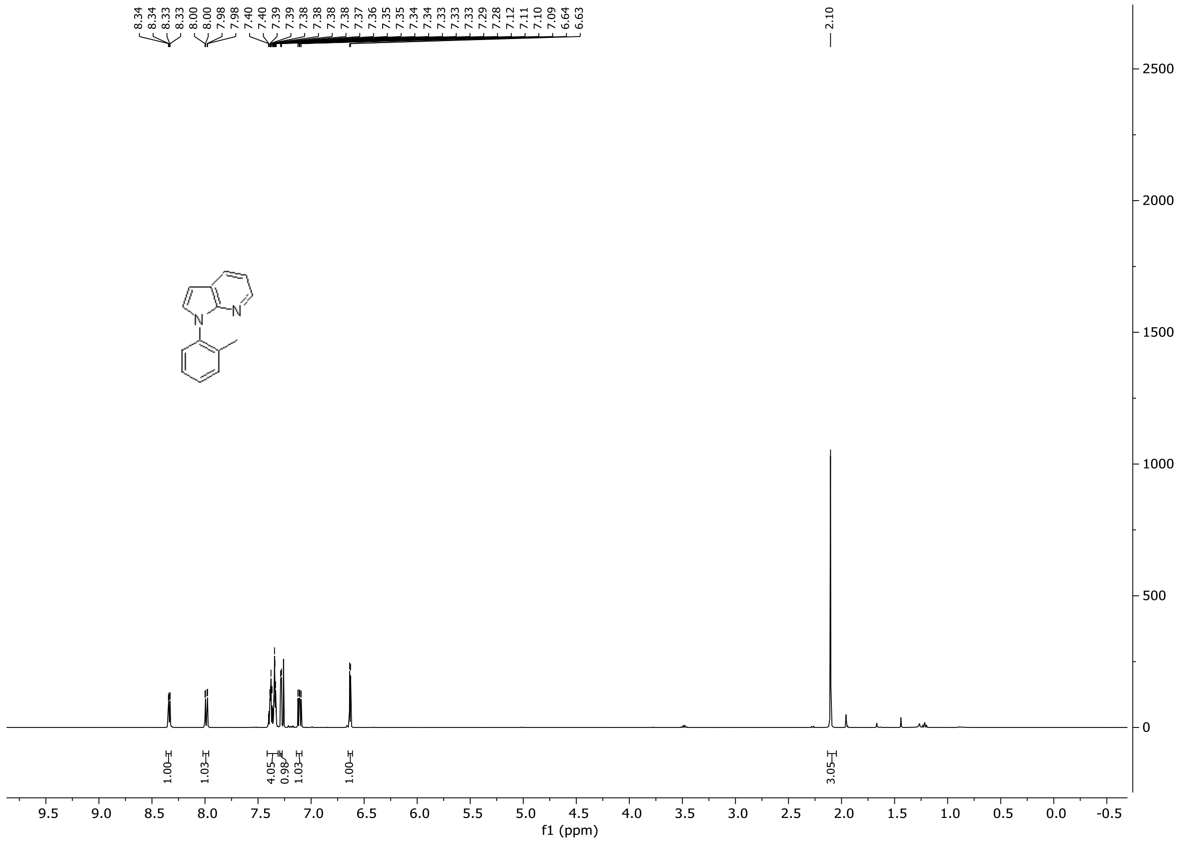
N
N
f1 (ppm)

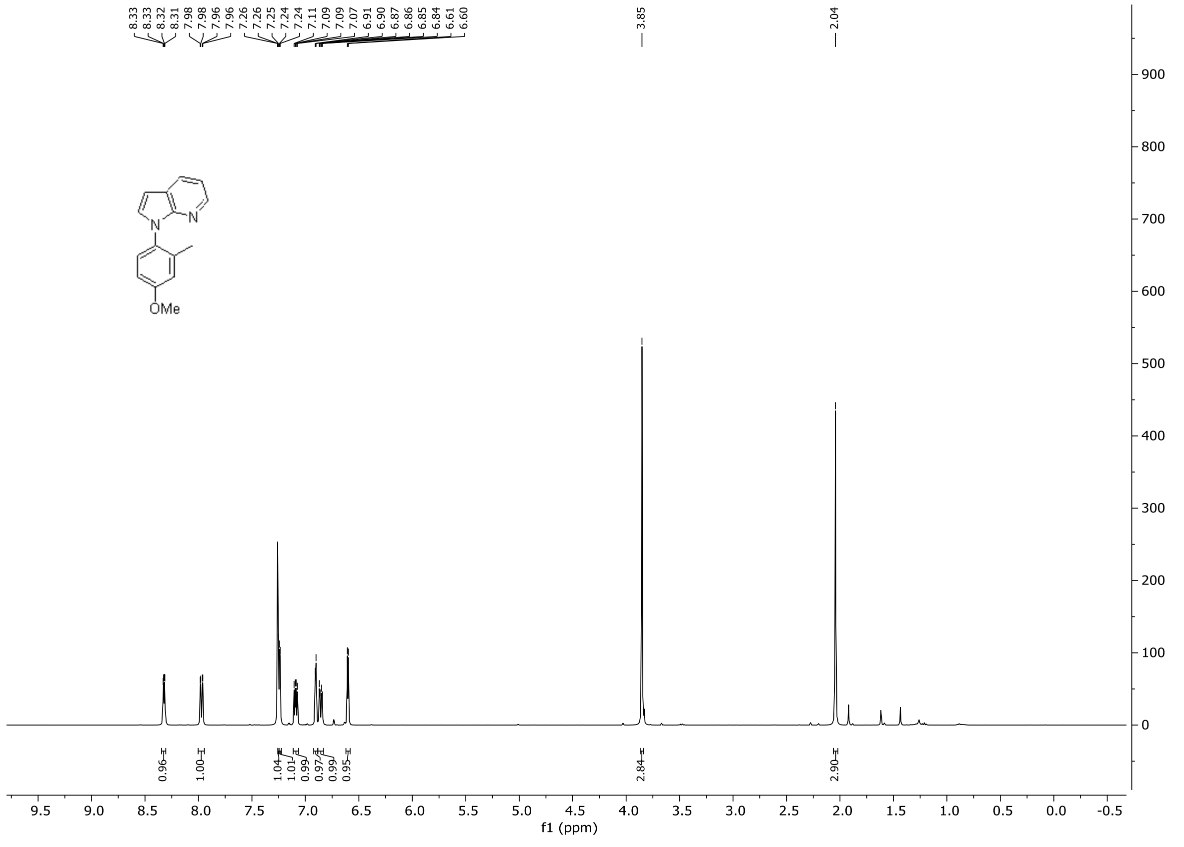
N
N
OMe
f1 (ppm)

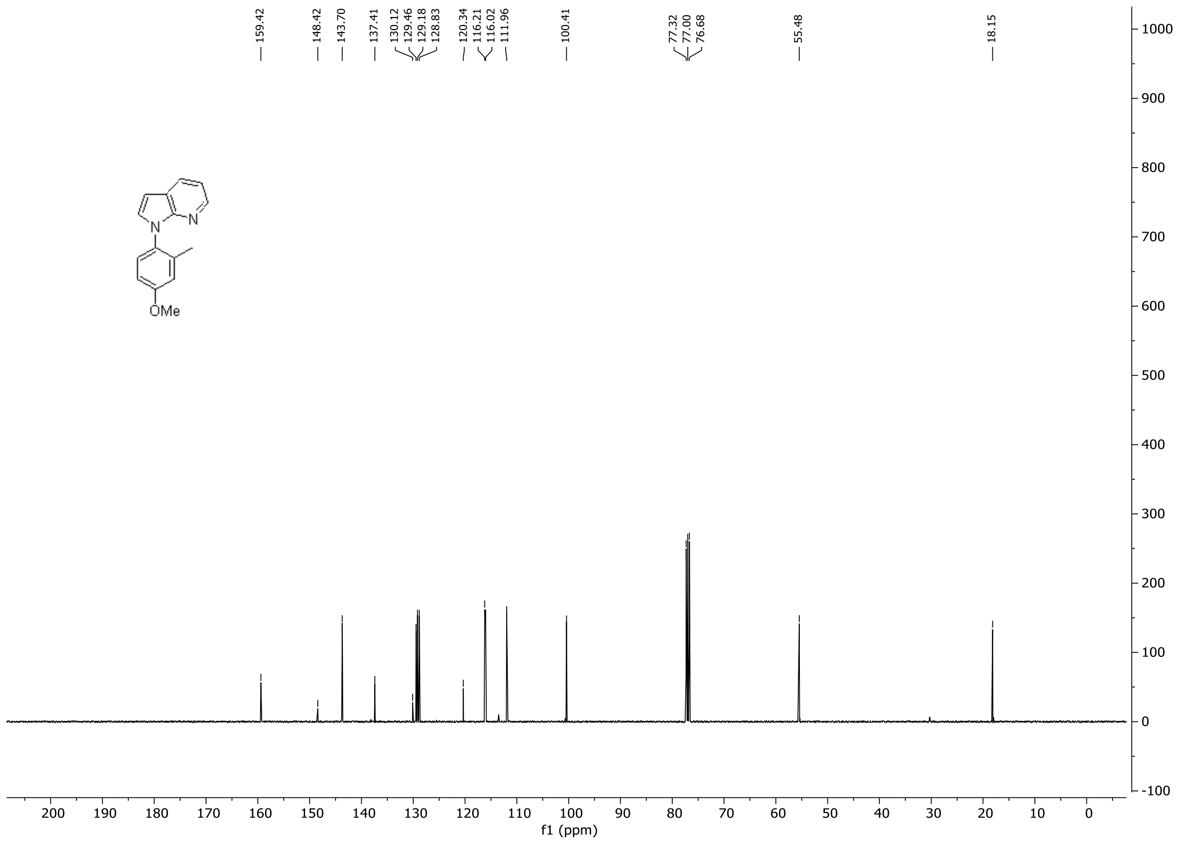
159.42
148.42
143.70
137.41
130.12
129.46
129.18
128.83
120.34
116.21
116.02
111.96
100.41
77.32
77.00
76.68
55.48
18.15
N
N
OMe
f1 (ppm)

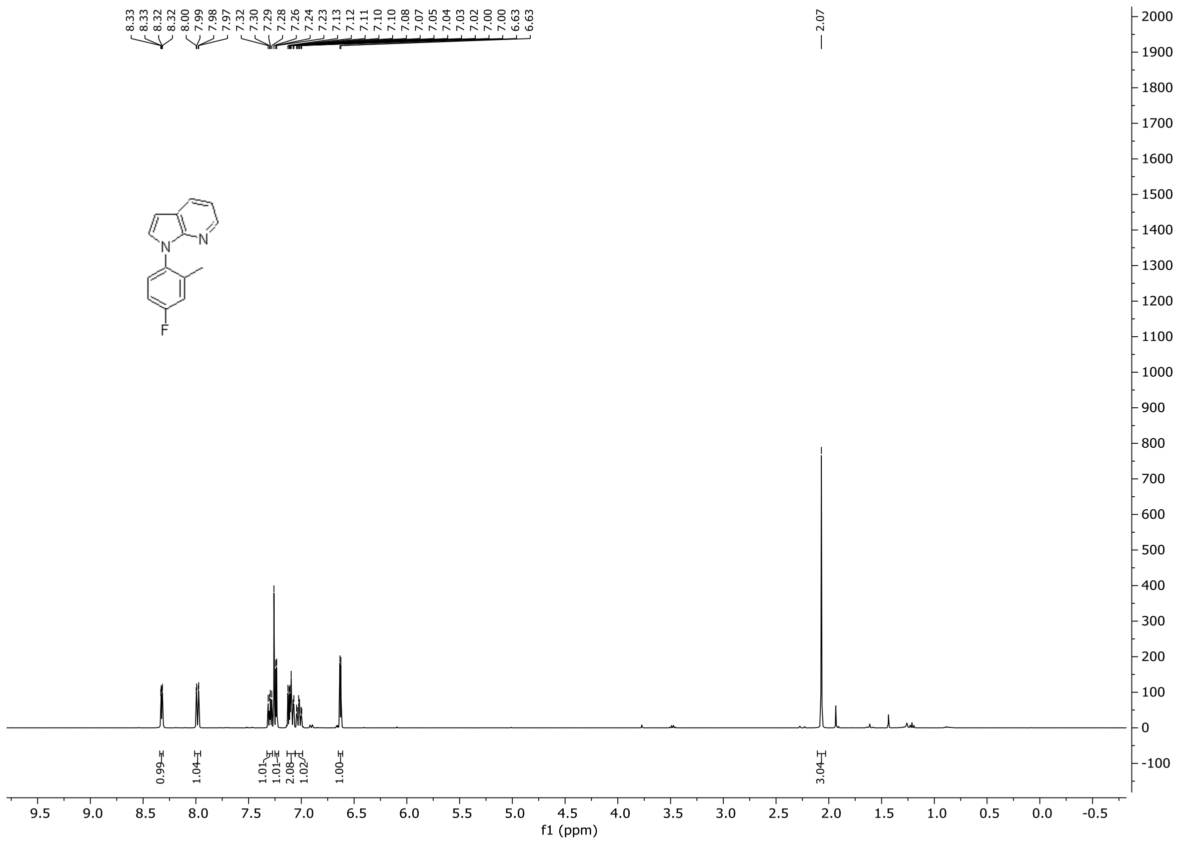
8.33
8.33
8.32
8.32
8.00
7.99
7.98
7.97
7.32
7.30
7.29
7.28
7.26
7.24
7.23
7.13
7.12
7.11
7.10
7.10
7.08
7.07
7.05
7.04
7.03
7.02
7.00
7.00
6.63
6.63
2.07
N
N
F
0.99
1.04
1.01
1.01
2.08
1.02
1.00
3.04
f1 (ppm)

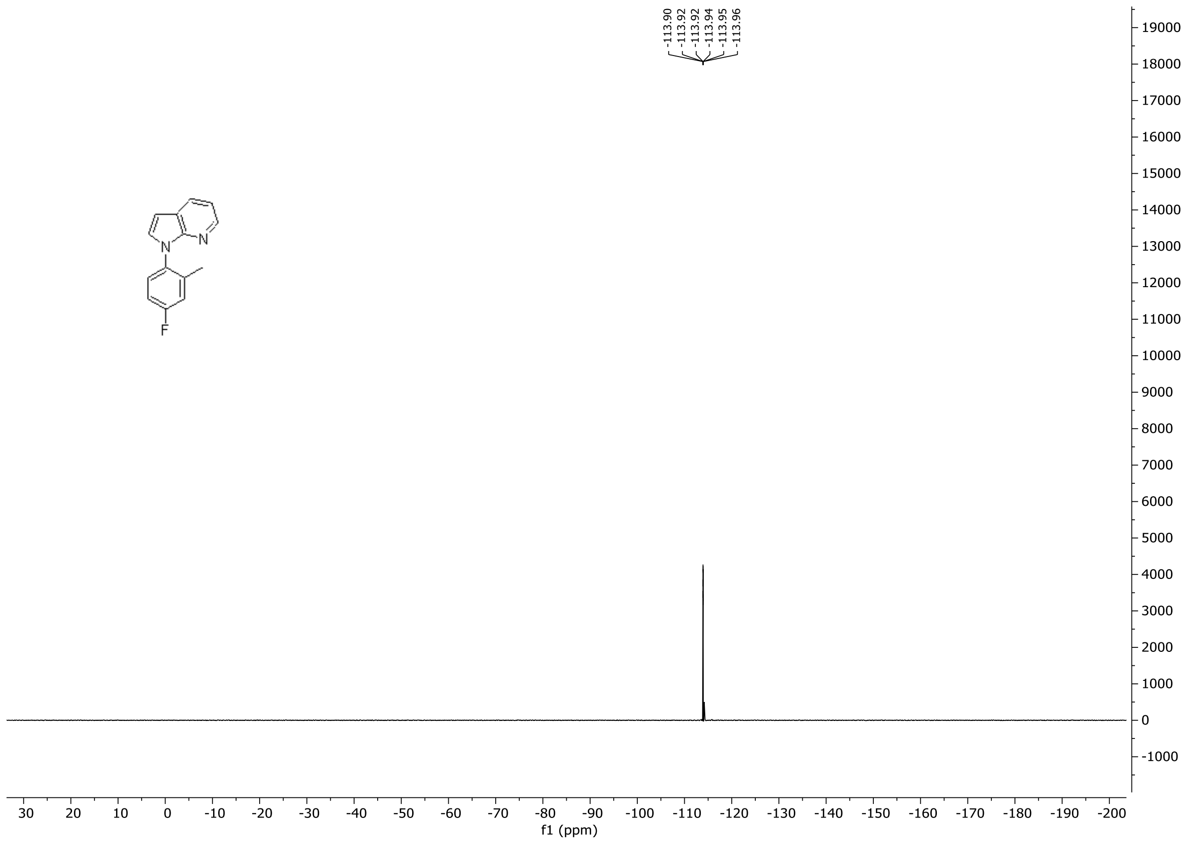
-113.90
-113.92
-113.92
-113.94
-113.95
-113.96
N
N
F
f1 (ppm)

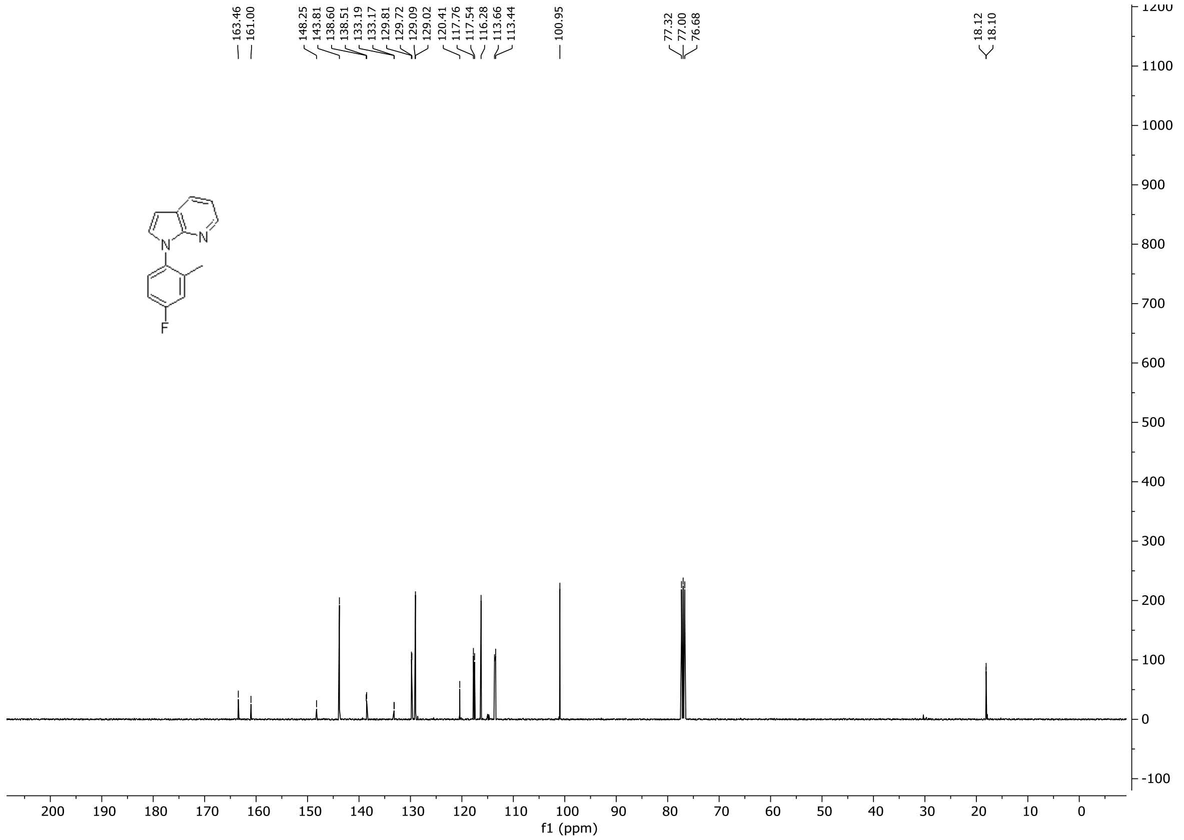
163.46
161.00
148.25
143.81
138.60
138.51
133.19
133.17
129.81
129.72
129.09
129.02
120.41
117.76
117.54
116.28
113.66
113.44
100.95
77.32
77.00
76.68
18.12
18.10
N
N
F
f1 (ppm)

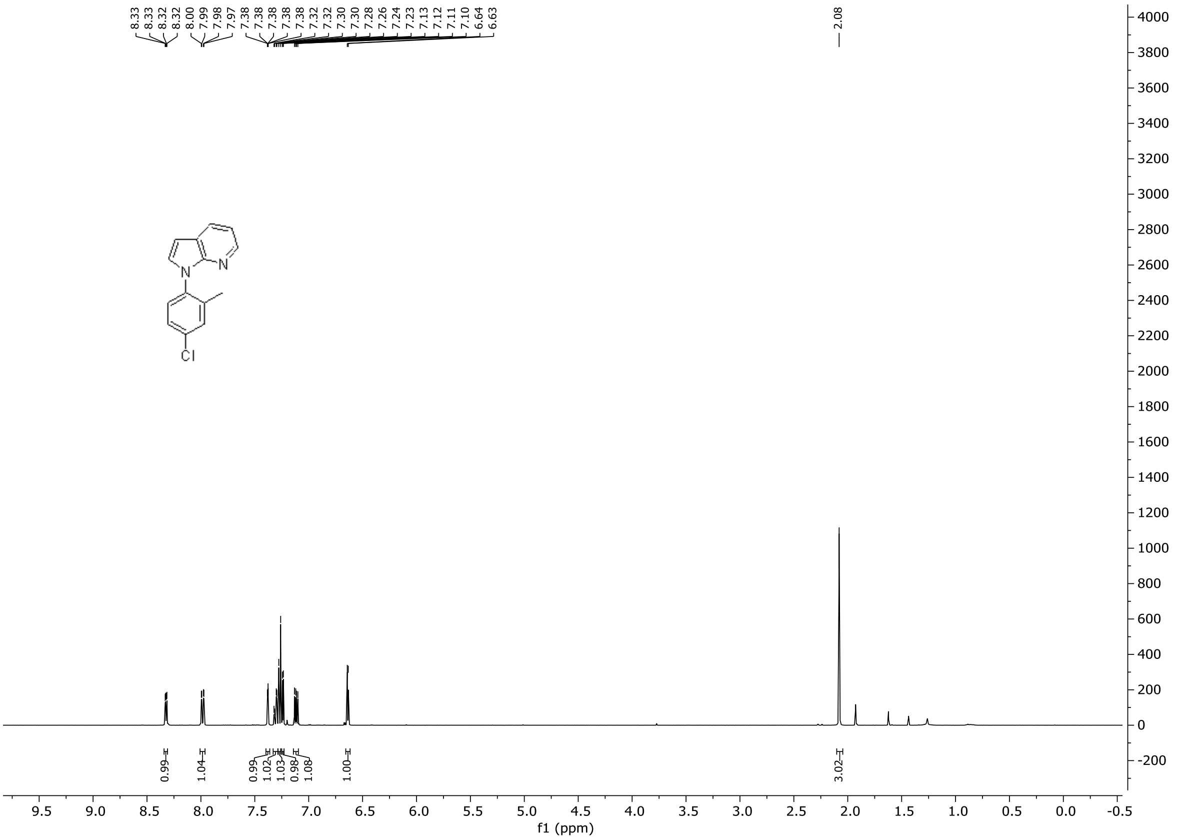
8.33
8.33
8.32
8.32
8.00
7.99
7.98
7.97
7.38
7.38
7.38
7.38
7.38
7.32
7.32
7.30
7.30
7.28
7.26
7.24
7.23
7.13
7.12
7.11
7.10
6.64
6.63
2.08
N
N
Cl
0.99
1.04
0.99
1.02
1.03
0.98
1.08
1.00
3.02
f1 (ppm)

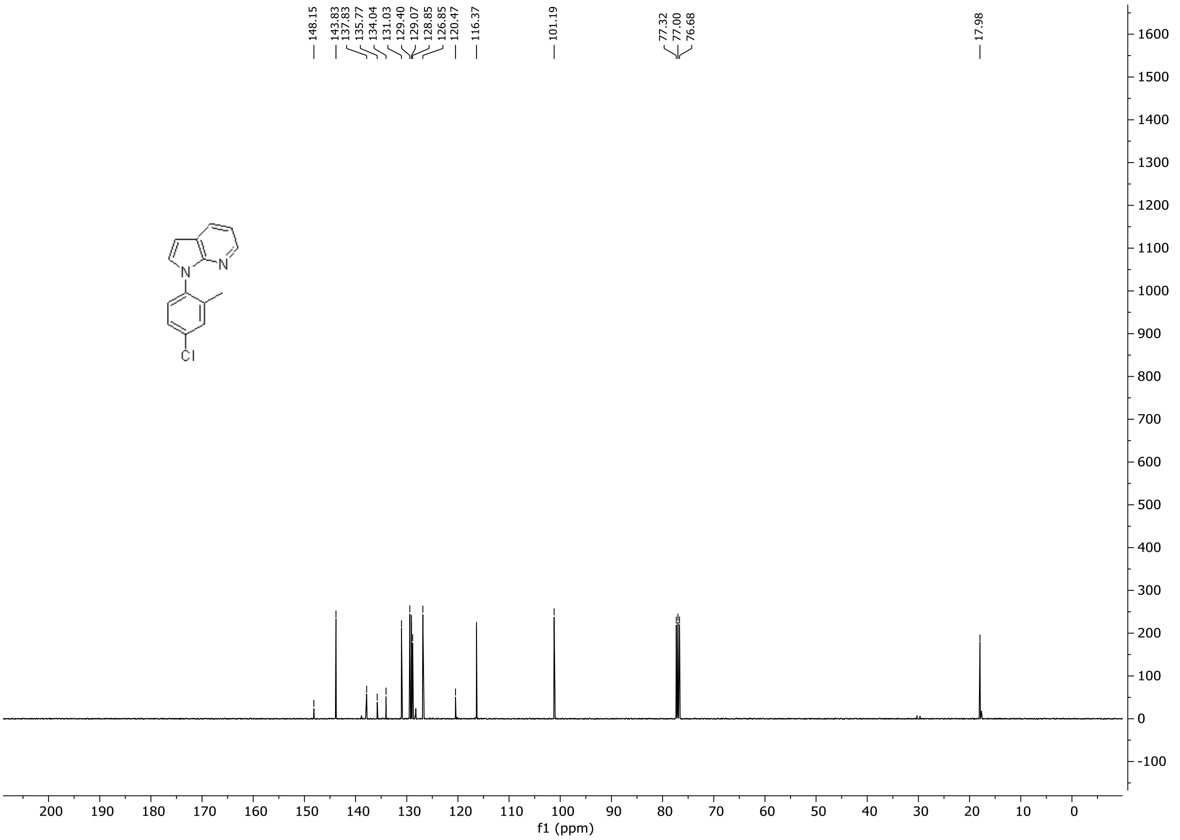
148.15
143.83
137.83
135.77
134.04
131.03
129.40
129.07
128.85
126.85
120.47
116.37
101.19
77.32
77.00
76.68
17.98
N
N
Cl
f1 (ppm)

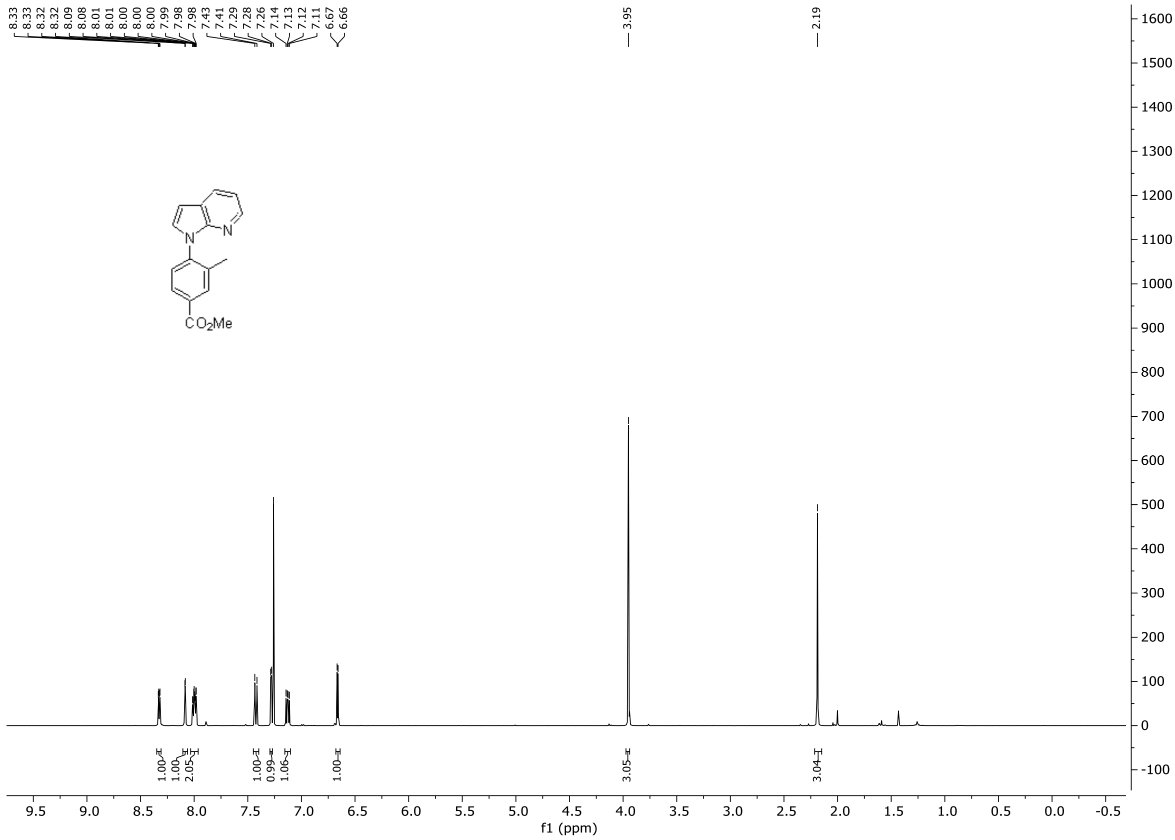
8.33 8.33 8.32 8.32 8.09 8.08 8.01 8.01 8.00 8.00 8.00 7.99 7.98 7.98 7.43 7.41 7.29 7.28 7.26 7.14 7.13 7.12 7.11 6.67 6.66
3.95
2.19
N
N
CO2Me
1.00 1.00 2.05 1.00 0.99 1.06 1.00 3.05 3.04
f1 (ppm)

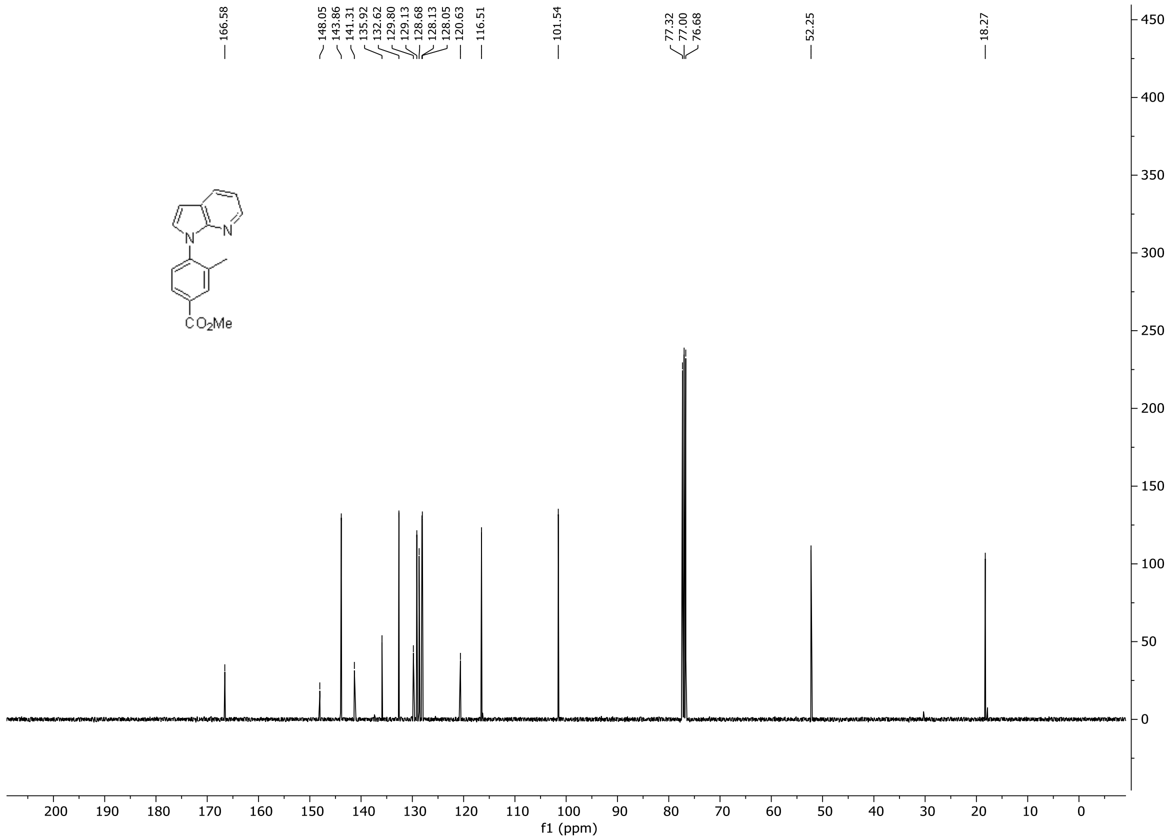
166.58
148.05 143.86 141.31 135.92 132.62 129.80 129.13 128.68 128.13 128.05 120.63 116.51
101.54
77.32 77.00 76.68
52.25
18.27
N
N
CO2Me
f1 (ppm)

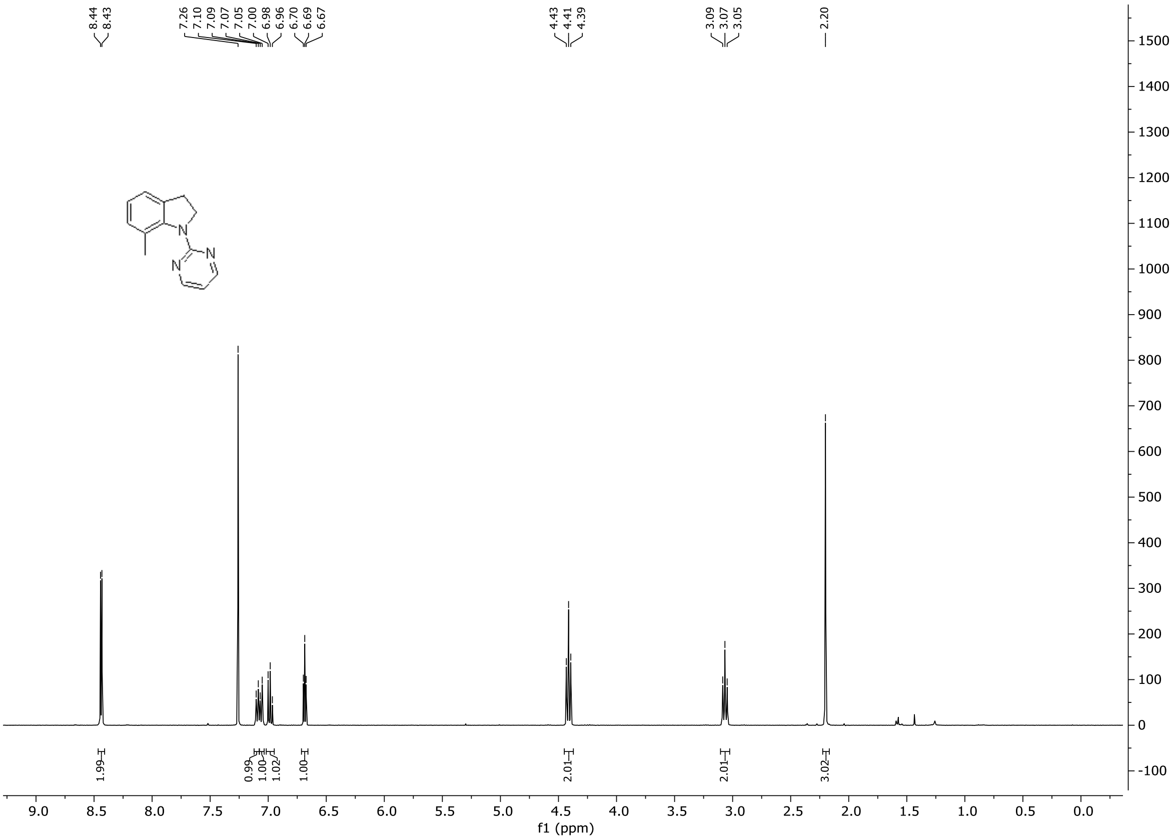
8.44
8.43
7.26
7.10
7.09
7.07
7.05
7.00
6.98
6.96
6.70
6.69
6.67
4.43
4.41
4.39
3.09
3.07
3.05
2.20
1.99
0.99
1.00
1.02
1.00
2.01
2.01
3.02
f1 (ppm)

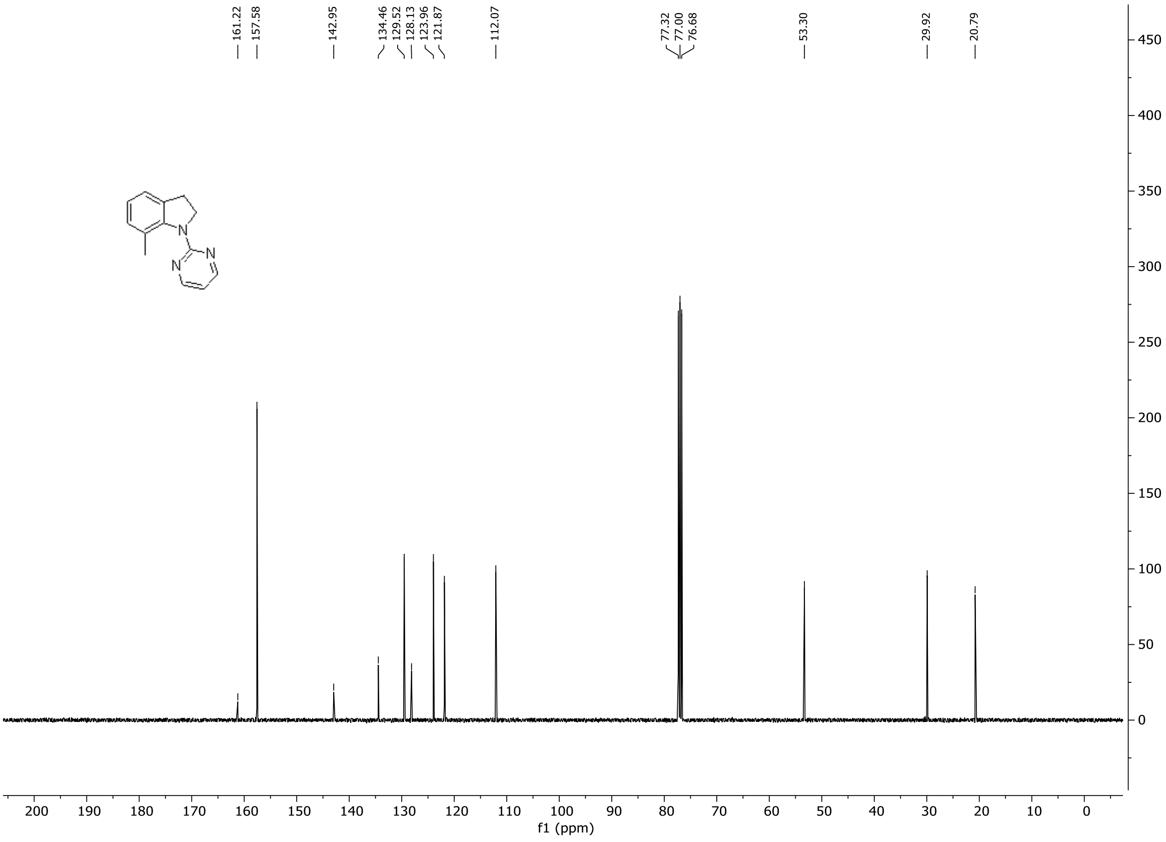
161.22
157.58
142.95
134.46
129.52
128.13
123.96
121.87
112.07
77.32
77.00
76.68
53.30
29.92
20.79
f1 (ppm)

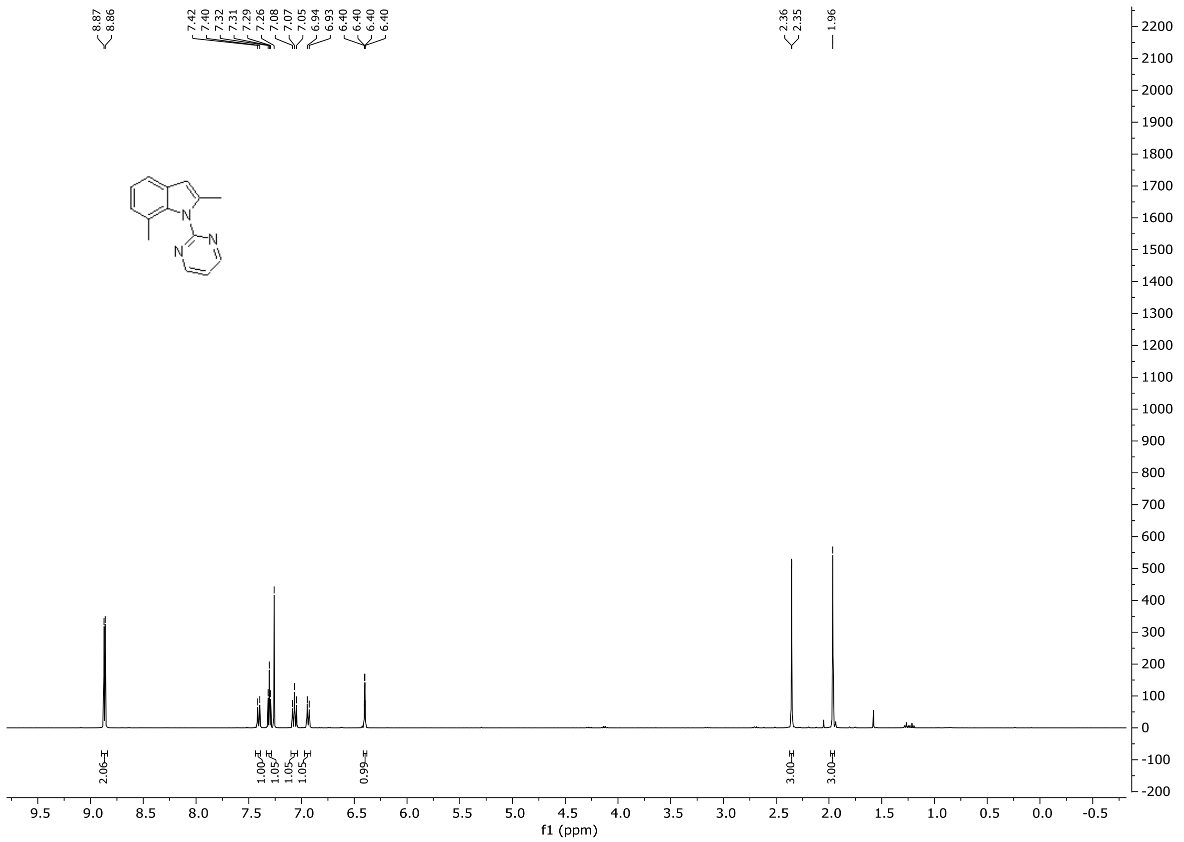
8.87
8.86
7.42
7.40
7.32
7.31
7.29
7.26
7.08
7.07
7.05
6.94
6.93
6.40
6.40
6.40
6.40
2.36
2.35
1.96
2.06
1.00
1.05
1.05
1.05
0.99
3.00
3.00
f1 (ppm)

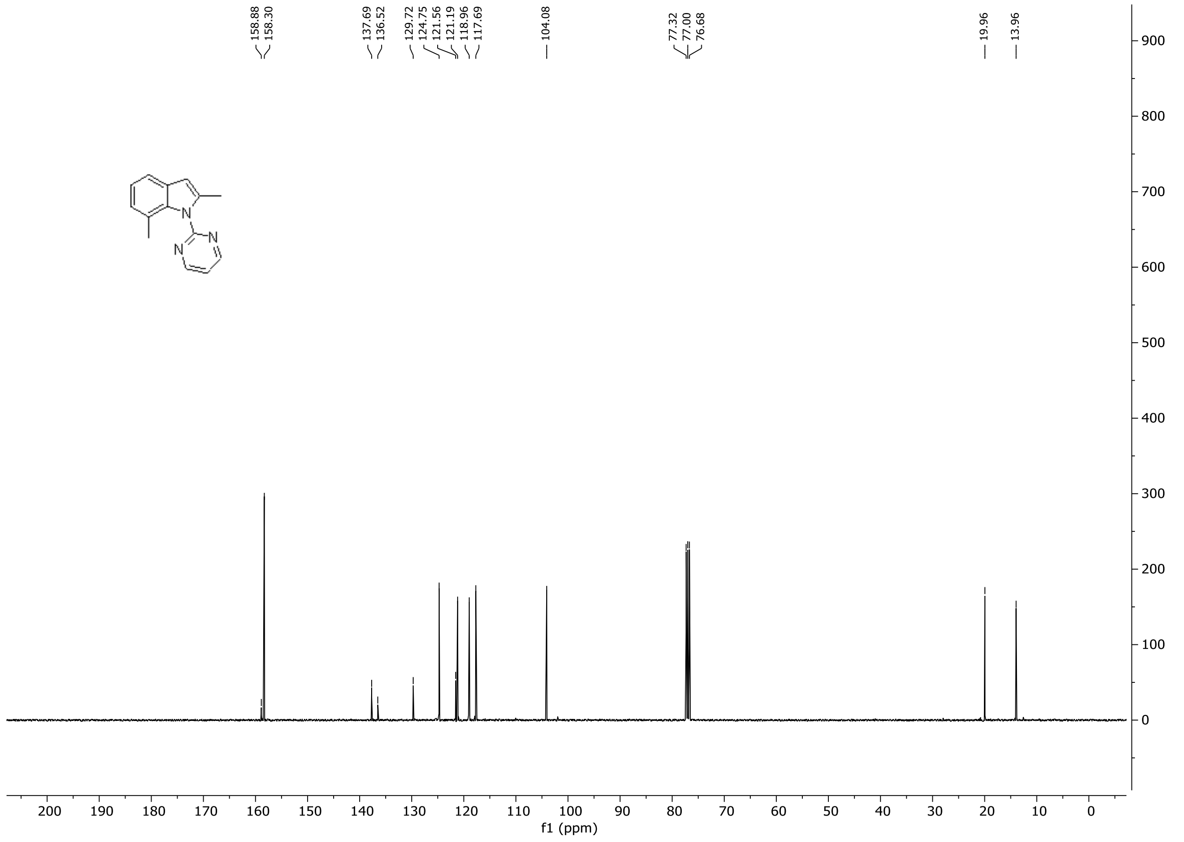
158.88
158.30
137.69
136.52
129.72
124.75
121.56
121.19
118.96
117.69
104.08
77.32
77.00
76.68
19.96
13.96
f1 (ppm)

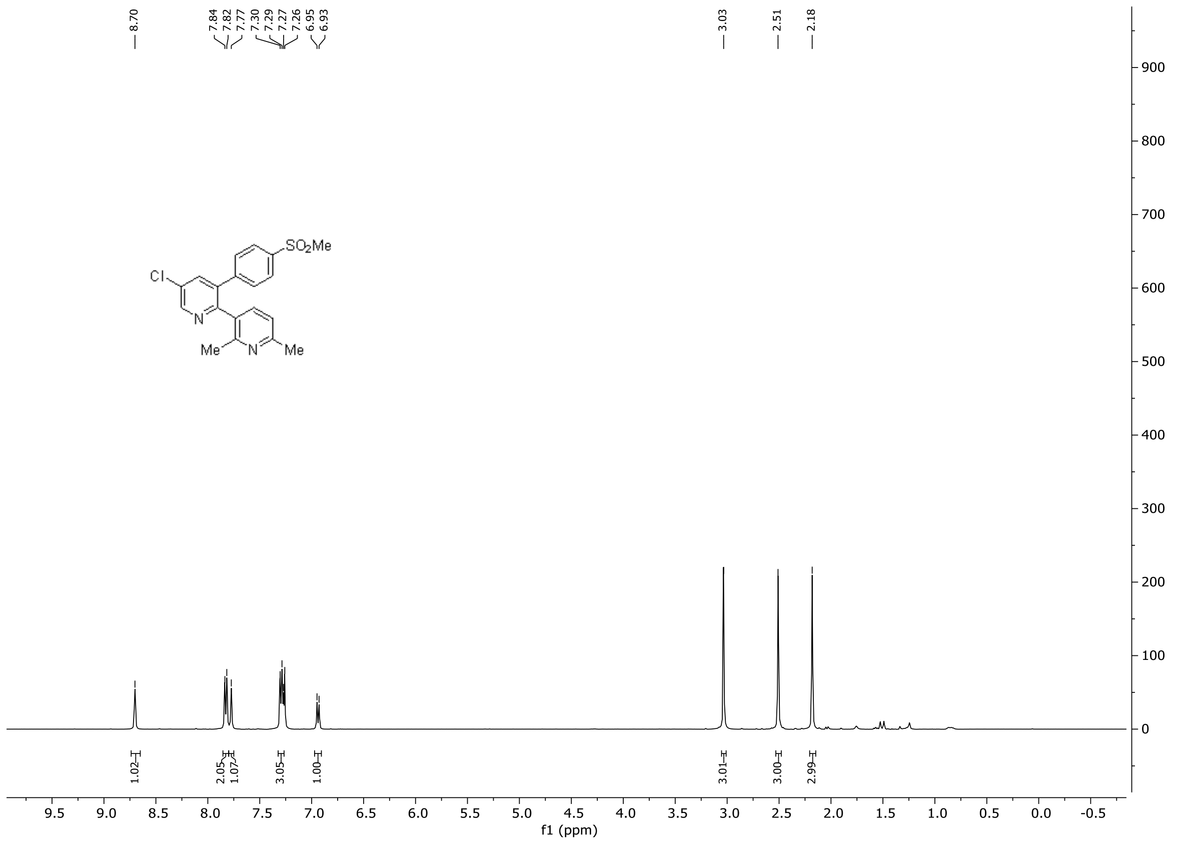
8.70
7.84
7.82
7.77
7.30
7.29
7.27
7.26
6.95
6.93
3.03
2.51
2.18
Cl
N
SO2Me
Me
N
Me
1.02
2.05
1.07
3.05
1.00
3.01
3.00
2.99
f1 (ppm)

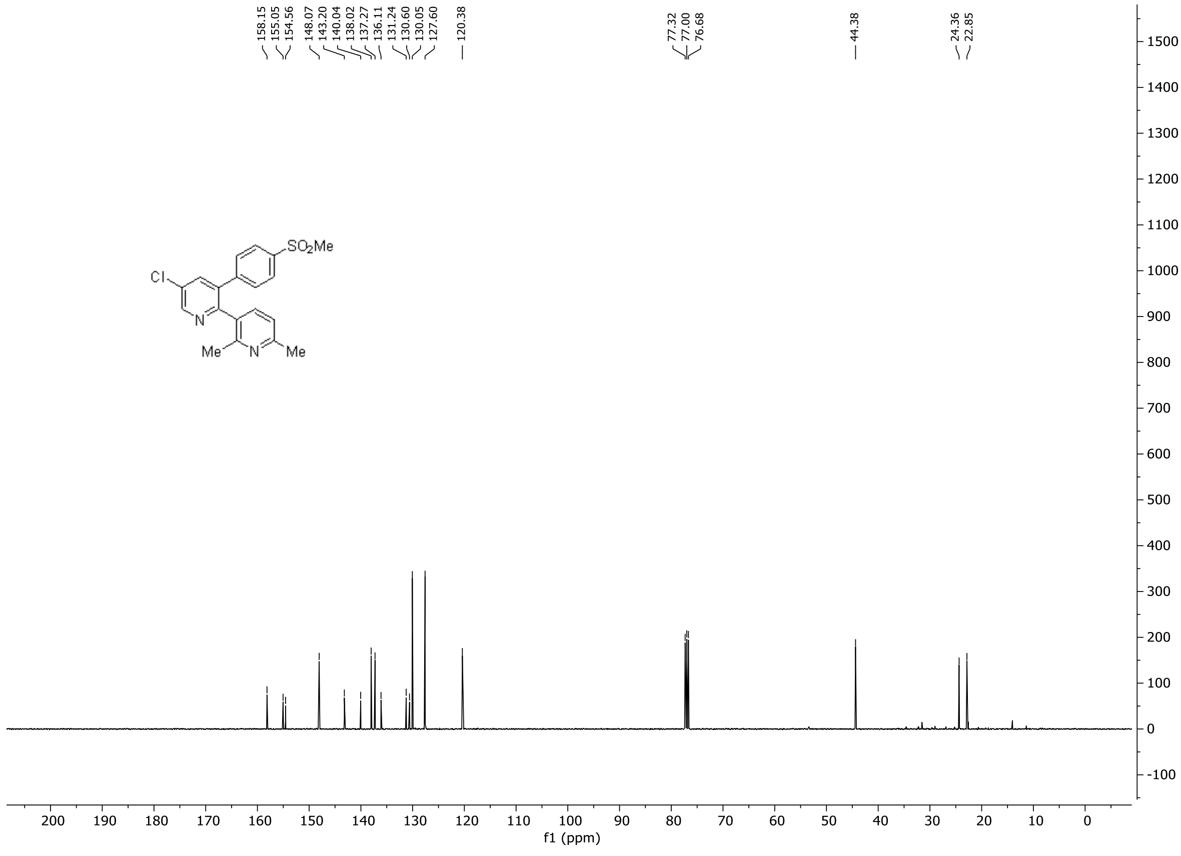
158.15
155.05
154.56
148.07
143.20
140.04
138.02
137.27
136.11
131.24
130.60
130.05
127.60
120.38
77.32
77.00
76.68
44.38
24.36
22.85
Cl
N
SO2Me
Me
N
Me
f1 (ppm)

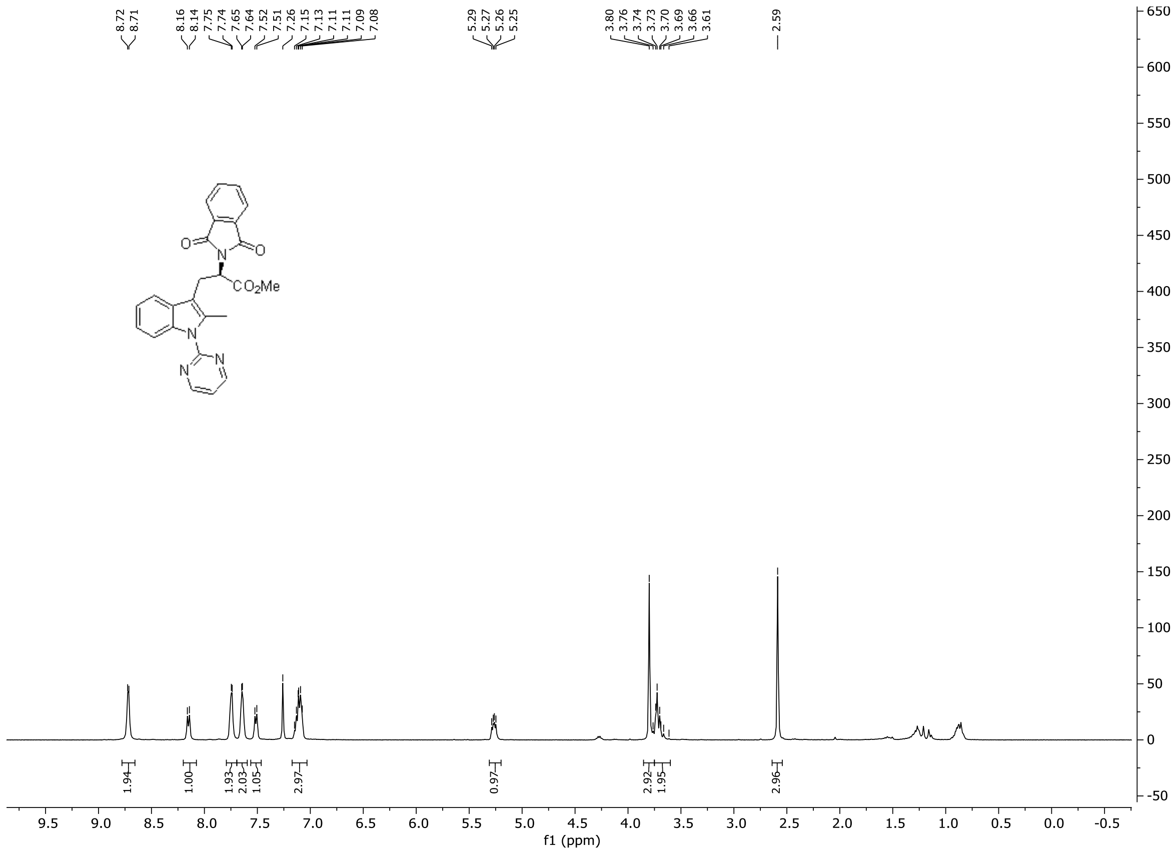
8.72
8.71
8.16
8.14
7.75
7.74
7.65
7.64
7.52
7.51
7.26
7.15
7.13
7.11
7.11
7.09
7.08
5.29
5.27
5.26
5.25
3.80
3.76
3.74
3.73
3.70
3.69
3.66
3.61
2.59
CO2Me
1.94
1.00
1.93
2.03
1.05
2.97
0.97
2.92
1.95
2.96
f1 (ppm)

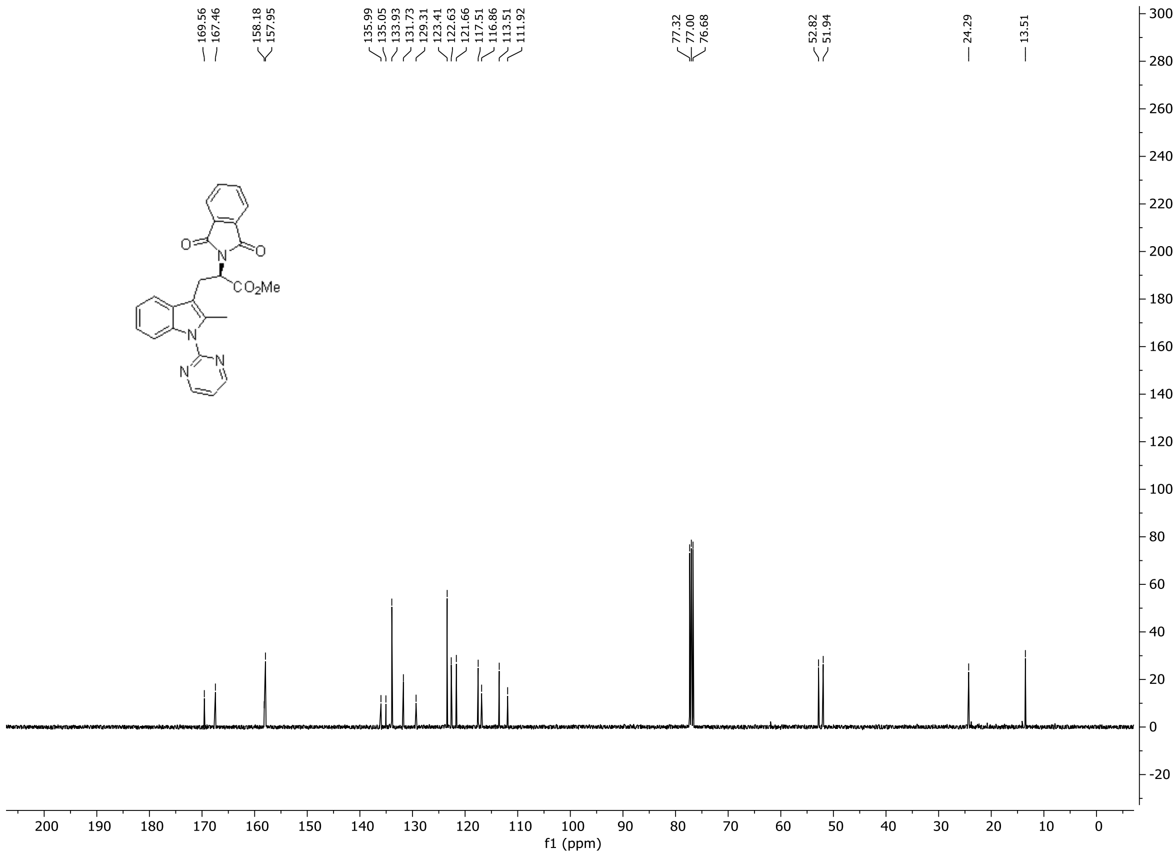
169.56
167.46
158.18
157.95
135.99
135.05
133.93
131.73
129.31
123.41
122.63
121.66
117.51
116.86
113.51
111.92
77.32
77.00
76.68
52.82
51.94
24.29
13.51
CO2Me
f1 (ppm)

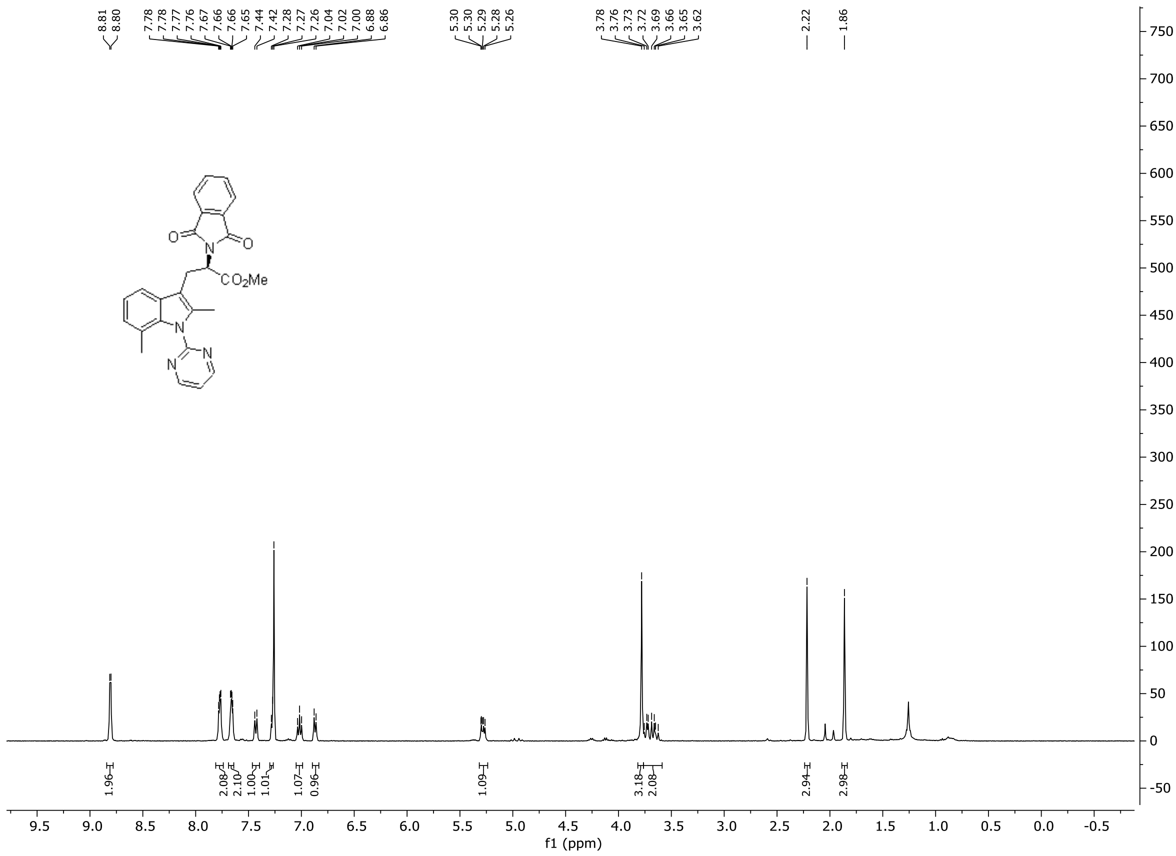
8.81
8.80
7.78
7.78
7.77
7.76
7.67
7.66
7.66
7.65
7.44
7.42
7.28
7.27
7.26
7.04
7.02
7.00
6.88
6.86
5.30
5.30
5.29
5.28
5.26
3.78
3.76
3.73
3.72
3.69
3.66
3.65
3.62
2.22
1.86
O
N
O
CO2Me
N
N
N
1.96
2.08
2.10
1.00
1.01
1.07
0.96
1.09
3.18
2.08
2.94
2.98
f1 (ppm)

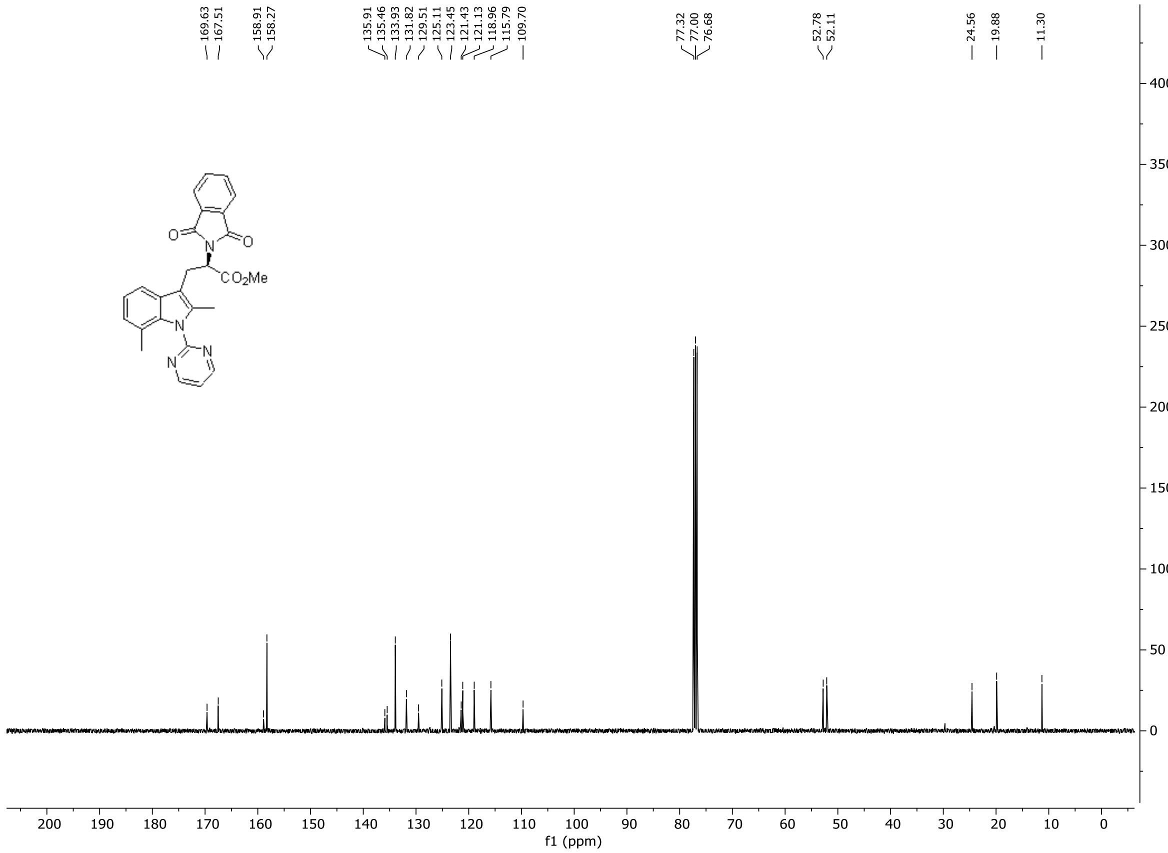
169.63
167.51
158.91
158.27
135.91
135.46
133.93
131.82
129.51
125.11
123.45
121.43
121.13
118.96
115.79
109.70
77.32
77.00
76.68
52.78
52.11
24.56
19.88
11.30
O
N
O
CO2Me
N
N
N
f1 (ppm)

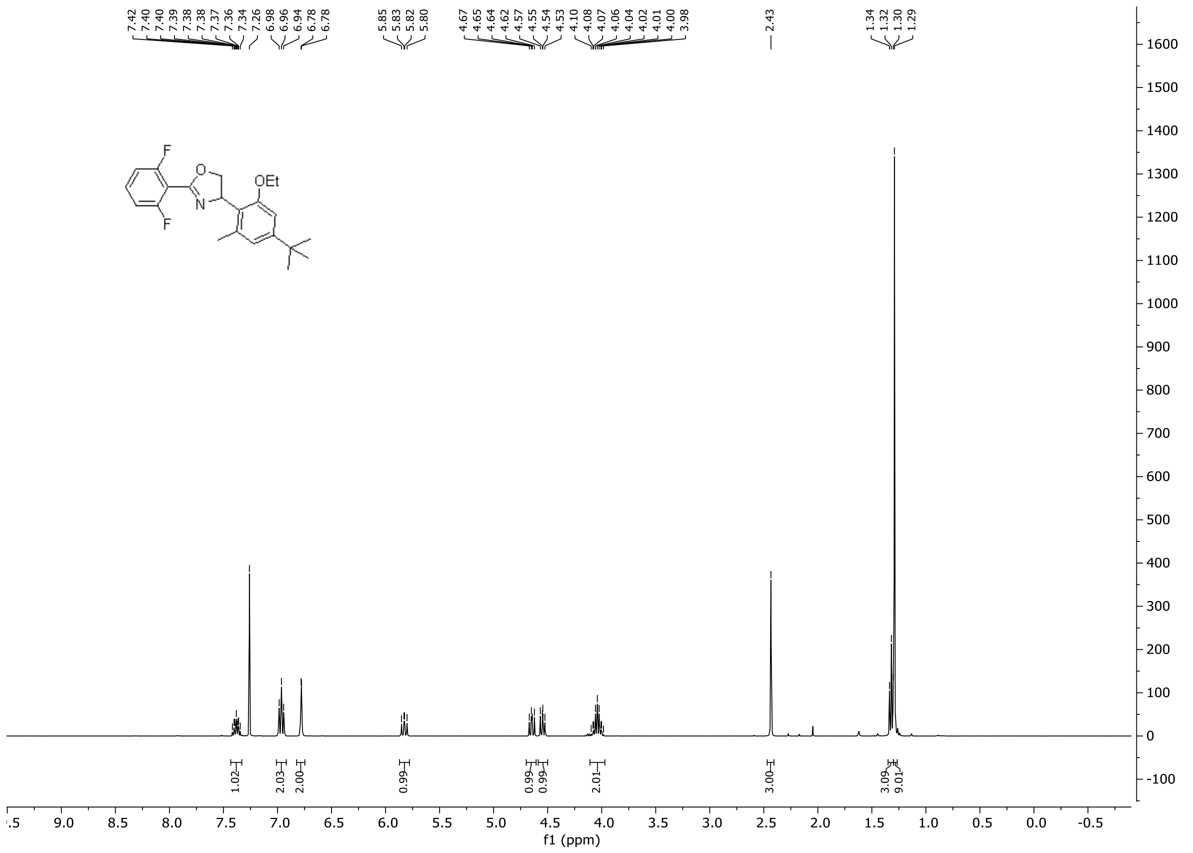
7.42 7.40 7.40 7.39 7.38 7.38 7.37 7.36 7.34 7.26 6.98 6.96 6.94 6.78 6.78
5.85 5.83 5.82 5.80
4.67 4.65 4.64 4.62 4.57 4.55 4.54 4.53 4.10 4.08 4.07 4.06 4.04 4.02 4.01 4.00 3.98
2.43
1.34 1.32 1.30 1.29
F
O
N
OEt
1.02
2.03
2.00
0.99
0.99
0.99
2.01
3.00
3.09
9.01
f1 (ppm)

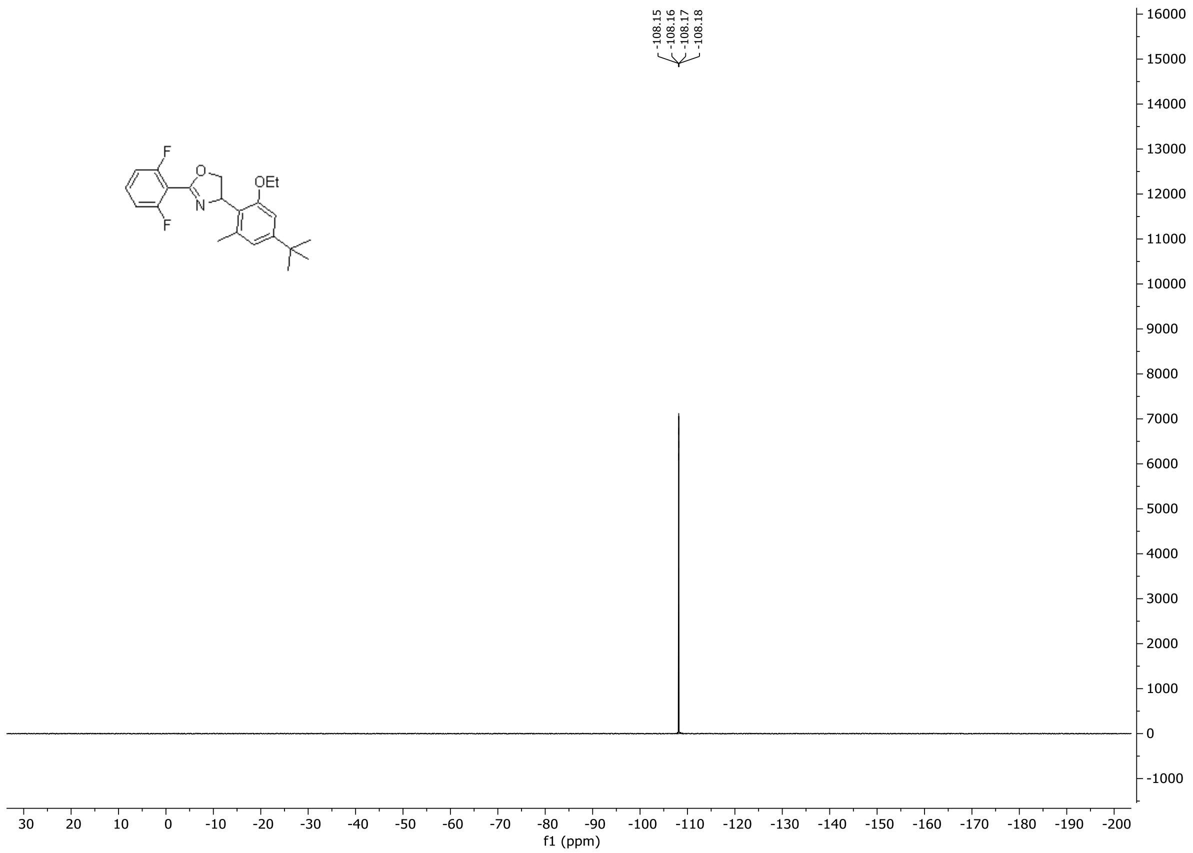
-108.15
-108.16
-108.17
-108.18
F
O
N
OEt
f1 (ppm)

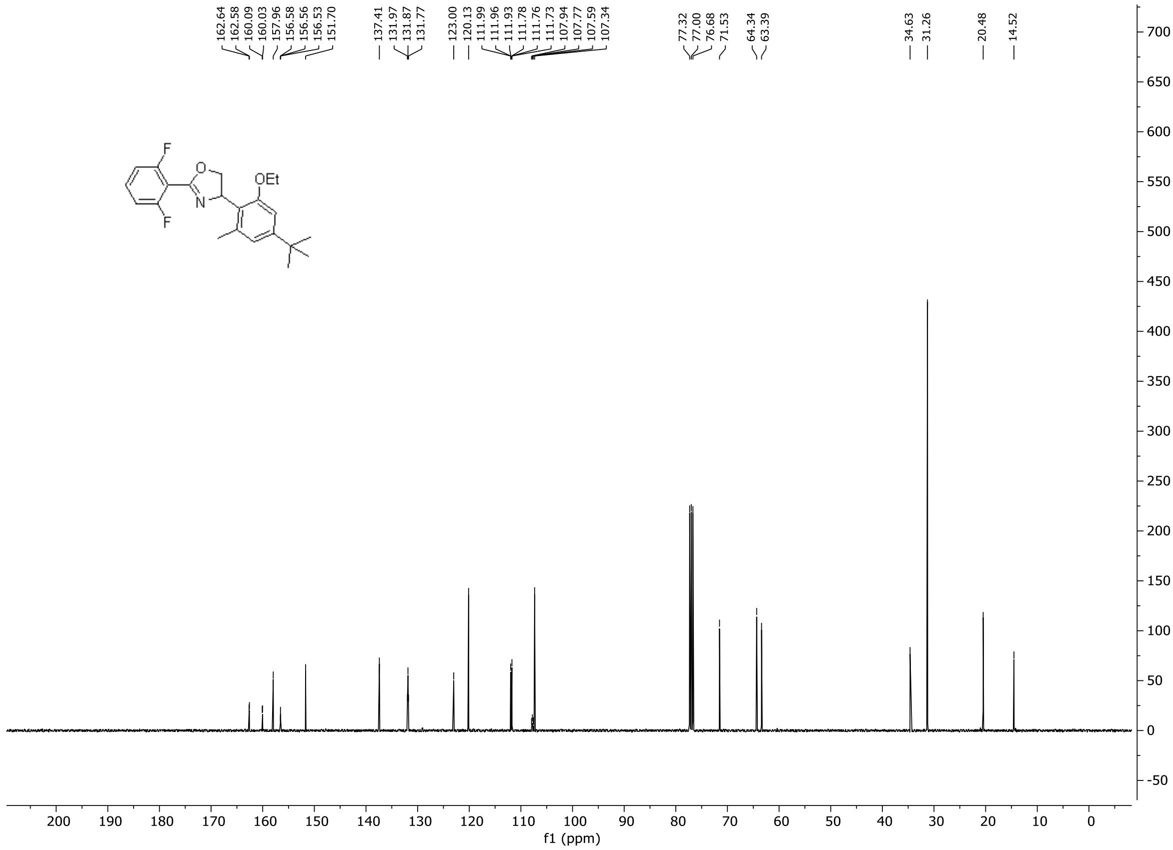
162.64
162.58
160.09
160.03
157.96
156.58
156.56
156.53
151.70
137.41
131.97
131.87
131.77
123.00
120.13
111.99
111.96
111.93
111.78
111.76
111.73
107.94
107.77
107.59
107.34
77.32
77.00
76.68
71.53
64.34
63.39
34.63
31.26
20.48
14.52
F
O
N
OEt
f1 (ppm)

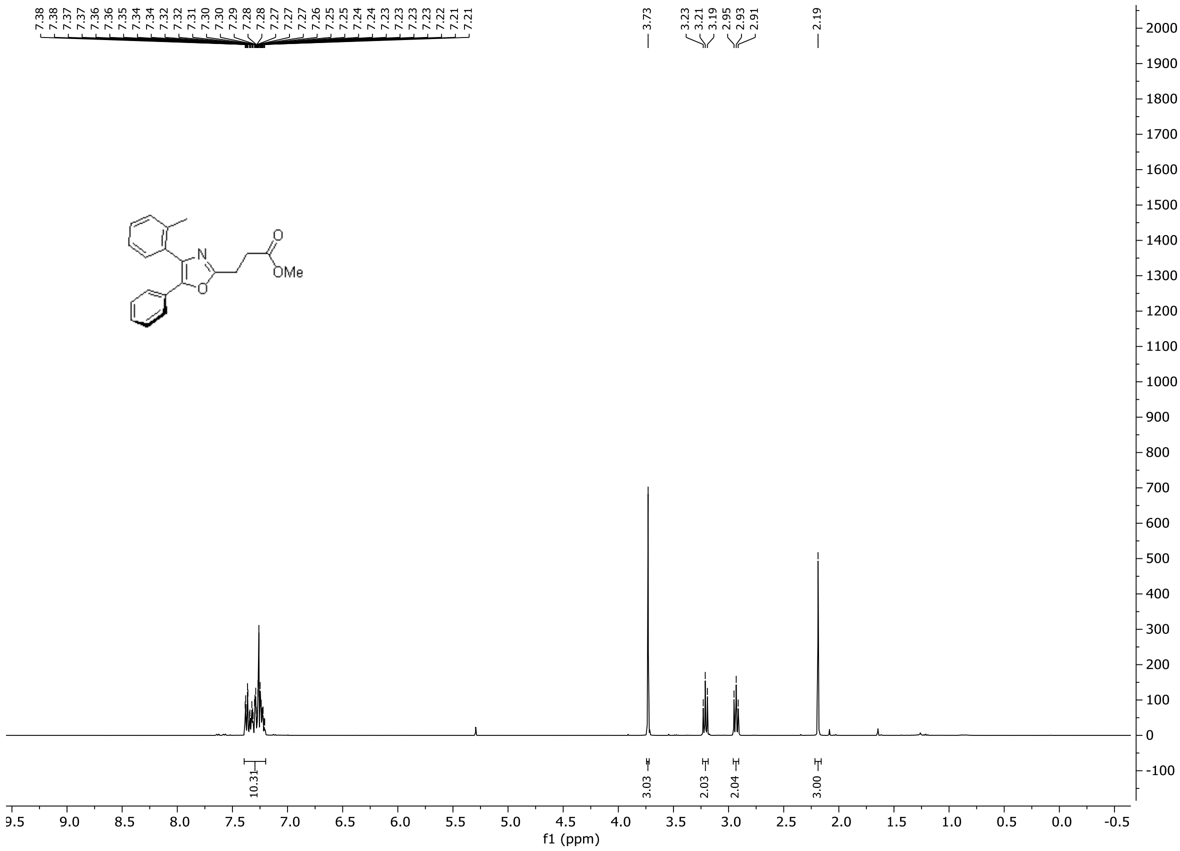
7.38
7.38
7.37
7.37
7.36
7.36
7.35
7.34
7.34
7.32
7.32
7.31
7.30
7.30
7.29
7.28
7.28
7.27
7.27
7.26
7.25
7.25
7.24
7.24
7.23
7.23
7.23
7.23
7.22
7.21
7.21
3.73
3.23
3.21
3.19
2.95
2.93
2.91
2.19
N
O
OMe
10.31
3.03
2.03
2.04
3.00
f1 (ppm)

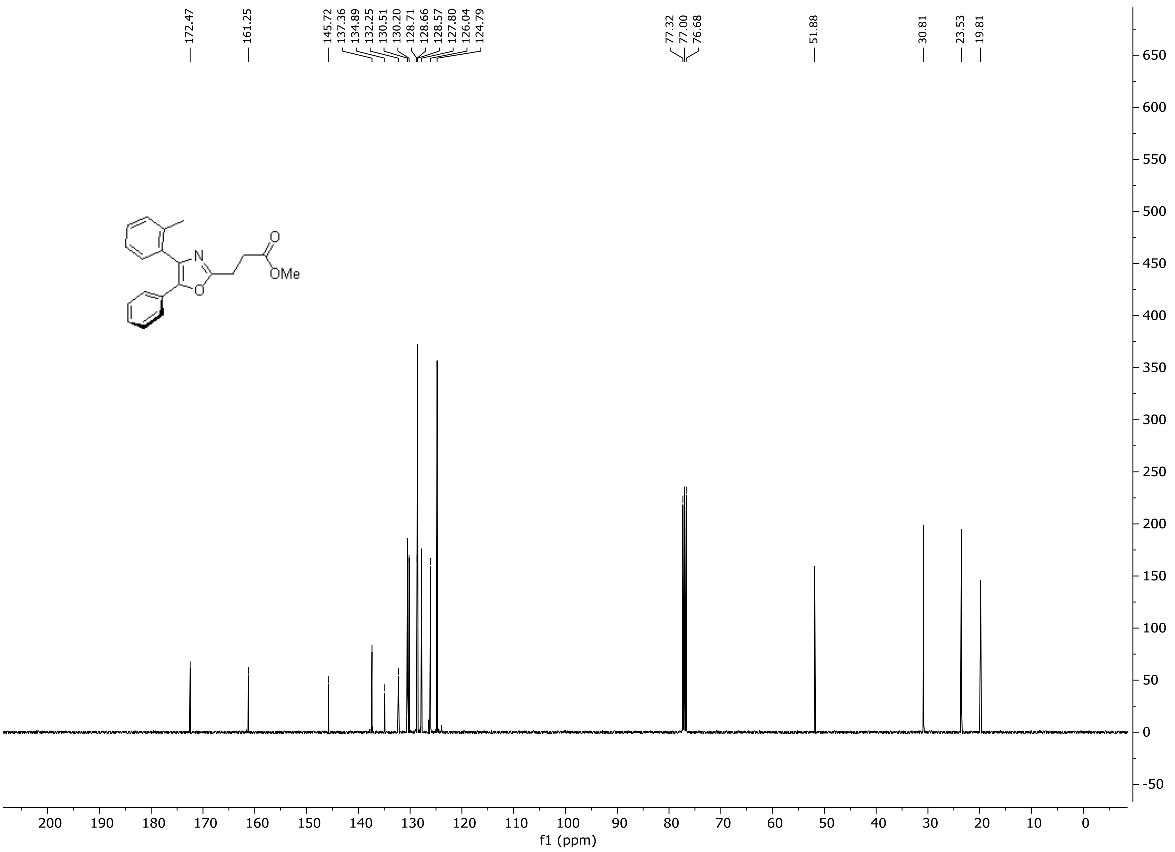
172.47
161.25
145.72
137.36
134.89
132.25
130.51
130.20
128.71
128.66
128.57
127.80
126.04
124.79
77.32
77.00
76.68
51.88
30.81
23.53
19.81
N
O
O
OMe
f1 (ppm)

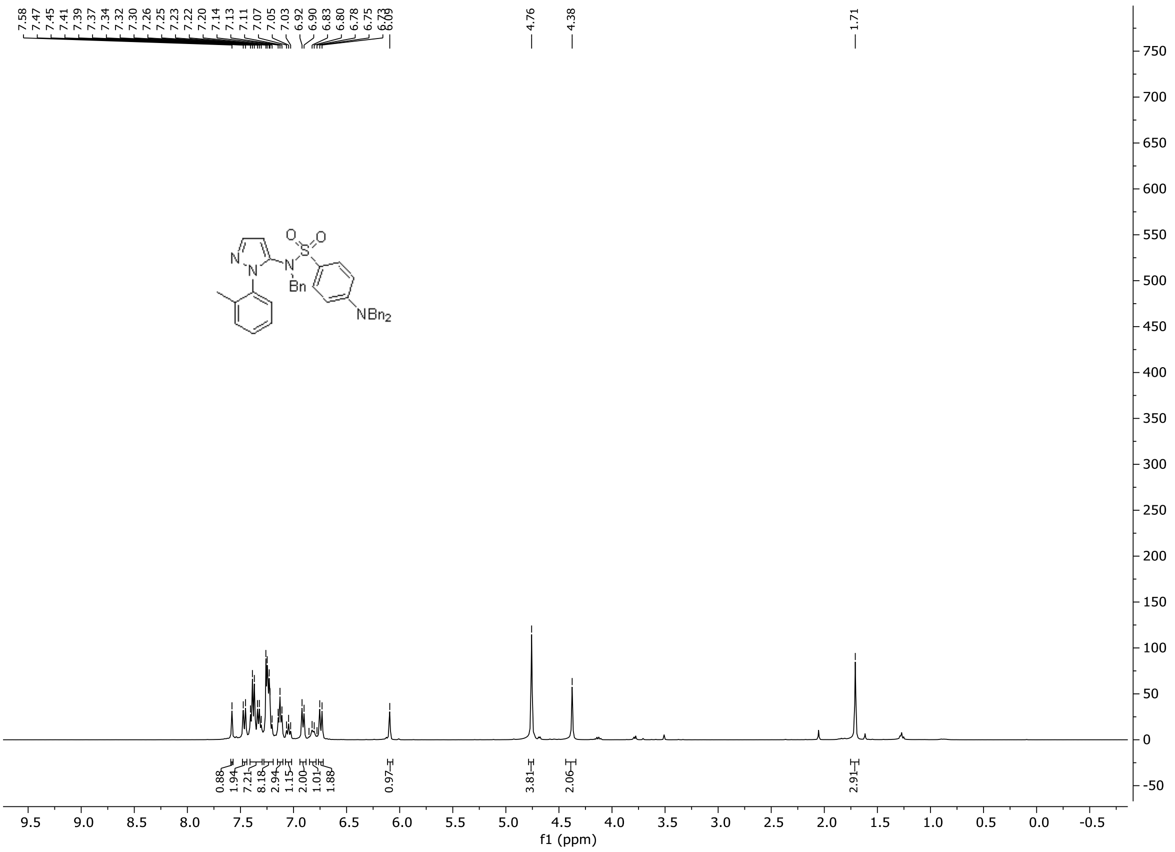
4.76
4.38
1.71
N
N
N
O
S
O
Bn
NBn2
f1 (ppm)

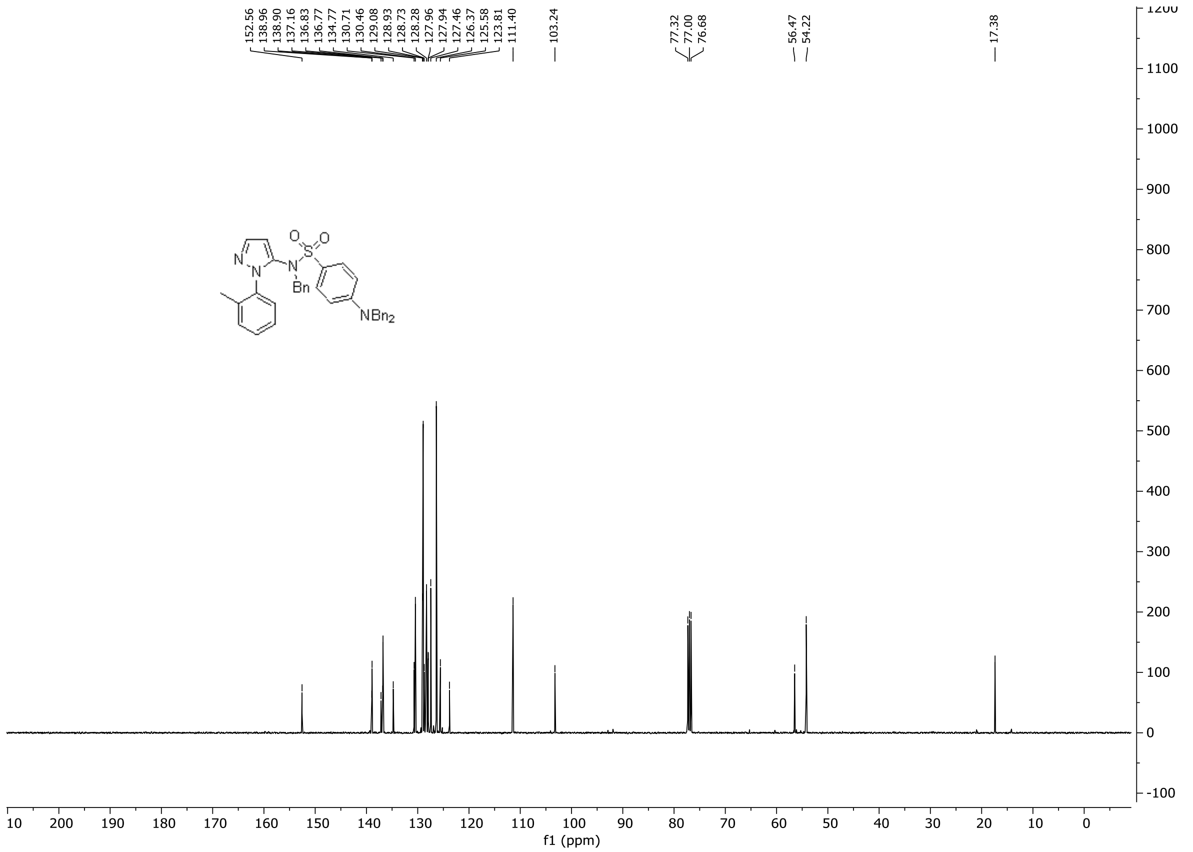
152.56
138.96
138.90
137.16
136.83
136.77
134.77
130.71
130.46
129.08
128.93
128.73
128.28
127.96
127.94
127.46
126.37
125.58
123.81
111.40
103.24
77.32
77.00
76.68
56.47
54.22
17.38
NBn2
Bn
f1 (ppm)

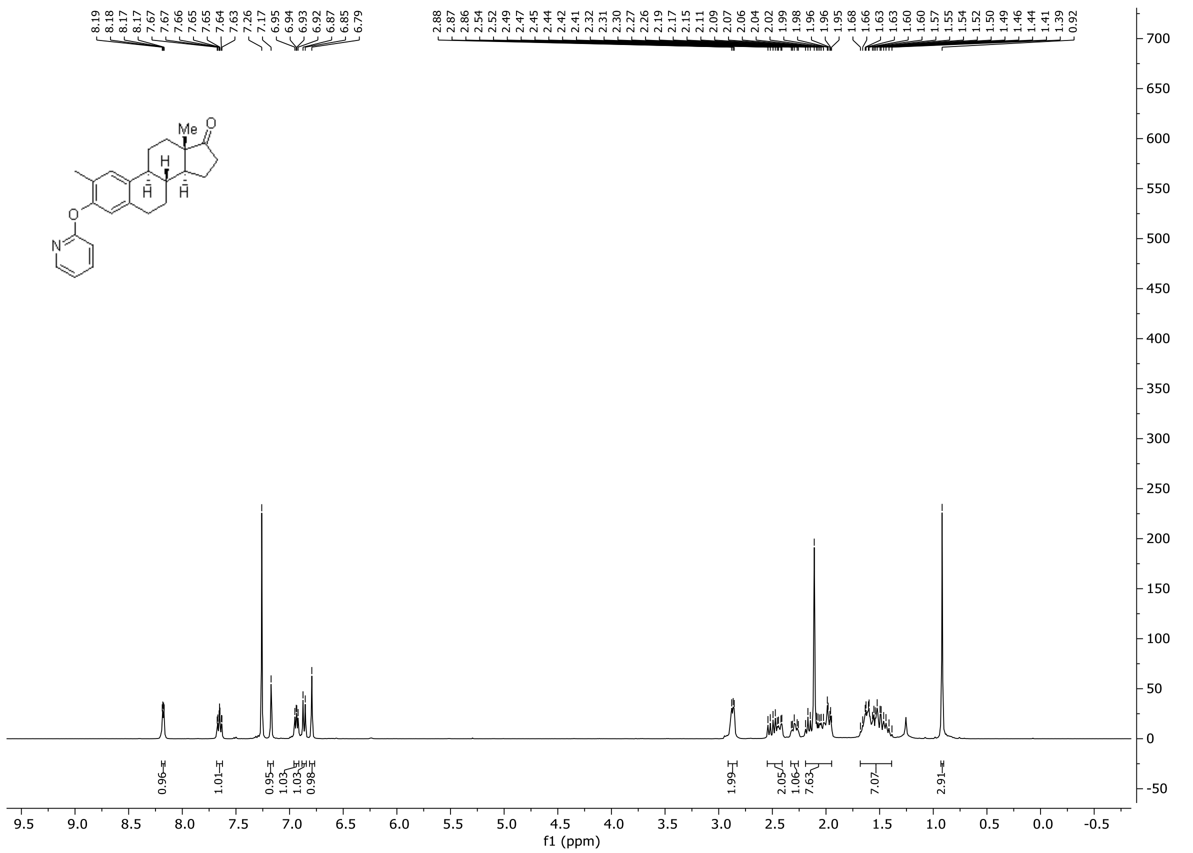
8.19
8.18
8.17
8.17
7.67
7.66
7.65
7.65
7.64
7.63
7.26
7.17
6.95
6.94
6.93
6.92
6.87
6.85
6.79
2.88
2.87
2.86
2.54
2.52
2.49
2.47
2.45
2.44
2.42
2.41
2.32
2.31
2.30
2.27
2.26
2.19
2.17
2.15
2.11
2.09
2.07
2.06
2.04
2.02
1.99
1.98
1.96
1.96
1.95
1.68
1.66
1.63
1.63
1.60
1.60
1.57
1.55
1.54
1.52
1.50
1.49
1.46
1.44
1.41
1.39
0.92
Me
0.96
1.01
0.95
1.03
1.03
0.98
1.99
2.05
1.06
7.63
7.07
2.91
f1 (ppm)

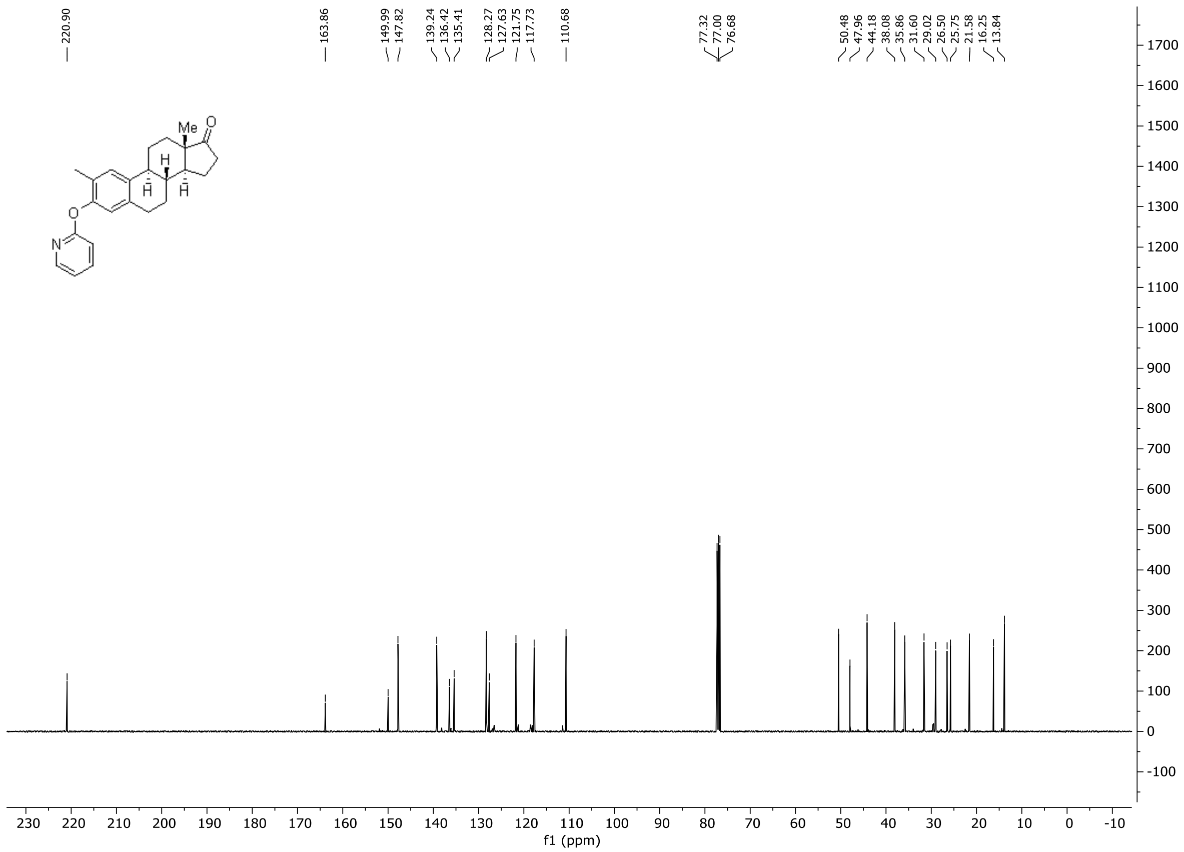
220.90
163.86
149.99
147.82
139.24
136.42
135.41
128.27
127.63
121.75
117.73
110.68
77.32
77.00
76.68
50.48
47.96
44.18
38.08
35.86
31.60
29.02
26.50
25.75
21.58
16.25
13.84
Me
O
H
N
f1 (ppm)

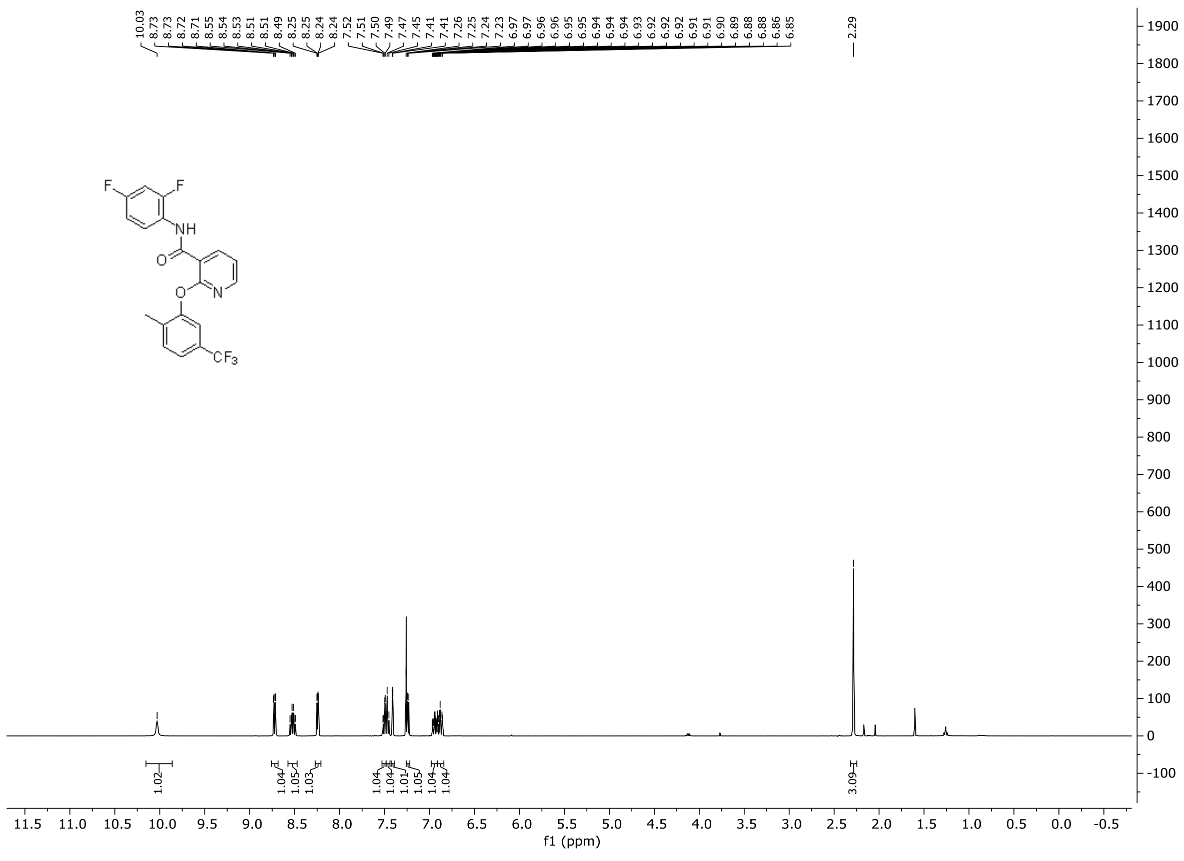
10.03
8.73
8.73
8.72
8.71
8.55
8.54
8.53
8.51
8.49
8.25
8.25
8.24
8.24
7.52
7.51
7.50
7.49
7.47
7.45
7.41
7.41
7.26
7.25
7.24
7.23
6.97
6.97
6.96
6.96
6.95
6.95
6.94
6.94
6.93
6.92
6.92
6.91
6.91
6.90
6.89
6.88
6.88
6.86
6.85
2.29
F
NH
O
N
CF3
1.02
1.04
1.05
1.03
1.04
1.04
1.01
1.05
1.04
1.04
3.09
f1 (ppm)

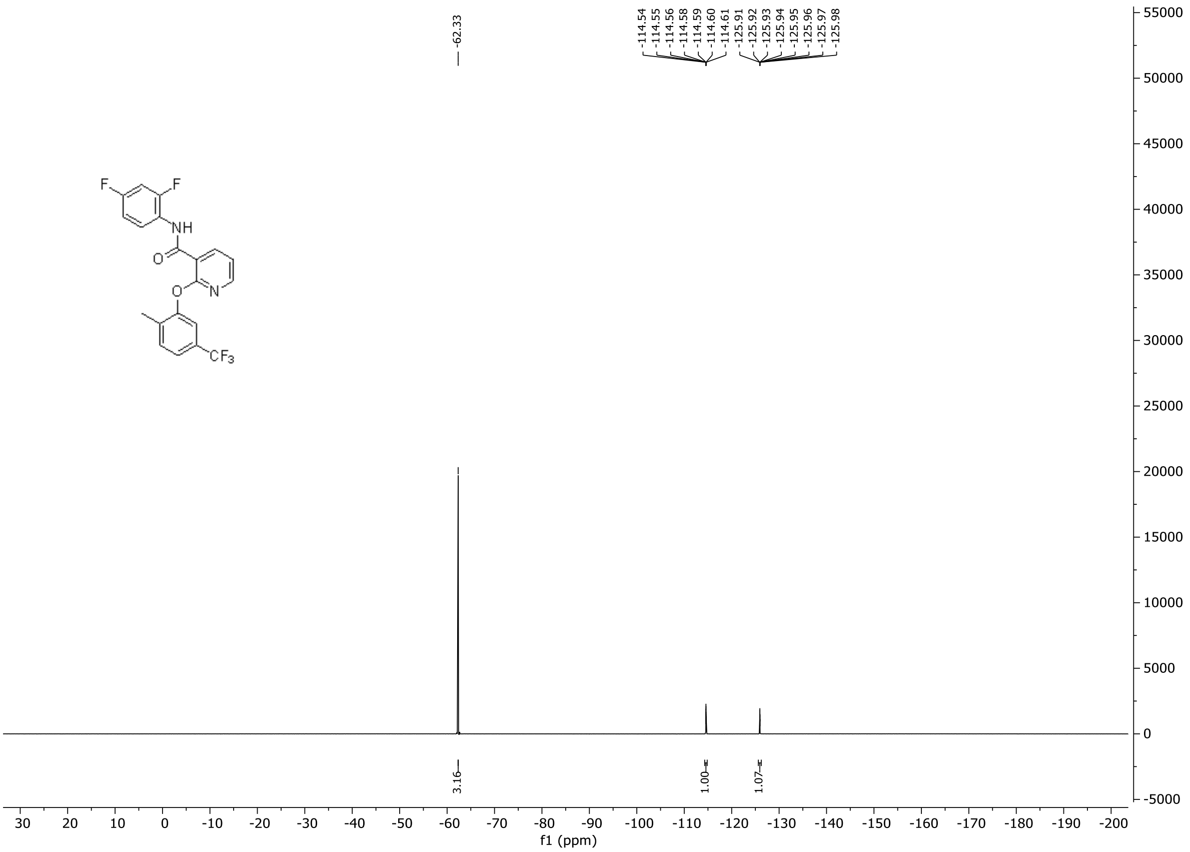
-62.33
-114.54
-114.55
-114.56
-114.58
-114.59
-114.60
-114.61
-125.91
-125.92
-125.93
-125.94
-125.95
-125.96
-125.97
-125.98
3.16
1.00
1.07
f1 (ppm)

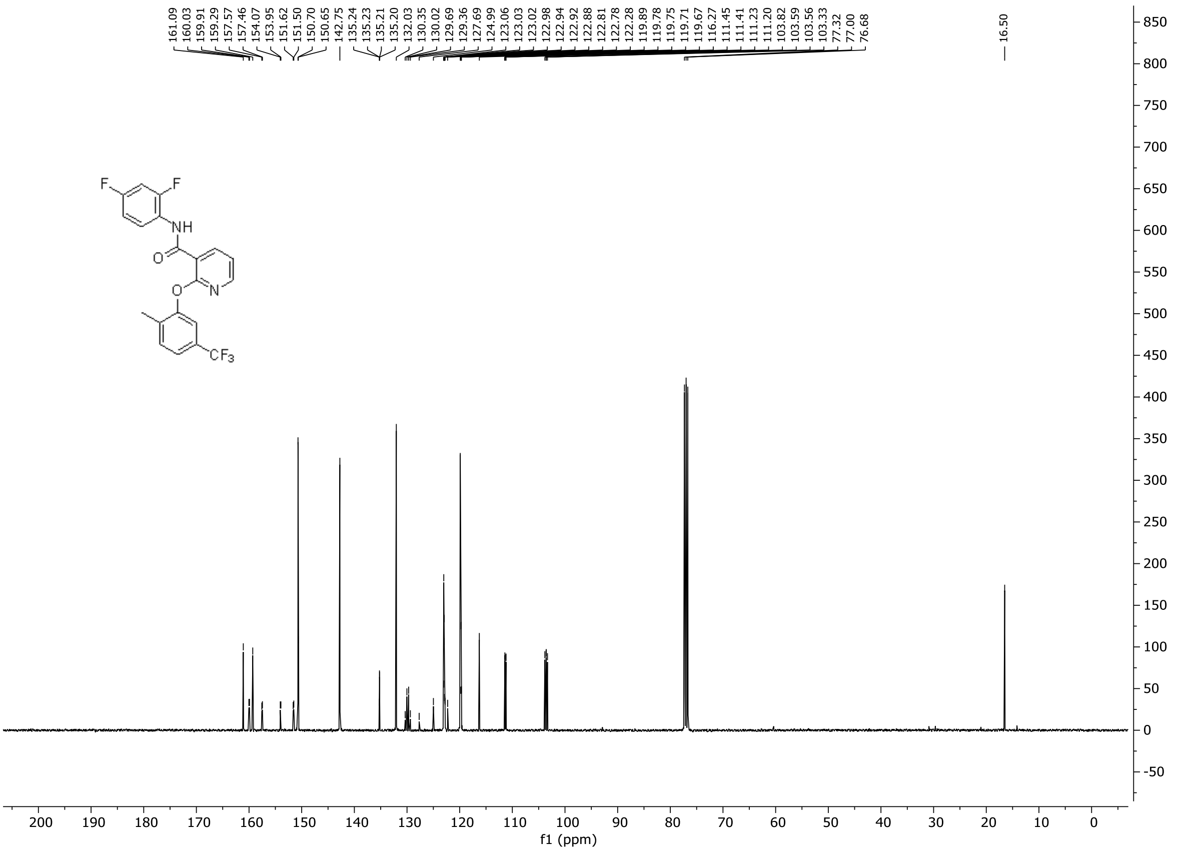
161.09
160.03
159.91
159.29
157.57
157.46
154.07
153.95
151.62
151.50
150.70
150.65
142.75
135.24
135.23
135.21
135.20
132.03
130.35
130.02
129.69
129.36
127.69
124.99
123.06
123.03
123.02
122.98
122.94
122.92
122.88
122.81
122.78
122.28
119.89
119.78
119.75
119.71
119.67
116.27
111.45
111.41
111.23
111.20
103.82
103.59
103.56
103.33
77.32
77.00
76.68
16.50
f1 (ppm)

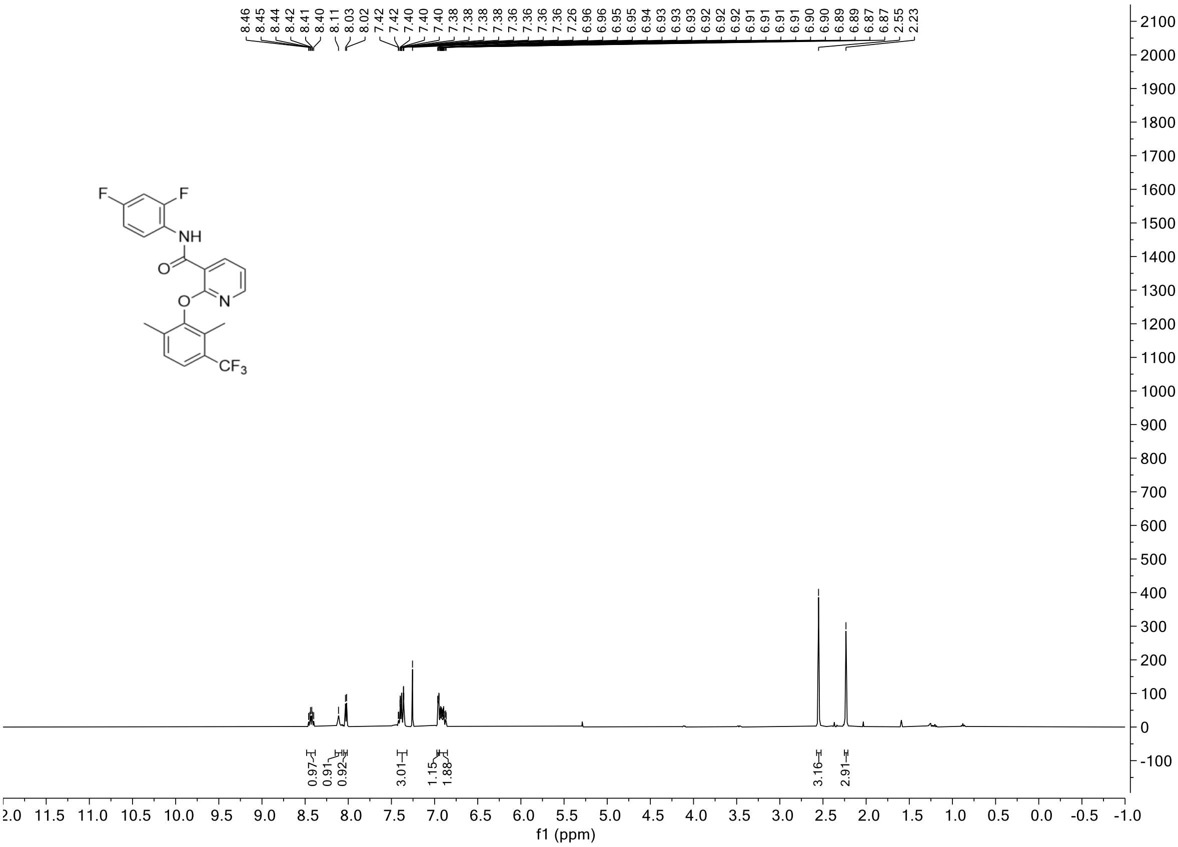
8.46
8.45
8.44
8.42
8.41
8.40
8.11
8.03
8.02
7.42
7.42
7.40
7.40
7.38
7.38
7.38
7.38
7.36
7.36
7.36
7.36
7.26
6.96
6.96
6.95
6.95
6.94
6.93
6.93
6.93
6.92
6.92
6.92
6.91
6.91
6.91
6.91
6.90
6.90
6.89
6.89
6.87
6.87
2.55
2.23
F
F
NH
O
N
O
CF3
0.97
0.91
0.92
3.01
1.15
1.88
3.16
2.91
f1 (ppm)

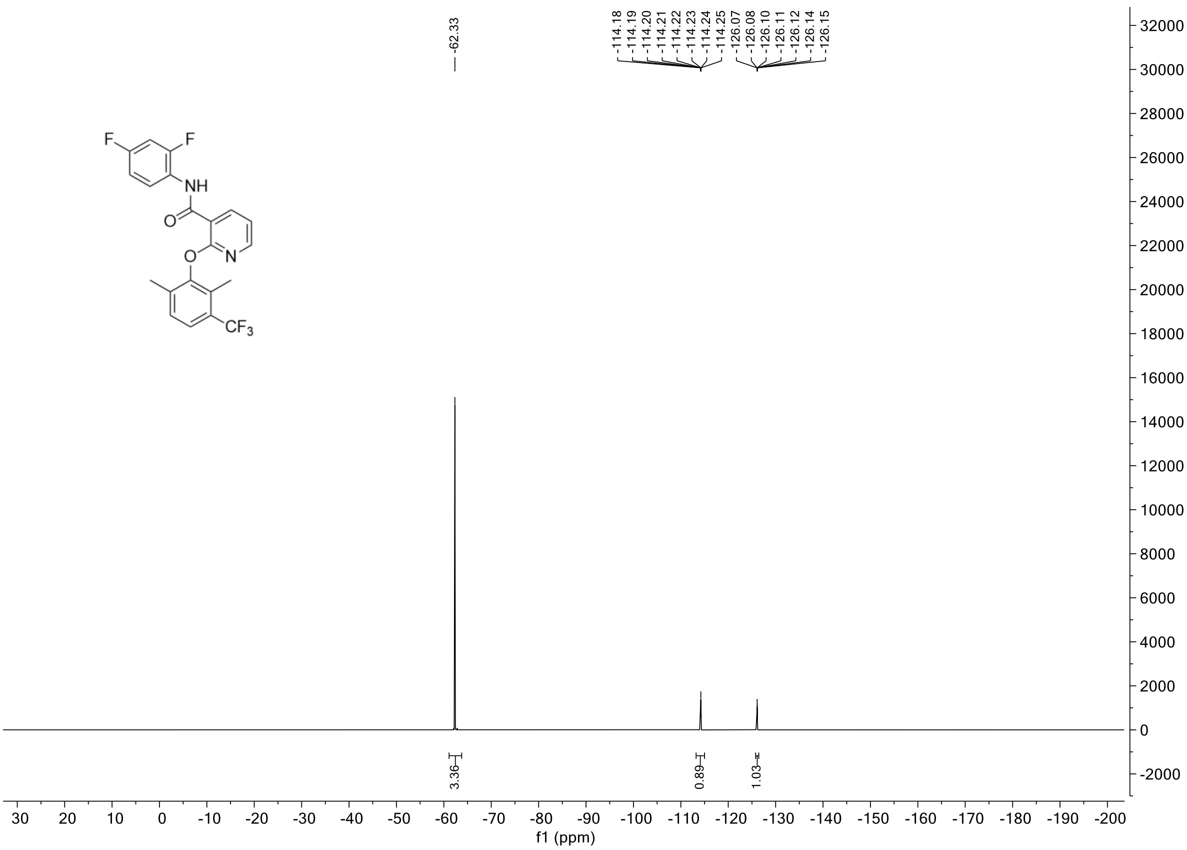
-62.33
-114.18
-114.19
-114.20
-114.21
-114.22
-114.23
-114.24
-114.25
-126.07
-126.08
-126.10
-126.11
-126.12
-126.14
-126.15
F
F
NH
O
N
O
CF3
3.36
0.89
1.03
f1 (ppm)

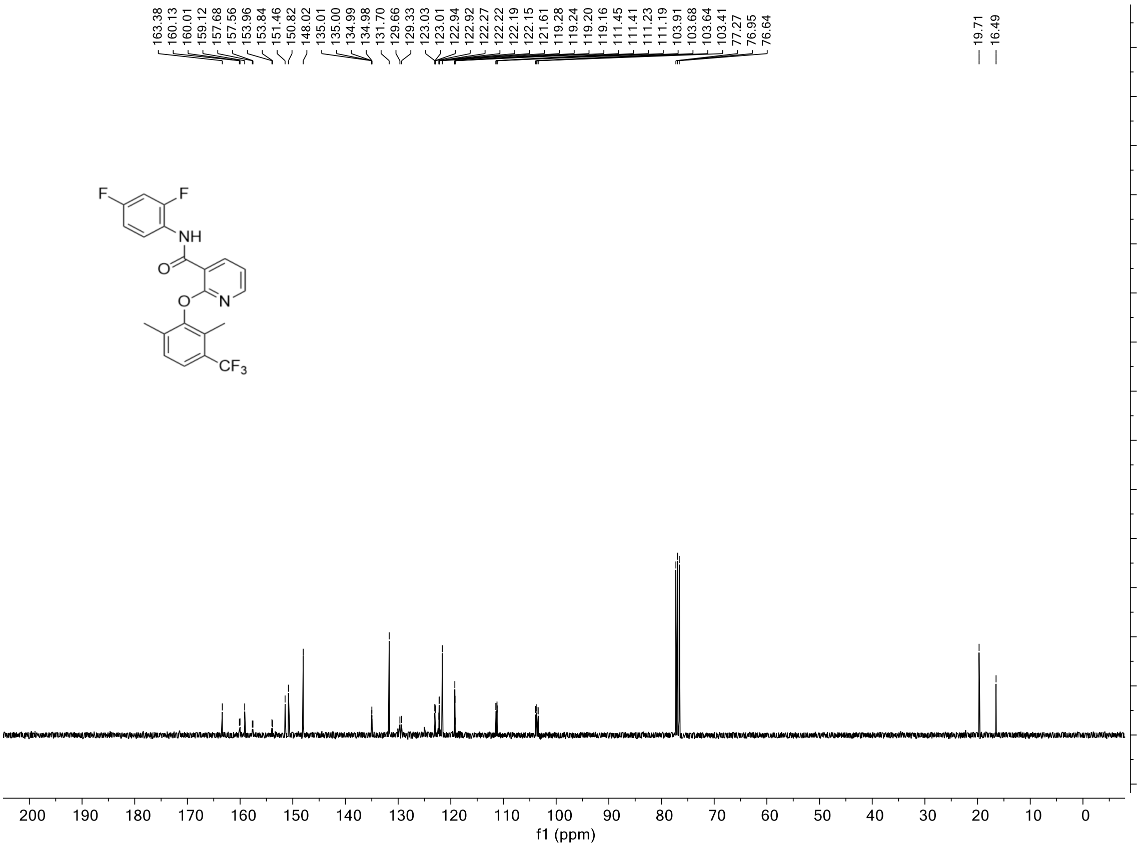
163.38
160.13
160.01
159.12
157.68
157.56
153.96
153.84
151.46
150.82
148.02
135.01
135.00
134.99
134.98
131.70
129.66
129.33
123.03
123.01
122.94
122.92
122.27
122.22
122.19
122.15
121.61
119.28
119.24
119.20
119.16
111.45
111.41
111.23
111.19
103.91
103.68
103.64
103.41
77.27
76.95
76.64
19.71
16.49
F
F
NH
O
O
N
CF3
f1 (ppm)

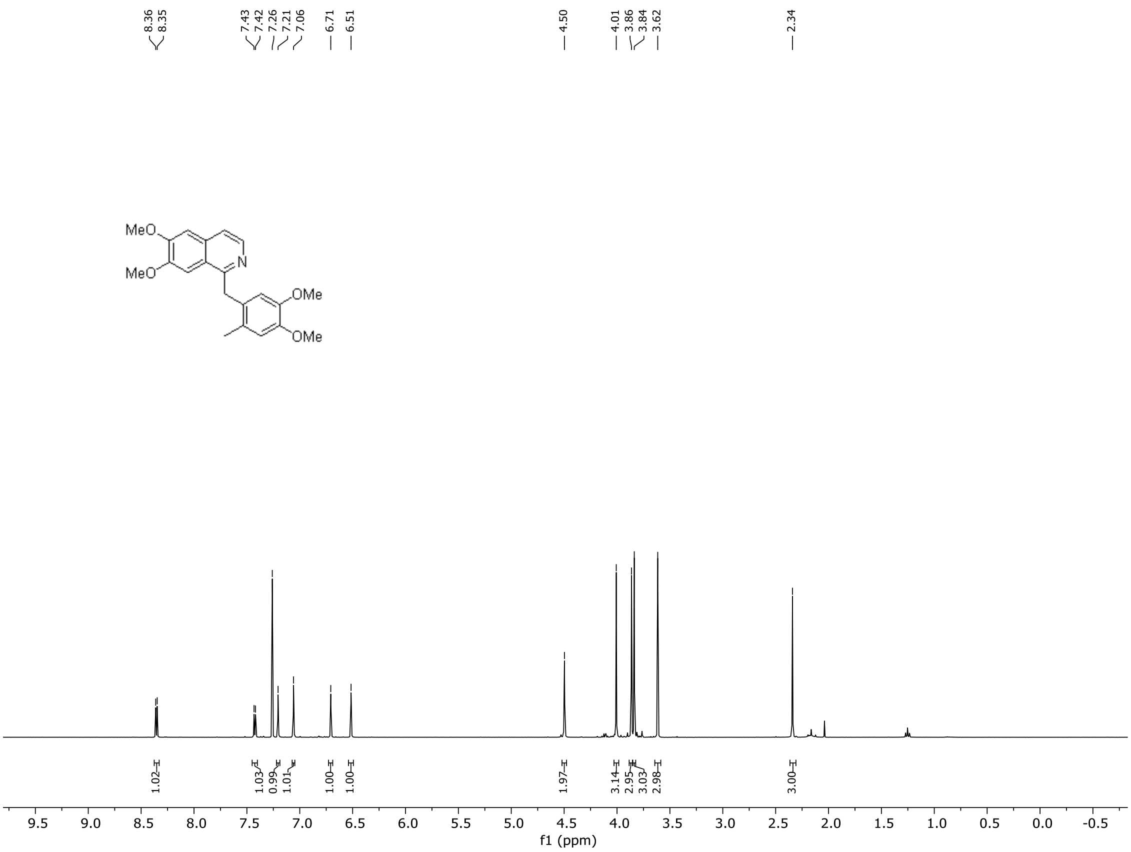
8.36
8.35
7.43
7.42
7.26
7.21
7.06
6.71
6.51
4.50
4.01
3.86
3.84
3.62
2.34
MeO
MeO
N
OMe
OMe
1.02
1.03
0.99
1.01
1.00
1.00
1.97
3.14
2.95
3.03
2.98
3.00
f1 (ppm)

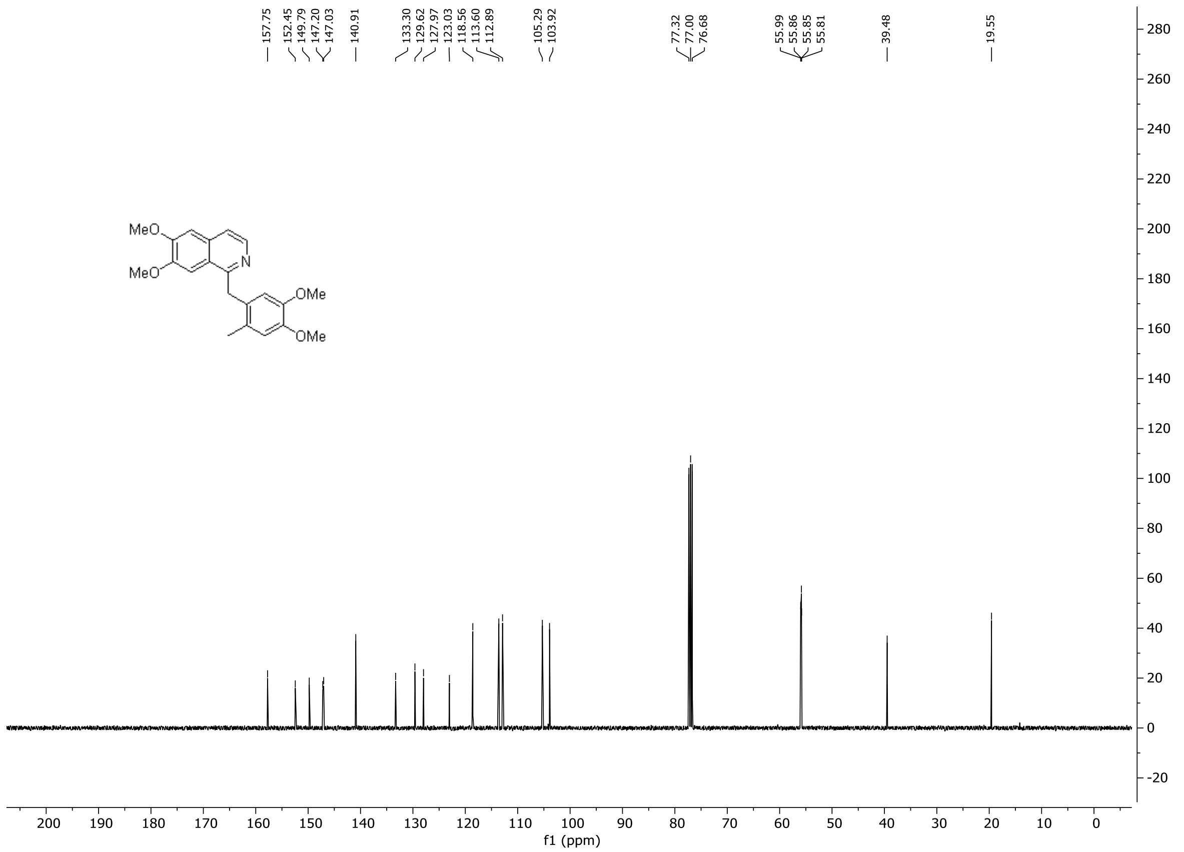
157.75
152.45
149.79
147.20
147.03
140.91
133.30
129.62
127.97
123.03
118.56
113.60
112.89
105.29
103.92
77.32
77.00
76.68
55.99
55.86
55.85
55.81
39.48
19.55
MeO
MeO
N
OMe
OMe
f1 (ppm)

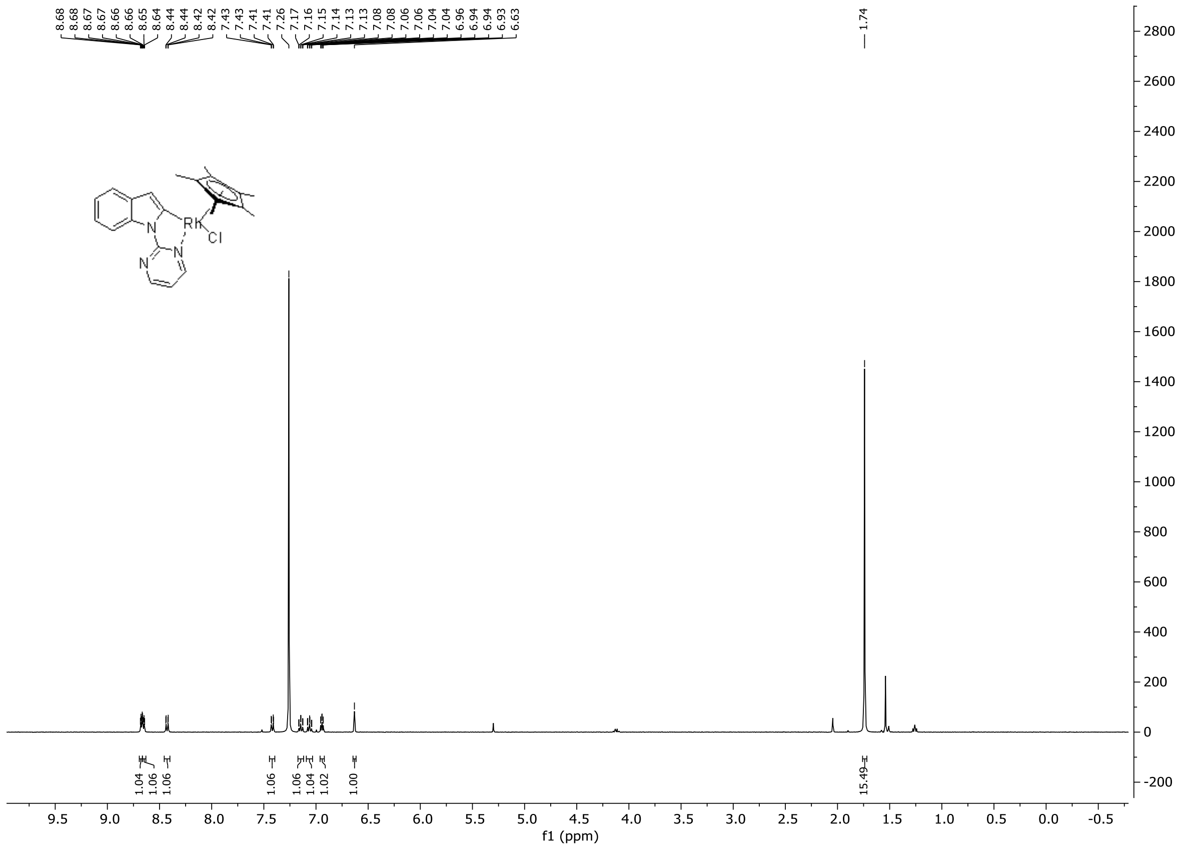
8.68
8.68
8.67
8.67
8.66
8.65
8.64
8.44
8.44
8.42
8.42
7.43
7.43
7.41
7.41
7.26
7.17
7.16
7.15
7.14
7.13
7.13
7.08
7.06
7.06
7.04
7.04
6.96
6.94
6.94
6.93
6.63
1.74
N
Rh
Cl
N
N
1.04
1.06
1.06
1.06
1.06
1.04
1.02
1.00
15.49
f1 (ppm)

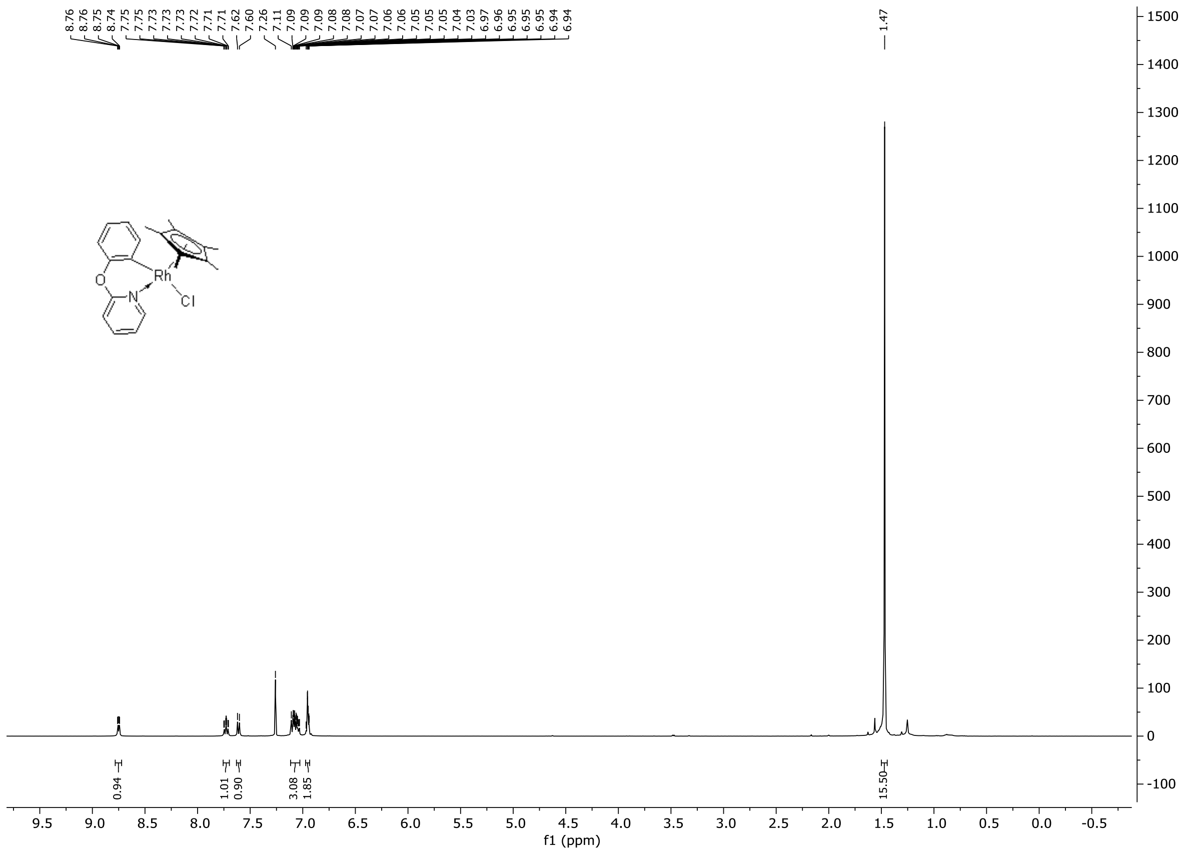
8.76
8.76
8.75
8.74
7.75
7.75
7.73
7.73
7.73
7.72
7.71
7.71
7.62
7.60
7.26
7.11
7.09
7.09
7.09
7.08
7.08
7.07
7.07
7.06
7.06
7.05
7.05
7.05
7.04
7.03
6.97
6.96
6.95
6.95
6.95
6.94
6.94
1.47
Rh
O
N
Cl
0.94
1.01
0.90
3.08
1.85
15.50
f1 (ppm)

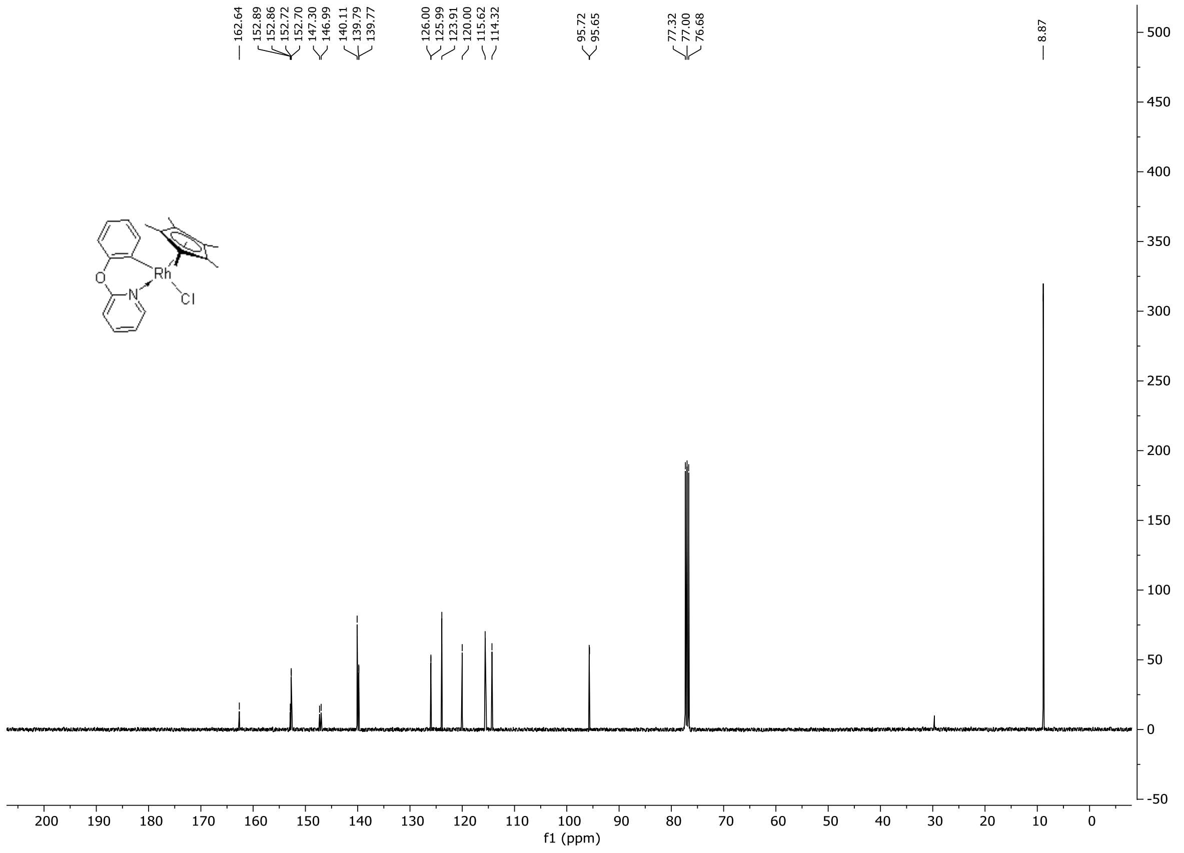
162.64
152.89
152.86
152.72
152.70
147.30
146.99
140.11
139.79
139.77
126.00
125.99
123.91
120.00
115.62
114.32
95.72
95.65
77.32
77.00
76.68
8.87
Rh
O
N
Cl
f1 (ppm)

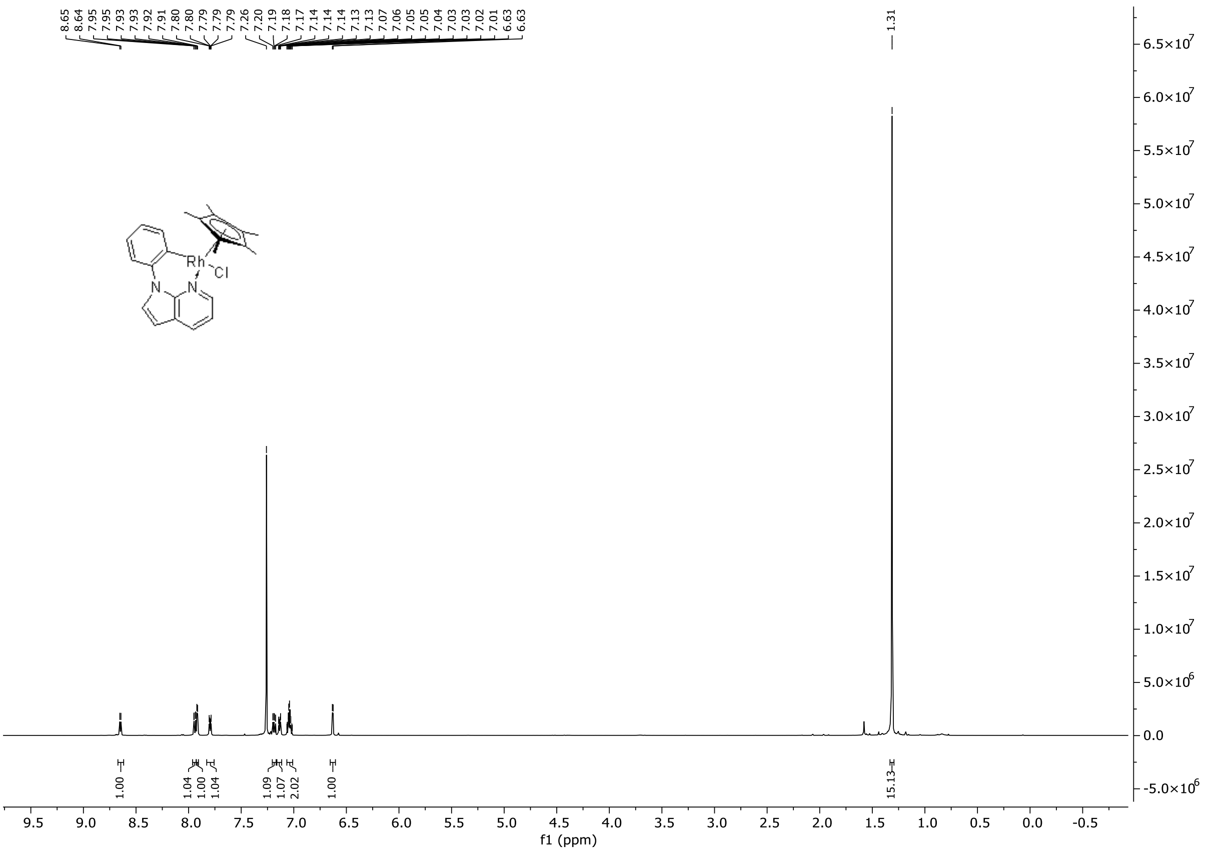
8.65 8.64 7.95 7.95 7.93 7.93 7.92 7.91 7.80 7.79 7.79 7.79 7.26 7.20 7.19 7.18 7.17 7.14 7.14 7.14 7.13 7.13 7.07 7.06 7.05 7.05 7.04 7.03 7.03 7.02 7.01 6.63 6.63
1.31
Rh
Cl
N
1.00 1.04 1.00 1.04 1.09 1.07 2.02 1.00 15.13
f1 (ppm)

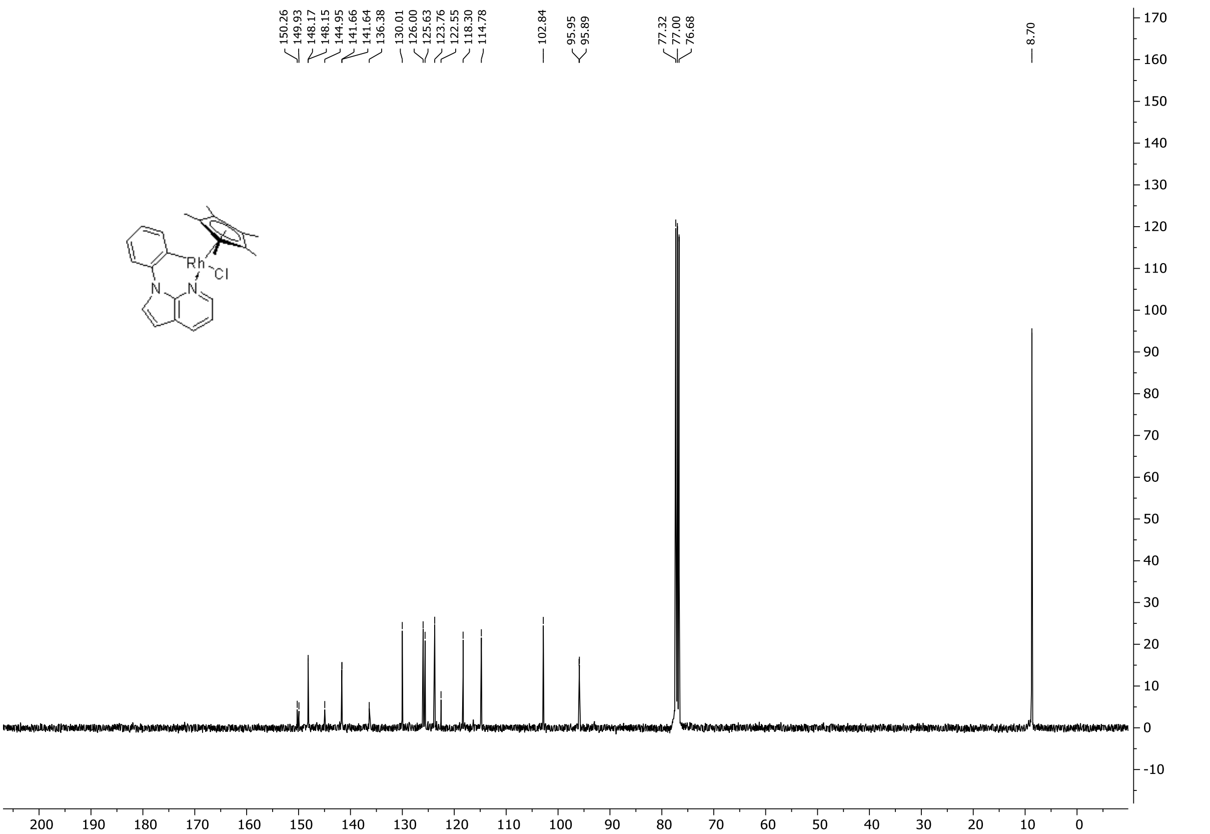
150.26 149.93 148.17 148.15 144.95 141.66 141.64 136.38 130.01 126.00 125.63 123.76 122.55 118.30 114.78
102.84
95.95 95.89
77.32 77.00 76.68
8.70
Rh
Cl
N
f1 (ppm)

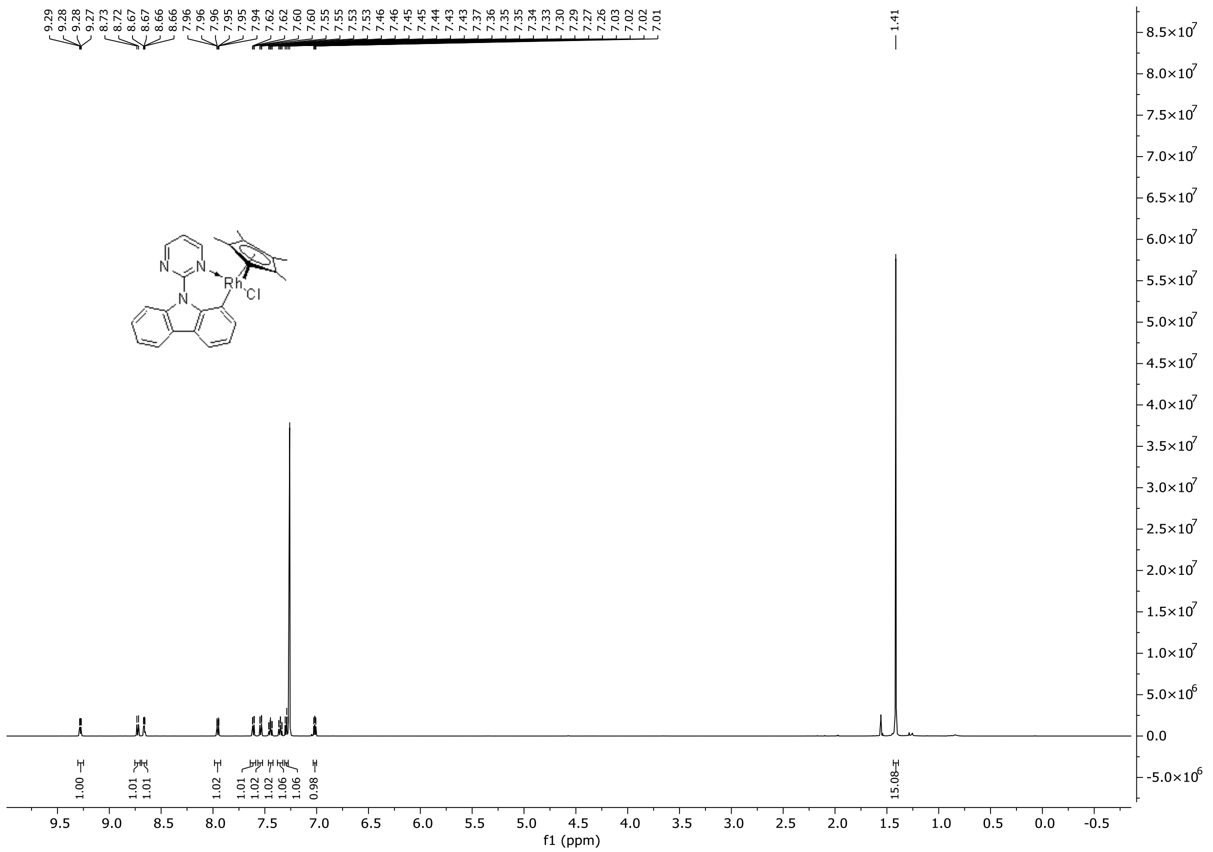

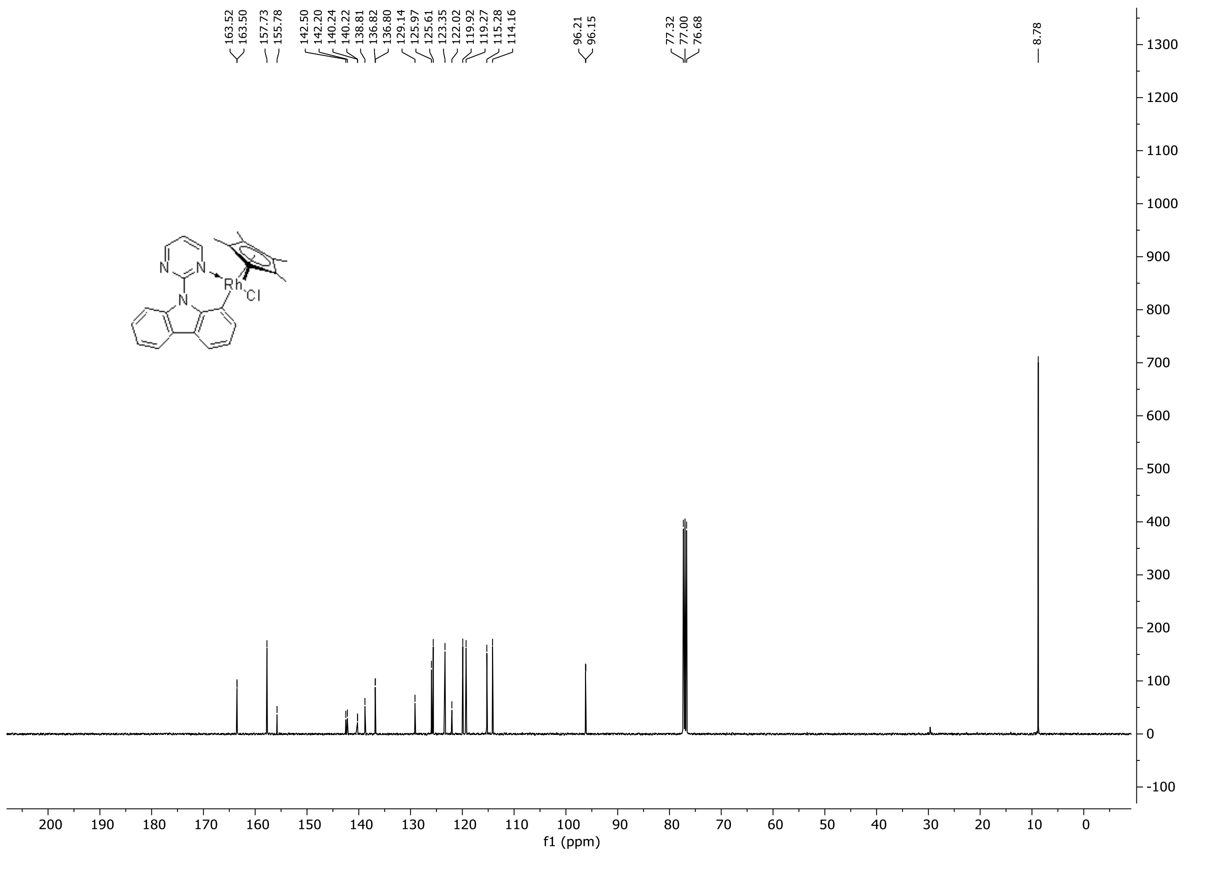

Supplement: Supplementary file 1 — Supplementary [file ANIE-60-6660-s001.pdf]
